# Plasma MicroRNAs as Potential Noninvasive Biomarkers for In-Stent Restenosis

**DOI:** 10.1371/journal.pone.0112043

**Published:** 2014-11-26

**Authors:** Meijiao He, Yongtai Gong, Jing Shi, Zhenwei Pan, Hui Zou, Danghui Sun, Xin Tu, Xiangyang Tan, Jianqiang Li, Weimin Li, Bin Liu, Jingyi Xue, Li Sheng, Chunhong Xiu, Ning Yang, Hongjie Xue, Xue Ding, Chengyuan Yu, Yue Li

**Affiliations:** 1 Cardiovascular Department, the First Affiliated Hospital of Harbin Medical University, Harbin, Heilongjiang Province, P.R. China; 2 Department of Pharmacology, Harbin Medical University, Harbin, Heilongjiang Province, P.R. China; 3 Cardiovascular Department, the Hospital of Heilongjiang Province, Harbin, Heilongjiang Province, P.R. China; 4 Life Science and Technology College of Huazhong University of Science and Technology, Wuhan, Hubei Province, P.R. China; 5 Wyeth Research, Cambridge, MA, United States of America; University of Massachusetts Medical, United States of America

## Abstract

**Objective:**

To investigate whether microRNAs (miRs) can serve as novel biomarkers for in-stent restenosis (ISR).

**Methods:**

This retrospective, observational single-centre study was conducted at the cardiovascular department of a tertiary hospital centre in the north of China. Follow-up coronary angiography at 6 to 12 months was performed in 181 consecutive patients implanted with drug-eluting stents. Fifty-two healthy volunteers served as the control group. The plasma miRs levels were analyzed by quantitative real-time PCR. Receiver-operating characteristic curve (ROC) analysis was performed to investigate the characters of these miRs as potential biomarkers of ISR.

**Results:**

MiR-21 levels in ISR patients were significantly higher than those in non-ISR patients and healthy controls (*P*<0.05), while miR-100 (*P*<0.05), miR-143 (*P*<0.001) and miR-145 (*P*<0.0001) levels were significantly decreased in ISR patients. Further analysis showed that miR-21 levels were remarkably increased (*P* = 0.045), while miR-100 (*P* = 0.041), miR-143 (*P* = 0.029) and miR-145 (*P*<0.01) levels were dramatically decreased in patients with diffuse ISR compared to those with focal ISR. ROC analysis demonstrated that the area under curve of miR-145, miR-143, miR-100 and miR-21 were 0.880 (95% confidence interval; CI = 0.791–0.987, *P*<0.001), 0.818 (95% confidence interval; CI = 0.755–0.963, *P*<0.001), 0.608 (95% confidence interval; CI = 0.372–0.757, *P*<0.05) and 0.568 (95% confidence interval; CI = 0.372–0.757, *P*<0.05), with specificity of 83.1%, 80.1%, 68.9% and 68.6%, and sensitivity of 88.7%, 82.1%, 60.2% and 50.1%, respectively.

**Conclusions:**

Circulating miR-143 and miR-145 levels are associated with the occurrence of ISR and can serve as novel noninvasive biomarkers for ISR.

## Introduction

Despite significant advances in technology, bioengineering and pharmacology in recent years, ISR after stent implantation is still the most troublesome problem in coronary intervention treatment. The introduction of drug-eluting stents (DES) has significantly reduced the incidence of ISR as compared to bare metal stents. However, ISR still occurs in 7% to 13% of patients receiving DES [1].

Increasing evidence indicate that ISR is mainly attributed to excessive proliferation of vascular smooth muscle cells (VSMCs) [2]. MicroRNAs are non-coding, small RNAs (∼22-nucleotide) that regulate gene expression at a post-transcriptional level through translational repression or mRNA decay [3]. Recent studies have demonstrated that miRs are abundantly expressed in vascular tissues and play important roles in vascular dysfunction, ischemic angiogenesis, reendothelialization, and vascular restenosis via regulating key vascular cellular events through modulating the expression of their target genes [4]–[5]. Ji et al. [6] demonstrated that miR-21 is one of the most up-regulated miRs in the vascular wall after balloon injury, and promotes VSMCs proliferation via activation of Akt and Bcl-2 with concomitant inhibition of phosphatase and tensin homolog. Antisense knockdown of miR-21 blunts the formation of a neointimal lesions in response to balloon injury of the rat carotid artery [6]. A recent study indicated that miR-221 and miR-222 are novel regulators for VSMCs proliferation and neointimal hyperplasia in balloon-injured rat carotid arteries [7]. Knockdown of miR-221 and miR-222 can suppress VSMCs proliferation and neointimal lesion formation after angioplasty in vivo. Cheng et al. [8] found that miR-145 expression is significantly down-regulated in the injured vascular walls and restoration of miR-145 inhibits VSMCs proliferation and reduces neointima formation in response to balloon injury. In addition, miR-143 and miR-145 also participate in the development of cardiovascular diseases by modulating smooth muscle cell phenotypic marker and controlling cell proliferation [9]. In recent years, it was reported that miR-100 affects endothelial cells proliferation and VSMCs migration [10].

Interestingly, recent studies have revealed that miRs are able to be released into circulating blood from the injured cells and tissues. And the cell-free miRs remain relatively stable due to binding with other materials such as microvesicles and exosomes in circulating blood [11]–[12]. Thus, circulating miRs are currently explored for their potential as biomarkers in cancers [13]–[15] and a wide range of cardiovascular diseases [16]–[18]. For example, recent studies have demonstrated that circulating miRs could be used as biomarkers for acute myocardial infarction [16], post-ischemic cardic remodeling [17] and heart failure [18] in humans.

In light of these findings, we measured the levels of miRs in patients with and without ISR and assessed their value as potential molecular markers for ISR in this study.

## Methods

### Study subjects

From December 2008 to October 2012, 181 consecutive patients were selected from the First Affiliated Hospital of Harbin Medical University for this study with the following inclusion criteria: a. patients received DES at the initial PCI; follow-up coronary angiography at 6 to 12 months was performed; b. patients received aspirin 100 mg/day and clopidogrel 75 mg/day. Patients with a total occluded artery or acute myocardial infarction were excluded [19]. Fifty-two healthy volunteers without any evidence of coronary artery disease or inflammatory disorders served as the control group. ISR was defined as the presence of ≥50% diameter stenosis in the stented segment [20]–[21]. All angiographic images were obtained with a digital flat-panel cardiac imaging system (Allura Xper FD 10, Philips Medical Systems; Innova 2000, GE and Innova 2100, GE). Quantitative coronary analysis was performed using a validated detection system (AngioSYS, China). Minimal luminal diameter, reference vessel diameter and percentage of diameter stenosis were assessed. The patterns of restenosis were classified according to the length of the lesion: focal (≤10 mm) or diffuse (>10 mm) [22]. The study followed the principals outlined in the Declaration of Helsinki and had been approved by the ethics committee of the First Affiliated Hospital of Harbin Medical University. All participants provided written informed consent.

We had deposited raw data of the study to the publicly available database (www.medresman.org, ChiCTR-OCH-14004263).

### Plasma collection

Blood samples for miRNAs detection were collected from the forearm veins of patients via a direct venous puncture into vacutainer tubes containing Ethylene Diamine Tetraacetic Acid (EDTA) in the ward or the cardiac catheterization laboratory before the angiography procedure and heparin administration at the time of follow up coronary angiography (CAG). Plasma samples were collected by centrifugation (15 minutes at 1200 g) within 30 minutes following peripheral blood draw from all patients, and were transferred into RNase-free tubes for extraction of RNA.

### RNA isolation

Total RNA in plasma was isolated by using Trizol LS reagent, phenol and chloroform with extraction procedures. Briefly, 1 mL plasma was lysed with Trizol LS (Invitrogen, 10296-010) in 1∶3 ratio. After 5 min, 0.8 mL of chloroform per 1 mL of sample was added and the samples were shaken vigorously for 15 sec, incubated for 5 min and then centrifuged at 12,000 g for 15 min at 4°C. The aqueous phase was then transferred into a fresh tube and 2.0 mL of isopropanol per 1 mL of sample was added and incubated for 10 min. Total RNA was precipitated after samples were centrifuged at 12,000 g for 10 min at 4°C. The supernatant was removed and the RNA pellet was washed with 1 mL of 75% ethanol and subsequently centrifuged at 7,500 g for 5 min at 4°C. After removing the ethanol, RNA pellet was briefly air-dried and dissolved in RNase-free water. RNA concentration was determined by Eppendorf Biophotometer system (Germany) and the yield was around 0.2 µg for plasma. The RNA samples were heated to 70°C for 10 min and rapidly placed in ice for subsequent use. The samples had an OD260/280 ratio between 1.8 and 2.0, and an OD260/230 ratio between 2.0 and 2.2. The value of RNA concentration was between 300 ng/ul to 500 ng/ul. cDNA synthesis was completed according to the protocol of the manufacturer (Reverse Transcription System, Promega) as described before [17], [23].

### MiRNA Profile

In this study, we carried out a 2-stage study for ISR in Chinese patients. In stage 1 (Discovery study), we used illumina human microRNA expression 12-sample Universal BeadChip to discover the potential ISR-related circulating miRs. The discovery study enrolled 6 ISR patients and 4 non-ISR patients. Hybridizing was performed by commercial company (Beijing Compass Biotechnology Co., Ltd, China) following standard experimental procedures from the manufacturer (Illumina). The result was analyzed by the software of Genome Studio (GenomeStudioV2009.1_ Installaction).The software of Gene Cluster 3.0 & TreeView was used to cluster analysis. Data are available at the Gene Expression Omnibus (http://www.ncbi.nlm.nih.gov/geo/) under the accession number GSE 60959.

In stage 2 (Replication study), we carried out a replication study to validate the level of circulating miRs by quantitative real-time PCR (QRT-PCR) in 51 ISR patients and 130 non-ISR patients. QRT-PCR was finished with SYBR Green PCR Master Mix Kit (Applied Biosystems) for relative quantification of miRs on Real-Time PCR System (7500 FAST, Applied Biosystems). The RT primers, forward and reverse primer sequences were summarized in Table 1. All samples were performed in triplicate and the Ct value was determined using the fixed threshold setting. The intensity level crossed the Ct was used to compare individual reaction. U6 showed no significant variation in intensity level among our samples. Therefore, miRNA values were normalized to U6 and were expressed as 2^−(CT[microRNA]-CT[U6]^ [24]. We calculated the relative changes of miRs expression by the 2^−ΔCt^.

**Table 1:** Primers sequence.

Primer name	Sequence (5′-3′)
**U6 RT**	CGCTTCACGAATTTGCGTGTCAT
**U6 FP**	GCTTCGGCAGCACATATACTAAAAT
**U6 RP**	CGCTTCACGAATTTGCGTGTCAT
**miR-21RT**	GTCGTATCCAGTGCGTGTCGTGGAGTCGGCAATTGCACTGGATACGACTCAACATC
**miR-21 FP**	GGGGTAGCTTATCAGACTGATG
**miR-21 RP**	TGTCGTGGAGTCGGCAATTG
**miR-31 RT**	GTCGTATCCAGTGCGTGTCGTGGAGTCGGCAATTGCACTGGATACGACAGCTATG
**miR-31 FP**	GCGAGGCAAGATGCTGGC
**miR-31 RP**	GTGCGTGTCGTGGAGTCG
**miR-100 RT**	GTCGTATCCAGTGCGTGTCGTGGAGTCGGCAATTGCACTGGATACGACCACAAG
**miR-100 FP**	GCAACCCGTAGATCCGAAC
**miR-100 RP**	TCGTGGAGTCGGCAATTGC
**miR-125b RT**	GTCGTATCCAGTGCGTGTCGTGGAGTCGGCAATTGCACTGGATACGACTCACAAG
**miR-125b FP**	GCTCCCTGAGACCCTAAC
**miR-125b RP**	AGTGCGTGTCGTGGAGTC
**miR-130a RT**	GTCGTATCCAGTGCGTGTCGTGGAGTCGGCAATTGCACTGGATACGACATGCCC
**miR-130a FP**	GGCGCAGTGCAATGTTAAAAG
**miR-130a RP**	TGTCGTGGAGTCGGCAATT
**miR-143 RT**	GTCGTATCCAGTGCGTGTCGTGGAGTCGGCAATTGCACTGGATACGACGAGCTAC
**miR-143 FP**	GGGTGAGATGAAGCACTGTAG
**miR-143 RP**	TGTCGTGGAGTCGGCAATTG
**miR-145 RT**	GTCGTATCCAGTGCGTGTCGTGGAGTCGGCAATTGCACTGGATACGACAGGGATTC
**miR-145 FP**	GTCCAGTTTTCCCAGGAATCC
**miR-145 RP**	TGTCGTGGAGTCGGCAATTG
**miR-146a RT**	GTCGTATCCAGTGCGTGTCGTGGAGTCGGCAATTGCACTGGATACGACAACCCAT
**miR-146a FP**	GGCGTGAGAACTGAATTCCA
**miR-146a RP**	TCGTGGAGTCGGCAATTG
**miR-210 RT**	GTCGTATCCAGTGCGTGTCGTGGAGTCGGCAATTGCACTGGATACGACTCAGCC
**miR-210 FP**	GCTGTGCGTGTGACAGCG
**miR-210 RP**	TGCGTGTCGTGGAGTCGG
**miR-221 RT**	GTCGTATCCAGTGCAGGGTCCGAGGTATTCGCACTGGATACGACGAAACC
**miR-221 FP**	CGAGCTACATTGTCTGCTGGGT
**miR-221 RP**	GTGCAGGGTCCGAGGT

RT, RT primers; FP, forward primer; RP, reverse primer.

### Screening of target gene

Target genes of miRNAs were predicted using the two databases miRecords [25] and miRTarBase [26].

### Protein interaction network

Using STRING to search all the interactions of differentially expressed genes and construct the interacting network. Coexpression and Occurrence analysis for this protein was obtained from STRING database.

### Statistical analysis

Data were described as mean ± SD and percentages for general characteristics of subjects. Mann-Whitney test was used for the comparison between two groups and Kruskal-Wallis test was used to compare the expression level among more than two groups. Logistics regression analyses were taken to evaluate risk factors of ISR. ROC analysis was established for discriminating ISR patients from non-ISR patients. The microarray data were normalized by the quantile normalization. The data obtained by microRNA microarray profiling were translated in log 2 (relative level) and statistical difference between groups (p<0.01; t-test). SPSS 16.0 was used for all statistical analyses. P-values were two-sided and less than 0.05 was considered statistically different.

## Results

### Clinical characteristics of subjects

**Figure 1:**
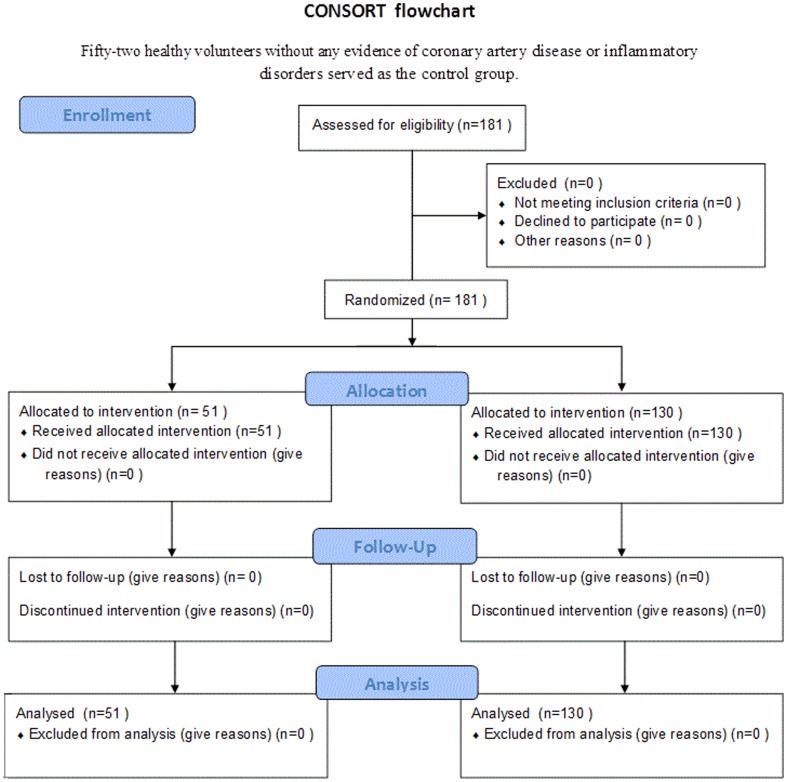
Consort flowchart.

**Figure 2:**
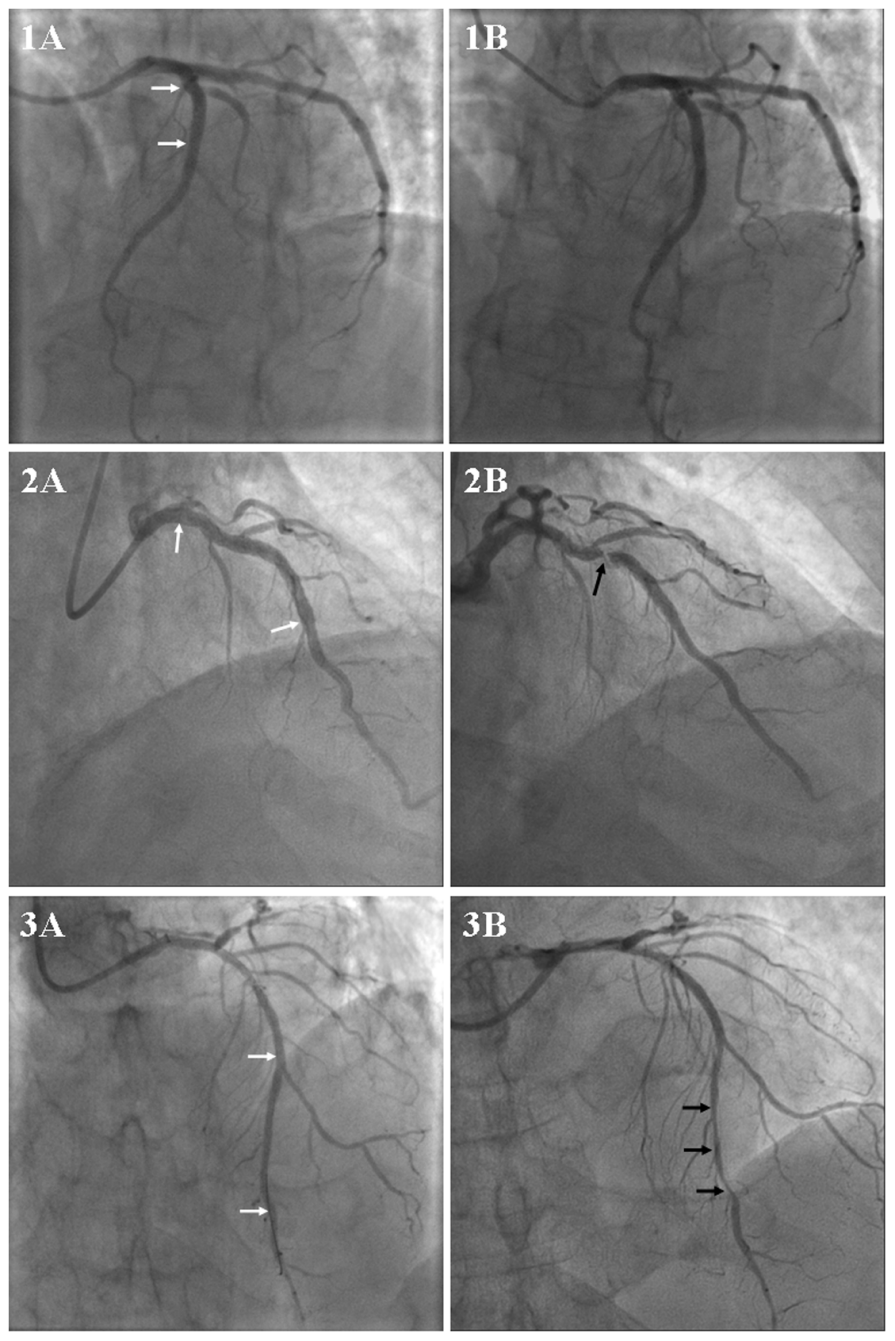
Representative angiographic images of non-ISR, focal and diffuse ISR. Angiographic results after successful percutaneous coronary intervention with stent implantation in the left anterior descending coronary artery from three different patients (1A, 2A and 3A). Follow-up angiography showed non-ISR (1B), focal ISR (2B) and diffuse ISR (3B) respectively. White arrows indicate the margins of stent. Black arrows indicate the stenotic lesions.

**Figure 3:**
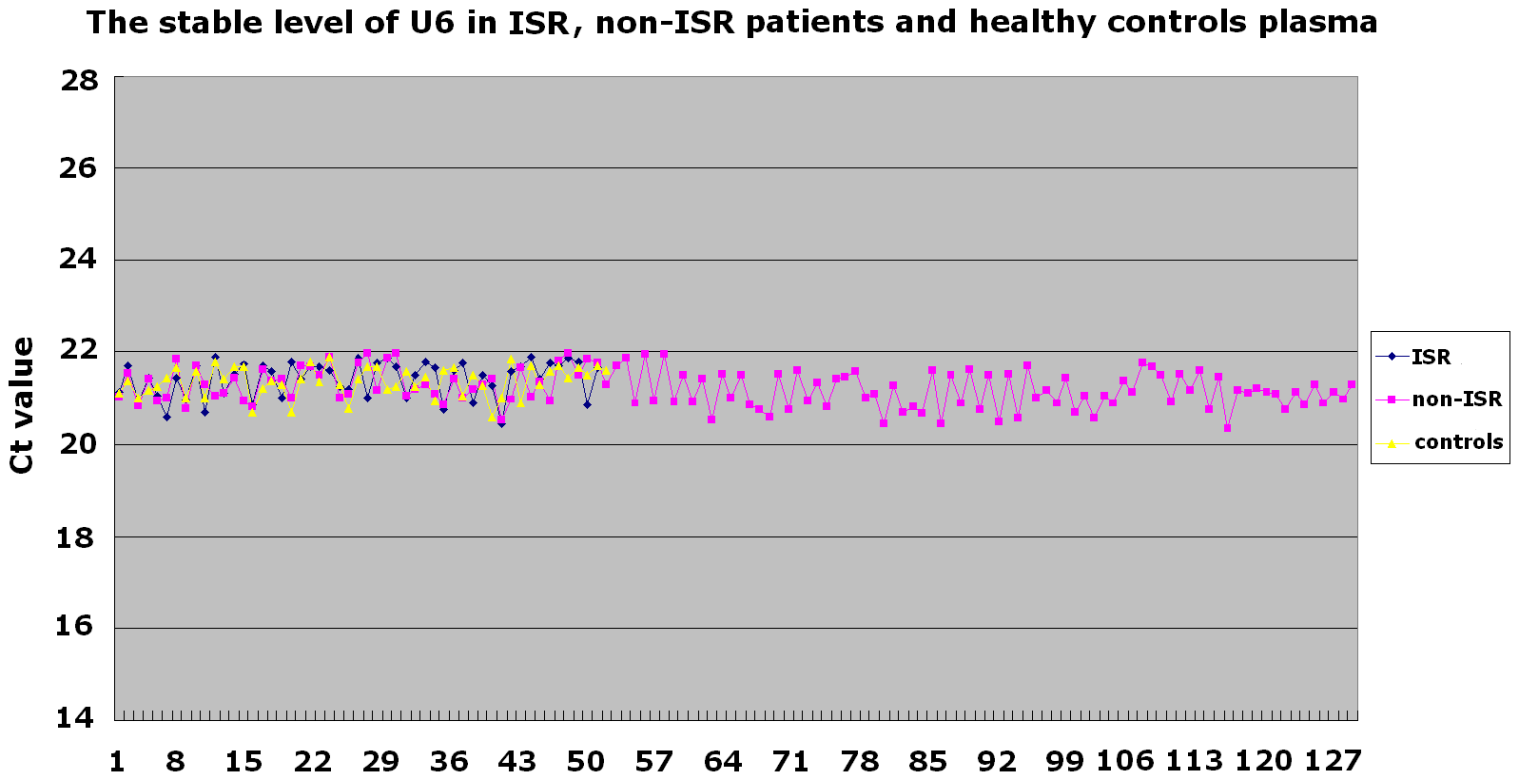
The stable level of U6 in non-ISR (n = 130), ISR (n = 51) patients and healthy controls (n = 52) plasma. The ranges of Ct values (%CV) in ISR patients are 20.4499–21.9049(1.60%), non-ISR patients are 20.3453–21.9871 (1.85%) and healthy controls are 20.5976–21.8976(1.51%) (P = 0.896).

Consort flowchart was shown in Figure 1, the patients were divided into ISR group (n = 51) and non-ISR group (n = 130) according to the results of follow-up angiography. Fifty-two healthy volunteers without any evidence of coronary artery disease or inflammatory disorders served as the control group. Patients with different ISR patterns were also compared in a sub-analysis (focal pattern group, n = 32 and diffuse pattern group, n = 19). The representative angiographic images of non-ISR, focal and diffuse ISR were shown in Figure 2. As shown in the Figure 3, we found that U6 has the low variation and no statistically significant difference among patients with ISR, non-ISR and healthy controls. The ranges of Ct values (%CV) in ISR patients are 20.4499–21.9049(1.60%), non-ISR patients are 20.3453–21.9871 (1.85%) and healthy controls are 20.5976–21.8976(1.51%) (P = 0.896).The baseline clinical characteristics, angiographic and procedural characteristics of the patients with ISR, non-ISR and healthy controls were summarized in Table 2.

**Table 2:** Clinical characteristics of patients between ISR group and non-ISR group.

Characteristics	ISR (n = 51)	Non-ISR (n = 130)	Control (n = 52)
Mean age	64.9±11.8	62.1±12.7	62.4±13.5
Male gender (%)	33(64.7)	82(63.1)	34(65.4)
Current smokers (%)	8(15.7)	20(15.4)	8(15.4)
Hypertension patients (%)	33(64.7)#	82(63.1)	0
Diabetes mellitus (%)	25(49.0)# *	58(44.6)	0
Body mass index(kg/m^2^)	24.6±4.6	24.3±4.2	24.0±4.5
Creatinine(mg/dL)	1.12±0.45	1.07±0.34	1.05±0.48
C-reactive protein(mg/L)	4.5±1.4# *	2.7±1.3	1.7±1.6
Total cholesterol (mg/dL)	178.3±35.6	176.1±36.9	175.5±35.9
LDL-C (mg/dL)	113.4±40.2# *	105.3±40.1	100.1±39.1
Drug information			
β-blocker (%)	32(62.7)	81(62.3)	NA
CCB (%)	14(27.5)	35(26.9)	NA
ACEI (%)	25(49.0)	63(48.5)	NA
ARB (%)	16(31.4)	39(30.0)	NA
Statin (%)	51(100.0)	130(100.0)	NA
Radial approach(%)	45(88.2)	116(89.2)	NA
Target coronary lesion(%)			
Left anterior descending	30(58.8)	75(57.7)	NA
Right coronary artery	21(41.2)	52(40.0)	NA
Circumflex	13(25.5)	33(25.4)	NA
Lesion characteristics			
Reference vessel diameter(mm)	2.65±0.49*	2.88±0.51	NA
Minimum lumen diameter(mm)	0.80±0.34	0.83±0.36	NA
Diameter stenosis(%)	62.9±14.6	62.2±13.9	NA
Angulated lesion(%)	7(13.7)	18(13.8)	NA
Bifurcation lesion(%)	16(31.4)	39(30.0)	NA
Number of stent(s)			
1	19(37.3)	50(38.5)	NA
2	21(41.2)	55(42.3)	NA
≥3	11(21.6)	25(19.2)	NA
Stent length(mm)	27.8±12.2	25.6±12.0	NA
Stent diameter/vessel diameter	1.061±0.04/1	1.049±0.048/1	NA

LDL-C, low density lipoprotein cholesterol; CCB, Calcium channel blockers;

ACEI, angiotensin converting enzyme inhibitor; ARB, angiotensin receptor blocker.

NA, not applicable.

Data are presented as means (±SD) or as numbers (percentages).

# comparison among patients with ISR, non-ISR and control: P<0.05 for three groups.

*comparison between patients with ISR and non-ISR: P<0.05 for ISR vs. Non-ISR.

The clinical characteristics of subjects with local ISR pattern and diffuse ISR pattern were summarized in the Table 3. There was no significant difference in all variables except for the levels of C-reactive protein. The levels of C-reactive protein was higher in patients with diffuse pattern (5.7±1.0 vs 3.8±1.2, *P*<0.05).

**Table 3:** Clinical characteristics of ISR patients between focal pattern group and diffuse pattern group.

Characteristics	Diffuse pattern (n = 19)	Focal pattern (n = 32)
Mean age	65.2±12.7	64.8±11.5
Male gender (%)	12 (63.2)	21 (65.6)
Current smokers (%)	3 (15.8)	5 (15.6)
Hypertension patients (%)	12 (63.2)	21 (65.6)
Diabetes mellitus (%)	9 (47.4)	16 (50.0)
Body mass index(kg/m^2^)	24.8±4.9	24.3±4.4
Creatinine(mg/dL)	1.15±0.47	1.10±0.41
C-reactive protein(mg/L)	5.7±1.0*	3.8±1.2
Total cholesterol (mg/dL)	178.9±36.7	177.9±35.5
LDL-C (mg/dL)	114.6±42.6	112.6±39.4
Drug information		
β-blocker (%)	12 (63.2)	20 (62.5)
CCB (%)	5 (26.3)	9 (28.1)
ACEI (%)	9 (47.4)	16 (50.0)
ARB (%)	6 (31.6)	10 (31.3)
Statin (%)	19(100.0)	32(100.0)
Radial approach(%)	17(89.5)	28(87.5)
Target coronary lesion(%)		
Left anterior descending	11(57.9)	19(59.4)
Right coronary artery	8(42.1)	13(40.6)
Circumflex	5(26.3)	8(25.0)
Lesion characteristics		
Reference vessel diameter(mm)	2.64±0.46	2.68±0.45
Minimum lumen diameter(mm)	0.79±0.34	0.81±0.32
Diameter stenosis(%)	63.0±14.4	62.3±14.2
Angulated lesion(%)	3(15.8)	4(15.4)
Bifurcation lesion(%)	6(31.6)	10(31.3)
Number of stent(s)		
1	7(36.8)	12(37.5)
2	8(42.1)	13(40.6)
≥3	4(21.1)	7(21.9)
Stent length(mm)	28.4±11.9	27.2±12.8
Stent diameter/vessel diameter	1.062±0.041/1	1.058±0.035/1

Data are presented as means (±SD) or as numbers (percentages).

*P<0.05 vs. Focal pattern group.

### MiRs profiles in ISR versus non-ISR patients

**Figure 4:**
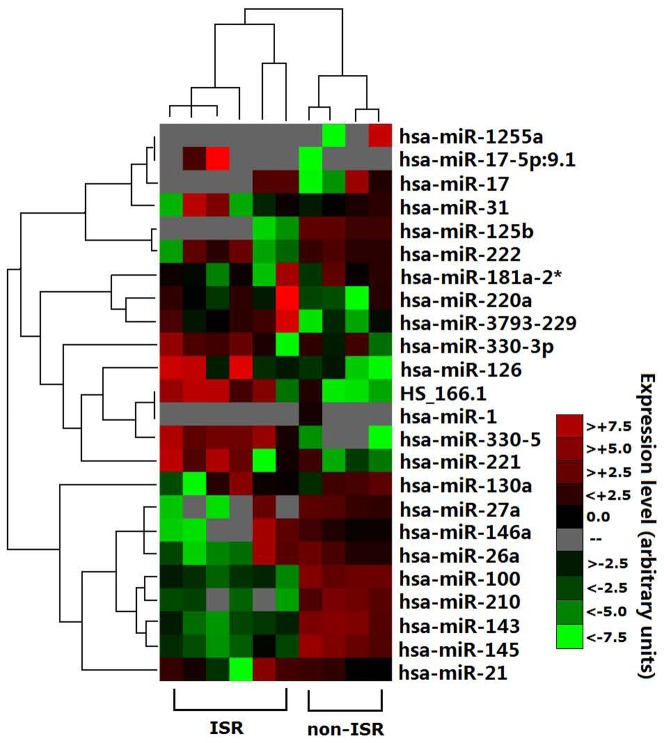
Profile of circulating miRNAs in patients with ISR patients vs non-ISR patients. RNA was isolated from plasma from ISR patients (n = 6) and non-ISR patients (n = 4). A heat map diagram is shown clustering the differentially expressed miRNAs. Data were normalized by the quantile normalization. Expression levels for miRNAs are shaded in colors such that red denotes high expression and green denotes low expression.

To identify the regulation of miRs, we used miRs microarrays from 6 ISR patients and 4 non-ISR patients. Clinical characteristics of patients were summarized in Table 4. The levels of circulating miRs in ISR patients differed from non-ISR patients, as illustrated in the heat map diagram (Figure 4). We had deposited the microRNAs raw data in the supporting information section as Table S1 and S2.

**Table 4:** Clinical characteristics of patients between ISR group and non-ISR group used for miRNAs profiling.

Characteristics	ISR (n = 6)	Non-ISR (n = 4)
Mean age	64.8±4.49	64.3±5.9
Male gender (%)	3 (50.0)	2 (50.0)
Current smokers (%)	1 (16.7)	1 (25.0)
Hypertension patients (%)	4 (66.7)	2 (50.0)
Diabetes mellitus (%)	3 (50.0)	2 (50.0)
Body mass index(kg/m^2^)	24.5±4.3	24.6±4.0
Creatinine(mg/dL)	1.10±0.44	1.11±0.43
C-reactive protein(mg/L)	4.2±1.1*	2.7±0.9
Total cholesterol (mg/dL)	179.9±34.7	170.1±33.9
LDL-C (mg/dL)	112.4±38.2*	102.3±38.1
Drug information		
β-blocker (%)	4(66.7)	2(50.0)
CCB (%)	2(33.3)	1(25.0)
ACEI (%)	2(33.3)	1(25.0)
ARB (%)	1(16.7)	1(25.0)
Statin (%)	6(100.0)	4(100.0)
Radial approach(%)	6(100.0)	4(100.0)
Target coronary lesion(%)		
Left anterior descending	4(66.7)	2(50.0)
Right coronary artery	2(33.3)	2(50.0)
Circumflex	2(33.3)	1(25.0)
Lesion characteristics		
Reference vessel diameter(mm)	2.61±0.50*	2.83±0.46
Minimum lumen diameter(mm)	0.80±0.30	0.82±0.36
Diameter stenosis(%)	62.9±13.9	62.1±13.3
Angulated lesion(%)	0	0
Bifurcation lesion(%)	0	0
Number of stent(s)		
1	1(16.7)	1(25.0)
2	3(50.0)	2(50.0)
≥3	2(33.3)	1(25.0)
Stent length(mm)	27.1±9.9	25.4±9.8
Stent diameter/vessel diameter	1.056±0.02/1	1.047±0.03/1

Data are presented as means (±SD) or as numbers (percentages).

*P<0.05 vs. Non-ISR.

### Identification of miRNAs potentially involved in ISR

**Figure 5:**
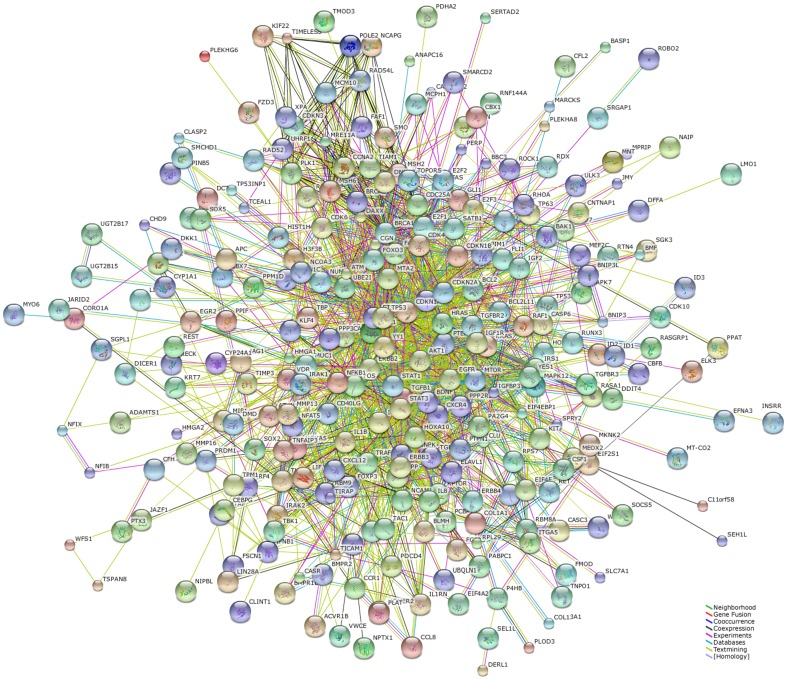
STRING analysis of the relationship between genes. The network nodes represent the proteins encoded by the genes. Different colored lines link a number of nodes and represent seven types of evidence used in predicting associations. A green line represents neighborhood evidence; a red line indicates the presence of gene fusion evidence; a blue line represents cooccurrence evidence; a black line represents coexpression evidence; a purple line represents experimental evidence; a light blue line represents database evidence and a yellow line represents text mining evidence.

**Figure 6:**
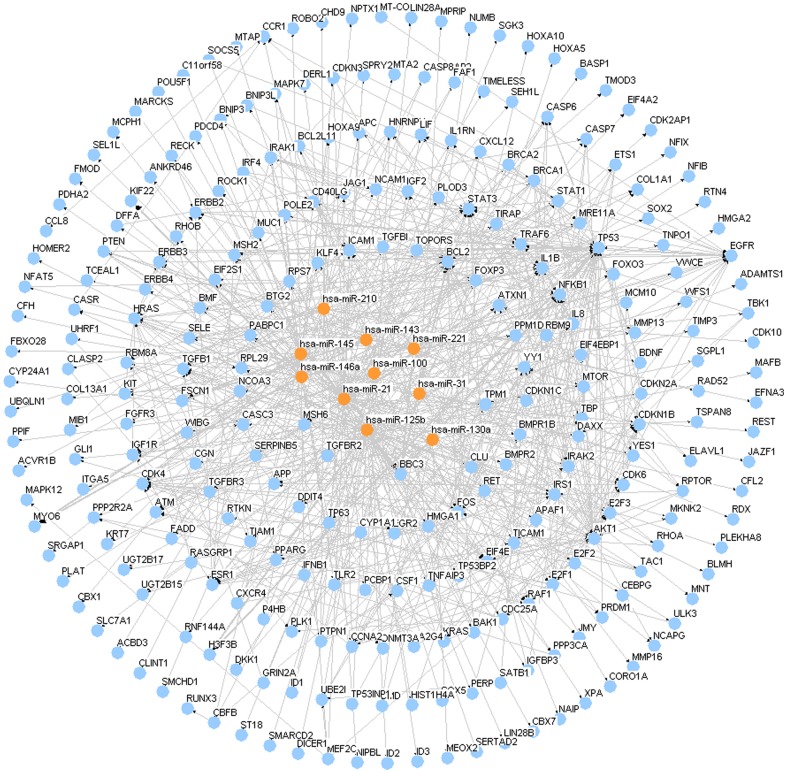
Global view of the network.

We firstly retrieved the genes knows to be associated with ISR from Entrez-Gene dabase using keywords “restenosis”, “Coronary stent” and limit to “homo sapiens”. The results indicated that CST3, FAS, AGT, CRP, ADIPOQ, ITGB3, ESR1, MMP3, PPARA, SELP, P2RY12, TNF, IL6, ACE, MMP9, NOS3, GPX1, RNASE3, AGTR1, IL1B, PPARG, TGFB1, PON1, SERPINE1, CCL2, AGER, NPPB, IGF1, SELPLG, ICAM1, BCHE, CIITA, CXCL8, THBS1, VCAM1, UCP3 and CKM are associated ISR. We transmitted our data to STRING which responds by displaying a network of nodes (proteins) connected by colored edges representing the functional relationships. From STRING database, proteome-scale interaction network of proteins was derived in Figure 5. Using the two databases miRecords and miRTarBase, which miRNAs would be most likely to be involved in the development of ISR. We found the top 10 high-traffic microRNAs which include miR-21, miR-31, miR-100, miR-125b, miR-130a, miR-143, miR-145, miR-146a, miR-210 and miR-221 known to be associated with ISR (Table S3). Using these genes and miRNAs, an interaction network was built (Figure 6). The target miRNAs predicted to regulate the expression of a high number of proteins involved in ISR, and therefore potentially involved in the process.

### Circulating miRs levels in ISR and non-ISR groups

**Figure 7:**
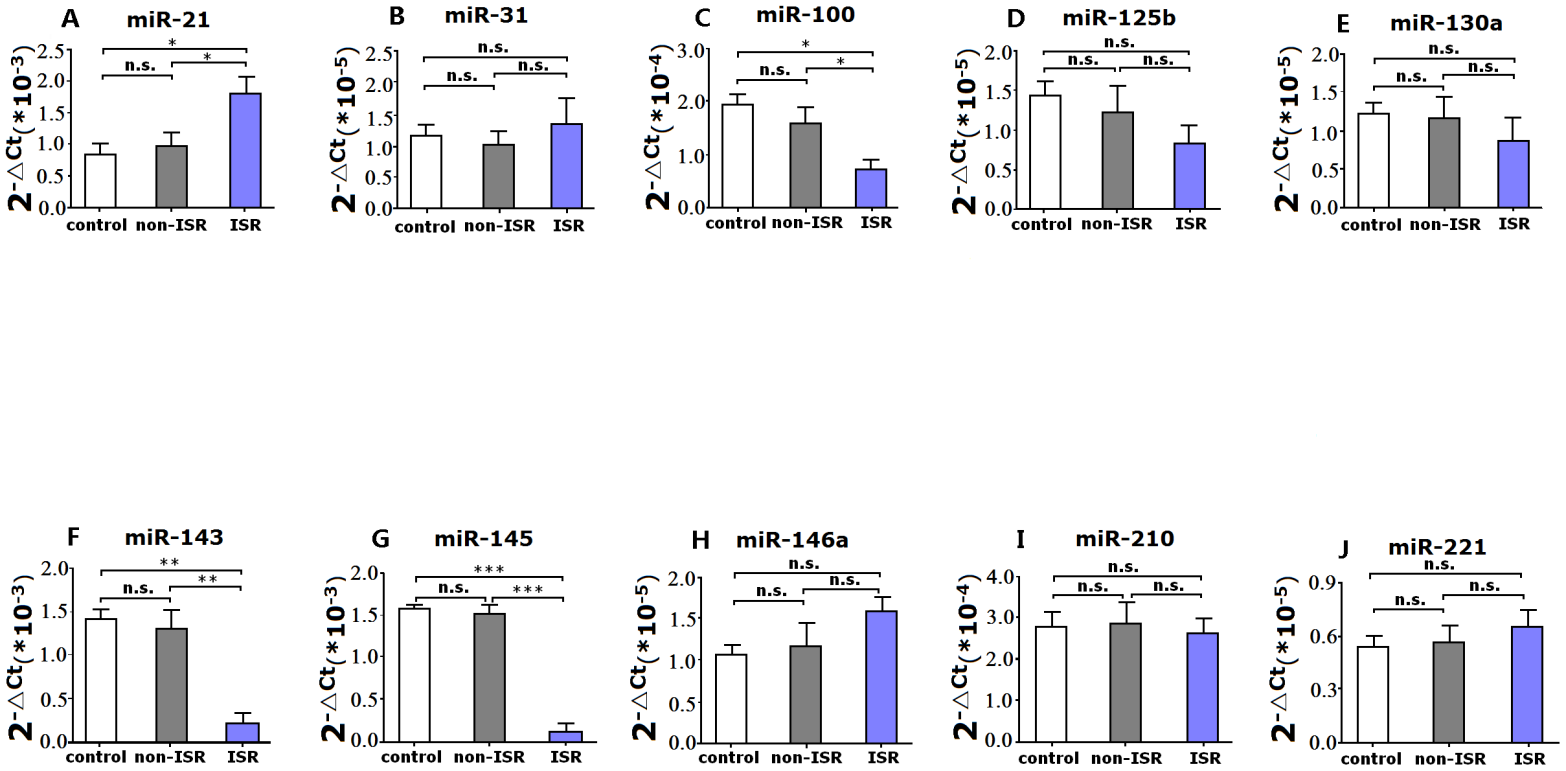
Relative expression of plasma miRNAs (qRT-PCR) in patients with ISR group (n = 51), non-ISR group (n = 130) and healthy controls (n = 52). Data are expressed as mean±SD. *P<0.05, **P<0.001, ***P<0.0001.

As shown in Figure 7, plasma levels of miR-21were higher in ISR patients compared with healthy controls (*P*<0.05) and non-ISR patients (*P*<0.05). In addition, Plasma miR-100, miR-143 and miR-145 levels were significantly lower in the ISR patients compared with healthy controls (*P*<0.05, *P*<0.001 and *P*<0.0001, respectively) and non-ISR patients (*P*<0.05, *P*<0.001 and *P*<0.0001, respectively). However, there was no significant difference in the levels of miR-31, miR-125b, miR-130a, miR-146a, miR-210 and miR-221 between the three groups.

Logistics regression model showed that miR-21, miR-100, miR-143 and miR-145 were the risk factors of the ISR (*P*<0.05 for all, shown in Table 5).

**Table 5:** Correlation between in-stent restenosis and various variables determined by logistics regression analyses.

Variable	Odds Ratio	95% Confidence Interval	P value
miR-21	1.063	1.007–1.119	0.0324
miR-100	1.009	1.001–1.017	0.0420
miR-143	2.526	1.422–4.568	0.0128
miR-145	3.342	1.092–9.921	0.0090
C-reactive protein(mg/L)	2.458	1.352–4.025	0.0362
LDL-C (mg/dL)	0.561	0.285–1.896	0.5715
Reference vessel diameter(mm)	0.404	0.135–1.209	0.1052
Diabetes mellitus (%)	2.091	0.465–9.408	0.3365

### Circulating miRs levels in ISR patients with different patterns

**Figure 8:**
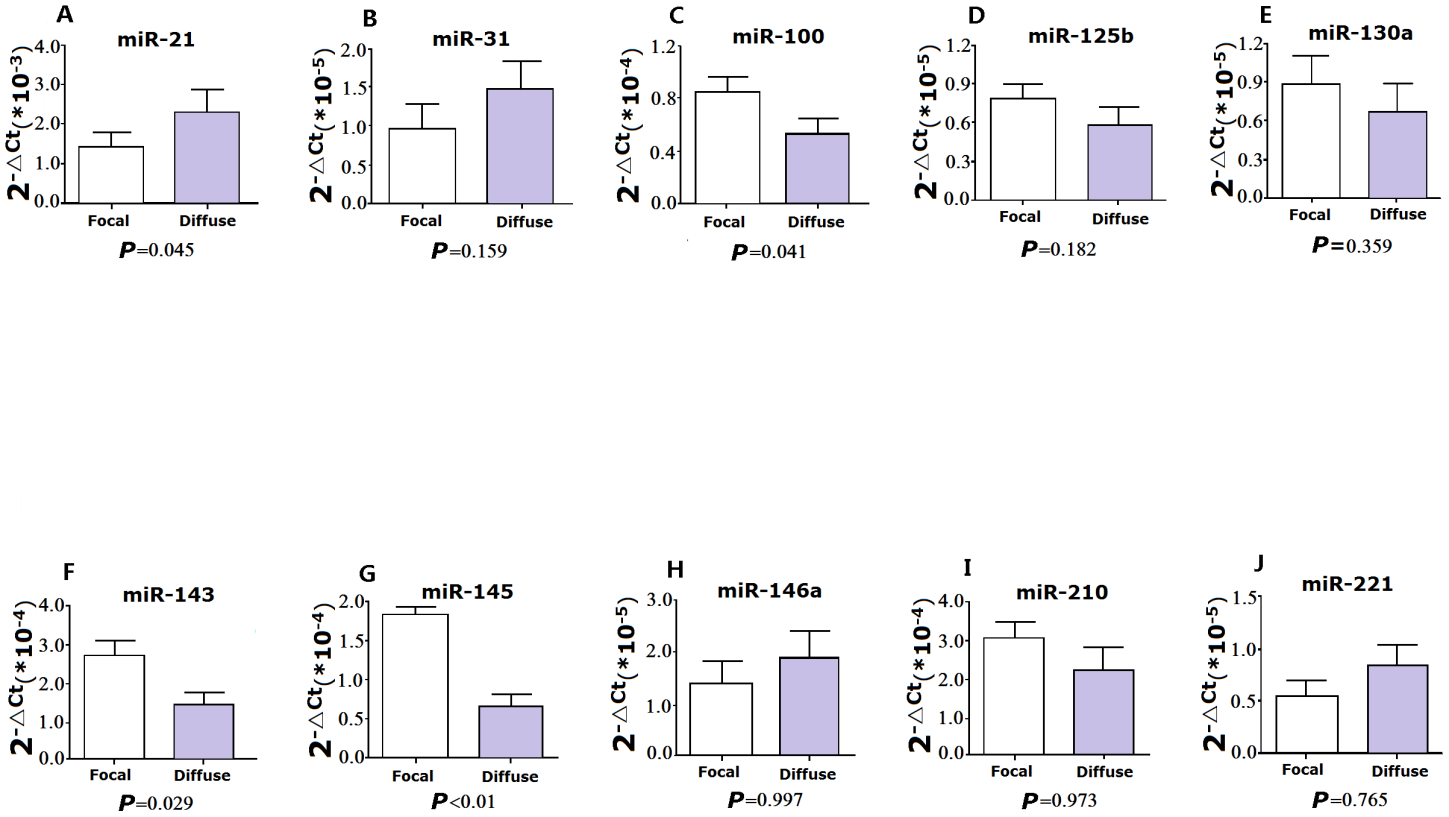
Relative expression of plasma miRNAs (qRT-PCR) in patients with focal ISR pattern group (n = 32) vs diffuse ISR pattern group (n = 19). Data are expressed as mean±SD.

Real-time PCR analysis demonstrated that plasma levels of miR-21 were higher in the diffuse ISR group than in the focal ISR group (*P* = 0.045). MiR-100, miR-143 and miR-145 were detected with lower levels in the diffuse ISR group than in the focal ISR group (*P* = 0.041, *P* = 0.029 and *P*<0.01, respectively) (Figure 8). However, there was no significant difference in the levels of miR-31, miR-125b, miR-130a, miR-146a, miR-210 and miR-221 between the two groups.

### ROC analysis of miR-21, miR-100, miR-143 and miR-145

**Figure 9:**
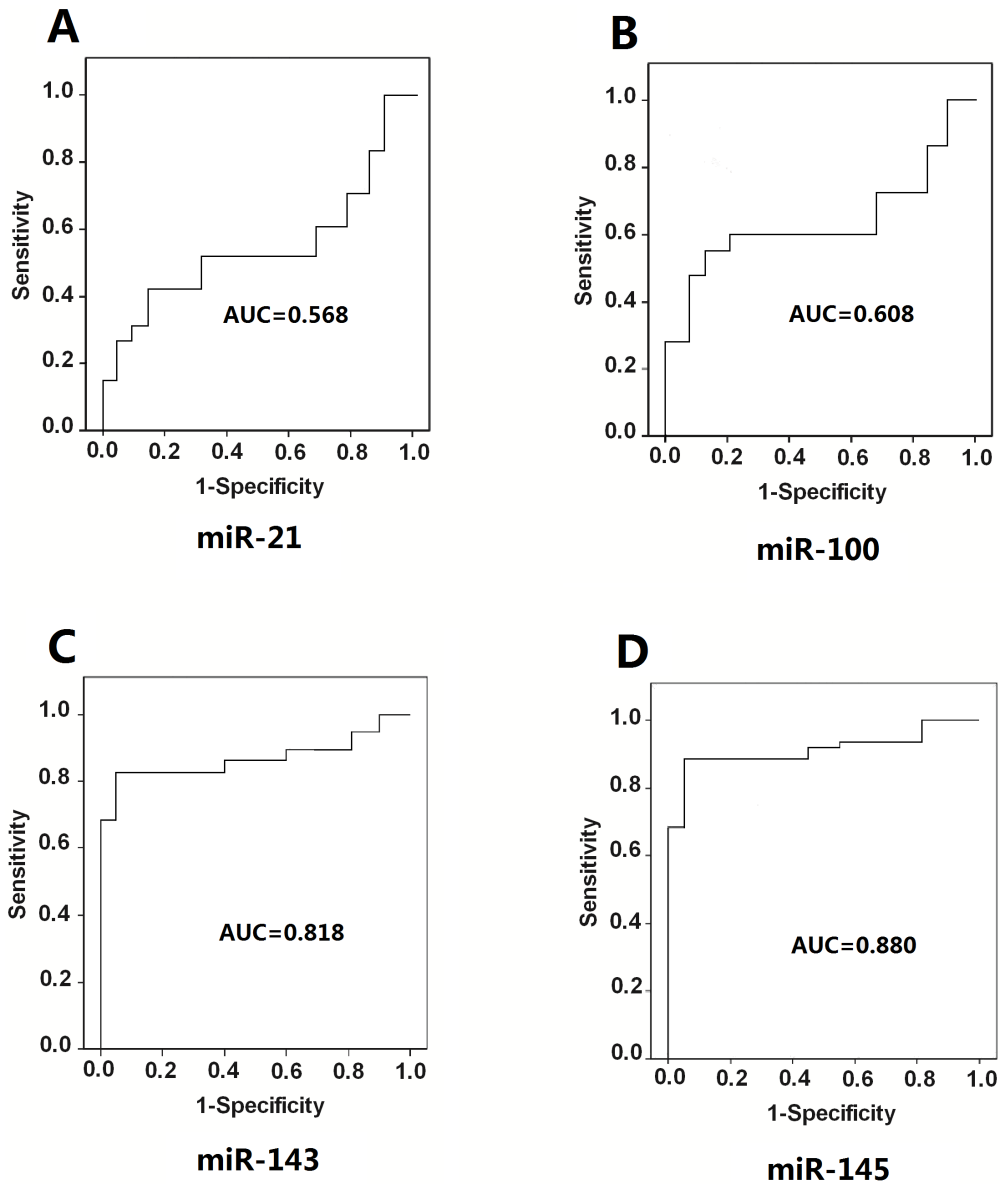
Receiver operating characteristics curve evaluation of plasma microRNAs for the diagnosis of in-stent restenosis. miR-21 (A), miR-100 (B), miR-143 (C) and miR-145 (D). AUC, the area under curve.

To investigate the relationship between the miRs and ISR, we conducted ROC analysis to evaluate the diagnostic ability of miR-21, miR-100, miR-143 and miR-145. As shown in Figure 9, the ROC curves of miR-100, miR-143and miR-145 reflected strong separation between ISR and control groups, with an AUC of 0.608 (95% confidence interval; CI = 0.372–0.757, *P*<0.05), 0.818 (95% confidence interval; CI = 0.755–0.963, *P*<0.001), and 0.880 (95% confidence interval; CI = 0.791–0.987, *P*<0.001). The ROC curves of miR-21 reflected slight separation between ISR and control groups, with AUC of 0.568 (95% confidence interval; CI = 0.372–0.757, *P*<0.05). The specificity of miR-21, miR-100, miR-143 and miR-145 were 68.6%, 68.9%, 80.1% and 83.1%, respectively. The sensitivity of miR-21, miR-100, miR-143 and miR-145 were 50.1%, 60.2%, 82.1% and 88.7%, respectively. Compared with miR-21 and miR-100, miR-143 and miR-145 displayed a higher sensitivity and specificity for diagnosis of ISR.

## Discussion

In the present study, we performed a 2-stage study to investigate the association between the circulating miRs and ISR. We observed that circulating miR-21, miR-100, miR-143 and miR-145 levels were closely associated with the occurrence of ISR after coronary DES implantation. Compared with the non-ISR patients, circulating miR-21 significantly increased in the ISR patients, while the miR-100, miR-143 and miR-145 dramatically decreased. Moreover, we found that miR-21 levels were significantly higher, while miR-100, miR-143 and miR-145 levels were much lower in patients with diffuse ISR than in those with focal ISR. ROC analysis further indicated that these four miRs might be useful biomarkers for ISR diagnosis, and miR-143 and miR-145 had the higher sensitivity and specificity. In summary, the present study provides the first clinical evidence of circulating miRs as biomarkers of ISR.

Recent studies suggested that circulating miRs are useful biomarkers for the diagnosis of cardiovascular diseases such as AMI [16], post-ischemic cardiac remodeling [17] and heart failure [18]. Furthermore, a few recent studies examined the predictive value of circulating miRs in the field of cardiovascular medicine. Eitel et al. [27] demonstrated that elevated circulating miR-133a levels in patients with AMI are associated with larger infarcts, more severe reperfusion injury, and decreased myocardial salvage. Matsumoto et al. [28] found that the serum levels of miR-155 and miR-380 at the time of discharge after AMI are higher in patients who subsequently died of cardiac cause within 1 year of discharge than in those who did not experience cardiovascular events during the 3-year follow-up period. More recently, the same group reported that the circulating p53-responsive miRs are predictive indicators of heart failure after AMI [29]. These observations indicate that circulating miRs have the potential to predict prognosis in patients with cardiovascular disease. So far, it remains unclear about the predictive role of serum miRs for post-stent intimal hyperplasia. In the present study, we for the first time identified the miRs(miR-21, miR-100, miR-143 and miR-145) that have the potential to predict the severity of post-stent intimal hyperplasia. Among them, miR-21 levels were significantly higher, while miR-100, miR-143 and miR-145 levels were lower in patients with diffuse ISR than in those with focal ISR.

The primary mechanism underlying ISR is an exaggerated neointimal proliferative response. It is well established that VSMCs proliferation and migration are critical cellular events responsible for the development of neointimal hyperplasia and the VSMCs phenotypic modulation(transformation from contractile to synthetic phenotype) plays an important role in this process [2], [30]. Interestingly, the serum miRs (miR-21, miR-100, miR-143 and miR-145) that are predictive for post-stent intimal hyperplasia have been demonstrated to participate in the pathogenesis of vascular diseases by recent studies [6], [31]–[33]. miR-21, an miRNA that is upregulated in vascular walls with neointima, was reported to exert pro-proliferative and anti-apoptotic effects on VSMCs, and play an important role in the control of VSMCs phenotype [6], [31]. Knockdown of miR-21 suppressed neointimal lesion formation after angioplasty in rat carotid arteries [6]. MiR-145 and miR-143 are highly expressed in VSMCs and their expression are significantly downregulated in the vascular walls with neointimal lesion formation [8], [32]. Moreover, miR-145 and miR-143 are critical modulators of VSMC phenotype, which promote differentiation and represses proliferation of VSMCs [8], [32]. Restoration of miR-145 and miR-143 in injured arteries inhibited neointimal growth. A recent study found that miR-100 is also highly expressed in VSMCs and inhibition of miR-100 has a significant stimulatory effect on VSMCs proliferation and migration [10].

In addition, inflammation may also play a role in ISR after coronary DES implantation. The inflammatory mechanisms in the pathogenesis of neointimal proliferation include sustained leukocyte infiltration in the arterial wall and VSMCs proliferation and migration [34]–[36]. In our study, there were more diabetes patients and higher C-reactive protein in ISR group may be related to inflammatory mechanisms. Recent study showed that miR-146 and miR-21 have been of particular interest for research associated with inflammatory and immune responses [37]. By means of promoter analysis, miR-146a was found to be a nuclear factor κB (NF-κB)-dependent gene [38] and it was demonstrated that RelB regulates inflammatory mediator production in part through a mechanism involving miR-146a [39]. MiR-21, is known to be a common inflammation-inducible miR are induced by pro-inflammatory stimuli such as interleukin-1β (IL-1β), tumor necrosis factor α (TNFα), Toll-like receptors (TLRs) and phosphatase and tensin homolog deleted on chromosome ten (PTEN) [40]–[41]. Inflammatory response such as TLR4 activation induces the expression of miR-125b expression [38] and miR-125b could contribute to the pro-inflammatory state associated with endothelial senescence [42]. The precise mechanism underlying the expression alterations of these circulating miRs remains unknown. Previous studies showed that miRNAs are able to be released into circulating blood from a variety of cells such as cardiomyocytes, endothelial cells, T cells and cancer cells [43]. One mode of miRNA secretion is by passive leakage from necrotic or apoptotic cells, and the other mode is by active secretion from living cells within microvesicles or in RNA-lipid/protein complexes [43]. As consistent alterations of miR-21, miR-100, miR-143 and miR-145 were observed in both the serum of patients with ISR and balloon-injured rat carotid arteries, with miR-21 upregulated, while miR-100, miR-143 and miR-145 downregulated. We speculated that these circulating miRs expression changes may be caused by the active secretion from VSMCs in neointimal lesions. It would be worthwhile to examine the expression of these miRs in human VSMCs in future studies to further explore the origin of plasma miRs in ISR patients.

In addition to the above-mentioned four miRs, many miRs such as miR-31, miR-125b, miR-130a, miR-146a, miR-210 and miR-221, have been reported to play important roles in VSMCs biology [7], [44]. However, we did not observe any significant association between these miRs and the occurrence of ISR in our study. There are several possible reasons for the disparity. Firstly, these miRs may not play roles in ISR of human subjects. Secondly, miRs release into the systemic circulation may be a relatively selective process, possibly miRNA-specific. Thirdly, the stability of circulating microRNAs may be different. It has been shown that serum miRs associated with vesicles appear to be more stable than those not associated with vesicles [45].

Recently, Kaudewitz et al. [46] demonstrated that intravenous administration of heparin affects the quantification of circulating microRNAs in patients with coronary artery disease. In our present study, blood samples for microRNAs detection were collected from the patients before the angiography procedure and heparin administration, thus the influences of heparin on quantification of circulating microRNAs can be excluded.

Besides of their potential role as biomarkers, circulating miRs may also play a role in influencing gene expression at intracellular locations. In fact, several studies have suggested that secreted microvesicles containing miRs could transfer the miRs to recipient cells and regulate gene expression [47]. Therefore, future studies are needed to clarify whether these miRs in the bloodstream are contained in exosomes or microparticles, and whether they can be delivered to VSMCs and modulate the proliferation and migration of VSMCs during ISR.

### Limitations

The present study provides the first clinical evidence of circulating miRs as biomarkers of ISR. However, the small number of enrolled patients from a single center is a major limitation that must be considered. In addition, the high rate of ISR was probably related to sampling only symptomatic patients who returned for CAG. Therefore, further larger-scale studies are needed to validate the clinical utility of miRs as practical biomarkers for ISR. Furthermore, a longitudinal study should be performed to better assess the predictive value of miRs as biomarkers for ISR, since our work is a cross-sectional study.

## Conclusions

In summary, the present study provides first insights into the levels of circulating miRs in patients with ISR. Our study shows that circulating miR-143 and miR-145 levels are associated with the occurrence of ISR and can serve as novel noninvasive biomarkers for ISR.

### Registration Information

Registration title: The relationship between microRNAs and in-stent restenosis of coronary artery. Registration number: ChiCTR-OCH-14004263.

## Supporting Information

Table S1 **MicroRNAs microarray raw data.**
(DOC)

Table S2 **Microarray data were normalized by the quantile normalization.**
(DOC)

Table S3 **List of 10 miRNAs and their target gene.**
(DOC)
