# Supplementary material for: Plasma MicroRNAs as Potential Noninvasive Biomarkers for In-Stent Restenosis
Source: PLoS One. 2014 Nov 26;9(11):e112043. doi: 10.1371/journal.pone.0112043 (PMC4245195; doi:10.1371/journal.pone.0112043)
Supplement: Table S1 — MicroRNAs microarray raw data. (DOC) [file pone.0112043.s001.doc]

Table S1. MicroRNAs microarray raw data

| SYMBOL | non-ISR-1.AVG_Signal | non-ISR-2.AVG_Signal | non-ISR-3.AVG_Signal | non-ISR-4.AVG_Signal | ISR-1.AVG_Signal | ISR-2.AVG_Signal | ISR-3.AVG_Signal | ISR-4.AVG_Signal | ISR-5.AVG_Signal | ISR-6.AVG_Signal | ILMN_GENE | ARRAY_ADDRESS_ID | |
| --- | --- | --- | --- | --- | --- | --- | --- | --- | --- | --- | --- | --- | --- |
| ILMN_3167304 | 65 | 125 | 45 | 57 | 71 | 69 | 68 | 97 | 59 | 42 | HS_1 | 2256 |  |
| ILMN_3168038 | 401 | 186 | 98 | 772 | 73 | 69 | 68 | 88 | 57 | 53 | HS_10 | 2257 |  |
| ILMN_3167890 | 18954 | 19455 | 20355 | 12592 | 67 | 65 | 64 | 81 | 3030 | 1659 | HS_100 | 2259 |  |
| ILMN_3167526 | 974 | 104 | 66 | 108 | 69 | 68 | 65 | 88 | 59 | 48 | HS_101 | 2261 |  |
| ILMN_3167209 | 351 | 107 | 544 | 521 | 69 | 68 | 65 | 97 | 59 | 45 | HS_104 | 2265 |  |
| ILMN_3168219 | 805 | 484 | 772 | 315 | 74 | 72 | 67 | 90 | 58 | 62 | HS_105 | 2271 |  |
| ILMN_3167464 | 225 | 194 | 370 | 168 | 88 | 87 | 80 | 104 | 70 | 62 | HS_106 | 2277 |  |
| ILMN_3167213 | 63 | 65 | 56 | 68 | 68 | 68 | 64 | 85 | 57 | 46 | HS_107 | 2281 |  |
| ILMN_3167057 | 19140 | 7915 | 8169 | 6855 | 87 | 71 | 82 | 97 | 61 | 49 | HS_108.1 | 2044 |  |
| ILMN_3168313 | 236 | 60 | 60 | 92 | 78 | 75 | 75 | 98 | 66 | 47 | HS_109 | 2291 |  |
| ILMN_3166972 | 149 | 152 | 138 | 439 | 85 | 84 | 83 | 110 | 70 | 56 | HS_11.1 | 2048 |  |
| ILMN_3168149 | 674 | 838 | 961 | 907 | 81 | 138 | 74 | 95 | 60 | 71 | HS_110 | 2296 |  |
| ILMN_3168527 | 701 | 103 | 84 | 70 | 71 | 69 | 66 | 91 | 58 | 46 | HS_111 | 2297 |  |
| ILMN_3167562 | 125 | 127 | 59 | 66 | 66 | 65 | 63 | 251 | 55 | 44 | HS_112 | 2298 |  |
| ILMN_3168099 | 722 | 845 | 791 | 734 | 113 | 94 | 82 | 102 | 68 | 96 | HS_113 | 2304 |  |
| ILMN_3168017 | 1232 | 1962 | 1864 | 2029 | 482 | 87 | 77 | 102 | 64 | 72 | HS_114 | 2305 |  |
| ILMN_3167545 | 359 | 64 | 567 | 73 | 77 | 75 | 73 | 568 | 61 | 48 | HS_115 | 2308 |  |
| ILMN_3167951 | 907 | 727 | 442 | 1066 | 76 | 73 | 69 | 100 | 61 | 51 | HS_116 | 2311 |  |
| ILMN_3167172 | 294 | 100 | 90 | 108 | 81 | 75 | 74 | 96 | 64 | 50 | HS_117 | 2312 |  |
| ILMN_3167994 | 626 | 348 | 315 | 322 | 81 | 80 | 91 | 185 | 73 | 62 | HS_119 | 2316 |  |
| ILMN_3168104 | 4383 | 109 | 907 | 101 | 69 | 68 | 65 | 87 | 55 | 45 | HS_12 | 2318 |  |
| ILMN_3166997 | 166 | 131 | 137 | 117 | 88 | 85 | 85 | 104 | 73 | 55 | HS_120 | 2320 |  |
| ILMN_3166980 | 137 | 153 | 137 | 124 | 90 | 77 | 77 | 101 | 64 | 54 | HS_121 | 2321 |  |
| ILMN_3168362 | 526 | 459 | 366 | 416 | 95 | 99 | 92 | 132 | 81 | 87 | HS_122.1 | 2049 |  |
| ILMN_3168532 | 242 | 206 | 195 | 176 | 136 | 128 | 130 | 163 | 108 | 76 | HS_123 | 2322 |  |
| ILMN_3167958 | 66 | 50 | 47 | 54 | 74 | 70 | 72 | 96 | 60 | 46 | HS_124 | 2324 |  |
| ILMN_3168523 | 781 | 853 | 973 | 815 | 76 | 75 | 78 | 103 | 5063 | 51 | HS_126 | 2327 |  |
| ILMN_3167169 | 291 | 75 | 55 | 71 | 78 | 72 | 74 | 100 | 67 | 50 | HS_127.1 | 2050 |  |
| ILMN_3167639 | 358 | 400 | 386 | 729 | 88 | 79 | 76 | 96 | 64 | 66 | HS_128 | 2328 |  |
| ILMN_3167021 | 315 | 99 | 112 | 108 | 87 | 78 | 81 | 114 | 71 | 51 | HS_129 | 2329 |  |
| ILMN_3167142 | 103 | 166 | 133 | 136 | 72 | 72 | 68 | 86 | 58 | 55 | HS_13 | 2330 |  |
| ILMN_3168254 | 225 | 256 | 196 | 189 | 81 | 82 | 77 | 101 | 65 | 60 | HS_130 | 2331 |  |
| ILMN_3168028 | 263 | 447 | 254 | 312 | 65 | 76 | 60 | 84 | 55 | 50 | HS_131 | 2342 |  |
| ILMN_3168531 | 178 | 245 | 230 | 195 | 67 | 67 | 64 | 89 | 55 | 43 | HS_132.1 | 2051 |  |
| ILMN_3168207 | 291 | 432 | 274 | 1126 | 84 | 87 | 88 | 123 | 71 | 80 | HS_133.1 | 2054 |  |
| ILMN_3168054 | 131 | 179 | 207 | 201 | 69 | 79 | 68 | 87 | 59 | 69 | HS_134 | 2347 |  |
| ILMN_3167512 | 1560 | 128 | 445 | 83 | 69 | 68 | 70 | 90 | 56 | 51 | HS_135 | 2350 |  |
| ILMN_3167120 | 168 | 82 | 71 | 75 | 110 | 78 | 75 | 112 | 60 | 57 | HS_136 | 2352 |  |
| ILMN_3168445 | 91 | 81 | 135 | 87 | 77 | 80 | 73 | 89 | 63 | 50 | HS_137 | 2354 |  |
| ILMN_3167622 | 539 | 420 | 386 | 330 | 121 | 99 | 105 | 165 | 81 | 91 | HS_138 | 2356 |  |
| ILMN_3167047 | 2086 | 2488 | 1391 | 1348 | 146 | 147 | 167 | 543 | 122 | 161 | HS_139 | 2358 |  |
| ILMN_3166987 | 165 | 137 | 360 | 122 | 72 | 78 | 71 | 95 | 59 | 56 | HS_14.1 | 2057 |  |
| ILMN_3167800 | 456 | 65 | 66 | 95 | 73 | 71 | 75 | 2157 | 59 | 43 | HS_140 | 2360 |  |
| ILMN_3167414 | 557 | 481 | 394 | 355 | 76 | 75 | 73 | 258 | 61 | 52 | HS_141 | 2361 |  |
| ILMN_3167153 | 553 | 678 | 1960 | 533 | 2940 | 78 | 77 | 104 | 68 | 60 | HS_142.1 | 2059 |  |
| ILMN_3167466 | 186 | 79 | 549 | 81 | 94 | 68 | 64 | 87 | 54 | 44 | HS_143 | 2363 |  |
| ILMN_3167612 | 216 | 226 | 251 | 643 | 74 | 74 | 68 | 91 | 59 | 60 | HS_144 | 2369 |  |
| ILMN_3168433 | 737 | 675 | 564 | 674 | 109 | 111 | 111 | 3635 | 85 | 81 | HS_145.1 | 2062 |  |
| ILMN_3167332 | 169 | 136 | 237 | 119 | 77 | 73 | 72 | 99 | 64 | 52 | HS_146.1 | 2065 |  |
| ILMN_3168135 | 542 | 199 | 597 | 134 | 73 | 73 | 68 | 95 | 61 | 57 | HS_147 | 2370 |  |
| ILMN_3168101 | 1630 | 690 | 2314 | 925 | 94 | 91 | 86 | 109 | 4171 | 72 | HS_149 | 2375 |  |
| ILMN_3167356 | 210 | 104 | 78 | 72 | 75 | 72 | 71 | 93 | 60 | 47 | HS_15.1 | 2081 |  |
| ILMN_3167952 | 2997 | 1697 | 1445 | 1357 | 143 | 205 | 191 | 1468 | 189 | 183 | HS_150 | 2383 |  |
| ILMN_3167470 | 106 | 63 | 58 | 68 | 73 | 70 | 68 | 90 | 59 | 44 | HS_151.1 | 2087 |  |
| ILMN_3168233 | 1041 | 1226 | 1215 | 1084 | 99 | 99 | 92 | 124 | 71 | 104 | HS_152 | 2390 |  |
| ILMN_3167049 | 443 | 480 | 377 | 378 | 77 | 78 | 76 | 260 | 63 | 69 | HS_153 | 2394 |  |
| ILMN_3167868 | 134 | 121 | 81 | 98 | 69 | 69 | 65 | 86 | 56 | 44 | HS_154 | 2398 |  |
| ILMN_3168500 | 152 | 99 | 49 | 55 | 70 | 68 | 66 | 91 | 58 | 43 | HS_155 | 2401 |  |
| ILMN_3168305 | 99 | 67 | 72 | 135 | 72 | 71 | 67 | 87 | 58 | 44 | HS_156 | 2402 |  |
| ILMN_3167993 | 256 | 225 | 281 | 105 | 62 | 62 | 74 | 80 | 54 | 40 | HS_157 | 2403 |  |
| ILMN_3167678 | 291 | 562 | 116 | 82 | 74 | 71 | 70 | 92 | 59 | 52 | HS_159 | 2406 |  |
| ILMN_3168232 | 110 | 136 | 109 | 123 | 70 | 67 | 65 | 92 | 54 | 44 | HS_16 | 2409 |  |
| ILMN_3167004 | 398 | 491 | 554 | 455 | 81 | 76 | 73 | 95 | 66 | 57 | HS_160 | 2413 |  |
| ILMN_3167329 | 102 | 659 | 60 | 529 | 76 | 74 | 74 | 102 | 65 | 48 | HS_161 | 2415 |  |
| ILMN_3167742 | 81 | 91 | 81 | 82 | 76 | 75 | 75 | 96 | 62 | 53 | HS_162 | 2416 |  |
| ILMN_3168069 | 552 | 244 | 83 | 85 | 76 | 71 | 71 | 90 | 59 | 47 | HS_163 | 2418 |  |
| ILMN_3168156 | 130 | 158 | 85 | 98 | 74 | 73 | 74 | 92 | 61 | 46 | HS_164 | 2420 |  |
| ILMN_3168315 | 979 | 613 | 395 | 418 | 91 | 93 | 104 | 163 | 72 | 57 | HS_166.1 | 2094 |  |
| ILMN_3168474 | 393 | 423 | 460 | 1929 | 99 | 94 | 109 | 125 | 86 | 73 | HS_167.1 | 2095 |  |
| ILMN_3167854 | 441 | 913 | 630 | 645 | 91 | 84 | 82 | 115 | 67 | 63 | HS_168 | 2424 |  |
| ILMN_3168235 | 485 | 2450 | 353 | 76 | 69 | 70 | 69 | 95 | 59 | 47 | HS_169 | 2425 |  |
| ILMN_3168132 | 3219 | 378 | 1942 | 480 | 69 | 69 | 65 | 87 | 56 | 45 | HS_17 | 2430 |  |
| ILMN_3167684 | 2716 | 3894 | 3520 | 93 | 74 | 77 | 73 | 95 | 61 | 52 | HS_170 | 2431 |  |
| ILMN_3167533 | 379 | 140 | 77 | 75 | 69 | 66 | 66 | 87 | 56 | 46 | HS_174.1 | 2097 |  |
| ILMN_3168435 | 432 | 421 | 226 | 200 | 87 | 87 | 85 | 120 | 72 | 59 | HS_175 | 2439 |  |
| ILMN_3167297 | 6974 | 1224 | 1853 | 586 | 3684 | 6102 | 7901 | 16363 | 7423 | 2804 | HS_176 | 2440 |  |
| ILMN_3167087 | 249 | 303 | 273 | 261 | 79 | 78 | 77 | 101 | 125 | 53 | HS_177 | 2442 |  |
| ILMN_3166990 | 563 | 423 | 211 | 256 | 78 | 79 | 1320 | 94 | 60 | 53 | HS_179 | 2458 |  |
| ILMN_3168159 | 425 | 171 | 95 | 99 | 72 | 66 | 69 | 86 | 59 | 50 | HS_18 | 2459 |  |
| ILMN_3168063 | 2238 | 520 | 2595 | 255 | 77 | 79 | 71 | 97 | 168 | 49 | HS_182.1 | 2110 |  |
| ILMN_3167343 | 52 | 41 | 36 | 58 | 66 | 64 | 63 | 82 | 55 | 40 | HS_183.1 | 2114 |  |
| ILMN_3168093 | 2094 | 1865 | 1870 | 1448 | 123 | 121 | 5440 | 195 | 88 | 160 | HS_184 | 2485 |  |
| ILMN_3168179 | 208 | 78 | 77 | 88 | 72 | 69 | 66 | 88 | 59 | 45 | HS_185.1 | 2117 |  |
| ILMN_3168398 | 780 | 1091 | 1057 | 895 | 100 | 669 | 94 | 129 | 78 | 130 | HS_186 | 2501 |  |
| ILMN_3167542 | 418 | 101 | 497 | 99 | 68 | 65 | 65 | 86 | 57 | 45 | HS_187 | 2534 |  |
| ILMN_3167559 | 3739 | 1044 | 2538 | 536 | 83 | 80 | 4025 | 144 | 71 | 60 | HS_188 | 2536 |  |
| ILMN_3168392 | 146 | 105 | 244 | 72 | 70 | 72 | 68 | 90 | 58 | 45 | HS_189.1 | 2122 |  |
| ILMN_3168383 | 1711 | 1714 | 2244 | 1081 | 196 | 174 | 176 | 266 | 136 | 183 | HS_19 | 2555 |  |
| ILMN_3167633 | 124 | 78 | 82 | 85 | 68 | 73 | 71 | 95 | 57 | 49 | HS_190 | 2584 |  |
| ILMN_3167585 | 9910 | 6611 | 9380 | 2568 | 67 | 67 | 63 | 89 | 53 | 41 | HS_192.1 | 2127 |  |
| ILMN_3167858 | 547 | 218 | 476 | 176 | 91 | 92 | 92 | 119 | 72 | 74 | HS_193 | 2619 |  |
| ILMN_3167733 | 641 | 54 | 50 | 147 | 69 | 67 | 104 | 84 | 56 | 43 | HS_194 | 2630 |  |
| ILMN_3167041 | 136 | 131 | 127 | 123 | 86 | 86 | 83 | 107 | 74 | 1703 | HS_195 | 2632 |  |
| ILMN_3166967 | 810 | 162 | 262 | 88 | 70 | 67 | 66 | 88 | 56 | 43 | HS_196.1 | 2129 |  |
| ILMN_3167792 | 168 | 147 | 118 | 123 | 79 | 77 | 74 | 96 | 63 | 62 | HS_197 | 2651 |  |
| ILMN_3167139 | 94 | 65 | 60 | 64 | 71 | 69 | 69 | 92 | 64 | 45 | HS_198 | 2658 |  |
| ILMN_3167749 | 604 | 375 | 361 | 293 | 77 | 80 | 70 | 92 | 60 | 82 | HS_199 | 2661 |  |
| ILMN_3167592 | 615 | 648 | 893 | 257 | 80 | 78 | 75 | 100 | 63 | 59 | HS_2 | 2680 |  |
| ILMN_3167795 | 1348 | 1316 | 1163 | 1043 | 109 | 141 | 149 | 463 | 137 | 131 | HS_20 | 2692 |  |
| ILMN_3167313 | 679 | 346 | 389 | 230 | 105 | 87 | 86 | 109 | 73 | 67 | HS_200 | 2693 |  |
| ILMN_3167401 | 480 | 449 | 748 | 354 | 102 | 104 | 98 | 125 | 81 | 67 | HS_201 | 2698 |  |
| ILMN_3167955 | 21636 | 2211 | 3844 | 6105 | 2976 | 5471 | 8029 | 15522 | 7225 | 4665 | HS_202.1 | 2136 |  |
| ILMN_3168524 | 663 | 677 | 752 | 331 | 90 | 85 | 83 | 109 | 69 | 61 | HS_203 | 2705 |  |
| ILMN_3167640 | 17326 | 15586 | 15529 | 14291 | 9763 | 6424 | 6203 | 12073 | 6827 | 7436 | HS_204.1 | 2137 |  |
| ILMN_3168332 | 141 | 104 | 95 | 107 | 82 | 74 | 78 | 107 | 65 | 60 | HS_205.1 | 2139 |  |
| ILMN_3168217 | 258 | 380 | 418 | 69 | 66 | 66 | 64 | 85 | 55 | 43 | HS_206 | 2706 |  |
| ILMN_3167907 | 187 | 175 | 580 | 282 | 78 | 85 | 74 | 97 | 66 | 60 | HS_208 | 2707 |  |
| ILMN_3167069 | 1983 | 341 | 756 | 148 | 82 | 83 | 78 | 163 | 69 | 55 | HS_209.1 | 2144 |  |
| ILMN_3167462 | 138 | 206 | 297 | 160 | 73 | 72 | 71 | 131 | 62 | 53 | HS_21 | 2708 |  |
| ILMN_3166952 | 167 | 125 | 143 | 172 | 74 | 72 | 69 | 92 | 59 | 46 | HS_211 | 2717 |  |
| ILMN_3166995 | 666 | 275 | 162 | 122 | 73 | 71 | 70 | 143 | 59 | 47 | HS_215 | 2723 |  |
| ILMN_3167862 | 210 | 97 | 88 | 92 | 97 | 82 | 83 | 114 | 78 | 57 | HS_216 | 2728 |  |
| ILMN_3168092 | 421 | 230 | 60 | 63 | 80 | 81 | 1589 | 1344 | 1314 | 681 | HS_217 | 2729 |  |
| ILMN_3167280 | 186 | 81 | 79 | 90 | 76 | 74 | 79 | 99 | 62 | 49 | HS_218 | 2732 |  |
| ILMN_3168421 | 471 | 454 | 462 | 362 | 91 | 91 | 95 | 141 | 75 | 75 | HS_219 | 2733 |  |
| ILMN_3167488 | 6965 | 2691 | 4985 | 4860 | 91 | 95 | 91 | 130 | 68 | 88 | HS_22.1 | 2152 |  |
| ILMN_3167068 | 209 | 100 | 112 | 112 | 76 | 75 | 70 | 93 | 59 | 4613 | HS_220 | 2738 |  |
| ILMN_3168335 | 540 | 79 | 48 | 49 | 66 | 68 | 67 | 84 | 57 | 42 | HS_221 | 2739 |  |
| ILMN_3168504 | 611 | 802 | 1895 | 413 | 70 | 2734 | 65 | 5101 | 8573 | 5040 | HS_228.1 | 2153 |  |
| ILMN_3166985 | 202 | 205 | 283 | 201 | 77 | 78 | 71 | 97 | 2430 | 58 | HS_23 | 2741 |  |
| ILMN_3167534 | 4602 | 161 | 72 | 88 | 71 | 90 | 69 | 442 | 60 | 47 | HS_231 | 2748 |  |
| ILMN_3168162 | 188 | 92 | 1247 | 133 | 75 | 72 | 73 | 100 | 62 | 49 | HS_232 | 2749 |  |
| ILMN_3167766 | 14976 | 2963 | 3407 | 4637 | 7760 | 4706 | 5398 | 12986 | 7473 | 4123 | HS_239 | 2753 |  |
| ILMN_3168122 | 180 | 126 | 94 | 286 | 78 | 78 | 74 | 98 | 64 | 56 | HS_24 | 2755 |  |
| ILMN_3168349 | 312 | 75 | 95 | 88 | 76 | 71 | 68 | 734 | 62 | 46 | HS_240 | 2760 |  |
| ILMN_3167586 | 494 | 352 | 797 | 188 | 71 | 75 | 73 | 100 | 64 | 49 | HS_241.1 | 2155 |  |
| ILMN_3167277 | 158 | 255 | 1304 | 490 | 5706 | 3647 | 1652 | 88 | 59 | 1918 | HS_242 | 2768 |  |
| ILMN_3168182 | 11078 | 3888 | 6801 | 2765 | 76 | 76 | 69 | 91 | 58 | 55 | HS_243.1 | 2157 |  |
| ILMN_3167082 | 2101 | 2517 | 2327 | 3172 | 7463 | 298 | 7568 | 5124 | 386 | 1239 | HS_244 | 2776 |  |
| ILMN_3167744 | 796 | 612 | 547 | 705 | 109 | 113 | 133 | 254 | 94 | 79 | HS_25 | 2787 |  |
| ILMN_3168052 | 4988 | 876 | 1605 | 304 | 73 | 73 | 68 | 91 | 62 | 47 | HS_250 | 2788 |  |
| ILMN_3167879 | 132 | 166 | 128 | 169 | 80 | 73 | 66 | 91 | 57 | 325 | HS_251.1 | 2158 |  |
| ILMN_3168013 | 613 | 317 | 355 | 463 | 81 | 82 | 78 | 109 | 66 | 55 | HS_252.1 | 2160 |  |
| ILMN_3167604 | 101 | 109 | 85 | 86 | 78 | 76 | 75 | 97 | 65 | 59 | HS_253 | 2789 |  |
| ILMN_3168276 | 1252 | 133 | 218 | 89 | 69 | 70 | 68 | 88 | 57 | 49 | HS_254 | 2791 |  |
| ILMN_3167880 | 282 | 203 | 102 | 122 | 87 | 77 | 84 | 129 | 75 | 55 | HS_255 | 2794 |  |
| ILMN_3168458 | 210 | 345 | 412 | 64 | 431 | 72 | 72 | 90 | 61 | 46 | HS_257 | 2797 |  |
| ILMN_3167051 | 80 | 79 | 76 | 78 | 68 | 72 | 66 | 89 | 58 | 46 | HS_258 | 2798 |  |
| ILMN_3167497 | 222 | 106 | 96 | 112 | 76 | 74 | 78 | 96 | 70 | 44 | HS_26.1 | 2161 |  |
| ILMN_3167212 | 430 | 474 | 1598 | 237 | 75 | 72 | 69 | 93 | 60 | 55 | HS_260 | 2802 |  |
| ILMN_3167097 | 3290 | 83 | 97 | 113 | 68 | 66 | 66 | 625 | 216 | 43 | HS_261.1 | 2162 |  |
| ILMN_3167361 | 784 | 670 | 809 | 593 | 77 | 95 | 72 | 91 | 61 | 60 | HS_262.1 | 2163 |  |
| ILMN_3167705 | 5693 | 6663 | 3936 | 4233 | 71 | 67 | 70 | 368 | 57 | 47 | HS_263.1 | 2164 |  |
| ILMN_3167357 | 139 | 56 | 57 | 71 | 71 | 71 | 68 | 92 | 60 | 42 | HS_264.1 | 2166 |  |
| ILMN_3168195 | 1531 | 305 | 443 | 512 | 79 | 1058 | 241 | 173 | 66 | 57 | HS_265.1 | 2167 |  |
| ILMN_3168251 | 170 | 195 | 545 | 72 | 70 | 67 | 65 | 730 | 54 | 49 | HS_266.1 | 2168 |  |
| ILMN_3167573 | 104 | 142 | 107 | 111 | 97 | 91 | 90 | 227 | 75 | 64 | HS_267 | 2803 |  |
| ILMN_3167015 | 514 | 609 | 892 | 518 | 79 | 75 | 73 | 97 | 63 | 77 | HS_268 | 2804 |  |
| ILMN_3168084 | 271 | 321 | 143 | 124 | 119 | 101 | 124 | 118 | 101 | 101 | HS_269 | 2807 |  |
| ILMN_3167388 | 566 | 678 | 546 | 446 | 132 | 125 | 123 | 185 | 95 | 152 | HS_27 | 2808 |  |
| ILMN_3168134 | 112 | 116 | 110 | 101 | 74 | 70 | 68 | 92 | 61 | 49 | HS_273 | 2810 |  |
| ILMN_3167214 | 2405 | 3593 | 3229 | 2749 | 148 | 155 | 131 | 196 | 106 | 196 | HS_275 | 2813 |  |
| ILMN_3167740 | 2000 | 731 | 2469 | 834 | 3756 | 2700 | 4005 | 1338 | 2772 | 1682 | HS_276.1 | 2170 |  |
| ILMN_3167595 | 641 | 53 | 126 | 63 | 75 | 71 | 69 | 97 | 60 | 46 | HS_278 | 2816 |  |
| ILMN_3168016 | 420 | 586 | 577 | 692 | 135 | 118 | 122 | 155 | 102 | 92 | HS_279_a | 2818 |  |
| ILMN_3167202 | 97 | 286 | 250 | 92 | 75 | 73 | 71 | 95 | 60 | 48 | HS_280_a | 2827 |  |
| ILMN_3168169 | 1637 | 185 | 555 | 77 | 1199 | 69 | 68 | 3151 | 58 | 48 | HS_280_b | 2828 |  |
| ILMN_3167579 | 244 | 241 | 937 | 117 | 94 | 92 | 92 | 183 | 78 | 57 | HS_282 | 2833 |  |
| ILMN_3167538 | 329 | 191 | 175 | 175 | 78 | 77 | 77 | 110 | 62 | 63 | HS_283_a | 2834 |  |
| ILMN_3168032 | 86 | 204 | 59 | 67 | 65 | 62 | 62 | 80 | 54 | 43 | HS_283_b | 2838 |  |
| ILMN_3167405 | 53 | 52 | 56 | 63 | 75 | 74 | 74 | 94 | 63 | 48 | HS_284 | 2840 |  |
| ILMN_3168214 | 4035 | 3335 | 4235 | 1653 | 93 | 86 | 87 | 111 | 73 | 69 | HS_284.1 | 2174 |  |
| ILMN_3167975 | 239 | 390 | 310 | 438 | 80 | 81 | 74 | 103 | 76 | 56 | HS_285 | 2843 |  |
| ILMN_3167544 | 63 | 100 | 67 | 65 | 68 | 68 | 67 | 91 | 56 | 44 | HS_286_a | 2853 |  |
| ILMN_3168268 | 490 | 379 | 227 | 113 | 1210 | 72 | 65 | 158 | 58 | 49 | HS_287 | 2856 |  |
| ILMN_3167616 | 5398 | 18478 | 15365 | 4726 | 78 | 76 | 76 | 96 | 67 | 48 | HS_29 | 2857 |  |
| ILMN_3168047 | 385 | 218 | 44 | 55 | 67 | 66 | 66 | 86 | 55 | 41 | HS_3 | 2867 |  |
| ILMN_3168501 | 107 | 58 | 56 | 65 | 74 | 68 | 69 | 94 | 60 | 49 | HS_30 | 2868 |  |
| ILMN_3167546 | 873 | 1981 | 1622 | 837 | 93 | 304 | 91 | 159 | 64 | 484 | HS_303_a | 2879 |  |
| ILMN_3167085 | 835 | 714 | 568 | 1362 | 83 | 89 | 80 | 107 | 68 | 57 | HS_303_b | 2882 |  |
| ILMN_3167121 | 913 | 362 | 479 | 237 | 83 | 161 | 78 | 100 | 5221 | 59 | HS_304_a | 2883 |  |
| ILMN_3167750 | 148 | 110 | 104 | 103 | 75 | 77 | 72 | 94 | 120 | 53 | HS_304_b | 2885 |  |
| ILMN_3167155 | 494 | 102 | 1120 | 66 | 71 | 70 | 68 | 90 | 58 | 45 | HS_305_b | 2892 |  |
| ILMN_3167689 | 1364 | 1497 | 911 | 434 | 3903 | 89 | 94 | 5819 | 75 | 81 | HS_31.1 | 2184 |  |
| ILMN_3168078 | 2173 | 3356 | 2912 | 1089 | 131 | 129 | 117 | 150 | 90 | 5432 | HS_32 | 2901 |  |
| ILMN_3168382 | 2297 | 334 | 396 | 618 | 142 | 123 | 197 | 572 | 109 | 102 | HS_33 | 2902 |  |
| ILMN_3167886 | 120 | 156 | 58 | 68 | 71 | 68 | 65 | 93 | 56 | 45 | HS_35 | 2903 |  |
| ILMN_3167197 | 137 | 132 | 124 | 125 | 86 | 70 | 69 | 102 | 63 | 49 | HS_36.1 | 2185 |  |
| ILMN_3167058 | 122 | 224 | 112 | 809 | 75 | 71 | 74 | 94 | 60 | 55 | HS_37 | 2906 |  |
| ILMN_3168353 | 879 | 1008 | 1263 | 927 | 153 | 153 | 158 | 204 | 142 | 192 | HS_38.1 | 2187 |  |
| ILMN_3168060 | 124 | 101 | 114 | 106 | 78 | 75 | 69 | 97 | 63 | 56 | HS_4.1 | 2190 |  |
| ILMN_3168171 | 645 | 848 | 809 | 832 | 101 | 98 | 104 | 141 | 78 | 73 | HS_40 | 2907 |  |
| ILMN_3168359 | 69 | 62 | 63 | 80 | 72 | 70 | 68 | 93 | 58 | 46 | HS_41 | 2910 |  |
| ILMN_3167878 | 296 | 109 | 116 | 115 | 71 | 71 | 69 | 97 | 63 | 48 | HS_42 | 2913 |  |
| ILMN_3166945 | 103 | 151 | 126 | 115 | 74 | 71 | 71 | 89 | 59 | 49 | HS_43.1 | 2192 |  |
| ILMN_3167055 | 164 | 59 | 90 | 66 | 71 | 65 | 67 | 85 | 55 | 53 | HS_44.1 | 2195 |  |
| ILMN_3167767 | 1134 | 142 | 156 | 134 | 92 | 89 | 89 | 1788 | 74 | 70 | HS_45.1 | 2198 |  |
| ILMN_3168098 | 142 | 114 | 70 | 78 | 76 | 72 | 75 | 93 | 62 | 54 | HS_46 | 2915 |  |
| ILMN_3168414 | 429 | 418 | 395 | 396 | 82 | 78 | 76 | 116 | 64 | 62 | HS_47 | 2916 |  |
| ILMN_3167790 | 292 | 331 | 312 | 286 | 87 | 86 | 90 | 110 | 78 | 60 | HS_48.1 | 2202 |  |
| ILMN_3167054 | 296 | 307 | 296 | 303 | 76 | 74 | 69 | 98 | 59 | 51 | HS_49 | 2926 |  |
| ILMN_3168310 | 240 | 108 | 60 | 68 | 73 | 74 | 72 | 95 | 64 | 46 | HS_5.1 | 2204 |  |
| ILMN_3167882 | 156 | 305 | 114 | 141 | 71 | 71 | 69 | 93 | 60 | 48 | HS_50 | 2927 |  |
| ILMN_3167941 | 213 | 195 | 339 | 172 | 77 | 75 | 73 | 89 | 59 | 77 | HS_51 | 2930 |  |
| ILMN_3167364 | 5231 | 2208 | 2383 | 2692 | 8085 | 3555 | 2624 | 4492 | 1985 | 5591 | HS_52 | 2932 |  |
| ILMN_3167776 | 76 | 63 | 120 | 65 | 70 | 67 | 67 | 86 | 58 | 43 | HS_53 | 2934 |  |
| ILMN_3168384 | 262 | 452 | 200 | 157 | 70 | 68 | 69 | 85 | 2734 | 45 | HS_54 | 2939 |  |
| ILMN_3167251 | 194 | 108 | 593 | 60 | 68 | 67 | 63 | 87 | 56 | 46 | HS_55 | 2941 |  |
| ILMN_3167404 | 248 | 103 | 52 | 58 | 69 | 68 | 66 | 88 | 57 | 47 | HS_55.1 | 2205 |  |
| ILMN_3167249 | 295 | 83 | 1742 | 91 | 79 | 77 | 83 | 116 | 68 | 54 | HS_56 | 2943 |  |
| ILMN_3167398 | 248 | 141 | 136 | 146 | 74 | 72 | 74 | 98 | 60 | 46 | HS_57.1 | 2208 |  |
| ILMN_3167986 | 92 | 123 | 140 | 101 | 69 | 66 | 66 | 83 | 55 | 44 | HS_58 | 2951 |  |
| ILMN_3167707 | 84 | 192 | 77 | 83 | 85 | 76 | 95 | 100 | 64 | 55 | HS_59 | 2952 |  |
| ILMN_3167368 | 466 | 610 | 579 | 124 | 74 | 73 | 71 | 94 | 60 | 49 | HS_6 | 2954 |  |
| ILMN_3167469 | 274 | 900 | 570 | 1200 | 80 | 94 | 68 | 92 | 61 | 58 | HS_60 | 2960 |  |
| ILMN_3167813 | 56 | 46 | 48 | 55 | 66 | 65 | 64 | 84 | 54 | 40 | HS_61 | 2962 |  |
| ILMN_3168150 | 323 | 298 | 246 | 242 | 90 | 78 | 80 | 104 | 65 | 59 | HS_62 | 2964 |  |
| ILMN_3167310 | 208 | 173 | 155 | 148 | 73 | 74 | 70 | 96 | 60 | 51 | HS_63 | 2966 |  |
| ILMN_3167080 | 265 | 125 | 75 | 95 | 71 | 71 | 70 | 93 | 60 | 47 | HS_64 | 2968 |  |
| ILMN_3168269 | 441 | 742 | 3773 | 3639 | 2813 | 100 | 108 | 117 | 3819 | 165 | HS_65 | 2976 |  |
| ILMN_3167939 | 60 | 59 | 48 | 65 | 71 | 72 | 67 | 89 | 58 | 44 | HS_66 | 2983 |  |
| ILMN_3167440 | 75 | 107 | 615 | 92 | 75 | 73 | 70 | 96 | 59 | 49 | HS_67 | 2985 |  |
| ILMN_3167723 | 771 | 491 | 229 | 189 | 96 | 93 | 114 | 207 | 81 | 185 | HS_68 | 2986 |  |
| ILMN_3167460 | 702 | 49 | 39 | 60 | 68 | 67 | 66 | 90 | 55 | 42 | HS_69 | 2988 |  |
| ILMN_3167061 | 2640 | 137 | 133 | 273 | 93 | 90 | 154 | 2547 | 73 | 55 | HS_7 | 2989 |  |
| ILMN_3168010 | 94 | 60 | 48 | 64 | 68 | 66 | 66 | 86 | 57 | 42 | HS_70 | 2990 |  |
| ILMN_3167396 | 1761 | 1244 | 1189 | 1106 | 151 | 175 | 214 | 371 | 121 | 119 | HS_71.1 | 2209 |  |
| ILMN_3168161 | 887 | 319 | 633 | 93 | 76 | 70 | 74 | 94 | 58 | 50 | HS_72 | 2991 |  |
| ILMN_3167416 | 146 | 168 | 129 | 130 | 76 | 78 | 71 | 94 | 62 | 54 | HS_73.1 | 2215 |  |
| ILMN_3167289 | 682 | 443 | 108 | 93 | 74 | 74 | 74 | 96 | 62 | 49 | HS_74 | 2994 |  |
| ILMN_3167342 | 252 | 889 | 705 | 174 | 76 | 74 | 71 | 98 | 60 | 52 | HS_75.1 | 2216 |  |
| ILMN_3167881 | 1318 | 95 | 57 | 56 | 71 | 69 | 69 | 90 | 61 | 46 | HS_76 | 2995 |  |
| ILMN_3168466 | 174 | 90 | 54 | 70 | 75 | 72 | 72 | 93 | 72 | 49 | HS_77 | 2999 |  |
| ILMN_3167505 | 1826 | 815 | 835 | 348 | 113 | 96 | 115 | 146 | 94 | 78 | HS_78 | 5003 |  |
| ILMN_3167830 | 438 | 167 | 693 | 157 | 254 | 73 | 73 | 97 | 61 | 52 | HS_79.1 | 2222 |  |
| ILMN_3167836 | 294 | 170 | 179 | 124 | 73 | 184 | 71 | 194 | 60 | 57 | HS_8 | 5009 |  |
| ILMN_3168190 | 636 | 285 | 219 | 159 | 78 | 75 | 76 | 97 | 67 | 57 | HS_80 | 5013 |  |
| ILMN_3167479 | 996 | 1056 | 1102 | 925 | 119 | 119 | 120 | 160 | 90 | 113 | HS_81 | 5021 |  |
| ILMN_3167389 | 63 | 60 | 60 | 68 | 73 | 70 | 68 | 91 | 59 | 45 | HS_82.1 | 2225 |  |
| ILMN_3167782 | 106 | 143 | 60 | 68 | 87 | 86 | 88 | 108 | 72 | 49 | HS_83.1 | 2230 |  |
| ILMN_3168381 | 190 | 179 | 170 | 163 | 81 | 75 | 75 | 99 | 65 | 49 | HS_84 | 5022 |  |
| ILMN_3167926 | 82 | 81 | 67 | 72 | 68 | 67 | 65 | 86 | 55 | 46 | HS_85.1 | 2232 |  |
| ILMN_3167504 | 652 | 412 | 305 | 261 | 97 | 85 | 92 | 151 | 75 | 81 | HS_86 | 5025 |  |
| ILMN_3168377 | 641 | 68 | 386 | 57 | 76 | 71 | 74 | 96 | 58 | 48 | HS_87 | 5035 |  |
| ILMN_3167877 | 128 | 161 | 120 | 127 | 72 | 72 | 66 | 87 | 58 | 51 | HS_88 | 5037 |  |
| ILMN_3168065 | 3946 | 137 | 403 | 137 | 86 | 79 | 71 | 8215 | 66 | 51 | HS_89 | 5044 |  |
| ILMN_3168453 | 840 | 66 | 82 | 117 | 67 | 66 | 66 | 1782 | 214 | 44 | HS_9 | 5047 |  |
| ILMN_3168036 | 382 | 393 | 432 | 472 | 83 | 79 | 82 | 127 | 68 | 57 | HS_90 | 5050 |  |
| ILMN_3167590 | 2432 | 1777 | 4005 | 2805 | 68 | 67 | 67 | 85 | 56 | 44 | HS_91.1 | 2236 |  |
| ILMN_3168044 | 135 | 138 | 146 | 157 | 72 | 72 | 68 | 91 | 60 | 51 | HS_92 | 5052 |  |
| ILMN_3167449 | 187 | 158 | 160 | 161 | 88 | 92 | 103 | 149 | 93 | 67 | HS_93 | 5054 |  |
| ILMN_3167802 | 953 | 2268 | 2045 | 957 | 116 | 408 | 260 | 115 | 831 | 615 | HS_94 | 5055 |  |
| ILMN_3166964 | 167 | 95 | 104 | 102 | 83 | 81 | 72 | 107 | 71 | 70 | HS_95 | 5056 |  |
| ILMN_3168020 | 7858 | 8283 | 7515 | 6627 | 1507 | 1204 | 1470 | 2390 | 1370 | 1695 | HS_96 | 5057 |  |
| ILMN_3168153 | 2487 | 1760 | 1736 | 2271 | 182 | 180 | 189 | 389 | 145 | 126 | HS_97 | 5058 |  |
| ILMN_3167429 | 454 | 233 | 258 | 212 | 104 | 96 | 116 | 112 | 96 | 69 | HS_99.1 | 2252 |  |
| ILMN_3167971 | 28101 | 29158 | 30451 | 28620 | 76 | 3217 | 70 | 1828 | 63 | 851 | hsa-let-7a | 101 |  |
| ILMN_3168708 | 93 | 72 | 58 | 73 | 74 | 69 | 68 | 89 | 226 | 45 | hsa-let-7a* | 3677 |  |
| ILMN_3167970 | 21251 | 23368 | 25785 | 25087 | 3062 | 4308 | 1448 | 372 | 59 | 3249 | hsa-let-7b | 102 |  |
| ILMN_3167699 | 5886 | 4464 | 8455 | 6777 | 2217 | 6341 | 203 | 4398 | 65 | 52 | hsa-let-7b* | 51 |  |
| ILMN_3168513 | 15455 | 14986 | 17505 | 17267 | 102 | 103 | 103 | 144 | 76 | 70 | hsa-let-7c | 103 |  |
| ILMN_3168792 | 199 | 454 | 568 | 77 | 73 | 72 | 168 | 104 | 59 | 50 | hsa-let-7c* | 5858 |  |
| ILMN_3167551 | 17266 | 19995 | 20827 | 19927 | 71 | 1256 | 67 | 86 | 57 | 43 | hsa-let-7d | 105 |  |
| ILMN_3168710 | 8328 | 3393 | 6228 | 6688 | 5414 | 634 | 5761 | 6063 | 62 | 10211 | hsa-let-7d* | 3011 |  |
| ILMN_3168463 | 20890 | 21700 | 20859 | 15793 | 106 | 2279 | 108 | 163 | 85 | 86 | hsa-let-7e | 109 |  |
| ILMN_3168711 | 908 | 939 | 517 | 410 | 136 | 136 | 136 | 154 | 111 | 77 | hsa-let-7e* | 3842 |  |
| ILMN_3167189 | 8589 | 19582 | 18717 | 15244 | 67 | 1345 | 63 | 84 | 54 | 42 | hsa-let-7f | 110 |  |
| ILMN_3167319 | 192 | 177 | 153 | 139 | 72 | 97 | 62 | 83 | 54 | 70 | hsa-let-7f-1* | 5643 |  |
| ILMN_3168709 | 526 | 445 | 485 | 432 | 77 | 70 | 68 | 110 | 59 | 58 | hsa-let-7f-2* | 6131 |  |
| ILMN_3168365 | 22031 | 28128 | 30209 | 27650 | 79 | 2875 | 79 | 1834 | 67 | 1337 | hsa-let-7g | 111 |  |
| ILMN_3168732 | 991 | 194 | 473 | 164 | 75 | 72 | 67 | 90 | 60 | 53 | hsa-let-7g* | 4204 |  |
| ILMN_3168316 | 18387 | 22903 | 23849 | 23573 | 94 | 2413 | 87 | 110 | 74 | 222 | hsa-let-7i | 113 |  |
| ILMN_3168724 | 589 | 205 | 244 | 239 | 73 | 69 | 68 | 89 | 59 | 49 | hsa-let-7i* | 3914 |  |
| ILMN_3168320 | 205 | 58 | 52 | 63 | 69 | 68 | 65 | 88 | 55 | 42 | hsa-miR-1 | 119 |  |
| ILMN_3167634 | 8533 | 12177 | 9395 | 14570 | 90 | 91 | 90 | 115 | 75 | 58 | hsa-miR-100 | 121 |  |
| ILMN_3168650 | 343 | 427 | 418 | 442 | 74 | 75 | 73 | 96 | 62 | 51 | hsa-miR-100* | 3294 |  |
| ILMN_3167808 | 1008 | 3658 | 949 | 3677 | 105 | 110 | 105 | 143 | 84 | 90 | hsa-miR-101 | 122 |  |
| ILMN_3168196 | 97 | 317 | 834 | 266 | 76 | 71 | 69 | 92 | 58 | 48 | hsa-miR-101* | 3461 |  |
| ILMN_3167027 | 4424 | 5516 | 5092 | 3769 | 85 | 2488 | 100 | 152 | 82 | 67 | hsa-miR-103 | 124 |  |
| ILMN_3167294 | 1473 | 1216 | 1402 | 1251 | 759 | 368 | 542 | 397 | 396 | 247 | hsa-miR-105 | 125 |  |
| ILMN_3168583 | 161 | 134 | 121 | 144 | 72 | 70 | 70 | 92 | 58 | 63 | hsa-miR-105* | 3910 |  |
| ILMN_3168544 | 4409 | 6221 | 5697 | 5269 | 70 | 190 | 66 | 87 | 56 | 43 | hsa-miR-106a | 5214 |  |
| ILMN_3168721 | 226 | 197 | 77 | 84 | 80 | 73 | 75 | 105 | 65 | 58 | hsa-miR-106a* | 3377 |  |
| ILMN_3168107 | 353 | 204 | 258 | 577 | 84 | 84 | 85 | 118 | 70 | 60 | hsa-miR-106a:9.1 | 130 |  |
| ILMN_3167865 | 864 | 3147 | 2643 | 2761 | 73 | 69 | 65 | 90 | 60 | 47 | hsa-miR-106b | 140 |  |
| ILMN_3168681 | 3106 | 2034 | 1249 | 813 | 79 | 76 | 71 | 94 | 63 | 57 | hsa-miR-106b* | 4057 |  |
| ILMN_3167353 | 799 | 1595 | 384 | 617 | 86 | 86 | 82 | 106 | 70 | 48 | hsa-miR-107 | 147 |  |
| ILMN_3167552 | 2643 | 4509 | 7246 | 4747 | 72 | 71 | 69 | 92 | 60 | 46 | hsa-miR-10a | 152 |  |
| ILMN_3168643 | 808 | 3109 | 2987 | 916 | 137 | 129 | 121 | 141 | 103 | 101 | hsa-miR-10a* | 6080 |  |
| ILMN_3167276 | 2157 | 5586 | 5695 | 4274 | 70 | 68 | 67 | 93 | 59 | 44 | hsa-miR-10b | 153 |  |
| ILMN_3168573 | 118 | 127 | 119 | 110 | 76 | 75 | 72 | 91 | 61 | 58 | hsa-miR-10b* | 6085 |  |
| ILMN_3168877 | 75 | 80 | 77 | 86 | 70 | 70 | 66 | 88 | 58 | 1574 | hsa-miR-1178 | 5145 |  |
| ILMN_3168560 | 917 | 2041 | 1680 | 1066 | 2533 | 3115 | 67 | 85 | 3888 | 2921 | hsa-miR-1179 | 5678 |  |
| ILMN_3168881 | 1014 | 418 | 962 | 557 | 90 | 119 | 1426 | 1877 | 106 | 55 | hsa-miR-1180 | 30 |  |
| ILMN_3168682 | 3410 | 4528 | 1176 | 2040 | 7331 | 2169 | 71 | 2630 | 58 | 76 | hsa-miR-1181 | 5123 |  |
| ILMN_3168745 | 208 | 139 | 224 | 90 | 73 | 73 | 69 | 1336 | 106 | 4171 | hsa-miR-1182 | 5237 |  |
| ILMN_3168659 | 317 | 172 | 248 | 77 | 5136 | 5759 | 6793 | 1685 | 2281 | 2195 | hsa-miR-1183 | 1588 |  |
| ILMN_3168689 | 489 | 661 | 1327 | 2848 | 176 | 88 | 6581 | 1849 | 75 | 2819 | hsa-miR-1184 | 3942 |  |
| ILMN_3168241 | 80 | 59 | 48 | 65 | 71 | 68 | 75 | 91 | 66 | 56 | hsa-miR-1185 | 2721 |  |
| ILMN_3168794 | 634 | 332 | 291 | 2413 | 11631 | 85 | 872 | 211 | 77 | 102 | hsa-miR-1197 | 5244 |  |
| ILMN_3168717 | 375 | 115 | 111 | 98 | 72 | 76 | 68 | 90 | 60 | 51 | hsa-miR-1200 | 4202 |  |
| ILMN_3168604 | 14214 | 15111 | 16481 | 6489 | 68 | 66 | 65 | 88 | 55 | 44 | hsa-miR-1201 | 3630 |  |
| ILMN_3168773 | 225 | 115 | 109 | 115 | 74 | 71 | 69 | 95 | 63 | 51 | hsa-miR-1202 | 3049 |  |
| ILMN_3168680 | 109 | 164 | 84 | 99 | 71 | 71 | 68 | 90 | 62 | 46 | hsa-miR-1203 | 4106 |  |
| ILMN_3168822 | 1068 | 1036 | 907 | 624 | 134 | 149 | 198 | 259 | 108 | 186 | hsa-miR-1204 | 2862 |  |
| ILMN_3168827 | 341 | 400 | 568 | 369 | 88 | 91 | 87 | 336 | 74 | 99 | hsa-miR-1205 | 4931 |  |
| ILMN_3168864 | 915 | 938 | 1119 | 677 | 91 | 88 | 88 | 131 | 78 | 85 | hsa-miR-1206 | 3936 |  |
| ILMN_3168809 | 141 | 119 | 183 | 56 | 3238 | 70 | 64 | 87 | 57 | 48 | hsa-miR-1207-3p | 3191 |  |
| ILMN_3168852 | 1138 | 1171 | 976 | 852 | 104 | 97 | 96 | 114 | 88 | 122 | hsa-miR-1207-5p | 3314 |  |
| ILMN_3168808 | 416 | 358 | 352 | 334 | 108 | 102 | 102 | 132 | 86 | 81 | hsa-miR-1208 | 3856 |  |
| ILMN_3167547 | 1225 | 654 | 447 | 518 | 625 | 6694 | 6503 | 97 | 64 | 4492 | hsa-miR-122 | 154 |  |
| ILMN_3168556 | 149 | 158 | 172 | 168 | 79 | 80 | 74 | 94 | 63 | 56 | hsa-miR-122* | 5567 |  |
| ILMN_3168679 | 1705 | 3810 | 2104 | 1654 | 133 | 128 | 136 | 214 | 103 | 152 | hsa-miR-1224-3p | 4018 |  |
| ILMN_3168771 | 196 | 401 | 357 | 77 | 68 | 66 | 66 | 88 | 57 | 43 | hsa-miR-1224-5p | 3500 |  |
| ILMN_3168832 | 2075 | 1633 | 1903 | 1615 | 199 | 170 | 164 | 434 | 142 | 155 | hsa-miR-1225-3p | 4954 |  |
| ILMN_3168775 | 878 | 618 | 462 | 1885 | 2699 | 4703 | 8649 | 5928 | 7094 | 2159 | hsa-miR-1225-5p | 5246 |  |
| ILMN_3168807 | 516 | 77 | 115 | 67 | 71 | 70 | 70 | 89 | 59 | 917 | hsa-miR-1226 | 4074 |  |
| ILMN_3168772 | 314 | 283 | 222 | 140 | 78 | 77 | 81 | 120 | 63 | 49 | hsa-miR-1226* | 3285 |  |
| ILMN_3168702 | 266 | 96 | 61 | 78 | 72 | 69 | 68 | 91 | 61 | 47 | hsa-miR-1227 | 4857 |  |
| ILMN_3168805 | 281 | 115 | 108 | 114 | 69 | 84 | 64 | 90 | 58 | 51 | hsa-miR-1228 | 2180 |  |
| ILMN_3168118 | 9652 | 6118 | 10626 | 5675 | 98 | 100 | 107 | 139 | 77 | 3308 | hsa-miR-1228* | 3988 |  |
| ILMN_3167337 | 811 | 311 | 300 | 237 | 97 | 94 | 97 | 127 | 85 | 55 | hsa-miR-1229 | 5060 |  |
| ILMN_3167675 | 4047 | 1063 | 883 | 842 | 109 | 94 | 100 | 2105 | 87 | 64 | hsa-miR-1231 | 5117 |  |
| ILMN_3167853 | 1707 | 1837 | 1481 | 1371 | 206 | 182 | 210 | 353 | 162 | 252 | hsa-miR-1233 | 5880 |  |
| ILMN_3168821 | 1199 | 2732 | 666 | 176 | 86 | 87 | 82 | 109 | 71 | 74 | hsa-miR-1234 | 6107 |  |
| ILMN_3168688 | 98 | 54 | 31 | 28 | 69 | 69 | 61 | 89 | 58 | 62 | hsa-miR-1236 | 4325 |  |
| ILMN_3168818 | 240 | 506 | 272 | 68 | 66 | 67 | 65 | 86 | 55 | 44 | hsa-miR-1237 | 4010 |  |
| ILMN_3168737 | 27742 | 12287 | 22237 | 22904 | 12332 | 13105 | 19475 | 22755 | 18549 | 11511 | hsa-miR-1238 | 3800 |  |
| ILMN_3168781 | 98 | 55 | 52 | 66 | 70 | 69 | 73 | 87 | 58 | 45 | hsa-miR-124 | 5329 |  |
| ILMN_3168704 | 69 | 44 | 39 | 54 | 618 | 65 | 61 | 81 | 55 | 43 | hsa-miR-124* | 4208 |  |
| ILMN_3168557 | 304 | 329 | 501 | 354 | 82 | 90 | 78 | 101 | 67 | 61 | hsa-miR-1243 | 3158 |  |
| ILMN_3168565 | 332 | 839 | 1077 | 181 | 68 | 68 | 64 | 86 | 56 | 46 | hsa-miR-1244 | 87 |  |
| ILMN_3168566 | 1013 | 953 | 857 | 1438 | 69 | 87 | 67 | 95 | 58 | 58 | hsa-miR-1245 | 5508 |  |
| ILMN_3168571 | 24307 | 9385 | 22102 | 9365 | 2227 | 2683 | 10440 | 1509 | 55 | 46 | hsa-miR-1246 | 3970 |  |
| ILMN_3168578 | 1236 | 1224 | 1118 | 1905 | 83 | 66 | 69 | 85 | 56 | 65 | hsa-miR-1247 | 6035 |  |
| ILMN_3168581 | 2885 | 5698 | 5540 | 1018 | 81 | 80 | 77 | 110 | 64 | 51 | hsa-miR-1248 | 1600 |  |
| ILMN_3168582 | 899 | 811 | 531 | 331 | 75 | 82 | 74 | 98 | 62 | 56 | hsa-miR-1249 | 5550 |  |
| ILMN_3168039 | 77 | 66 | 64 | 75 | 472 | 71 | 70 | 370 | 61 | 50 | hsa-miR-124a:9.1 | 162 |  |
| ILMN_3168585 | 397 | 183 | 324 | 108 | 91 | 81 | 82 | 106 | 69 | 54 | hsa-miR-1250 | 1595 |  |
| ILMN_3167649 | 217 | 110 | 126 | 100 | 72 | 70 | 66 | 88 | 57 | 59 | hsa-miR-1251 | 2612 |  |
| ILMN_3168596 | 267 | 67 | 67 | 67 | 77 | 70 | 73 | 97 | 61 | 49 | hsa-miR-1252 | 5548 |  |
| ILMN_3168598 | 107 | 92 | 92 | 106 | 92 | 86 | 95 | 170 | 73 | 55 | hsa-miR-1253 | 4173 |  |
| ILMN_3168605 | 18528 | 2858 | 3949 | 5734 | 542 | 3191 | 3343 | 12550 | 6544 | 3421 | hsa-miR-1254 | 4156 |  |
| ILMN_3168607 | 67 | 62 | 627 | 553 | 69 | 67 | 67 | 86 | 58 | 46 | hsa-miR-1255a | 3592 |  |
| ILMN_3168695 | 209 | 306 | 160 | 746 | 93 | 89 | 86 | 110 | 81 | 72 | hsa-miR-1255b | 3981 |  |
| ILMN_3168608 | 185 | 326 | 243 | 228 | 74 | 75 | 71 | 95 | 63 | 57 | hsa-miR-1256 | 3977 |  |
| ILMN_3168618 | 1678 | 1033 | 1021 | 1070 | 182 | 152 | 189 | 289 | 146 | 730 | hsa-miR-1257 | 5981 |  |
| ILMN_3168620 | 349 | 140 | 471 | 39 | 70 | 70 | 65 | 89 | 60 | 65 | hsa-miR-1258 | 5297 |  |
| ILMN_3168626 | 87 | 116 | 199 | 152 | 80 | 72 | 72 | 92 | 63 | 50 | hsa-miR-1259 | 4900 |  |
| ILMN_3168574 | 1510 | 913 | 1667 | 1299 | 101 | 90 | 92 | 201 | 106 | 108 | hsa-miR-125a-3p | 3087 |  |
| ILMN_3167670 | 12250 | 11929 | 17851 | 20857 | 74 | 77 | 71 | 93 | 62 | 51 | hsa-miR-125a-5p | 171 |  |
| ILMN_3168389 | 10171 | 20675 | 21394 | 23910 | 69 | 72 | 67 | 87 | 57 | 50 | hsa-miR-125b | 172 |  |
| ILMN_3168584 | 242 | 453 | 235 | 190 | 79 | 85 | 75 | 101 | 62 | 94 | hsa-miR-125b-1* | 3340 |  |
| ILMN_3168804 | 618 | 3576 | 2755 | 2687 | 80 | 77 | 78 | 103 | 68 | 49 | hsa-miR-125b-2* | 3561 |  |
| ILMN_3167695 | 5127 | 6125 | 6872 | 4104 | 548 | 573 | 515 | 1042 | 432 | 920 | hsa-miR-126 | 174 |  |
| ILMN_3168399 | 15161 | 21719 | 21358 | 15709 | 82 | 8912 | 74 | 4449 | 63 | 7873 | hsa-miR-126* | 178 |  |
| ILMN_3168628 | 22167 | 8930 | 16020 | 5793 | 75 | 4628 | 72 | 94 | 62 | 48 | hsa-miR-1260 | 6038 |  |
| ILMN_3168631 | 187 | 249 | 224 | 194 | 69 | 70 | 81 | 89 | 56 | 49 | hsa-miR-1261 | 3027 |  |
| ILMN_3168632 | 590 | 532 | 387 | 658 | 134 | 2272 | 122 | 162 | 104 | 99 | hsa-miR-1262 | 4148 |  |
| ILMN_3168633 | 423 | 169 | 143 | 232 | 78 | 83 | 98 | 202 | 76 | 55 | hsa-miR-1263 | 4044 |  |
| ILMN_3168651 | 105 | 99 | 101 | 104 | 81 | 79 | 67 | 87 | 57 | 58 | hsa-miR-1264 | 5114 |  |
| ILMN_3168661 | 74 | 82 | 61 | 75 | 74 | 67 | 71 | 94 | 60 | 47 | hsa-miR-1265 | 3841 |  |
| ILMN_3168685 | 53 | 49 | 134 | 65 | 66 | 64 | 61 | 82 | 57 | 46 | hsa-miR-1266 | 4248 |  |
| ILMN_3168692 | 1379 | 1184 | 1128 | 1179 | 124 | 124 | 132 | 200 | 96 | 157 | hsa-miR-1267 | 3063 |  |
| ILMN_3168697 | 2015 | 684 | 1383 | 773 | 74 | 73 | 71 | 93 | 60 | 50 | hsa-miR-1268 | 3510 |  |
| ILMN_3168725 | 156 | 195 | 157 | 118 | 73 | 85 | 70 | 94 | 63 | 72 | hsa-miR-1269 | 5798 |  |
| ILMN_3168726 | 202 | 192 | 168 | 603 | 79 | 73 | 74 | 99 | 65 | 53 | hsa-miR-1270 | 5073 |  |
| ILMN_3168738 | 2588 | 3154 | 3507 | 1842 | 91 | 85 | 83 | 107 | 71 | 58 | hsa-miR-1271 | 3051 |  |
| ILMN_3168746 | 932 | 1359 | 1323 | 1221 | 109 | 94 | 81 | 147 | 68 | 60 | hsa-miR-1272 | 3245 |  |
| ILMN_3168760 | 188 | 2022 | 979 | 1545 | 72 | 70 | 68 | 91 | 60 | 50 | hsa-miR-1273 | 3155 |  |
| ILMN_3167031 | 1736 | 4985 | 3970 | 2772 | 202 | 138 | 221 | 268 | 156 | 109 | hsa-miR-127-3p | 182 |  |
| ILMN_3168769 | 5461 | 1743 | 4947 | 799 | 83 | 91 | 81 | 110 | 63 | 102 | hsa-miR-1274a | 4985 |  |
| ILMN_3168816 | 25625 | 14739 | 21074 | 15764 | 66 | 4295 | 60 | 79 | 53 | 41 | hsa-miR-1274b | 4256 |  |
| ILMN_3168774 | 3318 | 1800 | 3549 | 3542 | 75 | 75 | 71 | 88 | 60 | 48 | hsa-miR-1275 | 6052 |  |
| ILMN_3168719 | 78 | 184 | 412 | 64 | 77 | 67 | 64 | 85 | 58 | 44 | hsa-miR-127-5p | 5639 |  |
| ILMN_3168778 | 145 | 156 | 145 | 151 | 89 | 79 | 82 | 104 | 72 | 77 | hsa-miR-1276 | 4160 |  |
| ILMN_3168787 | 62 | 56 | 52 | 72 | 70 | 70 | 70 | 87 | 58 | 43 | hsa-miR-1277 | 3283 |  |
| ILMN_3168795 | 90 | 228 | 41 | 38 | 70 | 68 | 66 | 83 | 1850 | 60 | hsa-miR-1278 | 4229 |  |
| ILMN_3168811 | 205 | 96 | 92 | 103 | 83 | 77 | 77 | 125 | 64 | 56 | hsa-miR-1279 | 5733 |  |
| ILMN_3168806 | 7866 | 3334 | 5265 | 965 | 89 | 1011 | 139 | 335 | 85 | 55 | hsa-miR-128 | 3633 |  |
| ILMN_3168814 | 8538 | 9428 | 9167 | 7670 | 515 | 2120 | 469 | 412 | 395 | 7403 | hsa-miR-1280 | 3295 |  |
| ILMN_3168820 | 492 | 2800 | 140 | 536 | 83 | 78 | 78 | 102 | 61 | 55 | hsa-miR-1281 | 3420 |  |
| ILMN_3168823 | 5465 | 262 | 392 | 358 | 1078 | 538 | 1994 | 9521 | 5247 | 1820 | hsa-miR-1282 | 3144 |  |
| ILMN_3168824 | 87 | 106 | 79 | 108 | 77 | 75 | 71 | 100 | 68 | 59 | hsa-miR-1283 | 6137 |  |
| ILMN_3168825 | 1919 | 1172 | 1067 | 944 | 192 | 176 | 228 | 555 | 173 | 185 | hsa-miR-1284 | 5599 |  |
| ILMN_3168828 | 2660 | 2565 | 2279 | 2018 | 73 | 3324 | 69 | 96 | 59 | 47 | hsa-miR-1285 | 4071 |  |
| ILMN_3168839 | 851 | 764 | 765 | 943 | 116 | 110 | 114 | 151 | 84 | 108 | hsa-miR-1286 | 3083 |  |
| ILMN_3168848 | 932 | 446 | 554 | 116 | 70 | 70 | 70 | 91 | 57 | 63 | hsa-miR-1287 | 3096 |  |
| ILMN_3168849 | 380 | 261 | 135 | 139 | 84 | 74 | 77 | 117 | 65 | 54 | hsa-miR-1288 | 3833 |  |
| ILMN_3168850 | 2806 | 330 | 441 | 620 | 133 | 239 | 497 | 2727 | 617 | 137 | hsa-miR-1289 | 4216 |  |
| ILMN_3168085 | 611 | 77 | 189 | 70 | 79 | 587 | 77 | 93 | 63 | 51 | hsa-miR-128a:9.1 | 185 |  |
| ILMN_3167491 | 556 | 119 | 255 | 127 | 82 | 80 | 76 | 103 | 65 | 143 | hsa-miR-128b:9.1 | 186 |  |
| ILMN_3168562 | 224 | 226 | 875 | 101 | 79 | 76 | 75 | 109 | 63 | 52 | hsa-miR-129* | 3085 |  |
| ILMN_3168851 | 3661 | 8402 | 6893 | 1540 | 810 | 488 | 1055 | 4735 | 584 | 288 | hsa-miR-1290 | 3481 |  |
| ILMN_3168853 | 251 | 222 | 113 | 108 | 71 | 70 | 69 | 93 | 60 | 46 | hsa-miR-1291 | 4951 |  |
| ILMN_3168854 | 125 | 57 | 60 | 62 | 66 | 64 | 62 | 86 | 56 | 43 | hsa-miR-1292 | 5600 |  |
| ILMN_3168857 | 1807 | 334 | 215 | 162 | 82 | 77 | 74 | 101 | 60 | 65 | hsa-miR-1293 | 3125 |  |
| ILMN_3168561 | 68 | 512 | 459 | 67 | 68 | 66 | 66 | 87 | 56 | 46 | hsa-miR-129-3p | 3014 |  |
| ILMN_3168862 | 924 | 927 | 750 | 403 | 81 | 83 | 78 | 112 | 68 | 91 | hsa-miR-1294 | 3858 |  |
| ILMN_3168865 | 1545 | 1203 | 1411 | 1929 | 101 | 92 | 94 | 113 | 79 | 64 | hsa-miR-1295 | 3736 |  |
| ILMN_3168183 | 15354 | 5460 | 5730 | 5767 | 1670 | 451 | 1334 | 11413 | 860 | 626 | hsa-miR-129-5p | 205 |  |
| ILMN_3167351 | 1525 | 1011 | 1513 | 484 | 69 | 70 | 68 | 89 | 57 | 46 | hsa-miR-1296 | 2373 |  |
| ILMN_3168867 | 343 | 5962 | 1714 | 479 | 91 | 83 | 80 | 103 | 65 | 61 | hsa-miR-1297 | 6012 |  |
| ILMN_3168109 | 136 | 135 | 142 | 155 | 74 | 76 | 71 | 91 | 60 | 51 | hsa-miR-1298 | 2434 |  |
| ILMN_3168873 | 334 | 332 | 438 | 799 | 79 | 91 | 6313 | 97 | 64 | 75 | hsa-miR-1299 | 5361 |  |
| ILMN_3168875 | 1121 | 1470 | 1913 | 3110 | 84 | 1103 | 3820 | 100 | 66 | 65 | hsa-miR-1300 | 5962 |  |
| ILMN_3168876 | 8704 | 1924 | 1590 | 1794 | 146 | 2324 | 1576 | 9444 | 3892 | 3084 | hsa-miR-1301 | 5093 |  |
| ILMN_3168878 | 111 | 84 | 76 | 93 | 76 | 73 | 72 | 94 | 66 | 50 | hsa-miR-1302 | 3574 |  |
| ILMN_3168880 | 1671 | 619 | 127 | 81 | 4489 | 70 | 67 | 187 | 57 | 51 | hsa-miR-1303 | 5188 |  |
| ILMN_3168882 | 735 | 265 | 214 | 1571 | 79 | 72 | 70 | 4965 | 60 | 50 | hsa-miR-1304 | 4214 |  |
| ILMN_3168883 | 857 | 1009 | 1387 | 802 | 132 | 133 | 127 | 174 | 103 | 118 | hsa-miR-1305 | 1587 |  |
| ILMN_3168587 | 4980 | 1941 | 3573 | 1085 | 4417 | 75 | 72 | 2337 | 62 | 51 | hsa-miR-1307 | 3919 |  |
| ILMN_3168750 | 26202 | 10276 | 25567 | 15220 | 70 | 1722 | 65 | 94 | 57 | 47 | hsa-miR-1308 | 5816 |  |
| ILMN_3168497 | 4445 | 11480 | 10017 | 12816 | 132 | 571 | 92 | 1587 | 1300 | 1270 | hsa-miR-130a | 206 |  |
| ILMN_3168870 | 5496 | 390 | 1871 | 1972 | 260 | 88 | 2409 | 4866 | 1993 | 153 | hsa-miR-130a* | 3780 |  |
| ILMN_3167099 | 357 | 2125 | 455 | 1200 | 480 | 84 | 83 | 105 | 68 | 60 | hsa-miR-130b | 208 |  |
| ILMN_3168588 | 416 | 466 | 455 | 455 | 73 | 85 | 66 | 93 | 55 | 4624 | hsa-miR-130b* | 6020 |  |
| ILMN_3168212 | 6272 | 21238 | 22020 | 13187 | 72 | 73 | 68 | 4145 | 58 | 2772 | hsa-miR-132 | 209 |  |
| ILMN_3168579 | 433 | 1210 | 549 | 285 | 76 | 76 | 69 | 110 | 63 | 91 | hsa-miR-132* | 4947 |  |
| ILMN_3168663 | 146 | 113 | 111 | 101 | 73 | 70 | 67 | 87 | 56 | 49 | hsa-miR-1321 | 3697 |  |
| ILMN_3168747 | 206 | 216 | 217 | 484 | 81 | 87 | 74 | 109 | 62 | 65 | hsa-miR-1322 | 5517 |  |
| ILMN_3168803 | 1261 | 1492 | 1198 | 965 | 110 | 104 | 107 | 177 | 84 | 81 | hsa-miR-1323 | 5943 |  |
| ILMN_3168675 | 89 | 77 | 69 | 81 | 76 | 69 | 94 | 96 | 60 | 51 | hsa-miR-1324 | 3648 |  |
| ILMN_3168314 | 920 | 1251 | 926 | 988 | 288 | 203 | 290 | 200 | 222 | 190 | hsa-miR-133a | 210 |  |
| ILMN_3168348 | 518 | 103 | 108 | 129 | 78 | 72 | 71 | 414 | 60 | 47 | hsa-miR-133b | 213 |  |
| ILMN_3168025 | 2800 | 7971 | 6374 | 7323 | 3342 | 2014 | 72 | 155 | 61 | 47 | hsa-miR-134 | 215 |  |
| ILMN_3167823 | 267 | 615 | 342 | 476 | 118 | 96 | 105 | 131 | 87 | 86 | hsa-miR-135a | 220 |  |
| ILMN_3168798 | 653 | 330 | 308 | 316 | 107 | 107 | 139 | 208 | 80 | 60 | hsa-miR-135a* | 4186 |  |
| ILMN_3167874 | 365 | 448 | 296 | 324 | 83 | 82 | 85 | 117 | 69 | 53 | hsa-miR-135b | 223 |  |
| ILMN_3168635 | 266 | 218 | 259 | 191 | 74 | 1857 | 73 | 93 | 61 | 48 | hsa-miR-135b* | 5241 |  |
| ILMN_3167624 | 141 | 702 | 117 | 128 | 75 | 72 | 76 | 100 | 62 | 52 | hsa-miR-136 | 230 |  |
| ILMN_3168667 | 79 | 358 | 449 | 134 | 67 | 69 | 65 | 82 | 56 | 46 | hsa-miR-136* | 5475 |  |
| ILMN_3166940 | 494 | 291 | 256 | 248 | 89 | 107 | 98 | 200 | 79 | 91 | hsa-miR-137 | 233 |  |
| ILMN_3167501 | 303 | 408 | 373 | 738 | 91 | 88 | 84 | 113 | 76 | 64 | hsa-miR-138 | 234 |  |
| ILMN_3168752 | 161 | 281 | 127 | 136 | 98 | 92 | 93 | 113 | 80 | 73 | hsa-miR-138-1* | 4304 |  |
| ILMN_3168753 | 270 | 318 | 252 | 239 | 86 | 78 | 82 | 113 | 72 | 62 | hsa-miR-138-2* | 5698 |  |
| ILMN_3167685 | 3907 | 1918 | 2925 | 1472 | 81 | 76 | 80 | 105 | 65 | 76 | hsa-miR-139-3p | 3879 |  |
| ILMN_3166992 | 7061 | 9551 | 14499 | 6004 | 86 | 86 | 81 | 148 | 69 | 64 | hsa-miR-139-5p | 240 |  |
| ILMN_3168786 | 8675 | 10238 | 8482 | 8098 | 2660 | 5843 | 1845 | 8192 | 5185 | 5116 | hsa-miR-140-3p | 5177 |  |
| ILMN_3167136 | 410 | 341 | 316 | 336 | 80 | 82 | 78 | 104 | 71 | 67 | hsa-miR-140-5p | 247 |  |
| ILMN_3168064 | 314 | 6137 | 4415 | 5261 | 76 | 72 | 72 | 99 | 1036 | 55 | hsa-miR-141 | 256 |  |
| ILMN_3168669 | 159 | 78 | 90 | 112 | 68 | 67 | 67 | 91 | 56 | 53 | hsa-miR-141* | 3588 |  |
| ILMN_3168012 | 202 | 709 | 352 | 1397 | 74 | 73 | 71 | 92 | 60 | 51 | hsa-miR-142-3p | 261 |  |
| ILMN_3168509 | 129 | 216 | 72 | 74 | 79 | 437 | 65 | 90 | 58 | 46 | hsa-miR-142-5p | 263 |  |
| ILMN_3166958 | 17447 | 28238 | 28523 | 26384 | 88 | 87 | 79 | 101 | 73 | 90 | hsa-miR-143 | 265 |  |
| ILMN_3168764 | 4270 | 4233 | 3206 | 2322 | 384 | 288 | 311 | 2477 | 280 | 514 | hsa-miR-143* | 4250 |  |
| ILMN_3168192 | 129 | 88 | 104 | 83 | 117 | 114 | 5647 | 135 | 90 | 2115 | hsa-miR-144 | 4222 |  |
| ILMN_3168756 | 405 | 811 | 1464 | 211 | 64 | 65 | 62 | 80 | 53 | 43 | hsa-miR-144* | 5322 |  |
| ILMN_3168030 | 481 | 235 | 186 | 166 | 535 | 312 | 785 | 140 | 390 | 344 | hsa-miR-144:9.1 | 269 |  |
| ILMN_3167456 | 22533 | 23705 | 24590 | 23887 | 193 | 1328 | 198 | 312 | 143 | 588 | hsa-miR-145 | 270 |  |
| ILMN_3168757 | 4085 | 6329 | 6950 | 5773 | 146 | 102 | 143 | 168 | 132 | 129 | hsa-miR-145* | 3136 |  |
| ILMN_3168434 | 10402 | 783 | 679 | 411 | 2758 | 1399 | 5772 | 12023 | 4668 | 1047 | hsa-miR-1468 | 2404 |  |
| ILMN_3168483 | 7835 | 16623 | 10203 | 13742 | 74 | 6172 | 69 | 90 | 59 | 7530 | hsa-miR-146a | 273 |  |
| ILMN_3168687 | 108 | 138 | 119 | 124 | 77 | 73 | 67 | 92 | 58 | 53 | hsa-miR-146a* | 3299 |  |
| ILMN_3168841 | 196 | 327 | 252 | 177 | 83 | 83 | 82 | 101 | 67 | 74 | hsa-miR-146b-3p | 3972 |  |
| ILMN_3167894 | 3292 | 12670 | 7439 | 7766 | 78 | 4227 | 74 | 106 | 65 | 4242 | hsa-miR-146b-5p | 285 |  |
| ILMN_3167920 | 369 | 85 | 56 | 66 | 118 | 115 | 119 | 227 | 3907 | 53 | hsa-miR-147 | 296 |  |
| ILMN_3168776 | 1911 | 2093 | 1559 | 1637 | 124 | 116 | 158 | 378 | 149 | 115 | hsa-miR-147b | 5968 |  |
| ILMN_3168105 | 5919 | 21779 | 20531 | 20541 | 81 | 83 | 81 | 112 | 67 | 77 | hsa-miR-148a | 300 |  |
| ILMN_3168549 | 99 | 461 | 84 | 95 | 70 | 74 | 67 | 87 | 60 | 52 | hsa-miR-148a* | 5397 |  |
| ILMN_3168426 | 3726 | 11547 | 10863 | 10993 | 76 | 749 | 73 | 101 | 61 | 419 | hsa-miR-148b | 312 |  |
| ILMN_3168567 | 280 | 598 | 310 | 279 | 80 | 77 | 73 | 94 | 62 | 58 | hsa-miR-148b* | 6123 |  |
| ILMN_3167902 | 665 | 2893 | 2208 | 2334 | 69 | 68 | 67 | 610 | 58 | 45 | hsa-miR-149 | 315 |  |
| ILMN_3168611 | 434 | 200 | 667 | 163 | 82 | 78 | 73 | 99 | 63 | 64 | hsa-miR-149* | 4223 |  |
| ILMN_3167703 | 23469 | 25732 | 26980 | 26093 | 4934 | 11362 | 72 | 101 | 62 | 10978 | hsa-miR-150 | 331 |  |
| ILMN_3168730 | 4548 | 2522 | 5037 | 4557 | 137 | 118 | 128 | 1030 | 1264 | 122 | hsa-miR-150* | 4227 |  |
| ILMN_3167062 | 3428 | 6053 | 6913 | 5481 | 79 | 84 | 78 | 125 | 66 | 6436 | hsa-miR-151:9.1 | 341 |  |
| ILMN_3168705 | 4668 | 14765 | 13490 | 9211 | 87 | 7251 | 81 | 99 | 70 | 9964 | hsa-miR-151-3p | 5784 |  |
| ILMN_3168819 | 13228 | 23740 | 22793 | 24596 | 72 | 7763 | 69 | 92 | 59 | 9151 | hsa-miR-151-5p | 4872 |  |
| ILMN_3168346 | 10908 | 22481 | 24082 | 22341 | 71 | 75 | 65 | 86 | 57 | 46 | hsa-miR-152 | 361 |  |
| ILMN_3166999 | 117 | 238 | 445 | 113 | 75 | 75 | 72 | 324 | 60 | 55 | hsa-miR-153 | 368 |  |
| ILMN_3167659 | 359 | 185 | 161 | 198 | 98 | 83 | 90 | 179 | 75 | 67 | hsa-miR-1537 | 2247 |  |
| ILMN_3167522 | 2156 | 8534 | 5971 | 5297 | 74 | 99 | 70 | 1259 | 5202 | 46 | hsa-miR-154 | 377 |  |
| ILMN_3167720 | 107 | 283 | 115 | 295 | 72 | 75 | 71 | 91 | 61 | 49 | hsa-miR-154* | 398 |  |
| ILMN_3168170 | 2685 | 6755 | 7371 | 6823 | 117 | 129 | 108 | 140 | 91 | 90 | hsa-miR-155 | 401 |  |
| ILMN_3168715 | 71 | 85 | 84 | 100 | 72 | 74 | 70 | 92 | 58 | 49 | hsa-miR-155* | 3712 |  |
| ILMN_3167434 | 7339 | 9788 | 9203 | 5616 | 79 | 1715 | 73 | 99 | 67 | 6376 | hsa-miR-15a | 403 |  |
| ILMN_3168662 | 11102 | 545 | 999 | 3418 | 304 | 4271 | 4699 | 10106 | 3139 | 43 | hsa-miR-15a* | 3534 |  |
| ILMN_3167060 | 20748 | 16803 | 18986 | 14723 | 2180 | 4642 | 68 | 468 | 60 | 8905 | hsa-miR-15b | 427 |  |
| ILMN_3168693 | 2324 | 4072 | 3754 | 2001 | 89 | 2544 | 80 | 104 | 71 | 4053 | hsa-miR-15b* | 4198 |  |
| ILMN_3167989 | 19727 | 20119 | 19996 | 15788 | 4295 | 7731 | 67 | 86 | 56 | 7848 | hsa-miR-16 | 430 |  |
| ILMN_3168676 | 418 | 930 | 213 | 94 | 69 | 78 | 62 | 86 | 55 | 43 | hsa-miR-16-1* | 6016 |  |
| ILMN_3168672 | 3000 | 3102 | 3481 | 1813 | 68 | 74 | 67 | 92 | 57 | 48 | hsa-miR-16-2* | 4225 |  |
| ILMN_3168642 | 5665 | 13213 | 8553 | 10358 | 66 | 2855 | 65 | 85 | 56 | 3840 | hsa-miR-17 | 3126 |  |
| ILMN_3167870 | 306 | 3482 | 2588 | 2607 | 67 | 67 | 66 | 86 | 56 | 44 | hsa-miR-17* | 433 |  |
| ILMN_3167426 | 243 | 136 | 51 | 70 | 69 | 69 | 89 | 459 | 56 | 44 | hsa-miR-17-5p:9.1 | 437 |  |
| ILMN_3167127 | 13205 | 13295 | 12045 | 8121 | 107 | 98 | 104 | 158 | 79 | 688 | hsa-miR-181a | 442 |  |
| ILMN_3168361 | 1068 | 1444 | 844 | 780 | 101 | 94 | 92 | 128 | 71 | 85 | hsa-miR-181a* | 740 |  |
| ILMN_3168577 | 1547 | 3350 | 4118 | 3143 | 97 | 86 | 96 | 130 | 80 | 610 | hsa-miR-181a-2* | 4092 |  |
| ILMN_3168257 | 9305 | 7884 | 7237 | 5040 | 86 | 866 | 81 | 210 | 71 | 2759 | hsa-miR-181b | 453 |  |
| ILMN_3168280 | 2593 | 1010 | 208 | 1554 | 78 | 83 | 75 | 101 | 61 | 70 | hsa-miR-181c | 460 |  |
| ILMN_3168555 | 520 | 846 | 1721 | 832 | 78 | 80 | 73 | 96 | 64 | 107 | hsa-miR-181c* | 3002 |  |
| ILMN_3167690 | 145 | 124 | 117 | 94 | 75 | 75 | 75 | 115 | 64 | 52 | hsa-miR-181d | 480 |  |
| ILMN_3167755 | 2642 | 6576 | 6835 | 3799 | 102 | 165 | 107 | 97 | 78 | 916 | hsa-miR-182 | 482 |  |
| ILMN_3166957 | 660 | 636 | 914 | 459 | 332 | 258 | 396 | 274 | 254 | 181 | hsa-miR-182* | 509 |  |
| ILMN_3168812 | 318 | 421 | 300 | 327 | 79 | 75 | 71 | 96 | 61 | 60 | hsa-miR-1825 | 3518 |  |
| ILMN_3168637 | 8693 | 7724 | 7960 | 13166 | 99 | 462 | 2030 | 148 | 73 | 98 | hsa-miR-1826 | 6062 |  |
| ILMN_3168834 | 97 | 133 | 83 | 92 | 71 | 72 | 68 | 90 | 60 | 47 | hsa-miR-1827 | 4823 |  |
| ILMN_3167529 | 2714 | 3041 | 2883 | 1928 | 69 | 68 | 64 | 86 | 56 | 42 | hsa-miR-183 | 515 |  |
| ILMN_3167593 | 173 | 156 | 330 | 110 | 72 | 70 | 67 | 91 | 59 | 51 | hsa-miR-183* | 5468 |  |
| ILMN_3167698 | 48 | 69 | 63 | 49 | 66 | 62 | 63 | 83 | 55 | 41 | hsa-miR-184 | 542 |  |
| ILMN_3167152 | 3760 | 6617 | 7423 | 6636 | 217 | 1130 | 74 | 250 | 63 | 148 | hsa-miR-185 | 559 |  |
| ILMN_3168613 | 453 | 282 | 712 | 278 | 73 | 69 | 67 | 92 | 59 | 50 | hsa-miR-185* | 5573 |  |
| ILMN_3168073 | 907 | 1308 | 644 | 1654 | 113 | 107 | 104 | 139 | 79 | 90 | hsa-miR-186 | 581 |  |
| ILMN_3168751 | 449 | 470 | 424 | 381 | 141 | 132 | 137 | 168 | 104 | 91 | hsa-miR-186* | 5725 |  |
| ILMN_3168167 | 257 | 3905 | 2129 | 2587 | 75 | 74 | 72 | 94 | 60 | 46 | hsa-miR-187 | 585 |  |
| ILMN_3168758 | 432 | 407 | 816 | 898 | 7147 | 5002 | 7068 | 2474 | 5422 | 3520 | hsa-miR-187* | 4178 |  |
| ILMN_3168714 | 248 | 248 | 244 | 212 | 79 | 78 | 71 | 93 | 65 | 63 | hsa-miR-188-3p | 5252 |  |
| ILMN_3167745 | 720 | 670 | 848 | 751 | 82 | 103 | 85 | 103 | 66 | 54 | hsa-miR-188-5p | 587 |  |
| ILMN_3168103 | 2612 | 2438 | 3143 | 3005 | 5185 | 3710 | 5503 | 1964 | 4161 | 3077 | hsa-miR-189:9.1 | 594 |  |
| ILMN_3168282 | 428 | 1032 | 675 | 686 | 68 | 66 | 66 | 85 | 56 | 45 | hsa-miR-18a | 601 |  |
| ILMN_3167178 | 4052 | 2643 | 2601 | 704 | 269 | 185 | 332 | 5969 | 280 | 164 | hsa-miR-18a* | 605 |  |
| ILMN_3167948 | 147 | 339 | 98 | 110 | 75 | 73 | 71 | 94 | 62 | 49 | hsa-miR-18b | 606 |  |
| ILMN_3168840 | 18388 | 1973 | 3839 | 5722 | 8118 | 6058 | 6767 | 14342 | 7643 | 5403 | hsa-miR-18b* | 3290 |  |
| ILMN_3167167 | 400 | 599 | 524 | 506 | 107 | 104 | 100 | 147 | 83 | 95 | hsa-miR-190 | 607 |  |
| ILMN_3168837 | 412 | 2071 | 2715 | 1561 | 72 | 73 | 72 | 96 | 61 | 49 | hsa-miR-190b | 4259 |  |
| ILMN_3167253 | 15087 | 15534 | 13372 | 18674 | 78 | 5819 | 74 | 92 | 62 | 6007 | hsa-miR-191 | 609 |  |
| ILMN_3167124 | 182 | 322 | 203 | 593 | 74 | 73 | 68 | 121 | 61 | 51 | hsa-miR-191* | 614 |  |
| ILMN_3167441 | 563 | 3523 | 1184 | 1279 | 75 | 70 | 71 | 478 | 59 | 4645 | hsa-miR-192 | 619 |  |
| ILMN_3168722 | 133 | 120 | 106 | 142 | 86 | 73 | 72 | 99 | 60 | 59 | hsa-miR-192* | 5070 |  |
| ILMN_3168366 | 4021 | 3395 | 3637 | 1639 | 99 | 108 | 137 | 132 | 79 | 85 | hsa-miR-193a-3p | 620 |  |
| ILMN_3168856 | 21864 | 20714 | 24385 | 16114 | 433 | 103 | 111 | 159 | 87 | 955 | hsa-miR-193a-5p | 5669 |  |
| ILMN_3167944 | 26580 | 10776 | 22077 | 5953 | 70 | 69 | 67 | 87 | 58 | 45 | hsa-miR-193b | 623 |  |
| ILMN_3168699 | 4504 | 1997 | 4810 | 484 | 77 | 73 | 71 | 95 | 62 | 54 | hsa-miR-193b* | 3141 |  |
| ILMN_3167122 | 1683 | 6233 | 3964 | 3916 | 97 | 86 | 89 | 5849 | 102 | 53 | hsa-miR-194 | 629 |  |
| ILMN_3168677 | 1435 | 1223 | 1260 | 994 | 152 | 147 | 156 | 4909 | 188 | 182 | hsa-miR-194* | 4091 |  |
| ILMN_3167191 | 16861 | 24687 | 24581 | 25790 | 107 | 1221 | 104 | 139 | 83 | 110 | hsa-miR-195 | 630 |  |
| ILMN_3168673 | 1311 | 3127 | 2857 | 3235 | 72 | 70 | 68 | 88 | 3864 | 53 | hsa-miR-195* | 3606 |  |
| ILMN_3167628 | 13099 | 18835 | 19401 | 20436 | 84 | 78 | 76 | 98 | 68 | 56 | hsa-miR-196a | 631 |  |
| ILMN_3168696 | 127 | 58 | 59 | 101 | 69 | 66 | 64 | 86 | 56 | 42 | hsa-miR-196a* | 4161 |  |
| ILMN_3168308 | 2280 | 4958 | 4905 | 5252 | 91 | 87 | 85 | 122 | 72 | 84 | hsa-miR-196b | 634 |  |
| ILMN_3167864 | 8341 | 8607 | 9573 | 13625 | 73 | 72 | 69 | 92 | 58 | 7535 | hsa-miR-197 | 636 |  |
| ILMN_3167418 | 760 | 364 | 1242 | 561 | 77 | 74 | 73 | 97 | 64 | 52 | hsa-miR-198 | 648 |  |
| ILMN_3168478 | 12355 | 24710 | 22623 | 16236 | 6688 | 5790 | 4780 | 10296 | 4708 | 5546 | hsa-miR-199a*:9.1 | 651 |  |
| ILMN_3168576 | 20507 | 23599 | 24227 | 22181 | 3884 | 2354 | 2900 | 9708 | 4954 | 7853 | hsa-miR-199a-3p,hsa-miR-199b-3p | 3888 |  |
| ILMN_3167976 | 7492 | 24935 | 23743 | 24045 | 68 | 3855 | 67 | 86 | 57 | 44 | hsa-miR-199a-5p | 649 |  |
| ILMN_3167259 | 4151 | 16787 | 11882 | 10815 | 86 | 79 | 81 | 118 | 89 | 56 | hsa-miR-199b-5p | 654 |  |
| ILMN_3167787 | 159 | 1120 | 903 | 2126 | 75 | 601 | 72 | 95 | 62 | 47 | hsa-miR-19a | 656 |  |
| ILMN_3168624 | 145 | 98 | 82 | 70 | 70 | 68 | 67 | 105 | 58 | 1672 | hsa-miR-19a* | 3979 |  |
| ILMN_3167260 | 2082 | 4577 | 1503 | 5196 | 105 | 2236 | 7066 | 142 | 85 | 78 | hsa-miR-19b | 657 |  |
| ILMN_3168622 | 157 | 162 | 120 | 179 | 69 | 69 | 68 | 187 | 61 | 50 | hsa-miR-19b-1* | 5078 |  |
| ILMN_3168623 | 620 | 392 | 443 | 539 | 100 | 87 | 90 | 150 | 7791 | 63 | hsa-miR-19b-2* | 3625 |  |
| ILMN_3167801 | 804 | 14650 | 9592 | 9976 | 74 | 69 | 69 | 123 | 60 | 48 | hsa-miR-200a | 671 |  |
| ILMN_3167179 | 350 | 2935 | 658 | 3598 | 1198 | 114 | 136 | 199 | 121 | 120 | hsa-miR-200a* | 674 |  |
| ILMN_3168294 | 3403 | 17158 | 8874 | 7948 | 102 | 96 | 97 | 354 | 81 | 98 | hsa-miR-200b | 678 |  |
| ILMN_3168668 | 1375 | 8704 | 7293 | 10847 | 79 | 80 | 73 | 113 | 66 | 59 | hsa-miR-200b* | 3881 |  |
| ILMN_3167002 | 18922 | 29313 | 30257 | 28945 | 121 | 3014 | 124 | 5764 | 783 | 88 | hsa-miR-200c | 682 |  |
| ILMN_3168701 | 120 | 243 | 169 | 91 | 74 | 69 | 68 | 92 | 61 | 48 | hsa-miR-200c* | 3072 |  |
| ILMN_3166953 | 140 | 447 | 124 | 124 | 79 | 74 | 74 | 109 | 61 | 53 | hsa-miR-202 | 691 |  |
| ILMN_3168871 | 375 | 294 | 337 | 293 | 82 | 89 | 80 | 105 | 66 | 86 | hsa-miR-202* | 4238 |  |
| ILMN_3167129 | 622 | 476 | 2498 | 1850 | 4162 | 5649 | 86 | 7033 | 9605 | 2863 | hsa-miR-202*:9.1 | 692 |  |
| ILMN_3167375 | 300 | 6141 | 3796 | 4131 | 65 | 66 | 62 | 86 | 56 | 42 | hsa-miR-203 | 696 |  |
| ILMN_3167196 | 839 | 1121 | 1151 | 938 | 442 | 281 | 512 | 275 | 364 | 386 | hsa-miR-204 | 697 |  |
| ILMN_3167091 | 10632 | 23545 | 22270 | 22724 | 93 | 82 | 4642 | 122 | 4477 | 60 | hsa-miR-205 | 700 |  |
| ILMN_3168019 | 2229 | 1834 | 4383 | 1121 | 83 | 80 | 79 | 121 | 67 | 57 | hsa-miR-206 | 715 |  |
| ILMN_3167135 | 61 | 71 | 57 | 73 | 78 | 74 | 72 | 97 | 82 | 47 | hsa-miR-208a | 717 |  |
| ILMN_3167105 | 429 | 183 | 142 | 148 | 92 | 87 | 88 | 134 | 71 | 64 | hsa-miR-208b | 2143 |  |
| ILMN_3167510 | 6041 | 21936 | 14978 | 17405 | 85 | 5360 | 89 | 2374 | 65 | 2805 | hsa-miR-20a | 718 |  |
| ILMN_3168590 | 943 | 1997 | 2089 | 1193 | 113 | 73 | 274 | 782 | 216 | 51 | hsa-miR-20a* | 4067 |  |
| ILMN_3167565 | 2510 | 2542 | 1886 | 236 | 89 | 81 | 77 | 4355 | 71 | 91 | hsa-miR-20b | 720 |  |
| ILMN_3168593 | 149 | 205 | 245 | 202 | 74 | 74 | 66 | 94 | 59 | 48 | hsa-miR-20b* | 2893 |  |
| ILMN_3167371 | 26683 | 30575 | 31574 | 28523 | 4983 | 21268 | 8311 | 3583 | 70 | 19491 | hsa-miR-21 | 721 |  |
| ILMN_3168646 | 696 | 402 | 185 | 251 | 73 | 75 | 69 | 115 | 62 | 59 | hsa-miR-21* | 4826 |  |
| ILMN_3167774 | 1071 | 3921 | 3905 | 3694 | 74 | 70 | 71 | 90 | 60 | 49 | hsa-miR-210 | 724 |  |
| ILMN_3167363 | 134 | 124 | 114 | 129 | 80 | 92 | 81 | 97 | 61 | 64 | hsa-miR-211 | 731 |  |
| ILMN_3167761 | 1274 | 3103 | 2856 | 356 | 75 | 75 | 71 | 94 | 61 | 49 | hsa-miR-212 | 732 |  |
| ILMN_3167046 | 25047 | 25289 | 27889 | 27381 | 72 | 72 | 72 | 94 | 59 | 53 | hsa-miR-214 | 742 |  |
| ILMN_3168845 | 1128 | 5091 | 6233 | 4685 | 86 | 78 | 75 | 98 | 69 | 55 | hsa-miR-214* | 5082 |  |
| ILMN_3167731 | 951 | 2411 | 3448 | 317 | 76 | 111 | 76 | 104 | 64 | 55 | hsa-miR-215 | 743 |  |
| ILMN_3167355 | 363 | 161 | 169 | 143 | 74 | 74 | 2163 | 100 | 59 | 1472 | hsa-miR-216a | 744 |  |
| ILMN_3168550 | 1580 | 1623 | 1536 | 2028 | 100 | 102 | 92 | 118 | 79 | 79 | hsa-miR-216b | 5910 |  |
| ILMN_3167478 | 121 | 103 | 94 | 100 | 74 | 74 | 69 | 93 | 60 | 47 | hsa-miR-217 | 745 |  |
| ILMN_3168380 | 1771 | 10054 | 10284 | 3136 | 3970 | 68 | 67 | 90 | 59 | 47 | hsa-miR-218 | 747 |  |
| ILMN_3168634 | 1557 | 1665 | 1500 | 1479 | 178 | 157 | 181 | 354 | 150 | 140 | hsa-miR-218-1* | 3752 |  |
| ILMN_3168670 | 132 | 148 | 127 | 125 | 70 | 67 | 65 | 86 | 57 | 52 | hsa-miR-218-2* | 4136 |  |
| ILMN_3168602 | 649 | 105 | 93 | 93 | 72 | 72 | 2679 | 212 | 60 | 50 | hsa-miR-219-1-3p | 5716 |  |
| ILMN_3168597 | 251 | 289 | 243 | 323 | 72 | 74 | 69 | 90 | 61 | 52 | hsa-miR-219-2-3p | 3363 |  |
| ILMN_3167523 | 96 | 1175 | 838 | 87 | 71 | 69 | 68 | 90 | 60 | 46 | hsa-miR-219-5p | 751 |  |
| ILMN_3167773 | 4137 | 7173 | 10311 | 3660 | 70 | 66 | 70 | 92 | 58 | 2463 | hsa-miR-22 | 752 |  |
| ILMN_3168621 | 5962 | 4469 | 5799 | 1079 | 72 | 80 | 71 | 1134 | 60 | 59 | hsa-miR-22* | 5449 |  |
| ILMN_3167666 | 315 | 224 | 447 | 1541 | 79 | 76 | 76 | 98 | 68 | 5220 | hsa-miR-220a | 753 |  |
| ILMN_3168674 | 2623 | 517 | 395 | 363 | 88 | 85 | 170 | 3479 | 2065 | 61 | hsa-miR-220b | 2182 |  |
| ILMN_3168572 | 358 | 180 | 189 | 177 | 75 | 76 | 69 | 92 | 61 | 50 | hsa-miR-220c | 5510 |  |
| ILMN_3167681 | 26199 | 25178 | 20552 | 20575 | 12843 | 4076 | 12367 | 20593 | 10162 | 8841 | hsa-miR-221 | 754 |  |
| ILMN_3168580 | 267 | 2198 | 1435 | 1753 | 74 | 70 | 72 | 90 | 61 | 3205 | hsa-miR-221* | 3541 |  |
| ILMN_3167963 | 7078 | 17967 | 17851 | 15965 | 78 | 77 | 2851 | 1007 | 2729 | 63 | hsa-miR-222 | 756 |  |
| ILMN_3168712 | 275 | 362 | 310 | 302 | 86 | 90 | 76 | 103 | 67 | 93 | hsa-miR-222* | 3450 |  |
| ILMN_3166979 | 5245 | 12245 | 10158 | 10489 | 8068 | 13301 | 2101 | 675 | 63 | 10992 | hsa-miR-223 | 758 |  |
| ILMN_3168703 | 504 | 450 | 449 | 129 | 79 | 76 | 79 | 119 | 63 | 504 | hsa-miR-223* | 5653 |  |
| ILMN_3168515 | 20506 | 16177 | 24749 | 5385 | 65 | 63 | 67 | 83 | 50 | 4806 | hsa-miR-224 | 759 |  |
| ILMN_3168226 | 26054 | 24224 | 25714 | 22101 | 121 | 9756 | 149 | 260 | 102 | 10908 | hsa-miR-23a | 764 |  |
| ILMN_3168762 | 3387 | 1810 | 5700 | 695 | 89 | 87 | 82 | 115 | 75 | 84 | hsa-miR-23a* | 3990 |  |
| ILMN_3167997 | 21723 | 15734 | 19932 | 14319 | 73 | 5137 | 65 | 90 | 58 | 5633 | hsa-miR-23b | 775 |  |
| ILMN_3168858 | 1227 | 634 | 695 | 470 | 84 | 80 | 79 | 101 | 70 | 63 | hsa-miR-23b* | 5911 |  |
| ILMN_3168211 | 20486 | 20347 | 22203 | 20967 | 828 | 5837 | 90 | 138 | 78 | 6360 | hsa-miR-24 | 778 |  |
| ILMN_3168844 | 448 | 1284 | 1732 | 984 | 79 | 76 | 81 | 109 | 64 | 52 | hsa-miR-24-1* | 5686 |  |
| ILMN_3168843 | 13955 | 3783 | 4279 | 3774 | 1046 | 999 | 1778 | 7453 | 1067 | 1738 | hsa-miR-24-2* | 3894 |  |
| ILMN_3168476 | 24296 | 25725 | 25973 | 24942 | 7884 | 11915 | 98 | 6357 | 4468 | 16008 | hsa-miR-25 | 780 |  |
| ILMN_3168609 | 1466 | 1722 | 1222 | 563 | 88 | 87 | 103 | 1289 | 69 | 78 | hsa-miR-25* | 4206 |  |
| ILMN_3168005 | 24292 | 24921 | 24622 | 24638 | 101 | 9249 | 77 | 143 | 81 | 9105 | hsa-miR-26a | 781 |  |
| ILMN_3168684 | 57 | 93 | 53 | 63 | 71 | 67 | 66 | 87 | 57 | 241 | hsa-miR-26a-1* | 5618 |  |
| ILMN_3168683 | 106 | 515 | 644 | 94 | 87 | 82 | 80 | 109 | 67 | 47 | hsa-miR-26a-2* | 4255 |  |
| ILMN_3167374 | 9631 | 23034 | 23044 | 25238 | 4929 | 6110 | 67 | 89 | 58 | 3991 | hsa-miR-26b | 791 |  |
| ILMN_3168691 | 1916 | 5936 | 6274 | 5018 | 72 | 72 | 69 | 952 | 63 | 6278 | hsa-miR-26b* | 5884 |  |
| ILMN_3168323 | 16299 | 26305 | 26064 | 27021 | 74 | 2630 | 68 | 94 | 59 | 47 | hsa-miR-27a | 799 |  |
| ILMN_3168612 | 1894 | 2823 | 2800 | 1949 | 165 | 106 | 88 | 137 | 76 | 66 | hsa-miR-27a* | 4990 |  |
| ILMN_3168409 | 6966 | 21332 | 20744 | 21920 | 94 | 86 | 95 | 133 | 76 | 100 | hsa-miR-27b | 800 |  |
| ILMN_3168599 | 1247 | 1140 | 600 | 1659 | 117 | 109 | 108 | 140 | 88 | 83 | hsa-miR-27b* | 5625 |  |
| ILMN_3168657 | 5996 | 7016 | 6611 | 5757 | 81 | 3707 | 78 | 4039 | 66 | 62 | hsa-miR-28-3p | 5133 |  |
| ILMN_3167223 | 7440 | 18987 | 16463 | 20041 | 74 | 74 | 73 | 94 | 68 | 76 | hsa-miR-28-5p | 805 |  |
| ILMN_3168049 | 433 | 1559 | 1410 | 104 | 92 | 75 | 88 | 101 | 80 | 70 | hsa-miR-296-3p | 2436 |  |
| ILMN_3167226 | 246 | 366 | 384 | 662 | 80 | 77 | 77 | 100 | 66 | 51 | hsa-miR-296-5p | 807 |  |
| ILMN_3168636 | 1076 | 775 | 723 | 691 | 99 | 91 | 96 | 133 | 79 | 68 | hsa-miR-297 | 5565 |  |
| ILMN_3168603 | 1351 | 1618 | 1559 | 1499 | 110 | 103 | 99 | 124 | 87 | 88 | hsa-miR-298 | 3497 |  |
| ILMN_3168177 | 592 | 387 | 526 | 285 | 72 | 76 | 66 | 90 | 57 | 53 | hsa-miR-299-3p | 813 |  |
| ILMN_3167913 | 830 | 722 | 267 | 1062 | 71 | 70 | 68 | 109 | 59 | 1849 | hsa-miR-299-5p | 814 |  |
| ILMN_3167035 | 4873 | 17133 | 14069 | 14370 | 77 | 83 | 74 | 91 | 60 | 55 | hsa-miR-29a | 815 |  |
| ILMN_3168589 | 456 | 4957 | 1605 | 3749 | 74 | 71 | 70 | 92 | 56 | 49 | hsa-miR-29a* | 5537 |  |
| ILMN_3168172 | 7045 | 21316 | 19579 | 14838 | 1932 | 5331 | 68 | 133 | 60 | 5070 | hsa-miR-29b | 820 |  |
| ILMN_3168755 | 6796 | 5227 | 6075 | 2881 | 82 | 82 | 75 | 97 | 65 | 150 | hsa-miR-29b-1* | 4301 |  |
| ILMN_3168731 | 2544 | 1127 | 862 | 1065 | 71 | 74 | 73 | 100 | 64 | 48 | hsa-miR-29b-2* | 3171 |  |
| ILMN_3167643 | 1787 | 8790 | 7545 | 5752 | 96 | 84 | 85 | 143 | 83 | 56 | hsa-miR-29c | 822 |  |
| ILMN_3168830 | 6942 | 8905 | 7483 | 6492 | 83 | 103 | 77 | 102 | 65 | 82 | hsa-miR-29c* | 3495 |  |
| ILMN_3168796 | 292 | 356 | 265 | 185 | 90 | 84 | 78 | 105 | 87 | 157 | hsa-miR-300 | 6140 |  |
| ILMN_3168116 | 1524 | 1793 | 1488 | 1199 | 122 | 121 | 2022 | 173 | 473 | 170 | hsa-miR-301a | 839 |  |
| ILMN_3168665 | 89 | 50 | 39 | 60 | 67 | 67 | 64 | 125 | 56 | 44 | hsa-miR-301b | 4939 |  |
| ILMN_3167116 | 80 | 109 | 72 | 157 | 72 | 69 | 68 | 89 | 58 | 50 | hsa-miR-302a | 840 |  |
| ILMN_3167474 | 170 | 168 | 201 | 146 | 70 | 74 | 69 | 92 | 56 | 52 | hsa-miR-302a* | 844 |  |
| ILMN_3167208 | 216 | 324 | 68 | 77 | 71 | 73 | 68 | 90 | 58 | 49 | hsa-miR-302b | 859 |  |
| ILMN_3168322 | 20280 | 2162 | 4569 | 9981 | 8651 | 6251 | 7907 | 13713 | 8331 | 6888 | hsa-miR-302b* | 861 |  |
| ILMN_3166944 | 182 | 91 | 83 | 79 | 76 | 79 | 72 | 95 | 63 | 58 | hsa-miR-302c | 864 |  |
| ILMN_3167269 | 310 | 161 | 162 | 179 | 91 | 92 | 89 | 118 | 71 | 79 | hsa-miR-302c* | 881 |  |
| ILMN_3167386 | 27367 | 1932 | 4686 | 7368 | 13045 | 10042 | 12049 | 18698 | 9889 | 9329 | hsa-miR-302d | 884 |  |
| ILMN_3168595 | 204 | 153 | 348 | 129 | 75 | 71 | 73 | 100 | 61 | 68 | hsa-miR-302d* | 3923 |  |
| ILMN_3168782 | 108 | 59 | 437 | 64 | 73 | 72 | 70 | 93 | 62 | 45 | hsa-miR-302e | 4261 |  |
| ILMN_3168784 | 50 | 47 | 40 | 52 | 68 | 65 | 65 | 87 | 56 | 41 | hsa-miR-302f | 4850 |  |
| ILMN_3167455 | 6792 | 16870 | 8896 | 7882 | 65 | 66 | 63 | 85 | 55 | 44 | hsa-miR-30a | 887 |  |
| ILMN_3167158 | 4079 | 10496 | 9298 | 7763 | 70 | 70 | 68 | 91 | 60 | 45 | hsa-miR-30a* | 885 |  |
| ILMN_3167448 | 6178 | 19420 | 18516 | 18942 | 72 | 4695 | 66 | 83 | 57 | 71 | hsa-miR-30b | 889 |  |
| ILMN_3168729 | 549 | 264 | 282 | 176 | 78 | 74 | 74 | 90 | 67 | 63 | hsa-miR-30b* | 5801 |  |
| ILMN_3167729 | 20299 | 25247 | 26381 | 24249 | 384 | 3425 | 419 | 262 | 327 | 356 | hsa-miR-30c | 890 |  |
| ILMN_3168728 | 3601 | 2143 | 3447 | 2854 | 76 | 75 | 69 | 94 | 61 | 48 | hsa-miR-30c-1* | 4944 |  |
| ILMN_3168727 | 636 | 682 | 661 | 1037 | 77 | 79 | 76 | 90 | 59 | 71 | hsa-miR-30c-2* | 5342 |  |
| ILMN_3167224 | 23435 | 26828 | 28687 | 26501 | 2060 | 4384 | 74 | 97 | 64 | 4479 | hsa-miR-30d | 891 |  |
| ILMN_3168739 | 2258 | 2032 | 1645 | 1413 | 232 | 175 | 200 | 398 | 175 | 192 | hsa-miR-30d* | 5959 |  |
| ILMN_3168138 | 2857 | 5937 | 3042 | 4948 | 209 | 208 | 215 | 352 | 171 | 352 | hsa-miR-30e | 897 |  |
| ILMN_3167711 | 9291 | 24117 | 21385 | 20112 | 183 | 1411 | 206 | 308 | 141 | 5794 | hsa-miR-30e* | 895 |  |
| ILMN_3167837 | 1102 | 6763 | 3621 | 7586 | 75 | 84 | 6561 | 1421 | 62 | 101 | hsa-miR-31 | 901 |  |
| ILMN_3168847 | 330 | 5498 | 4353 | 4220 | 78 | 4405 | 70 | 94 | 1015 | 78 | hsa-miR-31* | 5879 |  |
| ILMN_3167472 | 61 | 179 | 230 | 61 | 73 | 69 | 68 | 90 | 59 | 46 | hsa-miR-32 | 905 |  |
| ILMN_3168655 | 57 | 124 | 49 | 102 | 68 | 65 | 65 | 86 | 58 | 41 | hsa-miR-32* | 3482 |  |
| ILMN_3167403 | 25601 | 25134 | 27086 | 25601 | 4316 | 11085 | 103 | 151 | 58 | 7266 | hsa-miR-320d,hsa-miR-320b,hsa-miR-320a,hsa-miR-320c | 913 |  |
| ILMN_3167956 | 327 | 1249 | 148 | 139 | 77 | 75 | 76 | 103 | 64 | 117 | hsa-miR-323-3p | 915 |  |
| ILMN_3168615 | 220 | 166 | 171 | 172 | 80 | 81 | 73 | 103 | 66 | 55 | hsa-miR-323-5p | 3930 |  |
| ILMN_3168413 | 5869 | 16748 | 14263 | 12750 | 81 | 86 | 81 | 205 | 70 | 68 | hsa-miR-324-3p | 917 |  |
| ILMN_3166969 | 11546 | 6495 | 14489 | 15868 | 18166 | 14884 | 18952 | 17557 | 16337 | 16052 | hsa-miR-324-5p | 920 |  |
| ILMN_3167669 | 156 | 152 | 158 | 153 | 75 | 80 | 71 | 95 | 61 | 62 | hsa-miR-325 | 925 |  |
| ILMN_3167905 | 4905 | 2944 | 3072 | 1066 | 72 | 67 | 68 | 109 | 55 | 47 | hsa-miR-326 | 926 |  |
| ILMN_3168198 | 6348 | 6361 | 8776 | 9026 | 64 | 62 | 62 | 92 | 53 | 42 | hsa-miR-328 | 929 |  |
| ILMN_3166935 | 682 | 4994 | 4488 | 700 | 82 | 78 | 70 | 93 | 59 | 56 | hsa-miR-329 | 931 |  |
| ILMN_3168208 | 750 | 1173 | 736 | 438 | 85 | 81 | 81 | 111 | 69 | 51 | hsa-miR-330-3p | 935 |  |
| ILMN_3168826 | 214 | 106 | 106 | 113 | 88 | 88 | 83 | 120 | 71 | 60 | hsa-miR-330-5p | 4066 |  |
| ILMN_3167611 | 6815 | 5769 | 5803 | 4851 | 83 | 79 | 68 | 97 | 66 | 53 | hsa-miR-331-3p | 936 |  |
| ILMN_3168706 | 274 | 242 | 219 | 172 | 73 | 74 | 71 | 95 | 60 | 47 | hsa-miR-331-5p | 3156 |  |
| ILMN_3167996 | 2225 | 1929 | 2590 | 599 | 73 | 82 | 74 | 110 | 60 | 127 | hsa-miR-335 | 940 |  |
| ILMN_3168885 | 8711 | 8578 | 8782 | 1066 | 75 | 82 | 5963 | 97 | 69 | 4957 | hsa-miR-335* | 5988 |  |
| ILMN_3168404 | 87 | 126 | 72 | 84 | 71 | 96 | 3325 | 958 | 159 | 302 | hsa-miR-337:9.1 | 942 |  |
| ILMN_3168716 | 717 | 4432 | 2820 | 480 | 72 | 76 | 69 | 86 | 58 | 48 | hsa-miR-337-3p | 4009 |  |
| ILMN_3168742 | 135 | 161 | 105 | 118 | 92 | 84 | 89 | 105 | 81 | 68 | hsa-miR-337-5p | 5221 |  |
| ILMN_3167344 | 401 | 964 | 395 | 1172 | 76 | 80 | 69 | 91 | 61 | 68 | hsa-miR-338-3p | 943 |  |
| ILMN_3168552 | 133 | 151 | 139 | 134 | 85 | 78 | 85 | 101 | 76 | 67 | hsa-miR-338-5p | 4005 |  |
| ILMN_3168833 | 287 | 566 | 80 | 457 | 66 | 66 | 63 | 85 | 54 | 43 | hsa-miR-339-3p | 3996 |  |
| ILMN_3168301 | 2911 | 2239 | 2555 | 2391 | 65 | 63 | 61 | 82 | 52 | 45 | hsa-miR-339-5p | 944 |  |
| ILMN_3167691 | 7216 | 495 | 1608 | 1591 | 2218 | 72 | 4859 | 5701 | 1516 | 46 | hsa-miR-33a | 933 |  |
| ILMN_3168654 | 773 | 208 | 248 | 173 | 81 | 84 | 85 | 142 | 59 | 62 | hsa-miR-33a* | 3305 |  |
| ILMN_3166988 | 66 | 152 | 145 | 70 | 75 | 73 | 70 | 90 | 61 | 46 | hsa-miR-33b | 1604 |  |
| ILMN_3168666 | 123 | 87 | 81 | 81 | 74 | 71 | 117 | 90 | 59 | 47 | hsa-miR-33b* | 4008 |  |
| ILMN_3168866 | 354 | 821 | 553 | 385 | 79 | 87 | 77 | 120 | 64 | 74 | hsa-miR-340 | 5973 |  |
| ILMN_3166998 | 1562 | 1099 | 1570 | 712 | 72 | 1463 | 68 | 94 | 60 | 679 | hsa-miR-340* | 946 |  |
| ILMN_3168165 | 6269 | 9990 | 7678 | 7518 | 84 | 2321 | 81 | 94 | 67 | 1772 | hsa-miR-342-3p | 949 |  |
| ILMN_3168614 | 4838 | 6532 | 6469 | 7607 | 84 | 7024 | 82 | 112 | 83 | 64 | hsa-miR-342-5p | 3725 |  |
| ILMN_3168754 | 293 | 248 | 116 | 80 | 73 | 70 | 71 | 90 | 61 | 46 | hsa-miR-345 | 3465 |  |
| ILMN_3167702 | 365 | 743 | 738 | 587 | 92 | 84 | 84 | 114 | 72 | 71 | hsa-miR-345:9.1 | 950 |  |
| ILMN_3168451 | 19098 | 808 | 2902 | 3713 | 8486 | 6839 | 7968 | 15321 | 7804 | 6734 | hsa-miR-346 | 951 |  |
| ILMN_3168429 | 3158 | 19301 | 11691 | 14625 | 76 | 74 | 74 | 95 | 62 | 65 | hsa-miR-34a | 952 |  |
| ILMN_3168653 | 454 | 988 | 640 | 652 | 84 | 81 | 83 | 150 | 214 | 65 | hsa-miR-34a* | 5960 |  |
| ILMN_3168652 | 157 | 873 | 303 | 1069 | 65 | 66 | 61 | 83 | 54 | 44 | hsa-miR-34b | 5930 |  |
| ILMN_3167325 | 278 | 417 | 709 | 217 | 102 | 67 | 68 | 93 | 162 | 85 | hsa-miR-34b* | 958 |  |
| ILMN_3168568 | 346 | 538 | 62 | 281 | 82 | 81 | 79 | 113 | 69 | 61 | hsa-miR-34c-3p | 5854 |  |
| ILMN_3167743 | 134 | 1213 | 422 | 351 | 77 | 75 | 74 | 96 | 66 | 50 | hsa-miR-34c-5p | 960 |  |
| ILMN_3168815 | 1409 | 2361 | 2146 | 984 | 81 | 108 | 76 | 100 | 67 | 96 | hsa-miR-361-3p | 3007 |  |
| ILMN_3168391 | 5169 | 5542 | 5470 | 6384 | 91 | 2106 | 82 | 108 | 74 | 63 | hsa-miR-361-5p | 961 |  |
| ILMN_3168553 | 1409 | 3677 | 2954 | 1661 | 202 | 818 | 185 | 247 | 150 | 1266 | hsa-miR-362-3p | 5941 |  |
| ILMN_3167366 | 1213 | 562 | 61 | 1062 | 85 | 76 | 82 | 97 | 69 | 51 | hsa-miR-362-5p | 963 |  |
| ILMN_3168344 | 3052 | 2194 | 1988 | 881 | 108 | 91 | 90 | 147 | 85 | 85 | hsa-miR-363 | 969 |  |
| ILMN_3167509 | 82 | 62 | 54 | 78 | 83 | 79 | 81 | 114 | 74 | 53 | hsa-miR-363* | 971 |  |
| ILMN_3168297 | 1776 | 754 | 1983 | 369 | 81 | 82 | 78 | 103 | 63 | 63 | hsa-miR-365 | 973 |  |
| ILMN_3167195 | 153 | 201 | 200 | 190 | 81 | 73 | 72 | 94 | 63 | 49 | hsa-miR-367 | 974 |  |
| ILMN_3168594 | 854 | 666 | 636 | 604 | 154 | 204 | 155 | 209 | 120 | 126 | hsa-miR-367* | 5576 |  |
| ILMN_3167458 | 113 | 276 | 733 | 163 | 5211 | 80 | 88 | 4410 | 74 | 71 | hsa-miR-369-3p | 977 |  |
| ILMN_3166955 | 636 | 1591 | 745 | 504 | 114 | 107 | 111 | 182 | 88 | 90 | hsa-miR-369-5p | 978 |  |
| ILMN_3167215 | 2197 | 5173 | 4122 | 525 | 95 | 88 | 87 | 109 | 75 | 70 | hsa-miR-370 | 979 |  |
| ILMN_3167164 | 270 | 569 | 420 | 245 | 76 | 92 | 70 | 97 | 62 | 115 | hsa-miR-371-3p | 984 |  |
| ILMN_3168586 | 803 | 1093 | 974 | 729 | 124 | 110 | 95 | 139 | 80 | 76 | hsa-miR-371-5p | 4016 |  |
| ILMN_3167184 | 864 | 593 | 554 | 480 | 155 | 135 | 128 | 254 | 109 | 175 | hsa-miR-372 | 987 |  |
| ILMN_3167463 | 61 | 50 | 56 | 63 | 69 | 64 | 61 | 87 | 57 | 42 | hsa-miR-373 | 989 |  |
| ILMN_3167267 | 76 | 65 | 236 | 64 | 66 | 67 | 65 | 87 | 56 | 48 | hsa-miR-373* | 991 |  |
| ILMN_3168240 | 2221 | 2933 | 3111 | 1511 | 225 | 209 | 314 | 730 | 237 | 173 | hsa-miR-374a | 992 |  |
| ILMN_3168735 | 624 | 906 | 830 | 679 | 90 | 86 | 82 | 105 | 68 | 93 | hsa-miR-374a* | 5935 |  |
| ILMN_3168625 | 152 | 763 | 55 | 57 | 68 | 66 | 62 | 83 | 55 | 41 | hsa-miR-374b | 5736 |  |
| ILMN_3168734 | 79 | 88 | 62 | 69 | 71 | 66 | 65 | 86 | 56 | 45 | hsa-miR-374b* | 3523 |  |
| ILMN_3167229 | 2440 | 16283 | 11299 | 19447 | 7390 | 73 | 72 | 4860 | 5583 | 7524 | hsa-miR-375 | 1000 |  |
| ILMN_3168111 | 807 | 832 | 947 | 282 | 6914 | 84 | 5035 | 2232 | 71 | 4745 | hsa-miR-376a | 1001 |  |
| ILMN_3168767 | 95 | 342 | 278 | 64 | 70 | 66 | 66 | 88 | 56 | 46 | hsa-miR-376a* | 5602 |  |
| ILMN_3167419 | 1188 | 378 | 763 | 1691 | 5522 | 5130 | 467 | 1603 | 65 | 4197 | hsa-miR-376a*:9.1 | 1002 |  |
| ILMN_3167915 | 732 | 1156 | 1417 | 1679 | 393 | 137 | 136 | 4057 | 138 | 1579 | hsa-miR-376b | 1006 |  |
| ILMN_3166941 | 477 | 3225 | 1063 | 2777 | 4608 | 1266 | 1719 | 3095 | 5463 | 1899 | hsa-miR-376c | 976 |  |
| ILMN_3168481 | 682 | 822 | 1241 | 623 | 3904 | 4429 | 139 | 466 | 95 | 134 | hsa-miR-377 | 1009 |  |
| ILMN_3168600 | 274 | 371 | 231 | 422 | 77 | 78 | 69 | 94 | 62 | 59 | hsa-miR-377* | 3315 |  |
| ILMN_3167006 | 8300 | 13321 | 14825 | 7584 | 74 | 1000 | 71 | 91 | 62 | 67 | hsa-miR-378 | 1046 |  |
| ILMN_3168180 | 5055 | 2577 | 3720 | 557 | 6107 | 85 | 115 | 136 | 74 | 5135 | hsa-miR-378* | 1011 |  |
| ILMN_3167443 | 883 | 2603 | 1770 | 714 | 373 | 6704 | 97 | 5906 | 6320 | 55 | hsa-miR-379 | 1015 |  |
| ILMN_3168800 | 76 | 161 | 867 | 83 | 74 | 72 | 66 | 92 | 60 | 56 | hsa-miR-379* | 4101 |  |
| ILMN_3167450 | 386 | 432 | 1113 | 2003 | 6384 | 4944 | 4352 | 3749 | 7110 | 1735 | hsa-miR-380 | 1020 |  |
| ILMN_3168015 | 339 | 374 | 431 | 297 | 1476 | 314 | 89 | 120 | 74 | 179 | hsa-miR-380* | 1022 |  |
| ILMN_3168477 | 530 | 3491 | 1898 | 1294 | 93 | 131 | 78 | 104 | 68 | 192 | hsa-miR-381 | 1024 |  |
| ILMN_3167239 | 694 | 3908 | 2554 | 654 | 68 | 68 | 67 | 88 | 57 | 46 | hsa-miR-382 | 1030 |  |
| ILMN_3166965 | 290 | 841 | 784 | 167 | 97 | 92 | 93 | 126 | 77 | 61 | hsa-miR-383 | 1032 |  |
| ILMN_3167271 | 255 | 162 | 149 | 159 | 75 | 71 | 73 | 97 | 62 | 54 | hsa-miR-384 | 1037 |  |
| ILMN_3167969 | 823 | 2696 | 2399 | 74 | 72 | 188 | 69 | 92 | 63 | 47 | hsa-miR-409-3p | 1039 |  |
| ILMN_3168347 | 72 | 1070 | 944 | 61 | 70 | 72 | 64 | 90 | 56 | 47 | hsa-miR-409-5p | 1040 |  |
| ILMN_3167244 | 108 | 1056 | 530 | 505 | 71 | 70 | 69 | 92 | 58 | 48 | hsa-miR-410 | 1041 |  |
| ILMN_3167988 | 1485 | 6231 | 4053 | 1810 | 72 | 70 | 67 | 97 | 58 | 44 | hsa-miR-411 | 1608 |  |
| ILMN_3168799 | 127 | 315 | 428 | 175 | 69 | 70 | 69 | 92 | 60 | 43 | hsa-miR-411* | 3695 |  |
| ILMN_3168473 | 358 | 328 | 359 | 270 | 76 | 82 | 70 | 96 | 61 | 52 | hsa-miR-412 | 1042 |  |
| ILMN_3167569 | 2677 | 4936 | 6020 | 3371 | 91 | 89 | 74 | 102 | 68 | 60 | hsa-miR-421 | 1610 |  |
| ILMN_3167914 | 77 | 57 | 52 | 72 | 68 | 66 | 63 | 81 | 56 | 44 | hsa-miR-422a | 1044 |  |
| ILMN_3167373 | 6655 | 10487 | 10969 | 9742 | 77 | 2246 | 70 | 3066 | 61 | 64 | hsa-miR-423-3p | 1047 |  |
| ILMN_3168835 | 27227 | 7096 | 9630 | 12600 | 15662 | 10274 | 10996 | 17914 | 12427 | 10174 | hsa-miR-423-5p | 6115 |  |
| ILMN_3166938 | 2954 | 18891 | 15616 | 9305 | 77 | 75 | 71 | 94 | 63 | 59 | hsa-miR-424 | 1051 |  |
| ILMN_3168638 | 1584 | 2133 | 2135 | 438 | 71 | 69 | 67 | 90 | 59 | 47 | hsa-miR-424* | 5688 |  |
| ILMN_3167721 | 4572 | 9907 | 10654 | 6605 | 70 | 5968 | 67 | 94 | 59 | 5817 | hsa-miR-425 | 1617 |  |
| ILMN_3168410 | 1341 | 3226 | 5664 | 1303 | 81 | 76 | 402 | 135 | 65 | 65 | hsa-miR-425* | 1055 |  |
| ILMN_3167806 | 707 | 2627 | 1990 | 83 | 439 | 81 | 3283 | 96 | 54 | 4810 | hsa-miR-429 | 1060 |  |
| ILMN_3167383 | 3660 | 1091 | 276 | 2231 | 71 | 74 | 70 | 9734 | 60 | 51 | hsa-miR-431 | 1063 |  |
| ILMN_3168664 | 546 | 96 | 93 | 92 | 74 | 75 | 67 | 98 | 59 | 49 | hsa-miR-431* | 1590 |  |
| ILMN_3167818 | 3164 | 9042 | 8319 | 6516 | 88 | 5231 | 4760 | 108 | 73 | 7466 | hsa-miR-432 | 1066 |  |
| ILMN_3167794 | 130 | 78 | 62 | 79 | 68 | 69 | 66 | 86 | 57 | 44 | hsa-miR-432* | 1071 |  |
| ILMN_3167973 | 673 | 3917 | 2979 | 1675 | 67 | 98 | 63 | 83 | 54 | 41 | hsa-miR-433 | 1072 |  |
| ILMN_3168228 | 76 | 121 | 94 | 60 | 69 | 69 | 66 | 85 | 54 | 46 | hsa-miR-448 | 1077 |  |
| ILMN_3167451 | 175 | 93 | 474 | 87 | 77 | 74 | 72 | 101 | 67 | 51 | hsa-miR-449a | 1623 |  |
| ILMN_3168441 | 1090 | 1150 | 1101 | 1025 | 194 | 194 | 181 | 234 | 149 | 241 | hsa-miR-449b | 1628 |  |
| ILMN_3168187 | 810 | 1588 | 1679 | 911 | 91 | 89 | 75 | 143 | 67 | 89 | hsa-miR-450a | 1079 |  |
| ILMN_3168879 | 94 | 56 | 199 | 66 | 75 | 73 | 72 | 99 | 63 | 47 | hsa-miR-450b-3p | 4064 |  |
| ILMN_3168884 | 454 | 760 | 520 | 351 | 75 | 94 | 83 | 141 | 73 | 75 | hsa-miR-450b-5p | 4072 |  |
| ILMN_3167614 | 26526 | 25477 | 26830 | 13710 | 2761 | 123 | 182 | 3800 | 165 | 4604 | hsa-miR-451 | 1080 |  |
| ILMN_3167050 | 1931 | 1291 | 1865 | 408 | 68 | 69 | 63 | 85 | 57 | 46 | hsa-miR-452 | 1085 |  |
| ILMN_3168713 | 338 | 699 | 469 | 776 | 87 | 338 | 74 | 96 | 64 | 141 | hsa-miR-452* | 5384 |  |
| ILMN_3167652 | 344 | 870 | 663 | 440 | 72 | 66 | 68 | 85 | 57 | 42 | hsa-miR-452*:9.1 | 1086 |  |
| ILMN_3168292 | 222 | 377 | 658 | 212 | 83 | 84 | 75 | 98 | 63 | 96 | hsa-miR-453 | 1087 |  |
| ILMN_3168319 | 495 | 935 | 638 | 276 | 79 | 81 | 75 | 99 | 67 | 90 | hsa-miR-454 | 2254 |  |
| ILMN_3168024 | 983 | 651 | 1404 | 201 | 71 | 68 | 64 | 3006 | 58 | 379 | hsa-miR-454* | 2003 |  |
| ILMN_3168749 | 4820 | 9928 | 9210 | 8675 | 85 | 80 | 75 | 959 | 61 | 65 | hsa-miR-455-3p | 2829 |  |
| ILMN_3167714 | 2373 | 1929 | 1340 | 1727 | 4058 | 82 | 79 | 521 | 73 | 78 | hsa-miR-455-5p | 1088 |  |
| ILMN_3168173 | 3511 | 1357 | 1405 | 3298 | 90 | 86 | 83 | 113 | 75 | 85 | hsa-miR-483-3p | 1093 |  |
| ILMN_3168558 | 265 | 256 | 265 | 436 | 77 | 72 | 72 | 412 | 62 | 56 | hsa-miR-483-5p | 3041 |  |
| ILMN_3168457 | 8441 | 12205 | 12797 | 7932 | 90 | 4097 | 94 | 2776 | 68 | 5441 | hsa-miR-484 | 1097 |  |
| ILMN_3168166 | 516 | 1157 | 1178 | 638 | 76 | 79 | 74 | 97 | 64 | 68 | hsa-miR-485-3p | 1098 |  |
| ILMN_3167221 | 689 | 796 | 629 | 896 | 83 | 77 | 78 | 111 | 66 | 59 | hsa-miR-485-5p | 1099 |  |
| ILMN_3168698 | 7374 | 2221 | 3953 | 3600 | 83 | 75 | 72 | 97 | 63 | 60 | hsa-miR-486-3p | 4260 |  |
| ILMN_3167240 | 23979 | 23125 | 26422 | 19225 | 4121 | 6322 | 1166 | 6889 | 7264 | 18081 | hsa-miR-486-5p | 1102 |  |
| ILMN_3166948 | 670 | 498 | 82 | 128 | 79 | 72 | 71 | 2060 | 65 | 51 | hsa-miR-487a | 1104 |  |
| ILMN_3167805 | 868 | 6125 | 5425 | 1560 | 76 | 72 | 70 | 95 | 60 | 56 | hsa-miR-487b | 1105 |  |
| ILMN_3168874 | 143 | 95 | 104 | 111 | 75 | 72 | 68 | 91 | 65 | 52 | hsa-miR-488 | 5629 |  |
| ILMN_3168337 | 1167 | 190 | 193 | 233 | 191 | 99 | 73 | 1099 | 62 | 59 | hsa-miR-488* | 1109 |  |
| ILMN_3167272 | 72 | 80 | 54 | 58 | 73 | 70 | 68 | 94 | 58 | 42 | hsa-miR-489 | 1110 |  |
| ILMN_3168283 | 283 | 179 | 189 | 168 | 81 | 78 | 73 | 94 | 60 | 52 | hsa-miR-490-3p | 1111 |  |
| ILMN_3167370 | 186 | 117 | 105 | 291 | 88 | 78 | 81 | 103 | 68 | 53 | hsa-miR-490-5p | 4075 |  |
| ILMN_3168736 | 363 | 712 | 429 | 391 | 83 | 83 | 76 | 101 | 67 | 68 | hsa-miR-491-3p | 3324 |  |
| ILMN_3167553 | 1712 | 2684 | 1494 | 4387 | 141 | 131 | 130 | 238 | 112 | 143 | hsa-miR-491-5p | 1124 |  |
| ILMN_3167532 | 235 | 82 | 1010 | 62 | 75 | 74 | 75 | 96 | 66 | 48 | hsa-miR-492 | 1125 |  |
| ILMN_3168178 | 13194 | 2536 | 4551 | 3134 | 76 | 128 | 968 | 9574 | 62 | 50 | hsa-miR-493 | 1138 |  |
| ILMN_3167972 | 1035 | 1027 | 923 | 483 | 86 | 83 | 78 | 184 | 67 | 66 | hsa-miR-493* | 1145 |  |
| ILMN_3168446 | 4754 | 1144 | 464 | 512 | 92 | 88 | 85 | 134 | 74 | 111 | hsa-miR-494 | 1151 |  |
| ILMN_3167052 | 306 | 1048 | 559 | 453 | 81 | 825 | 74 | 105 | 69 | 66 | hsa-miR-495 | 1164 |  |
| ILMN_3167393 | 168 | 114 | 82 | 97 | 74 | 71 | 71 | 97 | 63 | 52 | hsa-miR-496 | 1170 |  |
| ILMN_3167437 | 4661 | 7658 | 5322 | 7881 | 70 | 74 | 67 | 90 | 57 | 48 | hsa-miR-497 | 1171 |  |
| ILMN_3168640 | 63 | 50 | 53 | 63 | 63 | 62 | 63 | 78 | 52 | 43 | hsa-miR-497* | 5546 |  |
| ILMN_3167977 | 147 | 525 | 682 | 795 | 76 | 72 | 69 | 98 | 1027 | 53 | hsa-miR-498 | 1173 |  |
| ILMN_3168554 | 206 | 184 | 105 | 1689 | 7857 | 70 | 72 | 98 | 64 | 48 | hsa-miR-499-3p | 3478 |  |
| ILMN_3167642 | 2484 | 4282 | 4683 | 2860 | 219 | 245 | 247 | 456 | 155 | 269 | hsa-miR-499-5p | 1178 |  |
| ILMN_3168783 | 3104 | 6403 | 5235 | 4153 | 78 | 75 | 72 | 98 | 63 | 58 | hsa-miR-500 | 4268 |  |
| ILMN_3168570 | 390 | 1352 | 1579 | 665 | 66 | 64 | 66 | 82 | 55 | 43 | hsa-miR-501-3p | 4087 |  |
| ILMN_3168378 | 277 | 592 | 978 | 63 | 76 | 75 | 75 | 98 | 65 | 1394 | hsa-miR-501-5p | 1190 |  |
| ILMN_3168102 | 2928 | 5091 | 6637 | 5052 | 74 | 73 | 70 | 88 | 60 | 52 | hsa-miR-502-3p,hsa-miR-500* | 1182 |  |
| ILMN_3167210 | 233 | 435 | 435 | 863 | 71 | 73 | 67 | 87 | 57 | 55 | hsa-miR-502-5p | 1191 |  |
| ILMN_3168273 | 3091 | 12530 | 10992 | 6819 | 74 | 72 | 72 | 93 | 62 | 56 | hsa-miR-503 | 1203 |  |
| ILMN_3168485 | 26031 | 5464 | 7938 | 15839 | 10528 | 7964 | 13980 | 21236 | 11758 | 6701 | hsa-miR-504 | 1219 |  |
| ILMN_3168412 | 2329 | 2385 | 2173 | 1178 | 71 | 67 | 64 | 92 | 56 | 45 | hsa-miR-505 | 1222 |  |
| ILMN_3168759 | 603 | 531 | 540 | 1466 | 68 | 64 | 62 | 84 | 55 | 41 | hsa-miR-505* | 5413 |  |
| ILMN_3168328 | 314 | 677 | 776 | 898 | 80 | 78 | 70 | 95 | 60 | 76 | hsa-miR-506 | 1225 |  |
| ILMN_3167727 | 975 | 866 | 1583 | 859 | 179 | 158 | 183 | 328 | 143 | 158 | hsa-miR-507 | 1227 |  |
| ILMN_3168488 | 100 | 241 | 406 | 141 | 66 | 69 | 77 | 82 | 53 | 45 | hsa-miR-508-3p | 1228 |  |
| ILMN_3167636 | 137 | 163 | 128 | 116 | 73 | 71 | 70 | 98 | 61 | 59 | hsa-miR-508-5p | 3726 |  |
| ILMN_3168790 | 77 | 62 | 60 | 91 | 71 | 68 | 67 | 93 | 59 | 49 | hsa-miR-509-3-5p | 4295 |  |
| ILMN_3168363 | 1491 | 151 | 577 | 302 | 5139 | 71 | 70 | 868 | 5179 | 49 | hsa-miR-509-3p | 1229 |  |
| ILMN_3168789 | 263 | 386 | 71 | 74 | 74 | 73 | 71 | 91 | 63 | 44 | hsa-miR-509-5p | 5333 |  |
| ILMN_3168087 | 163 | 129 | 136 | 138 | 74 | 72 | 72 | 93 | 61 | 46 | hsa-miR-510 | 1240 |  |
| ILMN_3167598 | 2944 | 3483 | 4906 | 4464 | 72 | 75 | 70 | 98 | 61 | 50 | hsa-miR-511 | 1241 |  |
| ILMN_3167362 | 1353 | 255 | 453 | 232 | 112 | 105 | 108 | 163 | 88 | 73 | hsa-miR-512-3p | 1248 |  |
| ILMN_3168253 | 4307 | 130 | 321 | 116 | 10356 | 4291 | 4721 | 8350 | 3599 | 5075 | hsa-miR-512-5p | 1250 |  |
| ILMN_3167896 | 128 | 49 | 55 | 65 | 79 | 76 | 76 | 97 | 65 | 48 | hsa-miR-513:9.1 | 1255 |  |
| ILMN_3168779 | 114 | 111 | 116 | 101 | 74 | 72 | 69 | 91 | 59 | 47 | hsa-miR-513a-3p | 74 |  |
| ILMN_3168869 | 1066 | 1145 | 984 | 910 | 220 | 196 | 207 | 327 | 161 | 178 | hsa-miR-513a-5p | 4167 |  |
| ILMN_3168868 | 178 | 113 | 84 | 112 | 73 | 70 | 65 | 91 | 60 | 55 | hsa-miR-513b | 4265 |  |
| ILMN_3167445 | 121 | 146 | 144 | 126 | 77 | 75 | 72 | 96 | 64 | 57 | hsa-miR-513c | 3870 |  |
| ILMN_3168464 | 149 | 48 | 45 | 52 | 68 | 66 | 64 | 90 | 56 | 41 | hsa-miR-514 | 1256 |  |
| ILMN_3167609 | 229 | 244 | 220 | 230 | 142 | 139 | 141 | 152 | 118 | 98 | hsa-miR-515-3p | 1262 |  |
| ILMN_3166959 | 84 | 85 | 73 | 240 | 349 | 70 | 493 | 89 | 59 | 46 | hsa-miR-515-5p | 1265 |  |
| ILMN_3168114 | 714 | 632 | 568 | 521 | 103 | 96 | 109 | 184 | 81 | 94 | hsa-miR-516a-3p,hsa-miR-516b* | 1267 |  |
| ILMN_3168872 | 1159 | 1390 | 1274 | 1114 | 129 | 120 | 127 | 216 | 101 | 119 | hsa-miR-516a-5p | 61 |  |
| ILMN_3168252 | 217 | 105 | 306 | 86 | 81 | 82 | 79 | 101 | 67 | 45 | hsa-miR-516b | 1269 |  |
| ILMN_3168129 | 124 | 187 | 70 | 75 | 69 | 70 | 69 | 94 | 57 | 51 | hsa-miR-517* | 1270 |  |
| ILMN_3168417 | 3061 | 2586 | 3222 | 2576 | 1564 | 560 | 1545 | 1226 | 1591 | 1427 | hsa-miR-517a | 1272 |  |
| ILMN_3167132 | 5691 | 290 | 1352 | 2682 | 9378 | 4129 | 5595 | 9985 | 6074 | 1727 | hsa-miR-517a,hsa-miR-517b | 1276 |  |
| ILMN_3168510 | 519 | 713 | 609 | 908 | 118 | 134 | 107 | 128 | 83 | 73 | hsa-miR-517c | 1277 |  |
| ILMN_3167014 | 6546 | 6266 | 5765 | 5023 | 1050 | 875 | 1116 | 2303 | 916 | 1079 | hsa-miR-518a-3p | 1285 |  |
| ILMN_3168499 | 140 | 82 | 80 | 94 | 86 | 85 | 79 | 101 | 70 | 49 | hsa-miR-518a-5p,hsa-miR-527 | 1462 |  |
| ILMN_3167241 | 7152 | 292 | 236 | 792 | 2424 | 1614 | 2144 | 5547 | 1593 | 272 | hsa-miR-518b | 1307 |  |
| ILMN_3167182 | 139 | 72 | 72 | 71 | 70 | 67 | 64 | 83 | 55 | 47 | hsa-miR-518c | 1309 |  |
| ILMN_3168300 | 515 | 525 | 532 | 599 | 274 | 212 | 858 | 256 | 230 | 244 | hsa-miR-518c* | 1311 |  |
| ILMN_3168373 | 943 | 991 | 960 | 725 | 160 | 149 | 158 | 232 | 130 | 156 | hsa-miR-518d-3p | 1312 |  |
| ILMN_3168546 | 204 | 154 | 153 | 163 | 101 | 81 | 83 | 116 | 67 | 54 | hsa-miR-518e | 5371 |  |
| ILMN_3168075 | 784 | 163 | 878 | 109 | 917 | 72 | 71 | 95 | 66 | 51 | hsa-miR-518e*,hsa-miR-519a*,hsa-miR-519b-5p,hsa-miR-519c-5p,hsa-miR-522*,hsa-miR-523* | 1439 |  |
| ILMN_3167884 | 412 | 315 | 337 | 221 | 79 | 77 | 74 | 97 | 61 | 53 | hsa-miR-518e:9.1 | 1317 |  |
| ILMN_3168740 | 166 | 247 | 535 | 77 | 70 | 67 | 65 | 5634 | 58 | 45 | hsa-miR-518f | 4000 |  |
| ILMN_3168194 | 132 | 98 | 79.5 | 103 | 73 | 69 | 69.5 | 96 | 61 | 49 | hsa-miR-518f*,hsa-miR-518d-5p,hsa-miR-520c-5p,hsa-miR-526a | 1392 |  |
| ILMN_3167757 | 155 | 88 | 440 | 77 | 65 | 64 | 71 | 86 | 57 | 42 | hsa-miR-518f:9.1 | 1319 |  |
| ILMN_3168168 | 174 | 230 | 267 | 285 | 77 | 72 | 71 | 94 | 62 | 48 | hsa-miR-519a | 1321 |  |
| ILMN_3168186 | 1448 | 1683 | 1595 | 1700 | 224 | 211 | 222 | 289 | 171 | 216 | hsa-miR-519b-3p | 1324 |  |
| ILMN_3167937 | 203 | 230 | 318 | 311 | 80 | 78 | 78 | 105 | 68 | 54 | hsa-miR-519c-3p | 1326 |  |
| ILMN_3168354 | 213 | 57 | 250 | 78 | 84 | 76 | 73 | 103 | 68 | 51 | hsa-miR-519d | 1330 |  |
| ILMN_3167306 | 573 | 428 | 320 | 406 | 78 | 88 | 101 | 174 | 75 | 92 | hsa-miR-519e | 1331 |  |
| ILMN_3168031 | 163 | 137 | 70 | 82 | 71 | 68 | 65 | 89 | 57 | 45 | hsa-miR-519e* | 1333 |  |
| ILMN_3167581 | 652 | 542 | 756 | 344 | 80 | 79 | 76 | 115 | 66 | 58 | hsa-miR-520a-3p | 1334 |  |
| ILMN_3168001 | 125 | 115 | 111 | 107 | 83 | 79 | 75 | 98 | 65 | 56 | hsa-miR-520a-5p | 1335 |  |
| ILMN_3167617 | 199 | 147 | 153 | 274 | 97 | 82 | 82 | 126 | 68 | 61 | hsa-miR-520b,hsa-miR-520c-3p,hsa-miR-520f | 1337 |  |
| ILMN_3168043 | 168 | 116 | 107 | 123 | 134 | 93 | 89 | 134 | 86 | 70 | hsa-miR-520c-3p,hsa-miR-520f | 1343 |  |
| ILMN_3167753 | 145 | 184 | 121 | 97 | 75 | 69 | 69 | 90 | 61 | 49 | hsa-miR-520d:9.1 | 1339 |  |
| ILMN_3168548 | 141 | 130 | 153 | 89 | 67 | 66 | 66 | 83 | 56 | 43 | hsa-miR-520d-3p | 5452 |  |
| ILMN_3167831 | 253 | 130 | 122 | 160 | 77 | 76 | 90 | 144 | 72 | 54 | hsa-miR-520d-5p | 1340 |  |
| ILMN_3168242 | 99 | 56 | 305 | 144 | 154 | 69 | 67 | 4088 | 3552 | 177 | hsa-miR-520e | 1341 |  |
| ILMN_3167075 | 106 | 111 | 103 | 108 | 74 | 74 | 67 | 93 | 57 | 47 | hsa-miR-520f | 1338 |  |
| ILMN_3168350 | 69 | 55 | 50 | 74 | 62 | 65 | 61 | 80 | 54 | 42 | hsa-miR-520g | 1344 |  |
| ILMN_3168147 | 378 | 510 | 1578 | 83 | 71 | 72 | 71 | 93 | 59 | 49 | hsa-miR-520h,hsa-miR-520g | 1360 |  |
| ILMN_3168215 | 515 | 60 | 60 | 73 | 71 | 67 | 66 | 87 | 56 | 53 | hsa-miR-521 | 1370 |  |
| ILMN_3167819 | 279 | 294 | 231 | 249 | 77 | 75 | 1521 | 102 | 611 | 58 | hsa-miR-522 | 1375 |  |
| ILMN_3167163 | 148 | 173 | 136 | 163 | 79 | 79 | 75 | 1598 | 64 | 60 | hsa-miR-523 | 1377 |  |
| ILMN_3167328 | 411 | 273 | 400 | 185 | 78 | 6652 | 73 | 106 | 74 | 199 | hsa-miR-524-3p | 1382 |  |
| ILMN_3168284 | 180 | 106 | 110 | 126 | 73 | 73 | 71 | 94 | 61 | 52 | hsa-miR-524-5p | 1384 |  |
| ILMN_3167778 | 411 | 616 | 599 | 583 | 75 | 78 | 69 | 94 | 61 | 57 | hsa-miR-525-3p | 1390 |  |
| ILMN_3167803 | 1262 | 776 | 965 | 729 | 172 | 180 | 242 | 579 | 164 | 145 | hsa-miR-525-5p | 1389 |  |
| ILMN_3168718 | 34 | 34 | 36 | 43 | 88 | 91 | 88 | 107 | 71 | 47 | hsa-miR-526b | 6139 |  |
| ILMN_3167709 | 76 | 56 | 52 | 60 | 69 | 69 | 66 | 87 | 56 | 46 | hsa-miR-526b* | 1432 |  |
| ILMN_3168401 | 158 | 180 | 168 | 170 | 366 | 74 | 73 | 96 | 63 | 53 | hsa-miR-526b:9.1 | 1407 |  |
| ILMN_3168686 | 3478 | 1349 | 3051 | 2557 | 83 | 77 | 81 | 97 | 65 | 53 | hsa-miR-532-3p | 3111 |  |
| ILMN_3167392 | 16130 | 4014 | 5369 | 5869 | 74 | 495 | 4285 | 11261 | 6116 | 62 | hsa-miR-532-5p | 1629 |  |
| ILMN_3167979 | 468 | 2632 | 811 | 337 | 89 | 80 | 72 | 95 | 60 | 73 | hsa-miR-539 | 1482 |  |
| ILMN_3168860 | 125 | 130 | 114 | 124 | 74 | 73 | 71 | 89 | 59 | 55 | hsa-miR-541 | 6030 |  |
| ILMN_3168547 | 504 | 117 | 117 | 138 | 84 | 78 | 80 | 232 | 66 | 58 | hsa-miR-541* | 5759 |  |
| ILMN_3167074 | 194 | 2407 | 631 | 203 | 73 | 70 | 69 | 90 | 60 | 52 | hsa-miR-542-3p | 1493 |  |
| ILMN_3167175 | 265 | 1464 | 1162 | 248 | 76 | 896 | 376 | 96 | 63 | 52 | hsa-miR-542-5p | 1543 |  |
| ILMN_3168545 | 213 | 349 | 273 | 199 | 76 | 74 | 70 | 95 | 62 | 427 | hsa-miR-543 | 4098 |  |
| ILMN_3168272 | 26744 | 24351 | 28795 | 26200 | 25699 | 23217 | 24067 | 23700 | 23573 | 21671 | hsa-miR-544 | 1549 |  |
| ILMN_3167719 | 240 | 152 | 167 | 203 | 85 | 77 | 80 | 95 | 74 | 54 | hsa-miR-545 | 5129 |  |
| ILMN_3168810 | 427 | 540 | 550 | 867 | 71 | 70 | 74 | 91 | 57 | 51 | hsa-miR-545* | 56 |  |
| ILMN_3168175 | 554 | 510 | 521 | 459 | 3602 | 131 | 92 | 127 | 97 | 2297 | hsa-miR-545:9.1 | 1550 |  |
| ILMN_3167646 | 1649 | 1421 | 1502 | 1476 | 218 | 243 | 263 | 533 | 189 | 742 | hsa-miR-548a-3p | 1634 |  |
| ILMN_3168537 | 94 | 101 | 95 | 97 | 68 | 69 | 66 | 90 | 57 | 49 | hsa-miR-548a-5p | 3814 |  |
| ILMN_3167030 | 221 | 277 | 364 | 213 | 181 | 203 | 218 | 126 | 107 | 99 | hsa-miR-548b-3p | 1640 |  |
| ILMN_3168541 | 1968 | 2429 | 2262 | 1888 | 203 | 197 | 204 | 334 | 149 | 400 | hsa-miR-548b-5p | 4230 |  |
| ILMN_3168221 | 302 | 292 | 256 | 252 | 84 | 84 | 83 | 129 | 4144 | 60 | hsa-miR-548c-3p | 1644 |  |
| ILMN_3168540 | 244 | 288 | 205 | 426 | 79 | 79 | 77 | 104 | 64 | 56 | hsa-miR-548c-5p | 4158 |  |
| ILMN_3168227 | 389 | 285 | 339 | 149 | 75 | 72 | 70 | 94 | 61 | 50 | hsa-miR-548d-3p | 1645 |  |
| ILMN_3168072 | 1317 | 1829 | 1767 | 1744 | 92 | 87 | 84 | 108 | 74 | 62 | hsa-miR-548d-5p | 3722 |  |
| ILMN_3168533 | 191 | 186 | 187 | 173 | 73 | 69 | 67 | 88 | 64 | 55 | hsa-miR-548e | 4180 |  |
| ILMN_3168534 | 383 | 390 | 343 | 317 | 104 | 100 | 106 | 129 | 83 | 82 | hsa-miR-548f | 4116 |  |
| ILMN_3168535 | 229 | 127 | 219 | 104 | 74 | 74 | 72 | 90 | 60 | 84 | hsa-miR-548g | 5786 |  |
| ILMN_3168536 | 116 | 74 | 67 | 72 | 72 | 72 | 71 | 94 | 59 | 48 | hsa-miR-548h | 3397 |  |
| ILMN_3168538 | 229 | 341 | 314 | 321 | 79 | 77 | 77 | 101 | 65 | 52 | hsa-miR-548i | 4317 |  |
| ILMN_3168539 | 313 | 131 | 256 | 125 | 79 | 87 | 77 | 102 | 64 | 83 | hsa-miR-548j | 6007 |  |
| ILMN_3168542 | 96 | 90 | 331 | 109 | 75 | 72 | 72 | 94 | 60 | 50 | hsa-miR-548k | 3150 |  |
| ILMN_3168543 | 226 | 207 | 163 | 188 | 81 | 77 | 76 | 109 | 65 | 4611 | hsa-miR-548l | 67 |  |
| ILMN_3168641 | 689 | 820 | 755 | 703 | 77 | 116 | 71 | 204 | 72 | 56 | hsa-miR-548m | 3706 |  |
| ILMN_3168639 | 60 | 54 | 55 | 56 | 70 | 67 | 66 | 86 | 55 | 44 | hsa-miR-548n | 4185 |  |
| ILMN_3168671 | 106 | 193 | 65 | 75 | 77 | 75 | 75 | 101 | 63 | 52 | hsa-miR-548o | 5365 |  |
| ILMN_3168793 | 172 | 281 | 249 | 239 | 80 | 79 | 71 | 102 | 64 | 55 | hsa-miR-548p | 3077 |  |
| ILMN_3167284 | 230 | 320 | 223 | 265 | 84 | 81 | 80 | 112 | 65 | 85 | hsa-miR-549 | 1646 |  |
| ILMN_3168619 | 1358 | 1276 | 1140 | 2103 | 76 | 68 | 72 | 112 | 61 | 52 | hsa-miR-550 | 5997 |  |
| ILMN_3168324 | 1365 | 1034 | 2645 | 3277 | 11243 | 92 | 10851 | 5826 | 8453 | 8147 | hsa-miR-550* | 1647 |  |
| ILMN_3168265 | 2224 | 1569 | 1694 | 2379 | 6831 | 7554 | 697 | 4907 | 741 | 886 | hsa-miR-551a | 1650 |  |
| ILMN_3166993 | 371 | 596 | 943 | 665 | 68 | 68 | 66 | 87 | 58 | 45 | hsa-miR-551b | 1651 |  |
| ILMN_3168741 | 316 | 79 | 700 | 31 | 74 | 70 | 65 | 87 | 56 | 53 | hsa-miR-551b* | 5899 |  |
| ILMN_3167849 | 137 | 80 | 138 | 79 | 112 | 76 | 75 | 113 | 61 | 59 | hsa-miR-552 | 1653 |  |
| ILMN_3167151 | 97 | 122 | 96 | 80 | 70 | 66 | 67 | 88 | 58 | 60 | hsa-miR-553 | 1655 |  |
| ILMN_3167591 | 988 | 897 | 879 | 477 | 85 | 90 | 93 | 3079 | 79 | 421 | hsa-miR-554 | 1658 |  |
| ILMN_3166984 | 142 | 119 | 105 | 103 | 71 | 70 | 70 | 93 | 58 | 46 | hsa-miR-555 | 1661 |  |
| ILMN_3168627 | 61 | 259 | 79 | 59 | 67 | 64 | 65 | 84 | 53 | 41 | hsa-miR-556-3p | 4977 |  |
| ILMN_3167655 | 60 | 127 | 59 | 60 | 69 | 68 | 67 | 88 | 59 | 45 | hsa-miR-556-5p | 1665 |  |
| ILMN_3167268 | 1437 | 89 | 263 | 636 | 74 | 110 | 103 | 3231 | 58 | 69 | hsa-miR-557 | 1671 |  |
| ILMN_3168423 | 65 | 96 | 64 | 70 | 74 | 72 | 241 | 92 | 62 | 2560 | hsa-miR-558 | 1676 |  |
| ILMN_3168387 | 192 | 285 | 999 | 1008 | 74 | 77 | 7215 | 91 | 63 | 64 | hsa-miR-559 | 1678 |  |
| ILMN_3167521 | 2731 | 2793 | 2552 | 1891 | 81 | 76 | 71 | 101 | 64 | 61 | hsa-miR-560:9.1 | 1679 |  |
| ILMN_3167109 | 139 | 148 | 142 | 149 | 94 | 98 | 88 | 111 | 84 | 67 | hsa-miR-561 | 1680 |  |
| ILMN_3168528 | 66 | 52 | 53 | 66 | 71 | 70 | 71 | 90 | 61 | 46 | hsa-miR-562 | 1682 |  |
| ILMN_3167408 | 325 | 308 | 273 | 234 | 94 | 97 | 84 | 104 | 68 | 97 | hsa-miR-563 | 1685 |  |
| ILMN_3167394 | 1088 | 980 | 875 | 765 | 97 | 104 | 91 | 135 | 76 | 79 | hsa-miR-564 | 1688 |  |
| ILMN_3167382 | 17631 | 7139 | 12593 | 3972 | 74 | 69 | 70 | 90 | 60 | 49 | hsa-miR-565:9.1 | 1689 |  |
| ILMN_3167704 | 1418 | 347 | 161 | 162 | 76 | 71 | 70 | 94 | 61 | 50 | hsa-miR-566 | 1693 |  |
| ILMN_3167137 | 258 | 235 | 241 | 218 | 85 | 84 | 85 | 116 | 71 | 61 | hsa-miR-567 | 1697 |  |
| ILMN_3167039 | 178 | 191 | 168 | 2130 | 80 | 73 | 79 | 4291 | 63 | 73 | hsa-miR-568 | 1700 |  |
| ILMN_3168004 | 217 | 149 | 196 | 113 | 74 | 73 | 74 | 108 | 64 | 59 | hsa-miR-569 | 1702 |  |
| ILMN_3167815 | 318 | 232 | 141 | 154 | 79 | 82 | 81 | 115 | 66 | 62 | hsa-miR-570 | 1707 |  |
| ILMN_3166996 | 758 | 741 | 1138 | 1203 | 108 | 105 | 99 | 131 | 76 | 91 | hsa-miR-571 | 1711 |  |
| ILMN_3167492 | 937 | 316 | 558 | 189 | 77 | 78 | 72 | 96 | 64 | 53 | hsa-miR-572 | 1714 |  |
| ILMN_3167242 | 463 | 381 | 354 | 318 | 106 | 107 | 105 | 133 | 83 | 68 | hsa-miR-573 | 1718 |  |
| ILMN_3167656 | 19188 | 10531 | 11289 | 14870 | 69 | 4675 | 68 | 5302 | 61 | 1099 | hsa-miR-574-3p | 1730 |  |
| ILMN_3168836 | 2983 | 3690 | 4392 | 5327 | 93 | 255 | 80 | 113 | 460 | 74 | hsa-miR-574-5p | 3846 |  |
| ILMN_3167433 | 73 | 117 | 50 | 67 | 69 | 67 | 66 | 86 | 55 | 43 | hsa-miR-575 | 1739 |  |
| ILMN_3168559 | 129 | 215 | 198 | 571 | 123 | 106 | 124 | 124 | 101 | 80 | hsa-miR-576-3p | 70 |  |
| ILMN_3167287 | 3857 | 8694 | 9300 | 7827 | 75 | 77 | 71 | 95 | 65 | 53 | hsa-miR-576-5p | 1740 |  |
| ILMN_3167406 | 95 | 76 | 82 | 86 | 68 | 67 | 66 | 88 | 57 | 46 | hsa-miR-577 | 1741 |  |
| ILMN_3168358 | 289 | 357 | 669 | 324 | 77 | 76 | 68 | 90 | 61 | 54 | hsa-miR-578 | 1743 |  |
| ILMN_3167110 | 122 | 60 | 64 | 77 | 78 | 69 | 70 | 88 | 60 | 60 | hsa-miR-579 | 1746 |  |
| ILMN_3167746 | 226 | 327 | 278 | 267 | 80 | 74 | 75 | 100 | 61 | 56 | hsa-miR-580 | 1749 |  |
| ILMN_3168066 | 265 | 459 | 319 | 319 | 90 | 105 | 74 | 1336 | 64 | 64 | hsa-miR-581 | 1753 |  |
| ILMN_3168780 | 123 | 155 | 197 | 146 | 72 | 74 | 68 | 93 | 56 | 73 | hsa-miR-582-3p | 5723 |  |
| ILMN_3168291 | 233 | 963 | 1458 | 180 | 77 | 75 | 73 | 96 | 64 | 51 | hsa-miR-582-5p | 1756 |  |
| ILMN_3168225 | 503 | 714 | 690 | 565 | 124 | 110 | 91 | 125 | 83 | 100 | hsa-miR-583 | 1757 |  |
| ILMN_3167282 | 7561 | 1623 | 2898 | 1515 | 5255 | 3519 | 1155 | 13185 | 4666 | 5421 | hsa-miR-584 | 1759 |  |
| ILMN_3166943 | 681 | 929 | 1175 | 821 | 81 | 89 | 80 | 1959 | 2603 | 60 | hsa-miR-585 | 1761 |  |
| ILMN_3167515 | 300 | 210 | 188 | 203 | 75 | 73 | 70 | 98 | 62 | 60 | hsa-miR-586 | 1762 |  |
| ILMN_3168430 | 125 | 144 | 122 | 425 | 75 | 77 | 68 | 94 | 58 | 51 | hsa-miR-587 | 1765 |  |
| ILMN_3167883 | 1036 | 100 | 99 | 102 | 6528 | 75 | 70 | 4827 | 153 | 49 | hsa-miR-588 | 1766 |  |
| ILMN_3168831 | 18396 | 1527 | 3940 | 5094 | 3571 | 87 | 5053 | 11681 | 7737 | 594 | hsa-miR-589 | 3962 |  |
| ILMN_3167439 | 236 | 144 | 110 | 113 | 75 | 81 | 70 | 93 | 61 | 52 | hsa-miR-589* | 1768 |  |
| ILMN_3168785 | 93 | 97 | 88 | 101 | 83 | 79 | 74 | 102 | 67 | 57 | hsa-miR-590-3p | 5724 |  |
| ILMN_3168498 | 98 | 105 | 106 | 113 | 102 | 92 | 93 | 108 | 86 | 87 | hsa-miR-590-5p | 1771 |  |
| ILMN_3168393 | 835 | 971 | 868 | 784 | 116 | 108 | 101 | 175 | 87 | 111 | hsa-miR-591 | 1772 |  |
| ILMN_3167245 | 389 | 524 | 458 | 414 | 170 | 107 | 125 | 165 | 105 | 96 | hsa-miR-592 | 1775 |  |
| ILMN_3168861 | 269 | 202 | 170 | 201 | 79 | 82 | 76 | 107 | 63 | 62 | hsa-miR-593 | 3330 |  |
| ILMN_3167146 | 79 | 139 | 126 | 98 | 82 | 1063 | 80 | 107 | 65 | 50 | hsa-miR-593* | 1776 |  |
| ILMN_3167299 | 9836 | 8316 | 10819 | 5982 | 74 | 70 | 67 | 90 | 59 | 46 | hsa-miR-594:9.1 | 1777 |  |
| ILMN_3167484 | 14599 | 610 | 1052 | 2576 | 77 | 318 | 1553 | 11852 | 4873 | 328 | hsa-miR-595 | 1780 |  |
| ILMN_3168048 | 461 | 565 | 591 | 509 | 83 | 90 | 75 | 1818 | 61 | 68 | hsa-miR-596 | 1784 |  |
| ILMN_3168141 | 204 | 266 | 245 | 281 | 75 | 70 | 74 | 96 | 61 | 54 | hsa-miR-597 | 1786 |  |
| ILMN_3168425 | 1386 | 3579 | 2187 | 1958 | 70 | 71 | 68 | 89 | 57 | 3678 | hsa-miR-598 | 1787 |  |
| ILMN_3166982 | 77 | 75 | 69 | 150 | 72 | 72 | 70 | 94 | 60 | 49 | hsa-miR-599 | 1789 |  |
| ILMN_3167736 | 201 | 219 | 277 | 215 | 84 | 88 | 81 | 104 | 69 | 73 | hsa-miR-600 | 1790 |  |
| ILMN_3167629 | 157 | 85 | 80 | 80 | 76 | 77 | 70 | 95 | 64 | 48 | hsa-miR-601 | 1795 |  |
| ILMN_3167275 | 145 | 208 | 182 | 142 | 73 | 70 | 68 | 91 | 60 | 64 | hsa-miR-602 | 1799 |  |
| ILMN_3167848 | 758 | 218 | 206 | 276 | 84 | 89 | 115 | 377 | 110 | 63 | hsa-miR-603 | 1804 |  |
| ILMN_3167804 | 136 | 165 | 396 | 68 | 67 | 66 | 66 | 85 | 57 | 44 | hsa-miR-604 | 1808 |  |
| ILMN_3168395 | 212 | 88 | 84 | 125 | 76 | 76 | 71 | 115 | 66 | 321 | hsa-miR-605 | 1823 |  |
| ILMN_3168372 | 52 | 53 | 46 | 58 | 66 | 64 | 62 | 81 | 54 | 41 | hsa-miR-606 | 1824 |  |
| ILMN_3167428 | 304 | 516 | 317 | 445 | 83 | 83 | 82 | 115 | 65 | 71 | hsa-miR-607 | 1825 |  |
| ILMN_3167500 | 160 | 812 | 321 | 84 | 72 | 73 | 66 | 85 | 55 | 55 | hsa-miR-608 | 1826 |  |
| ILMN_3167503 | 702 | 709 | 792 | 769 | 158 | 151 | 139 | 137 | 140 | 140 | hsa-miR-609 | 1828 |  |
| ILMN_3167193 | 665 | 239 | 142 | 136 | 74 | 70 | 69 | 88 | 57 | 51 | hsa-miR-610 | 1836 |  |
| ILMN_3167712 | 65 | 58 | 58 | 70 | 67 | 66 | 65 | 88 | 55 | 43 | hsa-miR-611 | 1838 |  |
| ILMN_3167686 | 606 | 678 | 963 | 1583 | 2115 | 73 | 2663 | 91 | 6332 | 3270 | hsa-miR-612 | 1841 |  |
| ILMN_3167696 | 318 | 363 | 330 | 166 | 85 | 81 | 77 | 104 | 64 | 62 | hsa-miR-613 | 1842 |  |
| ILMN_3167759 | 1629 | 2232 | 966 | 666 | 95 | 100 | 108 | 158 | 80 | 76 | hsa-miR-614 | 1844 |  |
| ILMN_3167508 | 4379 | 4934 | 5781 | 4152 | 333 | 328 | 325 | 741 | 255 | 600 | hsa-miR-615-3p | 1850 |  |
| ILMN_3168761 | 918 | 387 | 462 | 2036 | 98 | 161 | 122 | 124 | 70 | 115 | hsa-miR-615-5p | 3380 |  |
| ILMN_3168617 | 276 | 154 | 76 | 64 | 67 | 65 | 64 | 88 | 56 | 42 | hsa-miR-616 | 4270 |  |
| ILMN_3167770 | 142 | 93 | 419 | 99 | 80 | 78 | 78 | 104 | 67 | 49 | hsa-miR-616* | 1851 |  |
| ILMN_3167452 | 292 | 248 | 254 | 192 | 79 | 75 | 72 | 100 | 64 | 61 | hsa-miR-617 | 1856 |  |
| ILMN_3167768 | 495 | 753 | 745 | 894 | 93 | 81 | 69 | 109 | 65 | 51 | hsa-miR-618 | 1861 |  |
| ILMN_3168490 | 277 | 449 | 298 | 283 | 86 | 88 | 81 | 106 | 72 | 65 | hsa-miR-619 | 1862 |  |
| ILMN_3168009 | 262 | 190 | 228 | 227 | 86 | 91 | 74 | 107 | 65 | 61 | hsa-miR-620 | 1868 |  |
| ILMN_3168486 | 83 | 65 | 56 | 68 | 80 | 73 | 72 | 98 | 64 | 50 | hsa-miR-621 | 1869 |  |
| ILMN_3167141 | 23432 | 1580 | 3995 | 6229 | 10330 | 7968 | 8409 | 15818 | 9862 | 6061 | hsa-miR-622 | 1870 |  |
| ILMN_3167811 | 283 | 140 | 668 | 87 | 72 | 70 | 69 | 99 | 61 | 5387 | hsa-miR-623 | 1871 |  |
| ILMN_3168656 | 285 | 684 | 490 | 352 | 84 | 122 | 84 | 123 | 913 | 133 | hsa-miR-624 | 54 |  |
| ILMN_3167264 | 1084 | 3544 | 3926 | 938 | 105 | 96 | 104 | 122 | 85 | 5492 | hsa-miR-624* | 1873 |  |
| ILMN_3168289 | 6571 | 3233 | 4333 | 15285 | 2863 | 636 | 1585 | 3761 | 3066 | 46 | hsa-miR-625 | 1874 |  |
| ILMN_3168743 | 2539 | 2750 | 1445 | 2065 | 94 | 106 | 89 | 131 | 75 | 123 | hsa-miR-625* | 5768 |  |
| ILMN_3167145 | 361 | 182 | 174 | 2182 | 5055 | 93 | 89 | 2043 | 75 | 59 | hsa-miR-626 | 1876 |  |
| ILMN_3168224 | 709 | 2360 | 3641 | 519 | 69 | 69 | 83 | 88 | 57 | 49 | hsa-miR-627 | 1882 |  |
| ILMN_3167412 | 3768 | 5236 | 6472 | 7627 | 79 | 3280 | 68 | 92 | 61 | 48 | hsa-miR-628-3p | 1883 |  |
| ILMN_3168630 | 4814 | 5708 | 5992 | 6736 | 204 | 8261 | 235 | 155 | 143 | 1771 | hsa-miR-628-5p | 2173 |  |
| ILMN_3168859 | 1650 | 443 | 369 | 127 | 85 | 79 | 82 | 106 | 69 | 55 | hsa-miR-629 | 3968 |  |
| ILMN_3167185 | 335 | 54 | 56 | 587 | 67 | 70 | 64 | 90 | 57 | 45 | hsa-miR-629* | 1884 |  |
| ILMN_3167844 | 101 | 93 | 81 | 102 | 76 | 76 | 74 | 3956 | 62 | 50 | hsa-miR-630 | 1885 |  |
| ILMN_3168034 | 277 | 233 | 671 | 147 | 92 | 86 | 83 | 118 | 73 | 62 | hsa-miR-631 | 1886 |  |
| ILMN_3168336 | 19913 | 1559 | 3598 | 5080 | 9361 | 8046 | 4222 | 15130 | 8685 | 6437 | hsa-miR-632 | 1887 |  |
| ILMN_3167391 | 95 | 82 | 85 | 104 | 87 | 82 | 73 | 94 | 63 | 55 | hsa-miR-633 | 1888 |  |
| ILMN_3167493 | 130 | 152 | 151 | 175 | 101 | 99 | 99 | 121 | 80 | 64 | hsa-miR-634 | 1893 |  |
| ILMN_3168511 | 67 | 152 | 117 | 489 | 67 | 68 | 63 | 86 | 56 | 45 | hsa-miR-635 | 1895 |  |
| ILMN_3168368 | 215 | 262 | 59 | 70 | 73 | 70 | 68 | 91 | 57 | 43 | hsa-miR-636 | 1899 |  |
| ILMN_3167336 | 134 | 162 | 146 | 108 | 71 | 66 | 67 | 85 | 57 | 43 | hsa-miR-637 | 1908 |  |
| ILMN_3168108 | 1457 | 520 | 1954 | 58 | 69 | 68 | 69 | 88 | 57 | 42 | hsa-miR-638 | 1913 |  |
| ILMN_3167286 | 115 | 68 | 179 | 68 | 70 | 5205 | 63 | 86 | 56 | 44 | hsa-miR-639 | 1931 |  |
| ILMN_3167485 | 230 | 91 | 84 | 85 | 73 | 70 | 67 | 92 | 64 | 51 | hsa-miR-640 | 1935 |  |
| ILMN_3167028 | 384 | 544 | 851 | 435 | 79 | 80 | 80 | 102 | 67 | 69 | hsa-miR-641 | 1938 |  |
| ILMN_3168055 | 4982 | 3388 | 7535 | 4301 | 69 | 68 | 67 | 86 | 55 | 45 | hsa-miR-642 | 1942 |  |
| ILMN_3168247 | 453 | 346 | 1066 | 274 | 83 | 75 | 77 | 100 | 62 | 64 | hsa-miR-643 | 1944 |  |
| ILMN_3167580 | 1111 | 475 | 82 | 375 | 75 | 75 | 73 | 127 | 62 | 50 | hsa-miR-644 | 1946 |  |
| ILMN_3167467 | 754 | 412 | 404 | 379 | 84 | 72 | 72 | 236 | 59 | 51 | hsa-miR-645 | 1949 |  |
| ILMN_3168144 | 1812 | 710 | 1459 | 1384 | 7362 | 117 | 129 | 1042 | 105 | 5651 | hsa-miR-646 | 1950 |  |
| ILMN_3167198 | 700 | 450 | 545 | 447 | 127 | 116 | 112 | 182 | 115 | 97 | hsa-miR-647 | 1951 |  |
| ILMN_3168339 | 187 | 112 | 92 | 103 | 83 | 80 | 74 | 106 | 72 | 65 | hsa-miR-648 | 1953 |  |
| ILMN_3167315 | 230 | 158 | 797 | 145 | 80 | 77 | 73 | 108 | 62 | 52 | hsa-miR-649 | 1954 |  |
| ILMN_3167524 | 144 | 417 | 72 | 70 | 73 | 71 | 70 | 92 | 58 | 48 | hsa-miR-650 | 1955 |  |
| ILMN_3167465 | 749 | 822 | 651 | 590 | 99 | 101 | 102 | 145 | 81 | 81 | hsa-miR-651 | 1959 |  |
| ILMN_3167305 | 10314 | 5757 | 7141 | 3518 | 94 | 83 | 87 | 130 | 71 | 521 | hsa-miR-652 | 1961 |  |
| ILMN_3168777 | 311 | 387 | 447 | 325 | 94 | 5017 | 90 | 113 | 5262 | 67 | hsa-miR-653 | 3934 |  |
| ILMN_3168070 | 177 | 339 | 913 | 227 | 103 | 103 | 98 | 125 | 82 | 64 | hsa-miR-653:9.1 | 1967 |  |
| ILMN_3168801 | 21759 | 3024 | 3564 | 6547 | 12237 | 6151 | 7497 | 15542 | 6377 | 7493 | hsa-miR-654-3p | 3640 |  |
| ILMN_3167741 | 848 | 1246 | 815 | 607 | 145 | 120 | 120 | 5959 | 6491 | 65 | hsa-miR-654-5p | 1969 |  |
| ILMN_3168127 | 79 | 240 | 457 | 67 | 72 | 68 | 69 | 91 | 60 | 46 | hsa-miR-655 | 1974 |  |
| ILMN_3168467 | 71 | 54 | 59 | 66 | 71 | 69 | 69 | 91 | 59 | 46 | hsa-miR-656 | 1980 |  |
| ILMN_3167034 | 311 | 227 | 210 | 224 | 102 | 124 | 106 | 151 | 80 | 89 | hsa-miR-657 | 1983 |  |
| ILMN_3168097 | 354 | 67 | 66 | 73 | 74 | 68 | 68 | 91 | 60 | 50 | hsa-miR-658 | 1984 |  |
| ILMN_3168008 | 351 | 277 | 339 | 270 | 75 | 75 | 70 | 91 | 61 | 54 | hsa-miR-659 | 1985 |  |
| ILMN_3168091 | 272 | 266 | 166 | 164 | 88 | 86 | 84 | 109 | 69 | 63 | hsa-miR-660 | 1987 |  |
| ILMN_3167807 | 265 | 216 | 192 | 269 | 75 | 73 | 70 | 95 | 60 | 51 | hsa-miR-661 | 1989 |  |
| ILMN_3167594 | 89 | 56 | 52 | 55 | 67 | 67 | 62 | 86 | 125 | 43 | hsa-miR-662 | 1990 |  |
| ILMN_3167088 | 5261 | 2984 | 3620 | 1991 | 72 | 69 | 67 | 87 | 59 | 44 | hsa-miR-663 | 1994 |  |
| ILMN_3168765 | 2747 | 4267 | 3846 | 2981 | 80 | 78 | 77 | 109 | 72 | 60 | hsa-miR-663b | 3038 |  |
| ILMN_3168802 | 4831 | 6318 | 6344 | 8348 | 77 | 72 | 75 | 1426 | 63 | 1704 | hsa-miR-664 | 3069 |  |
| ILMN_3168592 | 4672 | 4964 | 5243 | 4354 | 93 | 939 | 73 | 100 | 64 | 71 | hsa-miR-664* | 5799 |  |
| ILMN_3168290 | 1381 | 662 | 707 | 766 | 88 | 85 | 80 | 180 | 70 | 68 | hsa-miR-665 | 2715 |  |
| ILMN_3167824 | 670 | 609 | 537 | 203 | 117 | 373 | 102 | 137 | 95 | 379 | hsa-miR-668 | 2008 |  |
| ILMN_3168396 | 425 | 196 | 323 | 327 | 73 | 70 | 71 | 92 | 59 | 46 | hsa-miR-671:9.1 | 2016 |  |
| ILMN_3167053 | 289 | 557 | 756 | 729 | 1284 | 71 | 197 | 1867 | 585 | 848 | hsa-miR-671-3p | 2895 |  |
| ILMN_3168606 | 102 | 101 | 97 | 106 | 73 | 72 | 70 | 91 | 62 | 54 | hsa-miR-671-5p | 4046 |  |
| ILMN_3167407 | 519 | 802 | 829 | 194 | 71 | 70 | 69 | 95 | 57 | 47 | hsa-miR-675 | 5061 |  |
| ILMN_3168388 | 2431 | 5294 | 5788 | 2048 | 76 | 2110 | 76 | 94 | 62 | 66 | hsa-miR-7 | 1553 |  |
| ILMN_3168563 | 3543 | 6414 | 6008 | 4059 | 77 | 74 | 75 | 97 | 65 | 47 | hsa-miR-708 | 3056 |  |
| ILMN_3168647 | 327 | 291 | 203 | 200 | 76 | 73 | 71 | 93 | 62 | 65 | hsa-miR-708* | 5607 |  |
| ILMN_3168644 | 30409 | 23502 | 28399 | 27774 | 16063 | 18685 | 22587 | 24761 | 22385 | 16550 | hsa-miR-7-1* | 3632 |  |
| ILMN_3168645 | 983 | 75 | 192 | 132 | 91 | 69 | 175 | 280 | 57 | 46 | hsa-miR-7-2* | 5350 |  |
| ILMN_3167461 | 19852 | 7563 | 9706 | 9876 | 91 | 7399 | 90 | 1975 | 76 | 4775 | hsa-miR-720 | 3790 |  |
| ILMN_3168846 | 4955 | 3338 | 4904 | 2157 | 88 | 85 | 4108 | 105 | 71 | 1983 | hsa-miR-744 | 5444 |  |
| ILMN_3168733 | 462 | 384 | 325 | 302 | 80 | 78 | 76 | 101 | 66 | 58 | hsa-miR-744* | 3740 |  |
| ILMN_3168216 | 1382 | 125 | 189 | 132 | 70 | 69 | 67 | 945 | 56 | 45 | hsa-miR-760 | 2799 |  |
| ILMN_3167959 | 214 | 203 | 163 | 170 | 83 | 93 | 74 | 108 | 66 | 53 | hsa-miR-765 | 2019 |  |
| ILMN_3167038 | 2087 | 1247 | 1542 | 744 | 78 | 3143 | 72 | 104 | 61 | 2501 | hsa-miR-766 | 2021 |  |
| ILMN_3167893 | 97 | 109 | 104 | 103 | 73 | 71 | 68 | 91 | 60 | 48 | hsa-miR-767-3p | 2022 |  |
| ILMN_3166974 | 2208 | 1441 | 1602 | 387 | 77 | 74 | 72 | 3889 | 61 | 60 | hsa-miR-767-5p | 2024 |  |
| ILMN_3167252 | 26843 | 26097 | 27307 | 24891 | 80 | 2513 | 73 | 95 | 61 | 55 | hsa-miR-768-3p:11.0 | 2027 |  |
| ILMN_3167843 | 7687 | 17120 | 21604 | 17865 | 88 | 83 | 79 | 104 | 73 | 82 | hsa-miR-768-5p:11.0 | 2029 |  |
| ILMN_3168022 | 446 | 386 | 319 | 439 | 84 | 74 | 76 | 101 | 65 | 51 | hsa-miR-769-3p | 2035 |  |
| ILMN_3167148 | 2165 | 2615 | 2329 | 2534 | 116 | 160 | 106 | 139 | 86 | 185 | hsa-miR-769-5p | 2037 |  |
| ILMN_3168455 | 105 | 135 | 85 | 108 | 70 | 72 | 67 | 90 | 58 | 46 | hsa-miR-770-5p | 2038 |  |
| ILMN_3167606 | 477 | 549 | 636 | 77 | 66 | 65 | 64 | 86 | 54 | 49 | hsa-miR-801:9.1 | 2039 |  |
| ILMN_3167200 | 124 | 200 | 161 | 197 | 82 | 73 | 68 | 99 | 64 | 59 | hsa-miR-802 | 2043 |  |
| ILMN_3167953 | 94 | 72 | 70 | 79 | 81 | 76 | 76 | 110 | 70 | 57 | hsa-miR-873 | 5104 |  |
| ILMN_3168723 | 3176 | 2238 | 2022 | 714 | 70 | 68 | 66 | 88 | 60 | 45 | hsa-miR-874 | 3927 |  |
| ILMN_3168690 | 159 | 164 | 157 | 143 | 74 | 74 | 71 | 95 | 62 | 50 | hsa-miR-875-3p | 5348 |  |
| ILMN_3168797 | 475 | 1250 | 783 | 988 | 1104 | 82 | 86 | 3984 | 69 | 74 | hsa-miR-875-5p | 3718 |  |
| ILMN_3168250 | 205 | 106 | 104 | 119 | 100 | 93 | 96 | 129 | 78 | 71 | hsa-miR-876-3p | 2898 |  |
| ILMN_3167672 | 141 | 323 | 146 | 130 | 74 | 75 | 69 | 98 | 61 | 53 | hsa-miR-876-5p | 2896 |  |
| ILMN_3168766 | 2530 | 5065 | 6151 | 4705 | 101 | 5560 | 8060 | 296 | 84 | 5206 | hsa-miR-877 | 4234 |  |
| ILMN_3168817 | 105 | 64 | 148 | 69 | 72 | 1515 | 68 | 98 | 58 | 47 | hsa-miR-877* | 3985 |  |
| ILMN_3167827 | 142 | 112 | 118 | 110 | 71 | 71 | 68 | 91 | 59 | 46 | hsa-miR-885-3p | 3099 |  |
| ILMN_3168813 | 177 | 157 | 65 | 62 | 76 | 71 | 69 | 88 | 60 | 46 | hsa-miR-885-5p | 3239 |  |
| ILMN_3168694 | 3790 | 3292 | 3730 | 956 | 77 | 74 | 74 | 103 | 62 | 59 | hsa-miR-886-3p | 3746 |  |
| ILMN_3168700 | 1230 | 2038 | 2078 | 257 | 71 | 73 | 73 | 96 | 61 | 58 | hsa-miR-886-5p | 5389 |  |
| ILMN_3168770 | 381 | 490 | 357 | 287 | 78 | 79 | 80 | 119 | 67 | 60 | hsa-miR-887 | 3303 |  |
| ILMN_3168788 | 177 | 272 | 330 | 163 | 79 | 79 | 77 | 104 | 67 | 54 | hsa-miR-888 | 3804 |  |
| ILMN_3168744 | 88 | 63 | 296 | 75 | 71 | 68 | 68 | 91 | 59 | 46 | hsa-miR-888* | 4041 |  |
| ILMN_3167991 | 1223 | 6087 | 5985 | 1892 | 130 | 299 | 99 | 132 | 84 | 601 | hsa-miR-889 | 2149 |  |
| ILMN_3168791 | 699 | 870 | 851 | 740 | 113 | 100 | 95 | 130 | 81 | 120 | hsa-miR-890 | 3429 |  |
| ILMN_3167219 | 630 | 420 | 75 | 175 | 71 | 65 | 66 | 87 | 56 | 44 | hsa-miR-891a | 2461 |  |
| ILMN_3168838 | 59 | 54 | 51 | 60 | 68 | 64 | 65 | 85 | 55 | 41 | hsa-miR-891b | 5379 |  |
| ILMN_3168660 | 75 | 70 | 69 | 66 | 72 | 66 | 65 | 86 | 56 | 46 | hsa-miR-892a | 5985 |  |
| ILMN_3168658 | 145 | 75 | 63 | 73 | 74 | 72 | 68 | 92 | 62 | 50 | hsa-miR-892b | 4997 |  |
| ILMN_3167447 | 250 | 6717 | 4027 | 1265 | 71 | 69 | 67 | 91 | 59 | 46 | hsa-miR-9 | 1558 |  |
| ILMN_3167194 | 4103 | 2090 | 884 | 857 | 3897 | 765 | 78 | 6434 | 278 | 2920 | hsa-miR-9* | 1559 |  |
| ILMN_3167527 | 328 | 377 | 452 | 217 | 110 | 115 | 115 | 158 | 88 | 83 | hsa-miR-920 | 4876 |  |
| ILMN_3168707 | 175 | 279 | 249 | 230 | 73 | 80 | 73 | 96 | 61 | 72 | hsa-miR-921 | 5735 |  |
| ILMN_3168748 | 354 | 449 | 373 | 505 | 76 | 77 | 71 | 95 | 60 | 111 | hsa-miR-922 | 4874 |  |
| ILMN_3168768 | 9294 | 24491 | 24705 | 16472 | 748 | 1554 | 68 | 87 | 58 | 53 | hsa-miR-923 | 3089 |  |
| ILMN_3168601 | 377 | 145 | 149 | 1224 | 194 | 114 | 167 | 182 | 169 | 185 | hsa-miR-924 | 4989 |  |
| ILMN_3166986 | 21114 | 23758 | 24079 | 24247 | 75 | 9765 | 7186 | 95 | 5175 | 14873 | hsa-miR-92a | 1562 |  |
| ILMN_3168616 | 352 | 477 | 1596 | 179 | 77 | 72 | 71 | 93 | 64 | 58 | hsa-miR-92a-1* | 6125 |  |
| ILMN_3168763 | 176 | 84 | 66 | 196 | 73 | 70 | 75 | 128 | 68 | 47 | hsa-miR-92a-2* | 3370 |  |
| ILMN_3167360 | 4891 | 6232 | 6469 | 8577 | 148 | 106 | 154 | 150 | 132 | 179 | hsa-miR-92b | 1998 |  |
| ILMN_3168610 | 548 | 205 | 392 | 80 | 73 | 71 | 70 | 89 | 60 | 47 | hsa-miR-92b* | 4300 |  |
| ILMN_3167769 | 12520 | 19226 | 19097 | 16395 | 73 | 4054 | 70 | 91 | 58 | 49 | hsa-miR-93 | 1563 |  |
| ILMN_3168591 | 738 | 834 | 412 | 407 | 77 | 75 | 74 | 94 | 62 | 46 | hsa-miR-93* | 4061 |  |
| ILMN_3168863 | 84 | 100 | 108 | 143 | 76 | 70 | 69 | 94 | 65 | 48 | hsa-miR-933 | 3686 |  |
| ILMN_3168018 | 128 | 145 | 127 | 130 | 71 | 74 | 69 | 94 | 59 | 52 | hsa-miR-934 | 2450 |  |
| ILMN_3168678 | 449 | 788 | 1269 | 741 | 72 | 68 | 67 | 91 | 59 | 46 | hsa-miR-935 | 3347 |  |
| ILMN_3168575 | 529 | 360 | 437 | 448 | 162 | 239 | 9216 | 456 | 300 | 143 | hsa-miR-936 | 3909 |  |
| ILMN_3168629 | 130 | 82 | 139 | 72 | 71 | 69 | 65 | 86 | 60 | 43 | hsa-miR-937 | 75 |  |
| ILMN_3168842 | 27016 | 7247 | 11949 | 19426 | 17316 | 13337 | 17770 | 21878 | 17602 | 12607 | hsa-miR-938 | 5091 |  |
| ILMN_3168855 | 4437 | 6083 | 5824 | 4163 | 565 | 670 | 542 | 1110 | 442 | 848 | hsa-miR-939 | 4028 |  |
| ILMN_3168564 | 20585 | 5123 | 7993 | 11240 | 10989 | 7664 | 9159 | 14097 | 8604 | 7916 | hsa-miR-940 | 3453 |  |
| ILMN_3168281 | 4698 | 1006 | 763 | 99 | 72 | 75 | 67 | 3133 | 63 | 48 | hsa-miR-941 | 2871 |  |
| ILMN_3168829 | 1882 | 740 | 1228 | 210 | 67 | 65 | 64 | 83 | 56 | 1533 | hsa-miR-942 | 3877 |  |
| ILMN_3168720 | 272 | 332 | 298 | 1428 | 83 | 82 | 80 | 107 | 71 | 58 | hsa-miR-943 | 5925 |  |
| ILMN_3168551 | 102 | 237 | 64 | 1439 | 6113 | 286 | 67 | 88 | 58 | 4900 | hsa-miR-944 | 3030 |  |
| ILMN_3166971 | 2239 | 3556 | 4967 | 3857 | 78 | 70 | 69 | 92 | 59 | 45 | hsa-miR-95 | 1564 |  |
| ILMN_3168507 | 682 | 1743 | 2000 | 1007 | 75 | 70 | 68 | 91 | 59 | 52 | hsa-miR-96 | 1565 |  |
| ILMN_3168569 | 109 | 92 | 87 | 94 | 71 | 69 | 67 | 96 | 58 | 48 | hsa-miR-96* | 62 |  |
| ILMN_3167422 | 4820 | 7047 | 6632 | 3919 | 76 | 72 | 71 | 93 | 60 | 49 | hsa-miR-98 | 1577 |  |
| ILMN_3168213 | 10615 | 16676 | 11182 | 11426 | 76 | 75 | 69 | 97 | 66 | 51 | hsa-miR-99a | 1581 |  |
| ILMN_3168648 | 353 | 351 | 463 | 265 | 82 | 84 | 80 | 106 | 68 | 56 | hsa-miR-99a* | 3043 |  |
| ILMN_3168262 | 9240 | 5973 | 5565 | 3387 | 74 | 76 | 71 | 93 | 62 | 42 | hsa-miR-99b | 1586 |  |
| ILMN_3168649 | 3958 | 1709 | 2346 | 977 | 86 | 85 | 81 | 1465 | 72 | 58 | hsa-miR-99b* | 4068 |  |
| ILMN_3168886 | 1438 | 468 | 167 | 464 | 69 | 66 | 66 | 88 | 58 | 45 | solexa-1460-671 | 5670 |  |
| ILMN_3168887 | 757 | 801 | 768 | 667 | 192 | 162 | 178 | 231 | 145 | 158 | solexa-15-44487 | 4901 |  |
| ILMN_3168888 | 72 | 156 | 65 | 72 | 67 | 71 | 69 | 93 | 60 | 45 | solexa-2502-366 | 3824 |  |
| ILMN_3168889 | 220 | 249 | 166 | 166 | 77 | 74 | 72 | 100 | 62 | 55 | solexa-2526-361 | 5157 |  |
| ILMN_3168890 | 87 | 170 | 112 | 124 | 79 | 68 | 65 | 84 | 56 | 54 | solexa-2580-353 | 5160 |  |
| ILMN_3168891 | 138 | 57 | 49 | 59 | 65 | 65 | 62 | 83 | 52 | 41 | solexa-2683-338 | 5357 |  |
| ILMN_3168892 | 17272 | 17403 | 17947 | 15241 | 70 | 960 | 66 | 91 | 56 | 70 | solexa-2952-306 | 1601 |  |
| ILMN_3168893 | 670 | 715 | 712 | 598 | 100 | 96 | 97 | 137 | 77 | 84 | solexa-3022-299 | 3448 |  |
| ILMN_3168894 | 716 | 1148 | 840 | 1043 | 78 | 73 | 72 | 108 | 61 | 48 | solexa-3044-295 | 5823 |  |
| ILMN_3168895 | 670 | 2234 | 1768 | 79 | 76 | 73 | 72 | 3037 | 62 | 48 | solexa-3126-285 | 4183 |  |
| ILMN_3167227 | 311 | 403 | 299 | 179 | 95 | 79 | 79 | 163 | 69 | 65 | solexa-3277-272 | 5851 |  |
| ILMN_3168896 | 12933 | 6412 | 10536 | 1957 | 6002 | 14071 | 9847 | 2286 | 2844 | 1716 | solexa-3464-254 | 5127 |  |
| ILMN_3168897 | 82 | 55 | 469 | 58 | 69 | 68 | 67 | 395 | 60 | 45 | solexa-3695-237 | 3887 |  |
| ILMN_3168898 | 177 | 303 | 401 | 622 | 78 | 78 | 72 | 97 | 65 | 4143 | solexa-3793-229 | 5853 |  |
| ILMN_3167303 | 32250 | 29084 | 33267 | 25574 | 83 | 3542 | 72 | 93 | 65 | 6587 | solexa-3927-221 | 5982 |  |
| ILMN_3168899 | 3110 | 4702 | 3272 | 1883 | 139 | 475 | 124 | 170 | 100 | 765 | solexa-4793-177 | 4276 |  |
| ILMN_3168900 | 5870 | 9982 | 10175 | 13924 | 9872 | 8619 | 9202 | 8634 | 9649 | 64 | solexa-499-2217 | 4104 |  |
| ILMN_3168901 | 998 | 4451 | 5334 | 4285 | 70 | 68 | 65 | 90 | 56 | 42 | solexa-51-13984 | 3840 |  |
| ILMN_3168902 | 108 | 88 | 131 | 74 | 89 | 86 | 85 | 109 | 72 | 51 | solexa-5169-164 | 4060 |  |
| ILMN_3168903 | 10318 | 8155 | 14666 | 15523 | 21527 | 15080 | 20665 | 19803 | 19180 | 18617 | solexa-539-2056 | 3435 |  |
| ILMN_3168904 | 19871 | 16482 | 18555 | 6130 | 4664 | 3445 | 5231 | 902 | 3583 | 4826 | solexa-555-1991 | 3539 |  |
| ILMN_3168905 | 383 | 350 | 309 | 110 | 75 | 70 | 71 | 404 | 62 | 49 | solexa-5620-151 | 3947 |  |
| ILMN_3168906 | 11734 | 1471 | 2552 | 1054 | 66 | 65 | 63 | 86 | 97 | 43 | solexa-578-1915 | 5708 |  |
| ILMN_3168907 | 248 | 87 | 91 | 111 | 81 | 87 | 92 | 147 | 74 | 56 | solexa-5874-144 | 3920 |  |
| ILMN_3167128 | 661 | 1362 | 2461 | 1695 | 5590 | 3232 | 8238 | 1812 | 6976 | 7036 | solexa-603-1846 | 3995 |  |
| ILMN_3168908 | 154 | 255 | 470 | 106 | 5925 | 75 | 71 | 2720 | 65 | 54 | solexa-6676-127 | 4283 |  |
| ILMN_3168909 | 89 | 91 | 76 | 370 | 84 | 79 | 79 | 101 | 68 | 1000 | solexa-7111-119 | 4928 |  |
| ILMN_3168910 | 1145 | 406 | 373 | 363 | 94 | 70 | 71 | 2218 | 61 | 158 | solexa-7297-115 | 4934 |  |
| ILMN_3167037 | 527 | 805 | 672 | 723 | 6594 | 176 | 285 | 280 | 291 | 346 | solexa-7509-112 | 4290 |  |
| ILMN_3168911 | 535 | 859 | 956 | 890 | 75 | 120 | 63 | 90 | 1388 | 59 | solexa-7534-111 | 3608 |  |
| ILMN_3168912 | 6532 | 769 | 1338 | 2050 | 3324 | 1982 | 2072 | 7846 | 2472 | 2344 | solexa-7764-108 | 5842 |  |
| ILMN_3168913 | 127 | 201 | 345 | 227 | 78 | 2536 | 79 | 1798 | 62 | 1988 | solexa-8000-104 | 4090 |  |
| ILMN_3167101 | 2178 | 879 | 1169 | 425 | 9444 | 7015 | 1981 | 188 | 7209 | 6490 | solexa-8048-104 | 41 |  |
| ILMN_3168914 | 1643 | 6768 | 5342 | 5242 | 6924 | 312 | 64 | 4486 | 58 | 557 | solexa-8211-102 | 4893 |  |
| ILMN_3168915 | 1086 | 1044 | 795 | 1286 | 1613 | 79 | 140 | 94 | 64 | 1534 | solexa-826-1288 | 5291 |  |
| ILMN_3168916 | 667 | 582 | 1646 | 818 | 1861 | 98 | 1470 | 178 | 96 | 103 | solexa-8926-93 | 6048 |  |
| ILMN_3168917 | 6661 | 7904 | 7801 | 7854 | 12401 | 1640 | 12571 | 2291 | 7811 | 7798 | solexa-9029-92 | 2820 |  |
| ILMN_3167228 | 4098 | 2670 | 5215 | 2546 | 9080 | 90 | 5170 | 5549 | 5921 | 66 | solexa-9081-91 | 5572 |  |
| ILMN_3168918 | 3320 | 1125 | 1792 | 1064 | 5051 | 2722 | 736 | 5570 | 996 | 179 | solexa-9124-90 | 3237 |  |
| ILMN_3168919 | 1134 | 843 | 874 | 1075 | 4048 | 110 | 5124 | 2048 | 4859 | 1191 | solexa-9578-86 | 5650 |  |
| ILMN_3168920 | 1054 | 1187 | 2186 | 667 | 120 | 604 | 2721 | 5145 | 8148 | 2701 | solexa-9655-85 | 4099 |  |
|  |  |  |  |  |  |  |  |  |  |  |  |  |  |
