# Supplementary material for: Plasma MicroRNAs as Potential Noninvasive Biomarkers for In-Stent Restenosis
Source: PLoS One. 2014 Nov 26;9(11):e112043. doi: 10.1371/journal.pone.0112043 (PMC4245195; doi:10.1371/journal.pone.0112043)
Supplement: Table S2 — Microarray data were normalized by the quantile normalization. (DOC) [file pone.0112043.s002.doc]

Table S2. Microarray data were normalized by the quantile normalization.

| Symbol | non.ISR.1.AVG | non.ISR.2.AVG | non.ISR.3.AVG | non.ISR.4.AVG | ISR.1.AVG | ISR.2.AVG | ISR.3.AVG | ISR.4.AVG | ISR.5.AVG | ISR.6.AVG | ttst P Value | FC |
| --- | --- | --- | --- | --- | --- | --- | --- | --- | --- | --- | --- | --- |
| HS_1 | 5.911691582 | 6.469234794 | 5.842978832 | 5.894817763 | 6.399171094 | 6.316507819 | 6.523561956 | 7.388878339 | 6.584962501 | 5.92243574 | 0.080600755 | 0.492905499 |
| HS_10 | 7.572131751 | 6.845490051 | 6.375908263 | 8.684047262 | 6.671010241 | 6.316507819 | 6.523561956 | 6.261154673 | 6.250772132 | 7.270996061 | 0.202183147 | -0.820393852 |
| HS_100 | 13.39700607 | 13.41629614 | 13.38548496 | 12.75713995 | 5.980710829 | 5.911691582 | 5.988684687 | 5.820178962 | 12.96155822 | 11.65745244 | 0.012044919 | -5.185602328 |
| HS_101 | 8.773468928 | 6.332707934 | 6.121015401 | 6.601399391 | 6.155830172 | 6.196725061 | 6.089582893 | 6.261154673 | 6.584962501 | 6.569855608 | 0.370079832 | -0.647462762 |
| HS_104 | 7.395319911 | 6.36018867 | 7.892998093 | 8.255500733 | 6.155830172 | 6.196725061 | 6.089582893 | 7.388878339 | 6.584962501 | 6.194756854 | 0.078819367 | -1.040879215 |
| HS_105 | 8.518849829 | 7.913487841 | 8.347842794 | 7.687900522 | 6.862947248 | 6.755555262 | 6.356672044 | 6.422905743 | 6.411087227 | 8.248876343 | 0.006917919 | -1.274012935 |
| HS_106 | 6.849248703 | 6.880195729 | 7.45532722 | 7.094869433 | 8.893756568 | 8.710462074 | 8.607700188 | 8.003096215 | 8.838573653 | 8.248876343 | 9.98E-05 | 1.480500569 |
| HS_107 | 5.898450233 | 6.029011087 | 5.988684687 | 6.105384749 | 6.05202456 | 6.196725061 | 5.988684687 | 5.988684687 | 6.250772132 | 6.329123596 | 0.115818909 | 0.128953098 |
| HS_108.1 | 13.41629614 | 11.88542947 | 11.849366 | 11.89958351 | 8.800252806 | 6.609548139 | 8.889199726 | 7.388878339 | 7.117902789 | 6.714245518 | 4.15E-05 | -4.675997559 |
| HS_109 | 6.900866808 | 5.988684687 | 6.05202456 | 6.421223299 | 7.641690523 | 7.316960287 | 8.002252452 | 7.515305605 | 8.300123725 | 6.45779126 | 0.007101396 | 1.198320803 |
| HS_11.1 | 6.483815777 | 6.636624621 | 6.628627802 | 8.018200179 | 8.607700188 | 8.437544026 | 8.986979709 | 8.405566974 | 8.838573653 | 7.625708843 | 0.01434032 | 1.541861804 |
| HS_110 | 8.275193334 | 8.539158811 | 8.643495471 | 8.919459269 | 8.151523994 | 10.36062765 | 7.78496195 | 7.1017131 | 6.83541884 | 8.814422247 | 0.470268094 | -0.419548758 |
| HS_111 | 8.342963574 | 6.318316841 | 6.292781749 | 6.136478518 | 6.399171094 | 6.316507819 | 6.207502459 | 6.543805176 | 6.411087227 | 6.329123596 | 0.497593005 | -0.404768942 |
| HS_112 | 6.301953395 | 6.483815777 | 6.027905997 | 6.053111336 | 5.905687849 | 5.911691582 | 5.927185358 | 10.41309898 | 5.9795681 | 6.100136671 | 0.541556325 | 0.489531464 |
| HS_113 | 8.388447876 | 8.566054038 | 8.379378367 | 8.626439137 | 10.21188829 | 9.226773423 | 8.889199726 | 7.857047916 | 8.597307691 | 9.688862232 | 0.144403412 | 0.588433359 |
| HS_114 | 9.076281167 | 9.6121313 | 9.528063348 | 10.10407452 | 11.7417619 | 8.710462074 | 8.289557848 | 7.857047916 | 7.864805194 | 8.862017377 | 0.3139581 | -0.692528865 |
| HS_115 | 7.43629512 | 6.019034669 | 7.974988112 | 6.196725061 | 7.484218708 | 7.316960287 | 7.599168993 | 11.139935 | 7.117902789 | 6.569855608 | 0.274481872 | 0.96457949 |
| HS_116 | 8.660174212 | 8.364134655 | 7.66035251 | 9.188712465 | 7.273795599 | 6.951867504 | 6.721782768 | 7.661778098 | 7.117902789 | 7.006746832 | 0.017197245 | -1.346031195 |
| HS_117 | 7.176422513 | 6.292781749 | 6.329123596 | 6.601399391 | 8.151523994 | 7.316960287 | 7.78496195 | 7.23935985 | 7.864805194 | 6.857980995 | 0.012075436 | 0.936000233 |
| HS_119 | 8.141596278 | 7.491853096 | 7.290940402 | 7.713558356 | 8.151523994 | 8.040837649 | 9.57799522 | 10.03589879 | 9.207990433 | 8.248876343 | 0.016058199 | 1.217700038 |
| HS_12 | 10.96079838 | 6.373300197 | 8.567385727 | 6.512542955 | 6.155830172 | 6.196725061 | 6.089582893 | 6.168922782 | 5.9795681 | 6.194756854 | 0.164214529 | -1.972609171 |
| HS_120 | 6.559950613 | 6.510961919 | 6.622783966 | 6.707359132 | 8.893756568 | 8.521207816 | 9.160249616 | 8.003096215 | 9.207990433 | 7.502235115 | 0.000802992 | 1.947825386 |
| HS_121 | 6.396604781 | 6.651769271 | 6.622783966 | 6.759555343 | 9.049167872 | 7.625708843 | 8.289557848 | 7.753885283 | 7.864805194 | 7.39145842 | 0.001647688 | 1.38808557 |
| HS_122.1 | 7.930441673 | 7.845490051 | 7.449561375 | 7.951867504 | 9.484420132 | 9.443565213 | 9.650154214 | 9.11204848 | 9.958697703 | 9.425740424 | 1.05E-05 | 1.718097543 |
| HS_123 | 6.927185358 | 6.957102042 | 6.874059203 | 7.159871337 | 10.70091649 | 10.16440416 | 10.84889674 | 9.783898589 | 10.92888844 | 9.044667331 | 7.65E-05 | 3.265724141 |
| HS_124 | 5.923624611 | 5.851749041 | 5.857980995 | 5.842978832 | 6.862947248 | 6.465974465 | 7.400025518 | 7.23935985 | 6.83541884 | 6.329123596 | 0.0020707 | 0.98639155 |
| HS_126 | 8.486231678 | 8.58082385 | 8.675957033 | 8.775116404 | 7.273795599 | 7.316960287 | 8.413204789 | 7.927777962 | 13.38548496 | 7.006746832 | 0.942147425 | -0.075537169 |
| HS_127.1 | 7.155830172 | 6.089582893 | 5.977279923 | 6.155830172 | 7.641690523 | 6.755555262 | 7.78496195 | 7.661778098 | 8.453682186 | 6.857980995 | 0.015477091 | 1.181310712 |
| HS_128 | 7.427103287 | 7.651410526 | 7.499845887 | 8.612499946 | 8.893756568 | 7.921245889 | 8.167418146 | 7.23935985 | 7.864805194 | 8.543805176 | 0.425110499 | 0.307350226 |
| HS_129 | 7.273795599 | 6.287250643 | 6.469234794 | 6.601399391 | 8.800252806 | 7.75087444 | 8.730809755 | 8.566054038 | 8.959132577 | 7.006746832 | 0.002542244 | 1.644391635 |
| HS_13 | 6.177917792 | 6.74281467 | 6.597680144 | 6.876516947 | 6.530601415 | 6.755555262 | 6.523561956 | 6.0725346 | 6.411087227 | 7.502235115 | 0.894729649 | 0.033863541 |
| HS_130 | 6.849248703 | 7.146186791 | 6.87774425 | 7.235535607 | 8.151523994 | 8.25502857 | 8.289557848 | 7.753885283 | 8.11997861 | 8.063395081 | 7.89E-05 | 1.078382727 |
| HS_131 | 7.017365205 | 7.776103988 | 7.099084761 | 7.682292371 | 5.837943242 | 7.501040996 | 5.62935662 | 5.942514505 | 5.9795681 | 6.857980995 | 0.015066807 | -1.102310838 |
| HS_132.1 | 6.62935662 | 7.106432078 | 7.011227255 | 7.263034406 | 5.980710829 | 6.092757141 | 5.988684687 | 6.332707934 | 5.9795681 | 6.001126665 | 0.002810397 | -0.93992003 |
| HS_133.1 | 7.155830172 | 7.735725245 | 7.166916252 | 9.299665944 | 8.518849829 | 8.710462074 | 9.32395546 | 8.830990481 | 8.959132577 | 9.184627436 | 0.119187341 | 1.081801906 |
| HS_134 | 6.348728154 | 6.789859641 | 6.925999419 | 7.293701542 | 6.155830172 | 7.921245889 | 6.523561956 | 6.168922782 | 6.584962501 | 8.689299161 | 0.732390017 | 0.167731554 |
| HS_135 | 9.412569847 | 6.493455201 | 7.6727789 | 6.338959175 | 6.155830172 | 6.196725061 | 6.930145948 | 6.422905743 | 6.10433666 | 7.006746832 | 0.251400051 | -1.009992378 |
| HS_136 | 6.570614722 | 6.154818109 | 6.161887682 | 6.224966365 | 10.1365426 | 7.75087444 | 8.002252452 | 8.486231678 | 6.83541884 | 7.712870868 | 0.008211915 | 1.875960094 |
| HS_137 | 6.093813673 | 6.14363831 | 6.60659028 | 6.374170076 | 7.484218708 | 8.040837649 | 7.599168993 | 6.332707934 | 7.635173947 | 6.857980995 | 0.008302073 | 1.020461619 |
| HS_138 | 7.958262697 | 7.710117632 | 7.499845887 | 7.736740155 | 10.44159552 | 9.443565213 | 10.2564446 | 9.821455119 | 9.958697703 | 9.57799522 | 3.37E-06 | 2.190383969 |
| HS_139 | 9.77016832 | 9.958697703 | 9.071194179 | 9.462297766 | 10.8622499 | 10.39687232 | 11.20939244 | 11.1028287 | 11.06709819 | 10.4268937 | 0.001742743 | 1.278633048 |
| HS_14.1 | 6.557655155 | 6.545350645 | 7.433794059 | 6.731997787 | 6.530601415 | 7.75087444 | 7.145677455 | 7.1017131 | 6.584962501 | 7.625708843 | 0.33194868 | 0.306056881 |
| HS_140 | 7.735725245 | 6.029011087 | 6.121015401 | 6.45779126 | 6.671010241 | 6.609548139 | 8.002252452 | 11.99848466 | 6.584962501 | 6.001126665 | 0.323550204 | 1.058678361 |
| HS_141 | 8.008988783 | 7.906289347 | 7.525129251 | 7.810571635 | 7.273795599 | 7.316960287 | 7.599168993 | 10.44935502 | 7.117902789 | 7.141596278 | 0.994767132 | 0.003718408 |
| HS_142.1 | 7.993221467 | 8.275193334 | 9.620036498 | 8.275193334 | 12.62956153 | 7.75087444 | 8.289557848 | 8.003096215 | 8.597307691 | 8.063395081 | 0.691367949 | 0.34805431 |
| HS_143 | 6.653919873 | 6.122051448 | 7.921245889 | 6.311975314 | 9.412569847 | 6.196725061 | 5.988684687 | 6.168922782 | 5.888743249 | 6.100136671 | 0.859392869 | -0.126334415 |
| HS_144 | 6.800252806 | 7.038918989 | 7.089582893 | 8.461479447 | 6.862947248 | 7.117383367 | 6.523561956 | 6.543805176 | 6.584962501 | 8.063395081 | 0.411329736 | -0.398215979 |
| HS_145.1 | 8.413204789 | 8.25502857 | 7.970393538 | 8.518849829 | 10.09882166 | 9.91064273 | 10.45491614 | 12.55837288 | 10.21188829 | 9.202491187 | 0.005191952 | 2.116819634 |
| HS_146.1 | 6.577428828 | 6.5360529 | 7.036723106 | 6.721782768 | 7.484218708 | 6.951867504 | 7.400025518 | 7.596935142 | 7.864805194 | 7.141596278 | 0.004364448 | 0.688577824 |
| HS_147 | 7.970393538 | 6.903279342 | 8.033973543 | 6.857980995 | 6.671010241 | 6.951867504 | 6.523561956 | 7.1017131 | 7.117902789 | 7.712870868 | 0.298340984 | -0.428252445 |
| HS_149 | 9.462297766 | 8.302867361 | 9.818582177 | 8.956666555 | 9.412569847 | 9.024308597 | 9.223036338 | 8.326204751 | 13.24425976 | 8.862017377 | 0.516451359 | 0.546962647 |
| HS_15.1 | 6.768184325 | 6.332707934 | 6.221103725 | 6.171927354 | 7.075746538 | 6.755555262 | 7.145677455 | 6.790511412 | 6.83541884 | 6.45779126 | 0.031463973 | 0.469969293 |
| HS_150 | 10.36654079 | 9.412569847 | 9.13481133 | 9.474719947 | 10.82631014 | 10.80727441 | 11.3810562 | 11.59577031 | 11.63444806 | 10.56985561 | 0.003520911 | 1.538625311 |
| HS_151.1 | 6.201633861 | 6.008988783 | 6.010108453 | 6.105384749 | 6.671010241 | 6.465974465 | 6.523561956 | 6.422905743 | 6.584962501 | 6.100136671 | 0.004024727 | 0.379896301 |
| HS_152 | 8.87036472 | 9.082149041 | 8.92659251 | 9.248164779 | 9.67357409 | 9.443565213 | 9.650154214 | 8.858291887 | 8.959132577 | 9.893756568 | 0.084758866 | 0.381261329 |
| HS_153 | 7.698357406 | 7.900866808 | 7.48058827 | 7.87282876 | 7.484218708 | 7.75087444 | 8.167418146 | 10.47421294 | 7.635173947 | 8.689299161 | 0.232130951 | 0.628705912 |
| HS_154 | 6.36719631 | 6.448735798 | 6.250772132 | 6.473299871 | 6.155830172 | 6.316507819 | 6.089582893 | 6.0725346 | 6.10433666 | 6.100136671 | 0.007496745 | -0.245179559 |
| HS_155 | 6.495855027 | 6.287250643 | 5.894817763 | 5.861707287 | 6.274261661 | 6.196725061 | 6.207502459 | 6.543805176 | 6.411087227 | 6.001126665 | 0.464885884 | 0.137510362 |
| HS_156 | 6.155830172 | 6.046578367 | 6.175923742 | 6.87036472 | 6.530601415 | 6.609548139 | 6.356672044 | 6.168922782 | 6.411087227 | 6.100136671 | 0.816624903 | 0.050653796 |
| HS_157 | 6.99095486 | 7.032321287 | 7.188836073 | 6.566815154 | 5.62935662 | 5.716990894 | 7.78496195 | 5.752213368 | 5.888743249 | 5.675251386 | 0.053896082 | -0.870145599 |
| HS_159 | 7.155830172 | 8.068509595 | 6.502235115 | 6.325530332 | 6.862947248 | 6.609548139 | 6.930145948 | 6.668175869 | 6.584962501 | 7.141596278 | 0.631028331 | -0.213463639 |
| HS_16 | 6.23457796 | 6.5360529 | 6.44625623 | 6.74281467 | 6.274261661 | 6.092757141 | 6.089582893 | 6.668175869 | 5.888743249 | 6.100136671 | 0.080982096 | -0.304315859 |
| HS_160 | 7.561478892 | 7.925109324 | 7.943686952 | 8.090906345 | 8.151523994 | 7.501040996 | 7.599168993 | 7.1017131 | 8.300123725 | 7.712870868 | 0.492835854 | -0.152555099 |
| HS_161 | 6.175923742 | 8.227856573 | 6.05202456 | 8.271463028 | 7.273795599 | 7.117383367 | 7.78496195 | 7.857047916 | 8.11997861 | 6.569855608 | 0.701988857 | 0.272020199 |
| HS_162 | 6.031218731 | 6.21916852 | 6.250772132 | 6.325530332 | 7.273795599 | 7.316960287 | 8.002252452 | 7.23935985 | 7.408117408 | 7.270996061 | 3.51E-05 | 1.211907848 |
| HS_163 | 7.99095486 | 7.1017131 | 6.283551423 | 6.354028938 | 7.273795599 | 6.609548139 | 7.145677455 | 6.422905743 | 6.584962501 | 6.45779126 | 0.689375246 | -0.183448631 |
| HS_164 | 6.340740281 | 6.678071905 | 6.302867361 | 6.473299871 | 6.862947248 | 6.951867504 | 7.78496195 | 6.668175869 | 7.117902789 | 6.329123596 | 0.055481456 | 0.503751638 |
| HS_166.1 | 8.781359714 | 8.160879862 | 7.533719071 | 7.958262697 | 9.14822234 | 9.177419538 | 10.17816685 | 9.783898589 | 9.098821663 | 7.712870868 | 0.03693447 | 1.074677973 |
| HS_167.1 | 7.544578117 | 7.721782768 | 7.726558779 | 10.01066796 | 9.67357409 | 9.226773423 | 10.43426334 | 8.897542973 | 10.31118066 | 8.910492832 | 0.105582627 | 1.324740979 |
| HS_168 | 7.695228291 | 8.653919873 | 8.093285504 | 8.470048726 | 9.14822234 | 8.437544026 | 8.889199726 | 8.60936345 | 8.453682186 | 8.324180547 | 0.153147826 | 0.415578114 |
| HS_169 | 7.802839441 | 9.945004816 | 7.414473836 | 6.23457796 | 6.155830172 | 6.465974465 | 6.721782768 | 7.1017131 | 6.584962501 | 6.45779126 | 0.199793668 | -1.267881636 |
| HS_17 | 10.46546437 | 7.586464526 | 9.601399391 | 8.15936681 | 6.155830172 | 6.316507819 | 6.089582893 | 6.168922782 | 6.10433666 | 6.194756854 | 0.024111963 | -2.781517578 |
| HS_170 | 10.20371505 | 10.65391987 | 10.33918193 | 6.431288654 | 6.862947248 | 7.625708843 | 7.599168993 | 7.1017131 | 7.117902789 | 7.141596278 | 0.117223165 | -2.16552017 |
| HS_174.1 | 7.499845887 | 6.569855608 | 6.211401637 | 6.224966365 | 6.155830172 | 5.992088609 | 6.207502459 | 6.168922782 | 6.10433666 | 6.329123596 | 0.220735175 | -0.466883328 |
| HS_175 | 7.664625055 | 7.71596199 | 6.99095486 | 7.279842694 | 8.800252806 | 8.710462074 | 9.160249616 | 8.763876285 | 9.098821663 | 7.925109324 | 0.000781192 | 1.330282478 |
| HS_176 | 11.79445648 | 9.073739915 | 9.517472554 | 8.342963574 | 12.89026428 | 13.5347649 | 13.83329532 | 14.11566957 | 13.83329532 | 12.179878 | 0.009933161 | 3.715703099 |
| HS_177 | 6.957682486 | 7.299665944 | 7.160879862 | 7.519243094 | 7.799605422 | 7.75087444 | 8.289557848 | 7.753885283 | 11.09275714 | 7.270996061 | 0.114371793 | 1.091911519 |
| HS_179 | 8.018200179 | 7.721782768 | 6.936637939 | 7.502235115 | 7.641690523 | 7.921245889 | 12.32673844 | 6.953032377 | 6.83541884 | 7.270996061 | 0.513352599 | 0.613473022 |
| HS_18 | 7.637349411 | 6.766860144 | 6.36018867 | 6.483815777 | 6.530601415 | 5.992088609 | 6.721782768 | 6.0725346 | 6.584962501 | 6.857980995 | 0.328784501 | -0.352061686 |
| HS_182.1 | 9.924812504 | 7.980710829 | 9.97053734 | 7.499845887 | 7.484218708 | 7.921245889 | 7.145677455 | 7.388878339 | 11.51491126 | 6.714245518 | 0.422328381 | -0.815780444 |
| HS_183.1 | 5.752213368 | 5.675251386 | 5.696272084 | 5.910492832 | 5.905687849 | 5.856736756 | 5.927185358 | 5.861707287 | 5.9795681 | 5.675251386 | 0.156926627 | 0.109132038 |
| HS_184 | 9.787902559 | 9.548436625 | 9.541677449 | 9.56414949 | 10.47421294 | 10.08832449 | 13.47772033 | 10.08308037 | 10.42500604 | 10.42311591 | 0.071678674 | 1.218035148 |
| HS_185.1 | 6.754887502 | 6.112700133 | 6.211401637 | 6.389738879 | 6.530601415 | 6.316507819 | 6.207502459 | 6.261154673 | 6.584962501 | 6.194756854 | 0.914125821 | -0.017934418 |
| HS_186 | 8.481799432 | 8.889199726 | 8.75788999 | 8.894817763 | 9.730979533 | 11.33220442 | 9.77016832 | 9.014299495 | 9.692266772 | 10.1321141 | 0.011473711 | 1.189412047 |
| HS_187 | 7.616181231 | 6.301953395 | 7.810571635 | 6.483815777 | 6.05202456 | 5.911691582 | 6.089582893 | 6.0725346 | 6.250772132 | 6.194756854 | 0.08758962 | -0.957903406 |
| HS_188 | 10.65391987 | 8.826389591 | 9.916028718 | 8.283319907 | 8.381110294 | 8.040837649 | 13.19746245 | 9.435878577 | 8.959132577 | 8.063395081 | 0.940963987 | -0.073611752 |
| HS_189.1 | 6.465974465 | 6.339850003 | 7.056366761 | 6.171927354 | 6.274261661 | 6.755555262 | 6.523561956 | 6.422905743 | 6.411087227 | 6.194756854 | 0.726404883 | -0.078174862 |
| HS_19 | 9.56414949 | 9.429825157 | 9.787902559 | 9.236970886 | 11.2444233 | 10.54515755 | 11.26982798 | 10.50143915 | 11.15785217 | 10.56985561 | 9.87E-05 | 1.376713937 |
| HS_190 | 6.292781749 | 6.112700133 | 6.268658955 | 6.354028938 | 6.05202456 | 6.951867504 | 7.145677455 | 7.1017131 | 6.250772132 | 6.714245518 | 0.062951327 | 0.445674268 |
| HS_192.1 | 12.46867495 | 11.59577031 | 12.12350713 | 10.36654079 | 5.980710829 | 6.092757141 | 5.927185358 | 6.332707934 | 5.804776378 | 5.842978832 | 0.00092209 | -5.641770548 |
| HS_193 | 7.978424465 | 7.006746832 | 7.785942819 | 7.159871337 | 9.14822234 | 9.101450482 | 9.650154214 | 8.725877459 | 9.098821663 | 8.961594391 | 0.002137804 | 1.631607061 |
| HS_194 | 8.168922782 | 5.894817763 | 5.902073579 | 6.957102042 | 6.155830172 | 6.092757141 | 10.17816685 | 5.942514505 | 6.10433666 | 6.001126665 | 0.986678523 | 0.015059625 |
| HS_195 | 6.383704292 | 6.510961919 | 6.569855608 | 6.74281467 | 8.68580002 | 8.60936345 | 8.986979709 | 8.203837384 | 9.328338334 | 11.72144102 | 0.003130635 | 2.704125864 |
| HS_196.1 | 8.534302882 | 6.715618859 | 7.126188211 | 6.389738879 | 6.274261661 | 6.092757141 | 6.207502459 | 6.261154673 | 6.10433666 | 6.001126665 | 0.115682589 | -1.034605664 |
| HS_197 | 6.570614722 | 6.60659028 | 6.518062976 | 6.74281467 | 7.799605422 | 7.625708843 | 7.78496195 | 7.23935985 | 7.635173947 | 8.248876343 | 0.000195488 | 1.112760397 |
| HS_198 | 6.112700133 | 6.029011087 | 6.05202456 | 6.004501392 | 6.399171094 | 6.316507819 | 6.721782768 | 6.668175869 | 7.864805194 | 6.194756854 | 0.048394042 | 0.64464064 |
| HS_199 | 8.084808388 | 7.566054038 | 7.438791853 | 7.640606469 | 7.484218708 | 8.040837649 | 6.930145948 | 6.668175869 | 6.83541884 | 9.250416861 | 0.739334205 | -0.147696207 |
| HS_2 | 8.113221243 | 8.208478242 | 8.554972496 | 7.508587112 | 7.982993575 | 7.75087444 | 8.002252452 | 7.661778098 | 7.635173947 | 7.925109324 | 0.308666038 | -0.269951134 |
| HS_20 | 9.164152708 | 9.171927354 | 8.87036472 | 9.123086751 | 10.09882166 | 10.38046107 | 11.07487734 | 11.02160465 | 11.16804527 | 10.14095755 | 0.000294162 | 1.565078373 |
| HS_200 | 8.280306806 | 7.477353527 | 7.511752654 | 7.410239331 | 9.969170641 | 8.710462074 | 9.223036338 | 8.326204751 | 9.207990433 | 8.589838376 | 0.00295195 | 1.334537356 |
| HS_201 | 7.790511412 | 7.790511412 | 8.297374861 | 7.799605422 | 9.857825524 | 9.644306962 | 9.958697703 | 8.897542973 | 9.958697703 | 8.589838376 | 0.000644337 | 1.564984096 |
| HS_202.1 | 13.70497352 | 9.809125002 | 10.48290877 | 11.64029013 | 12.65244168 | 13.36342298 | 13.87028765 | 14.04598144 | 13.7905725 | 12.94553528 | 0.093531896 | 2.035382566 |
| HS_203 | 8.227856573 | 8.259507895 | 8.30833903 | 7.747185967 | 9.049167872 | 8.521207816 | 8.986979709 | 8.326204751 | 8.725877459 | 8.168922782 | 0.035686879 | 0.494004365 |
| HS_204.1 | 13.27035373 | 13.00499288 | 13.05706098 | 13.00499288 | 13.94706157 | 13.65089467 | 13.60579201 | 13.87028765 | 13.70497352 | 13.70497352 | 0.000107895 | 0.662980372 |
| HS_205.1 | 6.422905743 | 6.332707934 | 6.36018867 | 6.578938713 | 8.259507895 | 7.117383367 | 8.413204789 | 8.203837384 | 8.11997861 | 8.063395081 | 0.00021431 | 1.605865923 |
| HS_206 | 7.002252452 | 7.596935142 | 7.5980525 | 6.121015401 | 5.905687849 | 5.992088609 | 5.988684687 | 5.988684687 | 5.9795681 | 6.001126665 | 0.050643648 | -1.103590441 |
| HS_208 | 6.661778098 | 6.778734244 | 8.014578465 | 7.600656308 | 7.641690523 | 8.521207816 | 7.78496195 | 7.388878339 | 8.300123725 | 8.063395081 | 0.12573932 | 0.686106127 |
| HS_209.1 | 9.722807531 | 7.452035274 | 8.324180547 | 6.964629667 | 8.259507895 | 8.344073938 | 8.413204789 | 9.783898589 | 8.725877459 | 7.502235115 | 0.593139687 | 0.388886376 |
| HS_21 | 6.404290064 | 6.957102042 | 7.231701199 | 7.038918989 | 6.671010241 | 6.755555262 | 7.145677455 | 9.082149041 | 7.408117408 | 7.270996061 | 0.268195503 | 0.480914505 |
| HS_211 | 6.564530601 | 6.469234794 | 6.66106548 | 7.115303806 | 6.862947248 | 6.755555262 | 6.721782768 | 6.668175869 | 6.584962501 | 6.329123596 | 0.77570331 | -0.048775796 |
| HS_215 | 8.244125943 | 7.192785959 | 6.75087444 | 6.731997787 | 6.671010241 | 6.609548139 | 6.930145948 | 9.403012024 | 6.584962501 | 6.45779126 | 0.841420329 | -0.120534347 |
| HS_216 | 6.768184325 | 6.273329387 | 6.318316841 | 6.421223299 | 9.570614722 | 8.25502857 | 8.986979709 | 8.566054038 | 9.692266772 | 7.712870868 | 0.000354462 | 2.352038983 |
| HS_217 | 7.625708843 | 7.056366761 | 6.05202456 | 5.987548259 | 7.982993575 | 8.15810472 | 12.58282411 | 11.52645033 | 12.46867495 | 11.2444233 | 0.003898504 | 3.980166393 |
| HS_218 | 6.653919873 | 6.14363831 | 6.224966365 | 6.404290064 | 7.273795599 | 7.117383367 | 8.514911265 | 7.596935142 | 7.408117408 | 6.714245518 | 0.005698132 | 1.080861063 |
| HS_219 | 7.77280941 | 7.836681593 | 7.73470962 | 7.836050355 | 9.14822234 | 9.024308597 | 9.821455119 | 9.339181934 | 9.456765088 | 8.986979709 | 5.54E-05 | 1.501089387 |
| HS_22.1 | 11.75768169 | 10.09882166 | 10.93199324 | 11.21249639 | 9.14822234 | 9.265146182 | 9.57799522 | 9.05853297 | 8.597307691 | 9.451211112 | 0.008194538 | -1.81717899 |
| HS_220 | 6.762880293 | 6.292781749 | 6.469234794 | 6.653203362 | 7.273795599 | 7.316960287 | 6.930145948 | 6.790511412 | 6.584962501 | 12.89026428 | 0.212579947 | 1.419914955 |
| HS_221 | 7.964052011 | 6.122051448 | 5.875288598 | 5.790511412 | 5.905687849 | 6.196725061 | 6.356672044 | 5.942514505 | 6.250772132 | 5.92243574 | 0.555292358 | -0.342174645 |
| HS_228.1 | 8.097505456 | 8.478971805 | 9.570614722 | 7.943686952 | 6.274261661 | 12.28004576 | 6.089582893 | 13.18780053 | 14.01553701 | 13.12028648 | 0.186383379 | 2.305224323 |
| HS_23 | 6.716990894 | 6.95070169 | 7.199672345 | 7.293701542 | 7.484218708 | 7.75087444 | 7.145677455 | 7.388878339 | 12.78426676 | 7.799605422 | 0.188243906 | 1.351986904 |
| HS_231 | 11.03981011 | 6.697662633 | 6.175923742 | 6.389738879 | 6.399171094 | 8.950993232 | 6.721782768 | 10.98463207 | 6.83541884 | 6.45779126 | 0.918095118 | 0.149181036 |
| HS_232 | 6.667466405 | 6.224966365 | 8.959132577 | 6.849248703 | 7.075746538 | 6.755555262 | 7.599168993 | 7.661778098 | 7.408117408 | 6.714245518 | 0.967999889 | 0.02723179 |
| HS_239 | 13.07437565 | 10.22363975 | 10.2701785 | 11.1508262 | 13.74706006 | 13.19746245 | 13.45752196 | 13.90966811 | 13.84325371 | 12.6077233 | 0.036387441 | 2.280693241 |
| HS_24 | 6.636624621 | 6.476543706 | 6.350497247 | 7.623515741 | 7.641690523 | 7.75087444 | 7.78496195 | 7.515305605 | 7.864805194 | 7.625708843 | 0.047379782 | 0.925429097 |
| HS_240 | 7.256444596 | 6.089582893 | 6.36018867 | 6.389738879 | 7.273795599 | 6.609548139 | 6.523561956 | 11.23595438 | 7.408117408 | 6.329123596 | 0.239309543 | 1.03936142 |
| HS_241.1 | 7.837312556 | 7.505414609 | 8.392961339 | 7.227856573 | 6.399171094 | 7.316960287 | 7.599168993 | 7.661778098 | 7.864805194 | 6.714245518 | 0.203713092 | -0.481531405 |
| HS_242 | 6.523561956 | 7.141596278 | 9.00365845 | 8.189330401 | 13.43564422 | 12.67185062 | 12.6077233 | 6.261154673 | 6.584962501 | 11.89958351 | 0.089801565 | 2.862283033 |
| HS_243.1 | 12.67185062 | 10.64925618 | 11.50858711 | 10.45491614 | 7.273795599 | 7.501040996 | 6.721782768 | 6.543805176 | 6.411087227 | 7.502235115 | 0.001398097 | -4.328861366 |
| HS_244 | 9.796364138 | 9.967802645 | 9.824322351 | 10.61369739 | 13.73190236 | 10.9918761 | 13.80583454 | 13.19746245 | 11.95728346 | 11.49355527 | 0.002860756 | 2.47910573 |
| HS_25 | 8.502235115 | 8.15936681 | 7.913487841 | 8.568716189 | 10.09882166 | 9.924812504 | 10.89231512 | 10.42500604 | 10.52209107 | 9.160249616 | 0.000214081 | 1.884597848 |
| HS_250 | 11.26982798 | 8.613973587 | 9.334385031 | 7.669593751 | 6.671010241 | 6.951867504 | 6.523561956 | 6.543805176 | 7.408117408 | 6.45779126 | 0.045694642 | -2.462586163 |
| HS_251.1 | 6.352264173 | 6.74281467 | 6.578938713 | 7.100136671 | 7.982993575 | 6.951867504 | 6.207502459 | 6.543805176 | 6.250772132 | 10.92577695 | 0.343919646 | 0.783581075 |
| HS_252.1 | 8.106955457 | 7.352264173 | 7.421223299 | 8.117902789 | 8.151523994 | 8.25502857 | 8.413204789 | 8.326204751 | 8.300123725 | 7.502235115 | 0.158336859 | 0.408467061 |
| HS_253 | 6.168922782 | 6.373300197 | 6.302867361 | 6.365447589 | 7.641690523 | 7.501040996 | 8.002252452 | 7.388878339 | 8.11997861 | 7.925109324 | 1.81E-05 | 1.460523892 |
| HS_254 | 9.096188046 | 6.520422249 | 6.953032377 | 6.399171094 | 6.155830172 | 6.465974465 | 6.523561956 | 6.261154673 | 6.250772132 | 6.714245518 | 0.271580154 | -0.846946956 |
| HS_255 | 7.116863758 | 6.930145948 | 6.392317423 | 6.731997787 | 8.800252806 | 7.625708843 | 9.082149041 | 9.014299495 | 9.456765088 | 7.502235115 | 0.001931811 | 1.787403836 |
| HS_257 | 6.768184325 | 7.469234794 | 7.588714636 | 6.004501392 | 11.65745244 | 6.755555262 | 7.400025518 | 6.422905743 | 7.117902789 | 6.329123596 | 0.491927439 | 0.656168771 |
| HS_258 | 6.029011087 | 6.122051448 | 6.199672345 | 6.276124405 | 6.05202456 | 6.755555262 | 6.207502459 | 6.332707934 | 6.411087227 | 6.329123596 | 0.12309214 | 0.191285352 |
| HS_26.1 | 6.836050355 | 6.350497247 | 6.36719631 | 6.653203362 | 7.273795599 | 7.117383367 | 8.413204789 | 7.23935985 | 8.838573653 | 6.100136671 | 0.06621227 | 0.945338836 |
| HS_260 | 7.66035251 | 7.883254231 | 9.318768743 | 7.433794059 | 7.075746538 | 6.755555262 | 6.721782768 | 6.790511412 | 6.83541884 | 7.502235115 | 0.072086039 | -1.127167396 |
| HS_261.1 | 10.48290877 | 6.165911939 | 6.372429792 | 6.663913842 | 6.05202456 | 5.992088609 | 6.207502459 | 11.18090371 | 11.70111149 | 6.001126665 | 0.78388167 | 0.43450183 |
| HS_262.1 | 8.493054842 | 8.248876343 | 8.409390936 | 8.366322214 | 7.484218708 | 9.265146182 | 7.400025518 | 6.543805176 | 7.117902789 | 8.063395081 | 0.113283317 | -0.733662175 |
| HS_263.1 | 11.44403133 | 11.62858224 | 10.51914479 | 11.02873489 | 6.399171094 | 6.092757141 | 6.930145948 | 10.78119576 | 6.250772132 | 6.45779126 | 0.002024597 | -4.003151089 |
| HS_264.1 | 6.411087227 | 5.927185358 | 6 | 6.155830172 | 6.399171094 | 6.609548139 | 6.523561956 | 6.668175869 | 6.83541884 | 5.92243574 | 0.05847289 | 0.369526251 |
| HS_265.1 | 9.366977836 | 7.310158318 | 7.664625055 | 8.23457796 | 7.799605422 | 11.52645033 | 11.66540254 | 9.889656058 | 8.300123725 | 7.712870868 | 0.162988987 | 1.338266699 |
| HS_266.1 | 6.582706527 | 6.8899602 | 7.898752526 | 6.171927354 | 6.274261661 | 6.092757141 | 6.089582893 | 11.20939244 | 5.888743249 | 6.714245518 | 0.867591067 | 0.158993832 |
| HS_267 | 6.187846909 | 6.575161033 | 6.428778891 | 6.64096791 | 9.570614722 | 9.024308597 | 9.507001733 | 10.31537603 | 9.456765088 | 8.405566974 | 2.89E-05 | 2.921750171 |
| HS_268 | 7.883254231 | 8.144658243 | 8.548436625 | 8.245790363 | 7.799605422 | 7.316960287 | 7.599168993 | 7.388878339 | 7.635173947 | 9.082149041 | 0.219726602 | -0.40187886 |
| HS_269 | 7.070389328 | 7.371558863 | 6.66106548 | 6.759555343 | 10.40801123 | 9.537703748 | 10.71896091 | 8.689299161 | 10.67410397 | 9.824322351 | 6.36E-05 | 3.009757976 |
| HS_27 | 8.026246773 | 8.275193334 | 7.906289347 | 8.053654418 | 10.5844928 | 10.14095755 | 10.68088692 | 10.03589879 | 10.54515755 | 10.32940394 | 1.10E-07 | 2.320786957 |
| HS_273 | 6.244125943 | 6.422905743 | 6.451211112 | 6.512542955 | 6.862947248 | 6.465974465 | 6.523561956 | 6.668175869 | 7.117902789 | 6.714245518 | 0.024200531 | 0.317771536 |
| HS_275 | 9.996897126 | 10.56367296 | 10.22363975 | 10.44159552 | 10.90914305 | 10.45491614 | 10.8622499 | 10.09354961 | 10.8591465 | 10.71020375 | 0.09631608 | 0.341750152 |
| HS_276.1 | 9.730979533 | 8.368506462 | 9.893756568 | 8.812498225 | 12.90809234 | 12.25017997 | 13.18780053 | 11.51491126 | 12.93049711 | 11.68877483 | 0.000347323 | 3.21194081 |
| HS_278 | 8.168922782 | 5.876516947 | 6.559950613 | 5.987548259 | 7.075746538 | 6.609548139 | 6.721782768 | 7.388878339 | 6.83541884 | 6.329123596 | 0.763852524 | 0.178515053 |
| HS_279_a | 7.621319301 | 8.106955457 | 8.003096215 | 8.539158811 | 10.68088692 | 10.02236781 | 10.65391987 | 9.66035251 | 10.71020375 | 9.616089314 | 7.05E-05 | 2.156337584 |
| HS_280_a | 6.139551352 | 7.256444596 | 7.084276911 | 6.421223299 | 7.075746538 | 6.951867504 | 7.145677455 | 7.1017131 | 6.83541884 | 6.569855608 | 0.477024347 | 0.221339135 |
| HS_280_b | 9.474719947 | 6.836050355 | 7.951867504 | 6.255500733 | 12.08470875 | 6.316507819 | 6.523561956 | 12.45213826 | 6.411087227 | 6.569855608 | 0.605635855 | 0.763441969 |
| HS_282 | 6.937815169 | 7.09117089 | 8.607700188 | 6.707359132 | 9.412569847 | 9.101450482 | 9.650154214 | 9.996897126 | 9.692266772 | 7.712870868 | 0.01135527 | 1.92502354 |
| HS_283_a | 7.318316841 | 6.861707287 | 6.807354922 | 7.14363831 | 7.641690523 | 7.625708843 | 8.289557848 | 8.405566974 | 7.408117408 | 8.324180547 | 0.002887063 | 0.916382684 |
| HS_283_b | 6.059614856 | 6.937815169 | 6.027905997 | 6.078951341 | 5.837943242 | 5.716990894 | 5.87282876 | 5.752213368 | 5.888743249 | 6.001126665 | 0.144713076 | -0.431097478 |
| HS_284 | 5.790511412 | 5.867896464 | 5.988684687 | 5.987548259 | 7.075746538 | 7.117383367 | 7.78496195 | 6.953032377 | 7.635173947 | 6.569855608 | 0.000652252 | 1.280698759 |
| HS_284.1 | 10.79075575 | 10.41309898 | 10.67410397 | 9.760553633 | 9.32395546 | 8.60936345 | 9.258330473 | 8.452035274 | 9.207990433 | 8.689299161 | 0.002100568 | -1.486132377 |
| HS_285 | 6.911691582 | 7.628627802 | 7.275193334 | 8.003939486 | 7.982993575 | 8.15810472 | 7.78496195 | 7.927777962 | 9.574309691 | 7.625708843 | 0.088943768 | 0.720779739 |
| HS_286_a | 5.898450233 | 6.292781749 | 6.132371199 | 6.029011087 | 6.05202456 | 6.196725061 | 6.356672044 | 6.543805176 | 6.10433666 | 6.100136671 | 0.265041047 | 0.137463129 |
| HS_287 | 7.815703503 | 7.591709333 | 6.995484519 | 6.663913842 | 12.1027959 | 6.755555262 | 6.089582893 | 9.688862232 | 6.411087227 | 6.714245518 | 0.522781521 | 0.693652039 |
| HS_29 | 11.35980446 | 13.3303147 | 13.03414554 | 11.18090371 | 7.641690523 | 7.501040996 | 8.167418146 | 7.23935985 | 8.453682186 | 6.569855608 | 0.00109219 | -4.630784217 |
| HS_3 | 7.528258743 | 7.006746832 | 5.82527683 | 5.861707287 | 5.980710829 | 5.992088609 | 6.207502459 | 6.0725346 | 5.9795681 | 5.842978832 | 0.291558003 | -0.542933518 |
| HS_30 | 6.215290306 | 5.966937979 | 5.988684687 | 6.029011087 | 6.862947248 | 6.196725061 | 6.721782768 | 6.953032377 | 6.83541884 | 6.714245518 | 0.000958394 | 0.664044288 |
| HS_303_a | 8.618385502 | 9.631540867 | 9.353587949 | 8.816343705 | 9.32395546 | 11.01164658 | 9.57799522 | 9.726899318 | 7.864805194 | 11.06709819 | 0.263048549 | 0.657102154 |
| HS_303_b | 8.564340058 | 8.344073938 | 7.985272714 | 9.480992104 | 8.381110294 | 8.894817763 | 8.607700188 | 8.203837384 | 8.597307691 | 7.712870868 | 0.614635691 | -0.194062339 |
| HS_304_a | 8.675957033 | 7.533719071 | 7.790511412 | 7.433794059 | 8.381110294 | 10.50476934 | 8.413204789 | 7.661778098 | 13.45752196 | 7.925109324 | 0.15999648 | 1.532086908 |
| HS_304_b | 6.476543706 | 6.385431037 | 6.404290064 | 6.54225805 | 7.075746538 | 7.625708843 | 7.400025518 | 6.953032377 | 11.02517418 | 7.270996061 | 0.072403958 | 1.439649871 |
| HS_305_b | 7.837312556 | 6.309248961 | 8.821773982 | 6.053111336 | 6.399171094 | 6.465974465 | 6.523561956 | 6.422905743 | 6.411087227 | 6.194756854 | 0.283601235 | -0.852452152 |
| HS_31.1 | 9.191059215 | 9.276589716 | 8.574025798 | 7.987548259 | 12.96155822 | 8.894817763 | 9.77016832 | 13.35328469 | 9.456765088 | 9.202491187 | 0.075900642 | 1.849208464 |
| HS_32 | 9.827660269 | 10.44159552 | 10.09882166 | 9.258330473 | 10.56367296 | 10.20371505 | 10.56985561 | 9.567385727 | 10.48290877 | 13.30457949 | 0.178026952 | 0.87541762 |
| HS_33 | 9.958697703 | 7.432959407 | 7.542644987 | 8.416164165 | 10.81382127 | 10.1096348 | 11.40375769 | 11.1508262 | 10.9544508 | 9.846430632 | 0.019093065 | 2.375536997 |
| HS_35 | 6.270528942 | 6.663202279 | 6.010108453 | 6.105384749 | 6.399171094 | 6.196725061 | 6.089582893 | 6.790511412 | 6.10433666 | 6.194756854 | 0.858509032 | 0.033541223 |
| HS_36.1 | 6.396604781 | 6.517275693 | 6.547665748 | 6.776762002 | 8.68580002 | 6.465974465 | 6.721782768 | 7.857047916 | 7.635173947 | 6.714245518 | 0.075648565 | 0.787093716 |
| HS_37 | 6.28169825 | 7.029563314 | 6.469234794 | 8.763876285 | 7.075746538 | 6.609548139 | 7.78496195 | 6.953032377 | 6.83541884 | 7.502235115 | 0.988353481 | -0.009269334 |
| HS_38.1 | 8.633721813 | 8.763876285 | 8.977852308 | 8.964052011 | 10.96079838 | 10.44935502 | 11.16804527 | 10.1572206 | 11.22427306 | 10.68088692 | 2.00E-05 | 1.938554271 |
| HS_4.1 | 6.292781749 | 6.301953395 | 6.483009577 | 6.570614722 | 7.641690523 | 7.316960287 | 6.721782768 | 7.388878339 | 7.635173947 | 7.625708843 | 0.000523086 | 0.976275924 |
| HS_40 | 8.173926932 | 8.574025798 | 8.409390936 | 8.806066226 | 9.792139552 | 9.393819448 | 10.17816685 | 9.339181934 | 9.692266772 | 8.910492832 | 0.001420698 | 1.060158759 |
| HS_41 | 5.944858446 | 6 | 6.091699834 | 6.300123725 | 6.530601415 | 6.465974465 | 6.523561956 | 6.790511412 | 6.411087227 | 6.329123596 | 0.004597413 | 0.424306177 |
| HS_42 | 7.190812368 | 6.373300197 | 6.502235115 | 6.682292371 | 6.399171094 | 6.609548139 | 6.721782768 | 7.388878339 | 7.635173947 | 6.569855608 | 0.483049469 | 0.200241636 |
| HS_43.1 | 6.177917792 | 6.623515741 | 6.559950613 | 6.682292371 | 6.862947248 | 6.609548139 | 7.145677455 | 6.332707934 | 6.584962501 | 6.714245518 | 0.255122462 | 0.197429003 |
| HS_44.1 | 6.548436625 | 5.980710829 | 6.329123596 | 6.053111336 | 6.399171094 | 5.911691582 | 6.356672044 | 5.988684687 | 5.9795681 | 7.270996061 | 0.724479491 | 0.089951665 |
| HS_45.1 | 8.975274788 | 6.575161033 | 6.719731057 | 6.857980995 | 9.248164779 | 8.894817763 | 9.40779885 | 11.71334355 | 9.328338334 | 8.75788999 | 0.018057784 | 2.276355243 |
| HS_46 | 6.433794059 | 6.410239331 | 6.154818109 | 6.276124405 | 7.273795599 | 6.755555262 | 8.002252452 | 6.790511412 | 7.408117408 | 7.39145842 | 0.002926977 | 0.951537783 |
| HS_47 | 7.655709596 | 7.700786478 | 7.533719071 | 7.906289347 | 8.259507895 | 7.75087444 | 8.167418146 | 8.647638302 | 7.864805194 | 8.248876343 | 0.017766435 | 0.45739393 |
| HS_48.1 | 7.162894799 | 7.416164165 | 7.282625134 | 7.623515741 | 8.800252806 | 8.60936345 | 9.507001733 | 8.405566974 | 9.692266772 | 8.063395081 | 0.00152152 | 1.475007843 |
| HS_49 | 7.190812368 | 7.315602457 | 7.225930411 | 7.666046429 | 7.273795599 | 7.117383367 | 6.721782768 | 7.515305605 | 6.584962501 | 7.006746832 | 0.116776501 | -0.312935137 |
| HS_5.1 | 6.917670434 | 6.365447589 | 6.05202456 | 6.105384749 | 6.671010241 | 7.117383367 | 7.400025518 | 7.1017131 | 7.864805194 | 6.329123596 | 0.041575514 | 0.720545003 |
| HS_50 | 6.510961919 | 7.310158318 | 6.483009577 | 6.912290583 | 6.399171094 | 6.609548139 | 6.721782768 | 6.790511412 | 6.83541884 | 6.569855608 | 0.51057474 | -0.149723789 |
| HS_51 | 6.782670659 | 6.8899602 | 7.384567923 | 7.115303806 | 7.484218708 | 7.316960287 | 7.599168993 | 6.332707934 | 6.584962501 | 9.082149041 | 0.424667144 | 0.356902264 |
| HS_52 | 11.32187174 | 9.796364138 | 9.846430632 | 10.4268937 | 13.80583454 | 12.65244168 | 12.96155822 | 13.00499288 | 12.65244168 | 13.36342298 | 0.001425733 | 2.725558611 |
| HS_53 | 5.988684687 | 6.008988783 | 6.529820947 | 6.029011087 | 6.274261661 | 6.092757141 | 6.356672044 | 6.0725346 | 6.411087227 | 6.001126665 | 0.691548331 | 0.062280181 |
| HS_54 | 7.011227255 | 7.815703503 | 6.897240426 | 7.017365205 | 6.274261661 | 6.196725061 | 6.721782768 | 5.988684687 | 12.90809234 | 6.194756854 | 0.86909552 | 0.195333131 |
| HS_55 | 6.691394579 | 6.365447589 | 8.026246773 | 5.94016675 | 6.05202456 | 6.092757141 | 5.927185358 | 6.168922782 | 6.10433666 | 6.329123596 | 0.248814669 | -0.64342224 |
| HS_55.1 | 6.952450058 | 6.318316841 | 5.930737338 | 5.910492832 | 6.155830172 | 6.196725061 | 6.207502459 | 6.261154673 | 6.250772132 | 6.45779126 | 0.931368248 | -0.023036641 |
| HS_56 | 7.183883459 | 6.165911939 | 9.462297766 | 6.411087227 | 7.799605422 | 7.625708843 | 8.986979709 | 8.647638302 | 8.597307691 | 7.39145842 | 0.340130526 | 0.868987967 |
| HS_57.1 | 6.952450058 | 6.571373436 | 6.613236955 | 6.951867504 | 6.862947248 | 6.755555262 | 7.78496195 | 7.515305605 | 6.83541884 | 6.329123596 | 0.352251353 | 0.241653429 |
| HS_58 | 6.100136671 | 6.454504938 | 6.6395216 | 6.512542955 | 6.155830172 | 5.992088609 | 6.207502459 | 5.902073579 | 5.9795681 | 6.100136671 | 0.040831319 | -0.370476609 |
| HS_59 | 6.053111336 | 6.872213144 | 6.211401637 | 6.338959175 | 8.607700188 | 7.501040996 | 9.821455119 | 7.661778098 | 7.864805194 | 7.502235115 | 0.00347403 | 1.790914462 |
| HS_6 | 7.761551232 | 8.156083076 | 8.008988783 | 6.759555343 | 6.862947248 | 6.951867504 | 7.145677455 | 6.953032377 | 6.83541884 | 6.714245518 | 0.092161313 | -0.761013118 |
| HS_60 | 7.083213368 | 8.6387979 | 7.993221467 | 9.347953493 | 7.982993575 | 9.226773423 | 6.523561956 | 6.668175869 | 7.117902789 | 7.799605422 | 0.298688051 | -0.712627718 |
| HS_61 | 5.815063017 | 5.744161096 | 5.875288598 | 5.861707287 | 5.905687849 | 5.911691582 | 5.988684687 | 5.942514505 | 5.888743249 | 5.675251386 | 0.284052941 | 0.061373877 |
| HS_62 | 7.302867361 | 7.290940402 | 7.06501215 | 7.461070114 | 9.049167872 | 7.75087444 | 8.607700188 | 8.003096215 | 8.11997861 | 7.925109324 | 0.00352414 | 0.962681935 |
| HS_63 | 6.754887502 | 6.773468928 | 6.716990894 | 6.964629667 | 6.671010241 | 7.117383367 | 6.930145948 | 7.23935985 | 6.83541884 | 7.006746832 | 0.137888414 | 0.164183265 |
| HS_64 | 7.032321287 | 6.469234794 | 6.194756854 | 6.45779126 | 6.399171094 | 6.609548139 | 6.930145948 | 6.790511412 | 6.83541884 | 6.45779126 | 0.535995372 | 0.131905067 |
| HS_65 | 7.695228291 | 8.384567923 | 10.44935502 | 10.78119576 | 12.58282411 | 9.49645436 | 10.39687232 | 8.670656249 | 13.10212335 | 10.44935502 | 0.202355852 | 1.455460818 |
| HS_66 | 5.857980995 | 5.980710829 | 5.875288598 | 6.029011087 | 6.399171094 | 6.755555262 | 6.356672044 | 6.332707934 | 6.411087227 | 6.100136671 | 0.002045806 | 0.456807161 |
| HS_67 | 5.984133595 | 6.36018867 | 8.073606041 | 6.421223299 | 7.075746538 | 6.951867504 | 6.930145948 | 7.23935985 | 6.584962501 | 6.714245518 | 0.691098633 | 0.206266742 |
| HS_68 | 8.470048726 | 7.925109324 | 7.00337736 | 7.235535607 | 9.52277767 | 9.177419538 | 10.4943056 | 10.19204618 | 9.958697703 | 10.60385812 | 0.00141169 | 2.332999714 |
| HS_69 | 8.346513733 | 5.815063017 | 5.760220946 | 5.94016675 | 6.05202456 | 6.092757141 | 6.207502459 | 6.422905743 | 5.9795681 | 5.92243574 | 0.615081813 | -0.352625488 |
| HS_7 | 10.12399203 | 6.545350645 | 6.597680144 | 7.55688919 | 9.32395546 | 8.950993232 | 11.11155955 | 12.19614103 | 9.207990433 | 7.502235115 | 0.108376912 | 2.009501133 |
| HS_70 | 6.112700133 | 5.988684687 | 5.875288598 | 6.004501392 | 6.05202456 | 5.992088609 | 6.207502459 | 6.0725346 | 6.250772132 | 5.92243574 | 0.251258252 | 0.087599314 |
| HS_71.1 | 9.58458675 | 9.09037711 | 8.910492832 | 9.271929844 | 10.93199324 | 10.56985561 | 11.54099979 | 10.80727441 | 11.04678297 | 10.04651016 | 0.000203807 | 1.609556061 |
| HS_72 | 8.643495471 | 7.362820526 | 8.106955457 | 6.431288654 | 7.273795599 | 6.465974465 | 7.78496195 | 6.953032377 | 6.411087227 | 6.857980995 | 0.2625092 | -0.678334591 |
| HS_73.1 | 6.465974465 | 6.754219433 | 6.584962501 | 6.83541884 | 7.273795599 | 7.75087444 | 7.145677455 | 6.953032377 | 7.408117408 | 7.39145842 | 0.001397103 | 0.660348807 |
| HS_74 | 8.300123725 | 7.753885283 | 6.43629512 | 6.431288654 | 6.862947248 | 7.117383367 | 7.78496195 | 7.23935985 | 7.408117408 | 6.714245518 | 0.936434178 | -0.042562305 |
| HS_75.1 | 6.972692654 | 8.626439137 | 8.244125943 | 7.136478518 | 7.273795599 | 7.117383367 | 7.145677455 | 7.515305605 | 6.83541884 | 7.141596278 | 0.255495104 | -0.573404539 |
| HS_76 | 9.14822234 | 6.251719093 | 6 | 5.87774425 | 6.399171094 | 6.316507819 | 6.721782768 | 6.422905743 | 7.117902789 | 6.329123596 | 0.755738701 | -0.268189119 |
| HS_77 | 6.599912842 | 6.209453366 | 5.965784285 | 6.136478518 | 7.075746538 | 6.755555262 | 7.400025518 | 6.790511412 | 9.098821663 | 6.714245518 | 0.033837815 | 1.077910399 |
| HS_78 | 9.638435914 | 8.510170751 | 8.453682186 | 7.77939106 | 10.21188829 | 9.315715658 | 10.52209107 | 9.474719947 | 10.52209107 | 9.116863758 | 0.036642159 | 1.265141655 |
| HS_79.1 | 7.687900522 | 6.74819285 | 8.227856573 | 7.017365205 | 11.44403133 | 6.951867504 | 7.599168993 | 7.388878339 | 7.117902789 | 7.141596278 | 0.527310599 | 0.520245417 |
| HS_8 | 7.176422513 | 6.762880293 | 6.815063017 | 6.759555343 | 6.671010241 | 10.65391987 | 7.145677455 | 10.07614753 | 6.83541884 | 7.712870868 | 0.126068113 | 1.304027176 |
| HS_80 | 8.15936681 | 7.24697806 | 6.957682486 | 7.031770114 | 7.641690523 | 7.316960287 | 8.167418146 | 7.388878339 | 8.453682186 | 7.712870868 | 0.244805662 | 0.431300691 |
| HS_81 | 8.816343705 | 8.848935856 | 8.803162444 | 8.956666555 | 10.40801123 | 10.04651016 | 10.60385812 | 9.741635516 | 10.48290877 | 9.97053734 | 0.000114139 | 1.352633049 |
| HS_82.1 | 5.898450233 | 5.988684687 | 6.05202456 | 6.105384749 | 6.671010241 | 6.465974465 | 6.523561956 | 6.543805176 | 6.584962501 | 6.194756854 | 0.000323219 | 0.486209141 |
| HS_83.1 | 6.201633861 | 6.58044702 | 6.05202456 | 6.105384749 | 8.800252806 | 8.60936345 | 9.32395546 | 8.25502857 | 9.098821663 | 6.714245518 | 0.001471097 | 2.23207203 |
| HS_84 | 6.673839056 | 6.789859641 | 6.789859641 | 7.062855655 | 8.151523994 | 7.316960287 | 8.002252452 | 7.596935142 | 8.11997861 | 6.714245518 | 0.014460922 | 0.821212502 |
| HS_85.1 | 6.040015679 | 6.14363831 | 6.132371199 | 6.171927354 | 6.05202456 | 6.092757141 | 6.089582893 | 6.0725346 | 5.9795681 | 6.329123596 | 0.739351619 | -0.019389654 |
| HS_86 | 8.194264383 | 7.685099172 | 7.257859235 | 7.519243094 | 9.570614722 | 8.521207816 | 9.650154214 | 9.595443985 | 9.456765088 | 9.202491187 | 0.000398589 | 1.668663031 |
| HS_87 | 8.168922782 | 6.053111336 | 7.499845887 | 5.894817763 | 7.273795599 | 6.609548139 | 7.78496195 | 7.23935985 | 6.411087227 | 6.569855608 | 0.903281095 | 0.077260287 |
| HS_88 | 6.325530332 | 6.697662633 | 6.529820947 | 6.794415866 | 6.530601415 | 6.755555262 | 6.207502459 | 6.168922782 | 6.411087227 | 7.006746832 | 0.672708471 | -0.073454782 |
| HS_89 | 10.74306722 | 6.545350645 | 7.566054038 | 6.881419908 | 8.68580002 | 7.921245889 | 7.145677455 | 13.60579201 | 8.300123725 | 7.006746832 | 0.560749262 | 0.843591368 |
| HS_9 | 8.574025798 | 6.038918989 | 6.268658955 | 6.707359132 | 5.980710829 | 5.992088609 | 6.207502459 | 11.68877483 | 11.66540254 | 6.100136671 | 0.454309897 | 1.041861938 |
| HS_90 | 7.511752654 | 7.641690523 | 7.628627802 | 8.132885254 | 8.381110294 | 7.921245889 | 8.889199726 | 8.956666555 | 8.597307691 | 7.712870868 | 0.026107283 | 0.680994446 |
| HS_91.1 | 10.01680829 | 9.480992104 | 10.59395128 | 10.4871366 | 6.05202456 | 6.092757141 | 6.356672044 | 5.988684687 | 6.10433666 | 6.100136671 | 0.000370315 | -4.028953441 |
| HS_92 | 6.374170076 | 6.558420713 | 6.676662335 | 7.017365205 | 6.530601415 | 6.755555262 | 6.523561956 | 6.543805176 | 6.83541884 | 7.006746832 | 0.797861374 | 0.042626998 |
| HS_93 | 6.661778098 | 6.678071905 | 6.736740155 | 7.046578367 | 8.893756568 | 9.101450482 | 10.11517373 | 9.541677449 | 10.50809187 | 8.589838376 | 0.000167168 | 2.677539282 |
| HS_94 | 8.763876285 | 9.857825524 | 9.676662335 | 9.014299495 | 10.30628959 | 11.139935 | 11.72144102 | 8.60936345 | 12.21577565 | 11.2062818 | 0.033141537 | 1.538348508 |
| HS_95 | 6.564530601 | 6.251719093 | 6.404290064 | 6.529820947 | 8.381110294 | 8.15810472 | 7.400025518 | 8.203837384 | 8.959132577 | 8.75788999 | 0.000209057 | 1.872426571 |
| HS_96 | 12.04668067 | 11.95728346 | 11.72144102 | 11.77020962 | 12.16091137 | 11.59577031 | 12.41280798 | 12.14063808 | 12.50061784 | 11.71334355 | 0.242783571 | 0.213444496 |
| HS_97 | 10.04916787 | 9.474719947 | 9.451211112 | 10.24257869 | 11.14539087 | 10.61966977 | 11.36380623 | 10.8560364 | 11.28952903 | 10.10145048 | 0.005358623 | 1.091561061 |
| HS_99.1 | 7.726558779 | 7.064473329 | 7.117383367 | 7.343407822 | 9.939285356 | 9.315715658 | 10.54515755 | 8.486231678 | 10.5844928 | 8.689299161 | 0.000964025 | 2.280407876 |
| hsa-let-7a | 14.51607549 | 14.51607549 | 14.51607549 | 14.61467534 | 7.273795599 | 12.45213826 | 6.930145948 | 11.75768169 | 7.635173947 | 11.33220442 | 0.004851603 | -4.977202143 |
| hsa-let-7a* | 6.105384749 | 6.0725346 | 6.010108453 | 6.196725061 | 6.862947248 | 6.316507819 | 6.523561956 | 6.332707934 | 11.72970571 | 6.194756854 | 0.223622545 | 1.230509704 |
| hsa-let-7b | 13.68986697 | 13.74706006 | 13.94706157 | 14.01553701 | 12.67185062 | 13.00499288 | 12.3835693 | 10.82033854 | 6.584962501 | 12.3835693 | 0.050629113 | -2.541667544 |
| hsa-let-7b* | 11.49355527 | 10.82631014 | 11.88542947 | 11.849366 | 12.36194377 | 13.63051741 | 11.48316077 | 12.93049711 | 8.11997861 | 7.141596278 | 0.631476867 | -0.569049559 |
| hsa-let-7c | 13.15864124 | 12.94553528 | 13.15864124 | 13.43564422 | 9.857825524 | 9.595443985 | 10.11517373 | 9.435878577 | 9.574309691 | 8.75788999 | 4.00E-07 | -3.61852858 |
| hsa-let-7c* | 6.707359132 | 7.836681593 | 7.985272714 | 6.255500733 | 6.671010241 | 6.755555262 | 11.23595438 | 8.003096215 | 6.584962501 | 6.857980995 | 0.584136994 | 0.488556389 |
| hsa-let-7d | 13.24425976 | 13.45752196 | 13.43564422 | 13.59243368 | 6.399171094 | 11.62858224 | 6.356672044 | 6.0725346 | 6.250772132 | 6.001126665 | 0.000894331 | -6.314321776 |
| hsa-let-7d* | 12.1027959 | 10.45491614 | 11.34318572 | 11.80779765 | 13.39700607 | 11.30691611 | 13.55601501 | 13.45752196 | 7.408117408 | 14.04598144 | 0.508363158 | 0.768085819 |
| hsa-let-7e | 13.65089467 | 13.60579201 | 13.45752196 | 13.3303147 | 10.00070427 | 12.03675744 | 10.39687232 | 9.783898589 | 10.21188829 | 9.403012024 | 0.000271127 | -3.205608678 |
| hsa-let-7e* | 8.670656249 | 8.68580002 | 7.829722735 | 7.933100475 | 10.70091649 | 10.33918193 | 10.94005661 | 9.638435914 | 10.98463207 | 9.082149041 | 0.00090014 | 2.001075473 |
| hsa-let-7f | 12.19614103 | 13.43564422 | 13.27035373 | 13.21598794 | 5.980710829 | 11.67330908 | 5.927185358 | 5.942514505 | 5.888743249 | 5.92243574 | 0.000938321 | -6.140381938 |
| hsa-let-7f-1* | 6.680886921 | 6.782670659 | 6.709428501 | 6.896029603 | 6.530601415 | 9.353587949 | 5.87282876 | 5.902073579 | 5.888743249 | 8.75788999 | 0.679801952 | 0.283700236 |
| hsa-let-7f-2* | 7.930441673 | 7.761551232 | 7.796364138 | 7.982993575 | 7.484218708 | 6.465974465 | 6.523561956 | 8.405566974 | 6.584962501 | 7.799605422 | 0.103663373 | -0.657189317 |
| hsa-let-7g | 13.76677734 | 14.37188552 | 14.41700199 | 14.41700199 | 7.799605422 | 12.33776126 | 8.514911265 | 11.77020962 | 8.453682186 | 11.52645033 | 0.003429458 | -4.176063364 |
| hsa-let-7g* | 8.812498225 | 6.880195729 | 7.77939106 | 7.074141463 | 7.075746538 | 6.755555262 | 6.356672044 | 6.422905743 | 6.83541884 | 7.270996061 | 0.144901396 | -0.850340871 |
| hsa-let-7i | 13.3303147 | 13.68986697 | 13.74706006 | 13.83329532 | 9.412569847 | 12.1027959 | 9.258330473 | 8.405566974 | 9.328338334 | 10.76088624 | 0.000749389 | -3.772052966 |
| hsa-let-7i* | 8.047669251 | 6.95070169 | 7.056366761 | 7.449561375 | 6.671010241 | 6.316507819 | 6.523561956 | 6.332707934 | 6.584962501 | 6.714245518 | 0.03677954 | -0.852242108 |
| hsa-miR-1 | 6.74281467 | 5.966937979 | 5.930737338 | 5.987548259 | 6.155830172 | 6.196725061 | 6.089582893 | 6.261154673 | 5.9795681 | 5.92243574 | 0.797651311 | -0.056126788 |
| hsa-miR-100 | 12.16091137 | 12.6077233 | 12.14063808 | 13.07437565 | 9.049167872 | 9.024308597 | 9.507001733 | 8.60936345 | 9.456765088 | 7.799605422 | 6.12E-06 | -3.588210072 |
| hsa-miR-100* | 7.365884968 | 7.726558779 | 7.5980525 | 8.033973543 | 6.862947248 | 7.316960287 | 7.599168993 | 7.23935985 | 7.408117408 | 7.006746832 | 0.044404617 | -0.442234011 |
| hsa-miR-101 | 8.830990481 | 10.57601187 | 8.626439137 | 10.80727441 | 9.969170641 | 9.852139328 | 10.2564446 | 9.403012024 | 10.1365426 | 9.52277767 | 0.817290084 | 0.146502167 |
| hsa-miR-101* | 6.139551352 | 7.352264173 | 8.450386479 | 7.532161085 | 7.273795599 | 6.609548139 | 6.721782768 | 6.668175869 | 6.411087227 | 6.569855608 | 0.261323061 | -0.659549904 |
| hsa-miR-103 | 10.98463207 | 11.1508262 | 10.96079838 | 10.8560364 | 8.607700188 | 12.12350713 | 10.04384754 | 9.62580015 | 10.027906 | 8.589838376 | 0.081807518 | -1.151640032 |
| hsa-miR-105 | 9.32395546 | 9.063395081 | 9.076281167 | 9.393819448 | 11.89958351 | 11.1028287 | 12.07271881 | 10.87198222 | 12.01300855 | 10.82033854 | 0.000106423 | 2.249047266 |
| hsa-miR-105* | 6.539158811 | 6.525129251 | 6.536830005 | 6.937815169 | 6.530601415 | 6.465974465 | 6.930145948 | 6.668175869 | 6.411087227 | 8.324180547 | 0.449245263 | 0.253627603 |
| hsa-miR-106a | 10.97763769 | 11.3810562 | 11.1508262 | 11.36779694 | 6.274261661 | 10.69156906 | 6.207502459 | 6.168922782 | 6.10433666 | 6.001126665 | 0.002185873 | -4.311376044 |
| hsa-miR-106a* | 6.860466259 | 6.898450233 | 6.211401637 | 6.348728154 | 7.982993575 | 6.951867504 | 8.002252452 | 8.106955457 | 8.11997861 | 7.799605422 | 0.001303084 | 1.247513933 |
| hsa-miR-106a:9.1 | 7.408117408 | 6.937815169 | 7.117383367 | 8.336283388 | 8.518849829 | 8.437544026 | 9.160249616 | 8.689299161 | 8.838573653 | 8.063395081 | 0.023636152 | 1.168085395 |
| hsa-miR-106b | 8.60936345 | 10.34751064 | 9.996897126 | 10.44935502 | 6.671010241 | 6.316507819 | 6.089582893 | 6.422905743 | 6.83541884 | 6.45779126 | 0.002905942 | -3.385245428 |
| hsa-miR-106b* | 10.42311591 | 9.676662335 | 8.964052011 | 8.773468928 | 7.799605422 | 7.501040996 | 7.145677455 | 6.953032377 | 7.635173947 | 7.712870868 | 0.008426132 | -2.001424619 |
| hsa-miR-107 | 8.506605116 | 9.342296946 | 7.48984796 | 8.413204789 | 8.68580002 | 8.60936345 | 8.889199726 | 8.15810472 | 8.838573653 | 6.569855608 | 0.787551623 | -0.146172507 |
| hsa-miR-10a | 10.14095755 | 10.8560364 | 11.62858224 | 11.1909358 | 6.530601415 | 6.609548139 | 6.721782768 | 6.668175869 | 6.83541884 | 6.329123596 | 0.000524116 | -4.338352892 |
| hsa-miR-10a* | 8.523953939 | 10.31955923 | 10.1321141 | 8.947783026 | 10.73538678 | 10.20371505 | 10.62561753 | 9.339181934 | 10.76088624 | 9.824322351 | 0.188160989 | 0.767332408 |
| hsa-miR-10b | 9.818582177 | 11.16804527 | 11.139935 | 11.03981011 | 6.274261661 | 6.196725061 | 6.356672044 | 6.790511412 | 6.584962501 | 6.100136671 | 0.000362235 | -4.407714914 |
| hsa-miR-10b* | 6.264911693 | 6.483815777 | 6.523561956 | 6.62935662 | 7.273795599 | 7.316960287 | 7.400025518 | 6.543805176 | 7.117902789 | 7.799605422 | 0.004539878 | 0.766604287 |
| hsa-miR-1178 | 5.984133595 | 6.133399125 | 6.211401637 | 6.365447589 | 6.274261661 | 6.465974465 | 6.207502459 | 6.261154673 | 6.411087227 | 11.62858224 | 0.296067375 | 1.034498301 |
| hsa-miR-1179 | 8.684047262 | 9.692266772 | 9.403012024 | 9.188712465 | 12.45213826 | 12.3835693 | 6.356672044 | 5.988684687 | 13.12847772 | 12.28004576 | 0.423080158 | 1.189588332 |
| hsa-miR-1180 | 8.843292973 | 7.700786478 | 8.651769271 | 8.30560579 | 9.049167872 | 10.04651016 | 12.36194377 | 11.82137539 | 10.8591465 | 7.502235115 | 0.050195406 | 1.89803284 |
| hsa-miR-1181 | 10.53974043 | 10.8622499 | 8.894817763 | 10.12399203 | 13.67325386 | 11.95728346 | 7.145677455 | 12.21577565 | 6.411087227 | 9.044667331 | 0.981798168 | -0.030575867 |
| hsa-miR-1182 | 6.754887502 | 6.561478892 | 6.983563697 | 6.404290064 | 6.671010241 | 6.951867504 | 6.721782768 | 11.50109077 | 10.8591465 | 12.65244168 | 0.071591963 | 2.550168204 |
| hsa-miR-1183 | 7.282625134 | 6.768184325 | 7.071462363 | 6.255500733 | 13.3303147 | 13.40900634 | 13.70497352 | 11.67330908 | 12.75713995 | 12.01300855 | 5.06E-07 | 5.970182219 |
| hsa-miR-1184 | 7.810571635 | 8.232660757 | 9.024308597 | 10.50143915 | 11.06709819 | 8.819061399 | 13.67325386 | 11.79445648 | 9.456765088 | 12.21577565 | 0.042341873 | 2.278823411 |
| hsa-miR-1185 | 6.029011087 | 5.980710829 | 5.875288598 | 6.029011087 | 6.399171094 | 6.196725061 | 8.002252452 | 6.543805176 | 8.300123725 | 7.625708843 | 0.022715988 | 1.199458992 |
| hsa-miR-1197 | 8.155324229 | 7.424586226 | 7.213347282 | 10.31955923 | 14.09416843 | 8.521207816 | 12.179878 | 10.24257869 | 9.63499251 | 9.846430632 | 0.053118472 | 2.475005104 |
| hsa-miR-1200 | 7.484218708 | 6.417852515 | 6.45779126 | 6.473299871 | 6.530601415 | 7.501040996 | 6.523561956 | 6.422905743 | 6.83541884 | 7.006746832 | 0.768710834 | 0.095088708 |
| hsa-miR-1201 | 13.03414554 | 12.96155822 | 13.12847772 | 11.71334355 | 6.05202456 | 5.992088609 | 6.089582893 | 6.261154673 | 5.9795681 | 6.100136671 | 0.000235089 | -6.630288673 |
| hsa-miR-1202 | 6.849248703 | 6.417852515 | 6.44625623 | 6.682292371 | 6.862947248 | 6.609548139 | 6.721782768 | 7.1017131 | 7.635173947 | 7.006746832 | 0.062587349 | 0.390739551 |
| hsa-miR-1203 | 6.229299509 | 6.733354341 | 6.292781749 | 6.483815777 | 6.399171094 | 6.609548139 | 6.523561956 | 6.422905743 | 7.408117408 | 6.329123596 | 0.390839107 | 0.180591812 |
| hsa-miR-1204 | 8.896181011 | 8.816343705 | 8.567385727 | 8.440453944 | 10.65391987 | 10.41811614 | 11.43514934 | 10.45491614 | 10.92888844 | 10.64925618 | 4.37E-06 | 2.076616588 |
| hsa-miR-1205 | 7.357552005 | 7.651410526 | 7.985272714 | 7.850499414 | 8.893756568 | 9.024308597 | 9.258330473 | 10.69156906 | 9.328338334 | 9.779883475 | 0.000560537 | 1.78484742 |
| hsa-miR-1206 | 8.680535344 | 8.684047262 | 8.816343705 | 8.521207816 | 9.14822234 | 8.819061399 | 9.32395546 | 9.082149041 | 9.692266772 | 9.353587949 | 0.004106792 | 0.561006962 |
| hsa-miR-1207-3p | 6.422905743 | 6.43629512 | 6.82527683 | 5.87774425 | 12.69522829 | 6.465974465 | 5.988684687 | 6.168922782 | 6.250772132 | 6.569855608 | 0.413162771 | 0.966017508 |
| hsa-miR-1207-5p | 8.982708429 | 8.977852308 | 8.684047262 | 8.830990481 | 9.939285356 | 9.353587949 | 9.87175126 | 8.566054038 | 10.42500604 | 10.07961811 | 0.025246531 | 0.836984173 |
| hsa-miR-1208 | 7.609548139 | 7.522385366 | 7.411087227 | 7.756889855 | 10.06743436 | 9.56414949 | 10.08308037 | 9.11204848 | 10.31118066 | 9.202491187 | 5.22E-05 | 2.148419778 |
| hsa-miR-122 | 9.063395081 | 8.221103725 | 7.687900522 | 8.245790363 | 11.86546264 | 13.68986697 | 13.63051741 | 7.388878339 | 7.864805194 | 12.78426676 | 0.055082554 | 2.899418798 |
| hsa-miR-122* | 6.483815777 | 6.678071905 | 6.798309782 | 7.094869433 | 7.799605422 | 8.040837649 | 7.78496195 | 6.953032377 | 7.635173947 | 7.625708843 | 0.00222787 | 0.876119974 |
| hsa-miR-1224-3p | 9.541677449 | 10.62561753 | 9.70822173 | 9.768101599 | 10.61966977 | 10.16440416 | 10.94005661 | 10.28505538 | 10.76088624 | 10.32940394 | 0.082071174 | 0.605674773 |
| hsa-miR-1224-5p | 6.697662633 | 7.658211483 | 7.425845306 | 6.255500733 | 6.05202456 | 5.992088609 | 6.207502459 | 6.261154673 | 6.250772132 | 6.001126665 | 0.070681112 | -0.881860189 |
| hsa-miR-1225-3p | 9.760553633 | 9.377644358 | 9.58458675 | 9.722807531 | 11.25844826 | 10.53974043 | 11.1909358 | 10.97763769 | 11.22427306 | 10.35469017 | 0.000129132 | 1.312889498 |
| hsa-miR-1225-5p | 8.626439137 | 8.170926525 | 7.73470962 | 9.945004816 | 12.50061784 | 13.18780053 | 13.96936853 | 13.40900634 | 13.74706006 | 11.99848466 | 0.000341189 | 4.516119635 |
| hsa-miR-1226 | 7.898752526 | 6.106432078 | 6.489446599 | 6.078951341 | 6.399171094 | 6.465974465 | 6.930145948 | 6.332707934 | 6.584962501 | 11.36779694 | 0.466550853 | 0.703397511 |
| hsa-miR-1226* | 7.266786541 | 7.23935985 | 6.966937979 | 6.903279342 | 7.641690523 | 7.625708843 | 8.730809755 | 8.763876285 | 7.635173947 | 6.714245518 | 0.063726424 | 0.75782655 |
| hsa-miR-1227 | 7.048214385 | 6.263973355 | 6.0725346 | 6.276124405 | 6.530601415 | 6.316507819 | 6.523561956 | 6.543805176 | 7.117902789 | 6.45779126 | 0.527051085 | 0.166483383 |
| hsa-miR-1228 | 7.113742166 | 6.417852515 | 6.43629512 | 6.676662335 | 6.155830172 | 8.437544026 | 5.988684687 | 6.422905743 | 6.411087227 | 7.006746832 | 0.855745356 | 0.075995081 |
| hsa-miR-1228* | 12.42385126 | 11.32187174 | 12.40167953 | 11.47126876 | 9.62580015 | 9.49645436 | 10.35110487 | 9.276589716 | 9.63499251 | 12.42385126 | 0.014646645 | -1.76986901 |
| hsa-miR-1229 | 8.539158811 | 7.328226119 | 7.246503098 | 7.433794059 | 9.570614722 | 9.226773423 | 9.920427296 | 8.956666555 | 10.21188829 | 7.502235115 | 0.012310956 | 1.594513712 |
| hsa-miR-1231 | 10.80727441 | 8.862017377 | 8.536441505 | 8.821773982 | 10.09882166 | 9.226773423 | 10.04384754 | 11.97799537 | 10.36888837 | 8.405566974 | 0.320386021 | 0.763438737 |
| hsa-miR-1233 | 9.548436625 | 9.541677449 | 9.171927354 | 9.487840034 | 11.32187174 | 10.64925618 | 11.52645033 | 10.72766524 | 11.47722702 | 10.82631014 | 3.33E-05 | 1.650659742 |
| hsa-miR-1234 | 9.034798963 | 10.11517373 | 8.167418146 | 7.159871337 | 8.68580002 | 8.710462074 | 8.889199726 | 8.326204751 | 8.959132577 | 8.961594391 | 0.84380516 | 0.136083378 |
| hsa-miR-1236 | 6.151777655 | 5.894817763 | 5.581953751 | 5.581953751 | 6.155830172 | 6.316507819 | 5.786596362 | 6.332707934 | 6.411087227 | 8.248876343 | 0.096185228 | 0.739308579 |
| hsa-miR-1237 | 6.917670434 | 7.943686952 | 7.155830172 | 6.105384749 | 5.905687849 | 6.092757141 | 6.089582893 | 6.0725346 | 5.9795681 | 6.100136671 | 0.078608871 | -0.990598534 |
| hsa-miR-1238 | 14.45914981 | 12.67185062 | 13.63051741 | 13.80583454 | 14.14972331 | 14.18441048 | 14.45914981 | 14.51607549 | 14.45914981 | 14.14972331 | 0.162773722 | 0.677867271 |
| hsa-miR-124 | 6.151777655 | 5.911691582 | 5.930737338 | 6.053111336 | 6.274261661 | 6.316507819 | 7.599168993 | 6.168922782 | 6.411087227 | 6.194756854 | 0.08484274 | 0.482288078 |
| hsa-miR-124* | 5.944858446 | 5.716990894 | 5.760220946 | 5.842978832 | 11.849366 | 5.911691582 | 5.786596362 | 5.820178962 | 5.9795681 | 6.001126665 | 0.328320222 | 1.075159 |
| hsa-miR-1243 | 7.230741003 | 7.410239331 | 7.815703503 | 7.799605422 | 8.259507895 | 8.950993232 | 8.413204789 | 7.753885283 | 8.453682186 | 8.168922782 | 0.007754815 | 0.769293713 |
| hsa-miR-1244 | 7.336283388 | 8.548436625 | 8.781359714 | 7.199672345 | 6.05202456 | 6.196725061 | 5.988684687 | 6.0725346 | 6.10433666 | 6.329123596 | 0.01931474 | -1.842533157 |
| hsa-miR-1245 | 8.838573653 | 8.692789835 | 8.495455333 | 9.541677449 | 6.155830172 | 8.710462074 | 6.356672044 | 7.1017131 | 6.411087227 | 7.799605422 | 0.005606531 | -1.802895728 |
| hsa-miR-1246 | 13.92203613 | 12.179878 | 13.60579201 | 12.42385126 | 12.40167953 | 12.21577565 | 14.07082534 | 11.6092711 | 5.9795681 | 6.329123596 | 0.126051896 | -2.598515462 |
| hsa-miR-1247 | 9.082149041 | 9.073739915 | 8.812498225 | 9.981995318 | 8.381110294 | 5.992088609 | 6.721782768 | 5.988684687 | 6.10433666 | 8.498849207 | 0.003864323 | -2.289786921 |
| hsa-miR-1248 | 10.30138187 | 11.18090371 | 11.08261478 | 9.096188046 | 8.151523994 | 8.040837649 | 8.289557848 | 8.405566974 | 7.864805194 | 7.006746832 | 0.008787695 | -2.455432353 |
| hsa-miR-1249 | 8.651769271 | 8.498849207 | 7.861707287 | 7.747185967 | 7.075746538 | 8.25502857 | 7.78496195 | 7.515305605 | 7.408117408 | 7.625708843 | 0.082751388 | -0.579066447 |
| hsa-miR-124a:9.1 | 6.001126665 | 6.038918989 | 6.105384749 | 6.224966365 | 11.72144102 | 6.609548139 | 6.930145948 | 10.79075575 | 7.117902789 | 6.857980995 | 0.061086362 | 2.245363248 |
| hsa-miR-1250 | 7.55688919 | 6.815063017 | 7.339850003 | 6.601399391 | 9.14822234 | 8.15810472 | 8.889199726 | 8.15810472 | 8.725877459 | 7.39145842 | 0.004686866 | 1.333527497 |
| hsa-miR-1251 | 6.815063017 | 6.385431037 | 6.559950613 | 6.501439145 | 6.530601415 | 6.465974465 | 6.207502459 | 6.261154673 | 6.250772132 | 7.925109324 | 0.88879129 | 0.041381458 |
| hsa-miR-1252 | 7.056366761 | 6.046578367 | 6.132371199 | 6.078951341 | 7.484218708 | 6.465974465 | 7.599168993 | 7.388878339 | 7.117902789 | 6.714245518 | 0.038403936 | 0.799831218 |
| hsa-miR-1253 | 6.215290306 | 6.224966365 | 6.339850003 | 6.570614722 | 9.248164779 | 8.60936345 | 9.821455119 | 9.852139328 | 9.207990433 | 7.502235115 | 0.00048852 | 2.702544355 |
| hsa-miR-1254 | 13.36342298 | 10.16440416 | 10.53974043 | 11.49355527 | 11.79445648 | 12.42385126 | 13.12847772 | 13.88718253 | 13.68986697 | 12.45213826 | 0.123447844 | 1.505714826 |
| hsa-miR-1255a | 5.930737338 | 6 | 8.084808388 | 8.297374861 | 6.155830172 | 6.092757141 | 6.356672044 | 6.0725346 | 6.411087227 | 6.329123596 | 0.282645095 | -0.841896017 |
| hsa-miR-1255b | 6.762880293 | 7.312882955 | 6.736740155 | 8.656067274 | 9.32395546 | 8.894817763 | 9.223036338 | 8.405566974 | 9.958697703 | 8.862017377 | 0.02141421 | 1.744205933 |
| hsa-miR-1256 | 6.651051691 | 7.394033895 | 7.048214385 | 7.400025518 | 6.862947248 | 7.316960287 | 7.145677455 | 7.1017131 | 7.635173947 | 7.712870868 | 0.466410668 | 0.172559112 |
| hsa-miR-1257 | 9.528063348 | 8.808964175 | 8.735725245 | 9.207990433 | 11.14539087 | 10.44159552 | 11.36380623 | 10.56985561 | 11.30691611 | 11.26982798 | 0.000100382 | 1.946046255 |
| hsa-miR-1258 | 7.384567923 | 6.569855608 | 7.77280941 | 5.744161096 | 6.274261661 | 6.465974465 | 6.089582893 | 6.332707934 | 6.83541884 | 8.498849207 | 0.844366744 | -0.118382676 |
| hsa-miR-1259 | 6.069315495 | 6.422905743 | 6.89056829 | 6.995484519 | 7.982993575 | 6.755555262 | 7.400025518 | 6.668175869 | 7.635173947 | 6.857980995 | 0.07909206 | 0.622082349 |
| hsa-miR-125a-3p | 9.342296946 | 8.653919873 | 9.377644358 | 9.435878577 | 9.792139552 | 8.950993232 | 9.650154214 | 10.1321141 | 10.8591465 | 9.920427296 | 0.061990777 | 0.681727543 |
| hsa-miR-125a-5p | 12.82348666 | 12.58282411 | 13.19263957 | 13.68986697 | 6.862947248 | 7.625708843 | 7.145677455 | 6.790511412 | 7.408117408 | 7.006746832 | 5.61E-06 | -5.932252797 |
| hsa-miR-125b | 12.50061784 | 13.5187515 | 13.5347649 | 13.85368967 | 6.155830172 | 6.755555262 | 6.356672044 | 6.168922782 | 6.250772132 | 6.857980995 | 2.17747E-05 | -6.92766708 |
| hsa-miR-125b-1* | 6.927185358 | 7.829722735 | 7.027905997 | 7.244125943 | 7.799605422 | 8.521207816 | 8.002252452 | 7.753885283 | 7.408117408 | 9.655262373 | 0.043355637 | 0.932820117 |
| hsa-miR-125b-2* | 8.117902789 | 10.53974043 | 10.027906 | 10.42311591 | 7.982993575 | 7.625708843 | 8.413204789 | 7.927777962 | 8.597307691 | 6.714245518 | 0.033897755 | -1.900293217 |
| hsa-miR-126 | 11.29582632 | 11.33770552 | 11.52645033 | 10.97763769 | 11.80779765 | 11.25844826 | 12.04668067 | 11.37444181 | 12.03675744 | 11.3810562 | 0.082425322 | 0.366458706 |
| hsa-miR-126* | 13.12028648 | 13.61622719 | 13.50039377 | 13.27035373 | 8.259507895 | 13.96936853 | 7.78496195 | 12.96155822 | 7.635173947 | 13.85368967 | 0.096007941 | -2.632771924 |
| hsa-miR-1260 | 13.7905725 | 12.14063808 | 13.10212335 | 11.55588324 | 7.075746538 | 13.10212335 | 7.400025518 | 6.953032377 | 7.408117408 | 6.569855608 | 0.004847149 | -4.562487493 |
| hsa-miR-1261 | 6.661778098 | 7.123604124 | 6.983563697 | 7.257859235 | 6.155830172 | 6.465974465 | 8.730809755 | 6.332707934 | 6.10433666 | 6.714245518 | 0.569860489 | -0.256050538 |
| hsa-miR-1262 | 8.053654418 | 8.003939486 | 7.505414609 | 8.486231678 | 10.65391987 | 12.01300855 | 10.65391987 | 9.760553633 | 10.79903872 | 9.779883475 | 0.000211966 | 2.597743974 |
| hsa-miR-1263 | 7.628627802 | 6.756222713 | 6.66106548 | 7.415319248 | 7.641690523 | 8.344073938 | 9.958697703 | 10.14095755 | 9.574309691 | 7.502235115 | 0.013979347 | 1.745018609 |
| hsa-miR-1264 | 6.195741293 | 6.287250643 | 6.387155718 | 6.559950613 | 8.151523994 | 7.921245889 | 6.356672044 | 6.168922782 | 6.250772132 | 7.799605422 | 0.108795026 | 0.750599144 |
| hsa-miR-1265 | 5.9795681 | 6.154818109 | 6.0725346 | 6.224966365 | 6.862947248 | 6.092757141 | 7.145677455 | 6.953032377 | 6.83541884 | 6.45779126 | 0.009384236 | 0.61663226 |
| hsa-miR-1266 | 5.790511412 | 5.815063017 | 6.601399391 | 6.029011087 | 5.905687849 | 5.856736756 | 5.786596362 | 5.861707287 | 6.250772132 | 6.329123596 | 0.786535815 | -0.060558896 |
| hsa-miR-1267 | 9.207990433 | 9.014299495 | 8.830990481 | 9.32395546 | 10.50476934 | 10.12805878 | 10.87198222 | 10.11517373 | 10.5844928 | 10.37585398 | 3.87E-05 | 1.335746175 |
| hsa-miR-1268 | 9.741635516 | 8.293471649 | 9.063395081 | 8.68580002 | 6.862947248 | 6.951867504 | 7.145677455 | 6.790511412 | 6.83541884 | 6.857980995 | 0.006153196 | -2.038674991 |
| hsa-miR-1269 | 6.510961919 | 6.8899602 | 6.726558779 | 6.715618859 | 6.671010241 | 8.521207816 | 6.930145948 | 6.953032377 | 7.635173947 | 8.862017377 | 0.063797345 | 0.884656345 |
| hsa-miR-1270 | 6.716990894 | 6.872213144 | 6.77478706 | 8.388447876 | 7.799605422 | 6.951867504 | 7.78496195 | 7.596935142 | 8.11997861 | 7.270996061 | 0.410507826 | 0.399281038 |
| hsa-miR-1271 | 10.09354961 | 10.35469017 | 10.31955923 | 9.924812504 | 9.14822234 | 8.521207816 | 8.986979709 | 8.203837384 | 8.959132577 | 7.799605422 | 0.000319816 | -1.569988671 |
| hsa-miR-1272 | 8.713386515 | 9.192539408 | 9.014299495 | 9.366977836 | 10.09882166 | 9.226773423 | 8.730809755 | 9.492153626 | 8.597307691 | 8.063395081 | 0.913054294 | -0.03692394 |
| hsa-miR-1273 | 6.667466405 | 9.66035251 | 8.692789835 | 9.650154214 | 6.530601415 | 6.465974465 | 6.523561956 | 6.543805176 | 6.83541884 | 6.857980995 | 0.061814566 | -2.041466933 |
| hsa-miR-127-3p | 9.57799522 | 10.98463207 | 10.57601187 | 10.46546437 | 11.27653156 | 10.36062765 | 11.59577031 | 10.50809187 | 11.41494944 | 9.929554316 | 0.304463197 | 0.446561639 |
| hsa-miR-1274a | 11.36779694 | 9.456765088 | 10.89231512 | 8.741635516 | 8.381110294 | 9.024308597 | 8.730809755 | 8.405566974 | 7.635173947 | 9.846430632 | 0.09435997 | -1.444061468 |
| hsa-miR-1274b | 13.98751273 | 12.90809234 | 13.47772033 | 13.28896698 | 5.905687849 | 12.97835296 | 5.62935662 | 5.675251386 | 5.804776378 | 5.842978832 | 0.002682845 | -6.442839091 |
| hsa-miR-1275 | 10.50143915 | 9.49645436 | 10.34751064 | 10.72766524 | 7.075746538 | 7.316960287 | 7.145677455 | 6.261154673 | 6.83541884 | 6.569855608 | 9.69E-05 | -3.400798446 |
| hsa-miR-127-5p | 6.008988783 | 6.82527683 | 7.588714636 | 6.004501392 | 7.484218708 | 6.092757141 | 5.988684687 | 5.988684687 | 6.411087227 | 6.100136671 | 0.58148579 | -0.26260889 |
| hsa-miR-1276 | 6.452858965 | 6.663202279 | 6.667466405 | 6.99095486 | 8.968378801 | 7.921245889 | 8.889199726 | 8.003096215 | 9.098821663 | 9.082149041 | 9.54E-05 | 1.966861262 |
| hsa-miR-1277 | 5.892391026 | 5.927185358 | 5.930737338 | 6.171927354 | 6.274261661 | 6.465974465 | 6.930145948 | 6.168922782 | 6.411087227 | 6.001126665 | 0.029872361 | 0.394692856 |
| hsa-miR-1278 | 6.091699834 | 7.049848549 | 5.815063017 | 5.716990894 | 6.274261661 | 6.196725061 | 6.207502459 | 5.902073579 | 12.62956153 | 8.063395081 | 0.261837643 | 1.377185989 |
| hsa-miR-1279 | 6.74281467 | 6.263973355 | 6.339850003 | 6.54225805 | 8.381110294 | 7.625708843 | 8.289557848 | 8.897542973 | 7.864805194 | 7.625708843 | 0.000165832 | 1.641848313 |
| hsa-miR-128 | 12.06062839 | 10.40290547 | 10.99957727 | 9.029563314 | 8.968378801 | 11.51491126 | 11.00562455 | 10.68088692 | 10.21188829 | 7.502235115 | 0.486777282 | -0.642514454 |
| hsa-miR-1280 | 12.179878 | 12.19614103 | 12.03675744 | 12.06062839 | 11.75768169 | 11.93475974 | 11.97799537 | 10.94005661 | 11.99848466 | 13.68986697 | 0.85964085 | -0.06854371 |
| hsa-miR-1281 | 7.829722735 | 10.14095755 | 6.6395216 | 8.283319907 | 8.381110294 | 7.75087444 | 8.413204789 | 7.857047916 | 7.117902789 | 7.502235115 | 0.639805708 | -0.386317891 |
| hsa-miR-1282 | 11.3810562 | 7.161887682 | 7.519243094 | 7.829722735 | 12.03675744 | 11.21249639 | 12.75713995 | 13.67325386 | 13.47772033 | 11.849366 | 0.018892499 | 4.0281449 |
| hsa-miR-1283 | 6.069315495 | 6.350497247 | 6.224966365 | 6.601399391 | 7.484218708 | 7.316960287 | 7.145677455 | 7.661778098 | 8.597307691 | 7.925109324 | 0.000635083 | 1.37696397 |
| hsa-miR-1284 | 9.685449638 | 8.982708429 | 8.775116404 | 8.986979709 | 11.18592847 | 10.58922979 | 11.62858224 | 11.12023788 | 11.5740258 | 10.60385812 | 0.000130024 | 2.009413505 |
| hsa-miR-1285 | 10.15000109 | 10.00450139 | 9.809125002 | 10.09354961 | 6.671010241 | 12.53225851 | 6.721782768 | 7.23935985 | 6.584962501 | 6.45779126 | 0.063213575 | -2.313100087 |
| hsa-miR-1286 | 8.58458675 | 8.423746229 | 8.342963574 | 8.982708429 | 10.30628959 | 9.852139328 | 10.4943056 | 9.595443985 | 10.1365426 | 9.920427296 | 0.0001135 | 1.46735682 |
| hsa-miR-1287 | 8.713386515 | 7.764871591 | 7.943686952 | 6.696272084 | 6.274261661 | 6.465974465 | 6.930145948 | 6.543805176 | 6.250772132 | 8.324180547 | 0.108570172 | -0.981364297 |
| hsa-miR-1288 | 7.502235115 | 7.158862106 | 6.60659028 | 6.896029603 | 8.518849829 | 7.117383367 | 8.289557848 | 8.670656249 | 8.11997861 | 7.39145842 | 0.015572466 | 0.977051444 |
| hsa-miR-1289 | 10.2701785 | 7.413204789 | 7.658211483 | 8.423746229 | 10.61966977 | 10.86712427 | 12.01300855 | 12.26617162 | 12.16091137 | 10.17816685 | 0.011507143 | 2.909506822 |
| hsa-miR-128a:9.1 | 8.097505456 | 6.106432078 | 6.851749041 | 6.136478518 | 7.799605422 | 11.26982798 | 8.289557848 | 6.790511412 | 7.635173947 | 7.006746832 | 0.139693329 | 1.333862634 |
| hsa-miR-128b:9.1 | 8.003939486 | 6.43629512 | 7.103287808 | 6.794415866 | 8.259507895 | 8.040837649 | 8.167418146 | 7.927777962 | 8.11997861 | 10.24257869 | 0.024111138 | 1.375198588 |
| hsa-miR-129* | 6.844234988 | 7.038918989 | 8.520029304 | 6.512542955 | 7.799605422 | 7.501040996 | 8.002252452 | 8.326204751 | 7.635173947 | 7.141596278 | 0.348688725 | 0.505380749 |
| hsa-miR-1290 | 10.62561753 | 11.99848466 | 11.54099979 | 9.631540867 | 11.93475974 | 11.1909358 | 12.21577565 | 13.03414554 | 12.1027959 | 10.8622499 | 0.182817492 | 0.940949712 |
| hsa-miR-1291 | 6.965784285 | 7.019034669 | 6.475733431 | 6.601399391 | 6.399171094 | 6.465974465 | 6.721782768 | 6.790511412 | 6.83541884 | 6.329123596 | 0.320858086 | -0.175157581 |
| hsa-miR-1292 | 6.301953395 | 5.944858446 | 6.05202456 | 5.970393538 | 5.905687849 | 5.856736756 | 5.87282876 | 6.0725346 | 6.10433666 | 6.001126665 | 0.337537984 | -0.09843227 |
| hsa-miR-1293 | 9.620036498 | 7.432959407 | 6.949534933 | 7.05202456 | 8.259507895 | 7.625708843 | 7.78496195 | 7.753885283 | 6.83541884 | 8.498849207 | 0.967166611 | 0.029416487 |
| hsa-miR-129-3p | 5.933100475 | 7.958262697 | 7.722466024 | 6.078951341 | 6.05202456 | 5.992088609 | 6.207502459 | 6.168922782 | 6.10433666 | 6.329123596 | 0.238923106 | -0.780862023 |
| hsa-miR-1294 | 8.700786478 | 8.664269492 | 8.302867361 | 7.913487841 | 8.151523994 | 8.344073938 | 8.413204789 | 8.486231678 | 8.597307691 | 9.57799522 | 0.49048621 | 0.199703425 |
| hsa-miR-1295 | 9.377644358 | 9.034798963 | 9.101450482 | 10.01066796 | 9.792139552 | 9.101450482 | 9.77016832 | 8.522581531 | 9.783898589 | 8.405566974 | 0.67271095 | -0.151839533 |
| hsa-miR-129-5p | 13.12847772 | 11.1028287 | 11.16804527 | 11.52645033 | 12.21577565 | 11.15785217 | 12.33776126 | 13.80583454 | 12.25017997 | 11.21249639 | 0.50765276 | 0.431866159 |
| hsa-miR-1296 | 9.36128586 | 8.781359714 | 9.207990433 | 8.170926525 | 6.155830172 | 6.465974465 | 6.523561956 | 6.332707934 | 6.250772132 | 6.329123596 | 0.001848371 | -2.537395591 |
| hsa-miR-1297 | 7.365884968 | 11.25844826 | 9.421644094 | 8.155324229 | 9.14822234 | 8.344073938 | 8.607700188 | 7.927777962 | 8.11997861 | 8.168922782 | 0.495503817 | -0.664212751 |
| hsa-miR-1298 | 6.383704292 | 6.529820947 | 6.653203362 | 7.010108453 | 6.862947248 | 7.501040996 | 7.145677455 | 6.543805176 | 6.83541884 | 7.006746832 | 0.112496942 | 0.338396828 |
| hsa-miR-1299 | 7.339850003 | 7.424586226 | 7.649615459 | 8.741635516 | 7.799605422 | 9.024308597 | 13.61622719 | 7.388878339 | 7.864805194 | 8.986979709 | 0.230685112 | 1.324545607 |
| hsa-miR-1300 | 8.959132577 | 9.252665432 | 9.589463893 | 10.56367296 | 8.518849829 | 11.55588324 | 13.15864124 | 7.661778098 | 8.300123725 | 8.498849207 | 0.980567927 | 0.024453842 |
| hsa-miR-1301 | 12.26617162 | 9.570614722 | 9.28748153 | 9.893756568 | 10.8622499 | 12.06062839 | 12.53225851 | 13.65089467 | 13.15864124 | 12.33776126 | 0.039473064 | 2.179232884 |
| hsa-miR-1302 | 6.238404739 | 6.170926525 | 6.199672345 | 6.431288654 | 7.273795599 | 6.951867504 | 7.400025518 | 6.953032377 | 8.300123725 | 6.857980995 | 0.004541448 | 1.029397887 |
| hsa-miR-1303 | 9.517472554 | 8.173926932 | 6.569855608 | 6.311975314 | 13.18780053 | 6.465974465 | 6.356672044 | 10.05521464 | 6.250772132 | 7.006746832 | 0.686028757 | 0.577222504 |
| hsa-miR-1304 | 8.405566974 | 7.170926525 | 6.944858446 | 9.685449638 | 7.799605422 | 6.755555262 | 6.930145948 | 13.15864124 | 6.83541884 | 6.857980995 | 0.99711395 | 0.004524223 |
| hsa-miR-1305 | 8.599540966 | 8.773468928 | 9.068240861 | 8.75788999 | 10.5844928 | 10.30138187 | 10.78598367 | 9.91064273 | 10.76088624 | 10.027906 | 2.65E-05 | 1.595430366 |
| hsa-miR-1307 | 11.2444233 | 9.601399391 | 10.36654079 | 9.252665432 | 13.15864124 | 7.316960287 | 7.400025518 | 12.1027959 | 7.408117408 | 7.006746832 | 0.420323838 | -1.050709364 |
| hsa-miR-1308 | 14.07082534 | 12.40167953 | 13.90966811 | 13.18780053 | 6.274261661 | 11.80779765 | 6.089582893 | 6.953032377 | 6.250772132 | 6.45779126 | 0.000580981 | -6.086953716 |
| hsa-miR-130a | 10.99957727 | 12.53225851 | 12.25017997 | 12.83547806 | 10.5844928 | 11.2444233 | 9.650154214 | 11.62858224 | 12.45213826 | 11.51491126 | 0.124436066 | -0.975256438 |
| hsa-miR-130a* | 11.39247843 | 7.628627802 | 9.548436625 | 10.06123617 | 11.47126876 | 8.819061399 | 12.94553528 | 13.10212335 | 12.67185062 | 10.34751064 | 0.111589757 | 1.901863588 |
| hsa-miR-130b | 7.421223299 | 9.741635516 | 7.712870868 | 9.347953493 | 11.72970571 | 8.437544026 | 8.986979709 | 8.106955457 | 8.597307691 | 8.063395081 | 0.610187935 | 0.431060484 |
| hsa-miR-130b* | 7.609548139 | 7.861707287 | 7.712870868 | 8.090906345 | 6.671010241 | 8.521207816 | 6.207502459 | 6.790511412 | 5.9795681 | 12.90809234 | 0.980634717 | 0.027557235 |
| hsa-miR-132 | 11.59577031 | 13.55601501 | 13.57510193 | 12.90809234 | 6.530601415 | 6.951867504 | 6.523561956 | 12.83547806 | 6.411087227 | 12.16091137 | 0.016173406 | -4.339826977 |
| hsa-miR-132* | 7.6727789 | 9.044667331 | 7.921245889 | 7.614709844 | 7.273795599 | 7.501040996 | 6.721782768 | 8.405566974 | 7.635173947 | 9.57799522 | 0.700974421 | -0.21079124 |
| hsa-miR-1321 | 6.465974465 | 6.404290064 | 6.45779126 | 6.512542955 | 6.671010241 | 6.465974465 | 6.356672044 | 6.168922782 | 6.10433666 | 6.714245518 | 0.675067597 | -0.046622735 |
| hsa-miR-1322 | 6.746178381 | 6.99095486 | 6.951867504 | 8.170926525 | 8.151523994 | 8.710462074 | 7.78496195 | 8.326204751 | 7.408117408 | 8.498849207 | 0.055042027 | 0.931704746 |
| hsa-miR-1323 | 9.101450482 | 9.271929844 | 8.919459269 | 9.029563314 | 10.1365426 | 9.644306962 | 10.35110487 | 9.929554316 | 10.1365426 | 9.202491187 | 0.003470976 | 0.819489696 |
| hsa-miR-1324 | 6.085339669 | 6.106432078 | 6.145677455 | 6.311975314 | 7.273795599 | 6.316507819 | 9.77016832 | 7.23935985 | 6.83541884 | 7.006746832 | 0.052910401 | 1.244643414 |
| hsa-miR-133a | 8.692789835 | 9.129283017 | 8.599540966 | 9.069718276 | 11.50858711 | 10.77107674 | 11.77020962 | 10.11517373 | 11.72144102 | 10.65391987 | 0.000174401 | 2.217234994 |
| hsa-miR-133b | 7.906289347 | 6.318316841 | 6.43629512 | 6.819540461 | 7.641690523 | 6.755555262 | 7.145677455 | 10.96079838 | 6.83541884 | 6.45779126 | 0.356832796 | 0.762711511 |
| hsa-miR-134 | 10.24257869 | 11.89958351 | 11.39247843 | 11.93475974 | 12.78426676 | 11.86546264 | 7.400025518 | 9.66035251 | 7.117902789 | 6.45779126 | 0.109454138 | -2.153049844 |
| hsa-miR-135a | 7.056366761 | 8.167418146 | 7.394033895 | 8.141596278 | 10.38046107 | 9.315715658 | 10.2564446 | 9.082149041 | 10.36888837 | 9.403012024 | 0.000739418 | 2.111258021 |
| hsa-miR-135a* | 8.208478242 | 7.413204789 | 7.266786541 | 7.694183743 | 10.03589879 | 9.768101599 | 11.00562455 | 10.20371505 | 9.852139328 | 8.063395081 | 0.00165513 | 2.175815738 |
| hsa-miR-135b | 7.458201525 | 7.783980414 | 7.225930411 | 7.721782768 | 8.381110294 | 8.25502857 | 9.160249616 | 8.670656249 | 8.725877459 | 7.270996061 | 0.020965715 | 0.863179262 |
| hsa-miR-135b* | 7.048214385 | 7.006746832 | 7.121015401 | 7.24697806 | 6.862947248 | 11.82137539 | 7.599168993 | 6.790511412 | 7.117902789 | 6.569855608 | 0.439468975 | 0.687888238 |
| hsa-miR-136 | 6.422905743 | 8.316507819 | 6.510961919 | 6.804776378 | 7.075746538 | 6.755555262 | 8.167418146 | 7.661778098 | 7.408117408 | 7.141596278 | 0.503781154 | 0.354580657 |
| hsa-miR-136* | 6.019034669 | 7.522385366 | 7.697315122 | 6.857980995 | 5.980710829 | 6.316507819 | 6.089582893 | 5.861707287 | 6.10433666 | 6.329123596 | 0.094288706 | -0.910517524 |
| hsa-miR-137 | 7.837312556 | 7.270996061 | 7.111657346 | 7.477353527 | 8.968378801 | 9.768101599 | 9.958697703 | 10.11517373 | 9.783898589 | 9.57799522 | 1.14E-05 | 2.271044402 |
| hsa-miR-138 | 7.225930411 | 7.675957033 | 7.469234794 | 8.633721813 | 9.14822234 | 8.819061399 | 9.082149041 | 8.522581531 | 9.574309691 | 8.405566974 | 0.021523359 | 1.17410415 |
| hsa-miR-138-1* | 6.539158811 | 7.230741003 | 6.569855608 | 6.876516947 | 9.62580015 | 9.101450482 | 9.726899318 | 8.522581531 | 9.852139328 | 8.910492832 | 1.53E-05 | 2.485825848 |
| hsa-miR-138-2* | 7.064473329 | 7.357552005 | 7.093813673 | 7.449561375 | 8.68580002 | 7.75087444 | 8.889199726 | 8.522581531 | 9.098821663 | 8.248876343 | 0.000602945 | 1.291342192 |
| hsa-miR-139-3p | 10.72766524 | 9.56414949 | 10.10407452 | 9.57799522 | 8.151523994 | 7.501040996 | 8.607700188 | 8.106955457 | 8.11997861 | 9.044667331 | 0.002124525 | -1.738160021 |
| hsa-miR-139-5p | 11.82137539 | 12.21577565 | 12.94553528 | 11.62858224 | 8.68580002 | 8.60936345 | 8.730809755 | 9.530893981 | 8.725877459 | 8.405566974 | 0.000203609 | -3.371431868 |
| hsa-miR-140-3p | 12.21577565 | 12.3835693 | 11.89958351 | 12.25017997 | 12.46867495 | 13.47772033 | 12.67185062 | 13.59243368 | 13.41629614 | 13.18780053 | 0.002763793 | 0.948518933 |
| hsa-miR-140-5p | 7.588714636 | 7.452035274 | 7.296457407 | 7.761551232 | 7.982993575 | 8.25502857 | 8.413204789 | 8.003096215 | 8.959132577 | 8.589838376 | 0.001780351 | 0.842526046 |
| hsa-miR-141 | 7.266786541 | 11.35980446 | 10.76088624 | 11.35980446 | 7.273795599 | 6.755555262 | 7.400025518 | 7.596935142 | 12.3835693 | 7.502235115 | 0.163077351 | -2.034801101 |
| hsa-miR-141* | 6.529820947 | 6.112700133 | 6.329123596 | 6.653203362 | 6.05202456 | 6.092757141 | 6.356672044 | 6.543805176 | 6.10433666 | 7.270996061 | 0.990412129 | -0.002780069 |
| hsa-miR-142-3p | 6.716990894 | 8.3226042 | 7.411087227 | 9.517472554 | 6.862947248 | 6.951867504 | 7.145677455 | 6.668175869 | 6.83541884 | 7.006746832 | 0.172099256 | -1.080233094 |
| hsa-miR-142-5p | 6.334496768 | 6.99095486 | 6.175923742 | 6.207502459 | 7.799605422 | 11.1508262 | 6.089582893 | 6.422905743 | 6.411087227 | 6.329123596 | 0.29794765 | 0.939969056 |
| hsa-miR-143 | 13.28896698 | 14.41700199 | 14.23044081 | 14.18441048 | 8.893756568 | 8.710462074 | 8.514911265 | 7.753885283 | 9.207990433 | 9.52277767 | 8.29E-07 | -5.26290785 |
| hsa-miR-143* | 10.92577695 | 10.76088624 | 10.20371505 | 10.2701785 | 11.61895898 | 10.98463207 | 11.79445648 | 12.179878 | 11.83543858 | 11.1028287 | 0.004097899 | 1.045892947 |
| hsa-miR-144 | 6.334496768 | 6.199672345 | 6.404290064 | 6.338959175 | 10.35110487 | 9.939285356 | 13.5347649 | 9.192539408 | 10.48290877 | 11.97799537 | 0.000814494 | 4.593745191 |
| hsa-miR-144* | 7.584210901 | 8.498849207 | 9.164152708 | 7.336283388 | 5.752213368 | 5.911691582 | 5.87282876 | 5.752213368 | 5.804776378 | 6.001126665 | 0.011734723 | -2.296732364 |
| hsa-miR-144:9.1 | 7.796364138 | 7.071462363 | 6.83541884 | 7.083213368 | 11.77020962 | 11.02517418 | 12.14063808 | 9.318768743 | 11.97799537 | 10.94807516 | 6.47E-05 | 4.000195514 |
| hsa-miR-145 | 13.80583454 | 13.80583454 | 13.84325371 | 13.84325371 | 11.2062818 | 11.65745244 | 11.43514934 | 10.61369739 | 11.25844826 | 11.1508262 | 8.39E-06 | -2.604234887 |
| hsa-miR-145* | 10.84172158 | 11.48316077 | 11.5740258 | 11.54099979 | 10.8622499 | 9.56414949 | 11.05372229 | 9.83019827 | 11.14539087 | 10.12399203 | 0.022299452 | -0.930026509 |
| hsa-miR-1468 | 12.58282411 | 8.450386479 | 8.208478242 | 7.936637939 | 12.53225851 | 11.68877483 | 13.57510193 | 13.85368967 | 13.30457949 | 11.42621233 | 0.045128869 | 3.4355211 |
| hsa-miR-146a | 12.03675744 | 13.12028648 | 12.32673844 | 12.96155822 | 6.862947248 | 13.59243368 | 6.721782768 | 6.422905743 | 6.584962501 | 13.76677734 | 0.058536785 | -3.619366934 |
| hsa-miR-146a* | 6.224001674 | 6.558420713 | 6.523561956 | 6.759555343 | 7.484218708 | 6.951867504 | 6.356672044 | 6.668175869 | 6.411087227 | 7.270996061 | 0.158727572 | 0.340784647 |
| hsa-miR-146b-3p | 6.697662633 | 7.398316163 | 7.093813673 | 7.168922782 | 8.381110294 | 8.344073938 | 8.889199726 | 7.753885283 | 8.453682186 | 8.961594391 | 0.000339853 | 1.37424549 |
| hsa-miR-146b-5p | 10.4871366 | 12.75713995 | 11.68877483 | 12.1027959 | 7.641690523 | 12.93049711 | 7.78496195 | 8.15810472 | 8.11997861 | 12.69522829 | 0.095130434 | -2.203884952 |
| hsa-miR-147 | 7.469234794 | 6.176921112 | 5.988684687 | 6.053111336 | 10.38046107 | 9.963257357 | 10.5844928 | 10.31537603 | 13.18780053 | 7.270996061 | 0.002757456 | 3.861742657 |
| hsa-miR-147b | 9.67047922 | 9.730979533 | 9.242578689 | 9.730979533 | 10.50476934 | 9.981995318 | 11.16804527 | 10.84172158 | 11.33220442 | 9.996897126 | 0.005312543 | 1.043851265 |
| hsa-miR-148a | 11.50858711 | 13.63051741 | 13.39700607 | 13.65089467 | 8.151523994 | 8.344073938 | 8.730809755 | 8.486231678 | 8.453682186 | 9.082149041 | 0.002121328 | -4.505339551 |
| hsa-miR-148a* | 6.155830172 | 7.85237345 | 6.292781749 | 6.45779126 | 6.274261661 | 7.117383367 | 6.356672044 | 6.168922782 | 6.83541884 | 7.141596278 | 0.92913452 | -0.040651662 |
| hsa-miR-148b | 10.64925618 | 12.55837288 | 12.46867495 | 12.65244168 | 7.273795599 | 11.35980446 | 7.599168993 | 7.753885283 | 7.117902789 | 11.02873489 | 0.007154547 | -3.393304418 |
| hsa-miR-148b* | 7.111657346 | 8.128252152 | 7.275193334 | 7.588714636 | 7.982993575 | 7.625708843 | 7.599168993 | 6.953032377 | 7.408117408 | 7.799605422 | 0.898974522 | 0.035483403 |
| hsa-miR-149 | 8.23457796 | 10.17816685 | 9.779883475 | 10.28505538 | 6.155830172 | 6.196725061 | 6.356672044 | 11.16804527 | 6.411087227 | 6.194756854 | 0.029392685 | -2.538901479 |
| hsa-miR-149* | 7.682292371 | 6.912290583 | 8.168922782 | 7.062855655 | 8.259507895 | 7.75087444 | 7.599168993 | 7.596935142 | 7.635173947 | 8.405566974 | 0.260384481 | 0.41794755 |
| hsa-miR-150 | 13.85368967 | 14.14972331 | 14.07082534 | 14.11566957 | 13.26034622 | 14.11566957 | 7.400025518 | 7.753885283 | 7.408117408 | 14.09416843 | 0.062838947 | -3.375441569 |
| hsa-miR-150* | 11.02160465 | 9.97053734 | 10.94807516 | 11.139935 | 10.73538678 | 10.02236781 | 10.81382127 | 11.35980446 | 12.42385126 | 10.07961811 | 0.77262969 | 0.135770245 |
| hsa-miR-151:9.1 | 10.54515755 | 11.28320413 | 11.55588324 | 11.42621233 | 7.799605422 | 8.437544026 | 8.413204789 | 8.897542973 | 8.300123725 | 13.5347649 | 0.074085743 | -1.972150009 |
| hsa-miR-151-3p | 11.06709819 | 12.93049711 | 12.83547806 | 12.3835693 | 8.800252806 | 13.76677734 | 8.730809755 | 7.596935142 | 8.838573653 | 14.01553701 | 0.151140294 | -2.012679713 |
| hsa-miR-151-5p | 12.97835296 | 13.83329532 | 13.68986697 | 13.92203613 | 6.530601415 | 13.85368967 | 6.721782768 | 6.668175869 | 6.584962501 | 13.96936853 | 0.031023194 | -4.551124385 |
| hsa-miR-152 | 12.65244168 | 13.67325386 | 13.76677734 | 13.76677734 | 6.399171094 | 7.316960287 | 6.089582893 | 6.0725346 | 6.250772132 | 6.329123596 | 1.00358E-06 | -7.055121788 |
| hsa-miR-153 | 6.263034406 | 7.081083929 | 7.6727789 | 6.663913842 | 7.075746538 | 7.316960287 | 7.400025518 | 10.62561753 | 6.83541884 | 7.502235115 | 0.219718796 | 0.872464536 |
| hsa-miR-1537 | 7.43629512 | 6.836050355 | 6.74281467 | 7.272396509 | 9.62580015 | 8.344073938 | 9.507001733 | 9.945004816 | 9.456765088 | 8.589838376 | 0.000118548 | 2.172858187 |
| hsa-miR-154 | 9.809125002 | 12.01300855 | 11.25844826 | 11.3810562 | 6.862947248 | 9.443565213 | 6.930145948 | 11.47126876 | 13.43564422 | 6.329123596 | 0.156886452 | -2.036627006 |
| hsa-miR-154* | 6.215290306 | 7.23935985 | 6.489446599 | 7.649615459 | 6.530601415 | 7.316960287 | 7.145677455 | 6.543805176 | 7.117902789 | 6.714245518 | 0.992548151 | -0.003562613 |
| hsa-miR-155 | 10.17816685 | 11.65745244 | 11.65745244 | 11.88542947 | 10.35110487 | 10.20371505 | 10.39687232 | 9.318768743 | 10.50143915 | 9.52277767 | 0.035797042 | -1.295512333 |
| hsa-miR-155* | 5.953032377 | 6.176921112 | 6.292781749 | 6.501439145 | 6.530601415 | 7.117383367 | 6.930145948 | 6.668175869 | 6.411087227 | 6.714245518 | 0.014721691 | 0.497562961 |
| hsa-miR-15a | 11.89958351 | 12.25017997 | 12.04668067 | 11.44403133 | 7.799605422 | 11.79445648 | 7.599168993 | 7.596935142 | 8.453682186 | 13.5187515 | 0.065594402 | -2.44968558 |
| hsa-miR-15a* | 12.69522829 | 8.033973543 | 8.713386515 | 10.69156906 | 11.51491126 | 12.94553528 | 13.30457949 | 13.75690028 | 13.00499288 | 6.001126665 | 0.310673483 | 1.721134957 |
| hsa-miR-15b | 13.63051741 | 13.19746245 | 13.28896698 | 13.12028648 | 12.33776126 | 13.12028648 | 6.523561956 | 11.03981011 | 6.83541884 | 13.92203613 | 0.096067536 | -2.679495866 |
| hsa-miR-15b* | 9.967802645 | 10.74306722 | 10.44159552 | 10.07614753 | 8.968378801 | 12.179878 | 8.607700188 | 8.003096215 | 8.959132577 | 12.58282411 | 0.627128245 | -0.42365158 |
| hsa-miR-16 | 13.45752196 | 13.47772033 | 13.36342298 | 13.30457949 | 13.12028648 | 13.84325371 | 6.356672044 | 6.0725346 | 6.10433666 | 13.83329532 | 0.088439923 | -3.51241472 |
| hsa-miR-16-1* | 7.616181231 | 8.675957033 | 6.938991439 | 6.447909749 | 6.155830172 | 7.75087444 | 5.87282876 | 6.0725346 | 5.9795681 | 6.001126665 | 0.103029378 | -1.114299407 |
| hsa-miR-16-2* | 10.37123213 | 10.30138187 | 10.31118066 | 9.916028718 | 6.05202456 | 7.117383367 | 6.356672044 | 6.668175869 | 6.250772132 | 6.569855608 | 4.53E-08 | -3.722475248 |
| hsa-miR-17 | 11.41494944 | 12.78426676 | 11.93475974 | 12.55837288 | 5.905687849 | 12.32673844 | 6.089582893 | 5.988684687 | 6.10433666 | 12.53225851 | 0.030278098 | -4.0152057 |
| hsa-miR-17* | 7.236014192 | 10.48290877 | 9.958697703 | 10.40290547 | 5.980710829 | 6.092757141 | 6.207502459 | 6.0725346 | 6.10433666 | 6.100136671 | 0.021018463 | -3.42713514 |
| hsa-miR-17-5p:9.1 | 6.93191939 | 6.5360529 | 5.911691582 | 6.136478518 | 6.155830172 | 6.316507819 | 9.40779885 | 11.01164658 | 6.10433666 | 6.100136671 | 0.258806504 | 1.137007195 |
| hsa-miR-181a | 12.96155822 | 12.82348666 | 12.69522829 | 12.26617162 | 10.03589879 | 9.393819448 | 10.17816685 | 9.688862232 | 9.783898589 | 11.25844826 | 3.88E-05 | -2.630095505 |
| hsa-miR-181a* | 8.896181011 | 9.236970886 | 8.476138625 | 8.700786478 | 9.792139552 | 9.226773423 | 9.650154214 | 8.977852308 | 8.959132577 | 9.353587949 | 0.052687749 | 0.49908742 |
| hsa-miR-181a-2* | 9.393819448 | 10.4268937 | 10.64925618 | 10.59395128 | 9.570614722 | 8.60936345 | 9.87175126 | 9.05853297 | 9.852139328 | 11.1909358 | 0.252649296 | -0.573757231 |
| hsa-miR-181b | 12.3835693 | 11.849366 | 11.6092711 | 11.26982798 | 8.68580002 | 11.41494944 | 8.730809755 | 10.22363975 | 8.959132577 | 12.14063808 | 0.033884933 | -1.752180326 |
| hsa-miR-181c | 10.09882166 | 8.775116404 | 6.928370323 | 9.66035251 | 7.641690523 | 8.344073938 | 8.002252452 | 7.753885283 | 7.117902789 | 8.75788999 | 0.282978341 | -0.929382729 |
| hsa-miR-181c* | 7.92243574 | 8.568716189 | 9.429825157 | 8.806066226 | 7.641690523 | 8.040837649 | 7.599168993 | 7.23935985 | 7.864805194 | 9.90523656 | 0.238545936 | -0.633244366 |
| hsa-miR-181d | 6.452858965 | 6.45779126 | 6.510961919 | 6.447909749 | 7.075746538 | 7.316960287 | 8.002252452 | 8.60936345 | 7.864805194 | 7.141596278 | 0.004345293 | 1.20107356 |
| hsa-miR-182 | 10.1321141 | 11.5740258 | 11.51491126 | 10.87198222 | 9.857825524 | 10.52503135 | 10.35110487 | 7.388878339 | 9.692266772 | 11.35980446 | 0.111172531 | -1.160773128 |
| hsa-miR-182* | 8.214804795 | 8.199181549 | 8.58458675 | 8.110091752 | 11.52645033 | 10.94807516 | 11.88542947 | 10.51914479 | 11.77020962 | 10.53974043 | 1.98E-05 | 2.921008754 |
| hsa-miR-1825 | 7.290940402 | 7.71596199 | 7.246503098 | 7.732676223 | 7.799605422 | 7.316960287 | 7.145677455 | 7.23935985 | 7.117902789 | 8.063395081 | 0.817487188 | -0.049370281 |
| hsa-miR-1826 | 12.25017997 | 11.82137539 | 11.80779765 | 12.89026428 | 9.67357409 | 11.16804527 | 12.82348666 | 9.530893981 | 9.207990433 | 9.760553633 | 0.02201394 | -1.831646975 |
| hsa-miR-1827 | 6.139551352 | 6.520422249 | 6.283551423 | 6.421223299 | 6.399171094 | 6.755555262 | 6.523561956 | 6.422905743 | 6.83541884 | 6.45779126 | 0.084545556 | 0.224546945 |
| hsa-miR-183 | 10.19204618 | 10.28505538 | 10.07614753 | 9.996897126 | 6.155830172 | 6.196725061 | 5.988684687 | 6.0725346 | 6.10433666 | 5.92243574 | 9.26E-09 | -4.064112065 |
| hsa-miR-183* | 6.596935142 | 6.663202279 | 7.353146825 | 6.62935662 | 6.530601415 | 6.465974465 | 6.356672044 | 6.543805176 | 6.584962501 | 7.006746832 | 0.314894708 | -0.229199811 |
| hsa-miR-184 | 5.675251386 | 6.059614856 | 6.091699834 | 5.790511412 | 5.905687849 | 5.716990894 | 5.927185358 | 5.902073579 | 5.9795681 | 5.842978832 | 0.828288755 | -0.025188603 |
| hsa-miR-185 | 10.67410397 | 11.6092711 | 11.67330908 | 11.79445648 | 11.34318572 | 11.5740258 | 7.78496195 | 10.40290547 | 7.635173947 | 10.30138187 | 0.075404417 | -1.597512698 |
| hsa-miR-185* | 7.719731057 | 7.235535607 | 8.255500733 | 7.584210901 | 6.671010241 | 6.316507819 | 6.356672044 | 6.668175869 | 6.584962501 | 6.857980995 | 0.008074258 | -1.122859663 |
| hsa-miR-186 | 8.660174212 | 9.164152708 | 8.137247341 | 9.768101599 | 10.21188829 | 9.768101599 | 10.17816685 | 9.276589716 | 9.783898589 | 9.52277767 | 0.084589807 | 0.857818156 |
| hsa-miR-186* | 7.712870868 | 7.87282876 | 7.614709844 | 7.887525271 | 10.78598367 | 10.28505538 | 10.98113912 | 9.83019827 | 10.79903872 | 9.57799522 | 5.24E-05 | 2.604584712 |
| hsa-miR-187 | 6.995484519 | 10.67410397 | 9.722807531 | 10.39081383 | 7.075746538 | 7.117383367 | 7.400025518 | 6.953032377 | 6.83541884 | 6.329123596 | 0.057079474 | -2.494014091 |
| hsa-miR-187* | 7.664625055 | 7.669593751 | 8.427522371 | 8.908842924 | 13.65089467 | 13.24425976 | 13.74706006 | 12.16091137 | 13.5187515 | 12.46867495 | 5.92E-06 | 4.964112692 |
| hsa-miR-188-3p | 6.952450058 | 7.116863758 | 7.056366761 | 7.343407822 | 7.799605422 | 7.75087444 | 7.145677455 | 6.790511412 | 8.11997861 | 8.324180547 | 0.075819645 | 0.537865881 |
| hsa-miR-188-5p | 8.384567923 | 8.248876343 | 8.481799432 | 8.664269492 | 8.259507895 | 9.595443985 | 9.160249616 | 7.927777962 | 8.300123725 | 7.39145842 | 0.987060808 | -0.005784697 |
| hsa-miR-189:9.1 | 10.10407452 | 9.939285356 | 10.19204618 | 10.54515755 | 13.35328469 | 12.75713995 | 13.50039377 | 11.86546264 | 13.21598794 | 12.32673844 | 4.08E-05 | 2.641360339 |
| hsa-miR-18a | 7.649615459 | 8.803162444 | 8.199181549 | 8.532161085 | 6.05202456 | 5.992088609 | 6.207502459 | 5.988684687 | 6.10433666 | 6.194756854 | 0.002570878 | -2.206131163 |
| hsa-miR-18a* | 10.82033854 | 10.07614753 | 9.981995318 | 8.566054038 | 11.48316077 | 10.67410397 | 11.849366 | 13.43564422 | 11.83543858 | 10.44159552 | 0.028125581 | 1.758750988 |
| hsa-miR-18b | 6.470862199 | 7.441699257 | 6.375908263 | 6.62935662 | 7.075746538 | 6.951867504 | 7.145677455 | 6.953032377 | 7.408117408 | 6.714245518 | 0.298975961 | 0.311991215 |
| hsa-miR-18b* | 13.34410169 | 9.620036498 | 10.46546437 | 11.48316077 | 13.83329532 | 13.5187515 | 13.68986697 | 13.98751273 | 13.85368967 | 13.27035373 | 0.053082674 | 2.464054155 |
| hsa-miR-190 | 7.566054038 | 8.132885254 | 7.842350343 | 8.221103725 | 10.03589879 | 9.644306962 | 10.04384754 | 9.492153626 | 10.07961811 | 9.67047922 | 5.66E-05 | 1.887119035 |
| hsa-miR-190b | 7.600656308 | 9.70822173 | 10.01680829 | 9.676662335 | 6.530601415 | 6.951867504 | 7.400025518 | 7.23935985 | 7.117902789 | 6.714245518 | 0.023514604 | -2.258253399 |
| hsa-miR-191 | 13.10212335 | 12.97835296 | 12.82348666 | 13.50039377 | 7.641690523 | 13.43564422 | 7.78496195 | 6.668175869 | 7.408117408 | 13.43564422 | 0.034381047 | -3.705383486 |
| hsa-miR-191* | 6.641690523 | 7.378511623 | 6.912290583 | 8.366322214 | 6.862947248 | 6.951867504 | 6.523561956 | 8.797337288 | 7.117902789 | 7.006746832 | 0.825769163 | -0.114643133 |
| hsa-miR-192 | 8.018200179 | 10.50809187 | 8.907191126 | 9.412569847 | 7.075746538 | 6.465974465 | 7.145677455 | 11.05372229 | 6.584962501 | 12.93049711 | 0.605888176 | -0.668749861 |
| hsa-miR-192* | 6.36018867 | 6.444600814 | 6.422905743 | 6.918863237 | 8.68580002 | 6.951867504 | 7.400025518 | 7.596935142 | 6.83541884 | 7.925109324 | 0.012533568 | 1.029219776 |
| hsa-miR-193a-3p | 10.78119576 | 10.46546437 | 10.40290547 | 9.741635516 | 9.67357409 | 9.809125002 | 10.98113912 | 9.11204848 | 9.783898589 | 9.353587949 | 0.139175244 | -0.562238075 |
| hsa-miR-193a-5p | 13.75690028 | 13.5347649 | 13.80583454 | 13.38548496 | 11.67330908 | 9.595443985 | 10.45491614 | 9.726899318 | 10.36888837 | 11.39247843 | 0.000170622 | -3.085423619 |
| hsa-miR-193b | 14.11566957 | 12.50061784 | 13.59243368 | 11.59577031 | 6.274261661 | 6.316507819 | 6.356672044 | 6.168922782 | 6.411087227 | 6.194756854 | 0.001251474 | -6.664088118 |
| hsa-miR-193b* | 11.01164658 | 9.644306962 | 10.84172158 | 8.170926525 | 7.484218708 | 6.951867504 | 7.145677455 | 7.1017131 | 7.408117408 | 7.39145842 | 0.025757526 | -2.66997498 |
| hsa-miR-194 | 9.533719071 | 11.42621233 | 10.56367296 | 10.92577695 | 9.570614722 | 8.60936345 | 9.40779885 | 13.38548496 | 10.71020375 | 7.270996061 | 0.430794131 | -0.786601694 |
| hsa-miR-194* | 9.271929844 | 9.068240861 | 8.972692654 | 9.076281167 | 10.94807516 | 10.39687232 | 11.1508262 | 13.12847772 | 11.6092711 | 10.54515755 | 0.002687341 | 2.199160542 |
| hsa-miR-195 | 13.21598794 | 13.92203613 | 13.83329532 | 14.09416843 | 10.03589879 | 11.6092711 | 10.17816685 | 9.276589716 | 10.07961811 | 9.939285356 | 1.40E-05 | -3.579900302 |
| hsa-miR-195* | 9.129283017 | 10.33918193 | 10.06743436 | 10.62561753 | 6.530601415 | 6.465974465 | 6.523561956 | 6.261154673 | 13.12028648 | 7.270996061 | 0.086918283 | -2.344950035 |
| hsa-miR-196a | 12.93049711 | 13.34410169 | 13.3303147 | 13.63051741 | 8.518849829 | 7.75087444 | 8.167418146 | 7.515305605 | 8.597307691 | 7.625708843 | 1.85E-08 | -5.279613635 |
| hsa-miR-196a* | 6.314696526 | 5.966937979 | 6.027905997 | 6.512542955 | 6.155830172 | 5.992088609 | 5.988684687 | 6.0725346 | 6.10433666 | 5.92243574 | 0.28659944 | -0.166202453 |
| hsa-miR-196b | 9.945004816 | 10.96079838 | 10.8622499 | 11.34318572 | 9.14822234 | 8.710462074 | 9.160249616 | 8.812498225 | 9.098821663 | 9.315715658 | 0.006771084 | -1.736814774 |
| hsa-miR-197 | 12.12350713 | 12.04668067 | 12.16091137 | 12.93049711 | 6.671010241 | 6.755555262 | 6.721782768 | 6.668175869 | 6.411087227 | 13.7905725 | 0.012584751 | -4.479035093 |
| hsa-miR-198 | 8.461479447 | 7.540709263 | 8.95419631 | 8.316507819 | 7.484218708 | 7.117383367 | 7.599168993 | 7.388878339 | 7.864805194 | 7.141596278 | 0.048885435 | -0.885548063 |
| hsa-miR-199a*:9.1 | 12.83547806 | 13.94706157 | 13.67325386 | 13.39700607 | 13.59243368 | 13.41629614 | 13.35328469 | 13.76677734 | 13.3303147 | 13.35328469 | 0.98339682 | 0.005531984 |
| hsa-miR-199a-3p,hsa-miR-199b-3p | 13.60579201 | 13.7905725 | 13.7905725 | 13.75690028 | 12.93049711 | 12.08470875 | 13.07437565 | 13.70497352 | 13.36342298 | 13.84325371 | 0.080072911 | -0.569087366 |
| hsa-miR-199a-5p | 11.97799537 | 13.98751273 | 13.73190236 | 13.87028765 | 6.05202456 | 12.78426676 | 6.356672044 | 6.0725346 | 6.250772132 | 6.100136671 | 0.001650704 | -6.122523398 |
| hsa-miR-199b-5p | 10.89231512 | 13.18780053 | 12.65244168 | 12.6077233 | 8.68580002 | 7.921245889 | 8.730809755 | 8.689299161 | 10.45491614 | 7.625708843 | 0.000948166 | -3.65044019 |
| hsa-miR-19a | 6.529820947 | 8.907191126 | 8.562624039 | 10.17816685 | 7.075746538 | 11.28320413 | 7.400025518 | 7.1017131 | 7.408117408 | 6.45779126 | 0.489396281 | -0.756684415 |
| hsa-miR-19a* | 6.452858965 | 6.277984747 | 6.268658955 | 6.136478518 | 6.274261661 | 6.196725061 | 6.356672044 | 8.106955457 | 6.411087227 | 11.67330908 | 0.227126497 | 1.219173125 |
| hsa-miR-19b | 9.766031913 | 10.87198222 | 9.196970898 | 11.32187174 | 9.969170641 | 11.97799537 | 13.73190236 | 9.364134655 | 10.21188829 | 9.116863758 | 0.629979262 | 0.439444986 |
| hsa-miR-19b-1* | 6.517275693 | 6.715618859 | 6.529820947 | 7.180406485 | 6.155830172 | 6.316507819 | 6.523561956 | 10.05521464 | 7.117902789 | 6.857980995 | 0.506591136 | 0.435385899 |
| hsa-miR-19b-2* | 8.122051448 | 7.6395216 | 7.664625055 | 8.289557848 | 9.730979533 | 8.710462074 | 9.507001733 | 9.567385727 | 13.88718253 | 8.324180547 | 0.055880749 | 2.025593036 |
| hsa-miR-200a | 8.514911265 | 12.89026428 | 12.179878 | 12.50061784 | 6.862947248 | 6.316507819 | 6.721782768 | 8.830990481 | 6.83541884 | 6.569855608 | 0.015442734 | -4.498500717 |
| hsa-miR-200a* | 7.388878339 | 10.20371505 | 8.156083076 | 10.74306722 | 12.06062839 | 9.939285356 | 10.94005661 | 10.09882166 | 11.04678297 | 10.06433859 | 0.144601677 | 1.56871634 |
| hsa-miR-200b | 10.52503135 | 13.27035373 | 11.99848466 | 12.21577565 | 9.857825524 | 9.315715658 | 9.920427296 | 10.75200424 | 9.958697703 | 9.760553633 | 0.028905032 | -2.074874005 |
| hsa-miR-200b* | 9.196970898 | 12.08470875 | 11.64029013 | 12.62956153 | 7.799605422 | 8.040837649 | 7.599168993 | 8.522581531 | 8.300123725 | 7.925109324 | 0.019547915 | -3.356645055 |
| hsa-miR-200c | 13.38548496 | 14.61467534 | 14.45914981 | 14.71007995 | 10.44159552 | 12.36194377 | 10.71896091 | 13.34410169 | 12.19614103 | 9.451211112 | 0.003322528 | -2.873355179 |
| hsa-miR-200c* | 6.270528942 | 7.098032083 | 6.784634846 | 6.411087227 | 6.862947248 | 6.316507819 | 6.523561956 | 6.668175869 | 7.117902789 | 6.569855608 | 0.877748283 | 0.035421107 |
| hsa-miR-202 | 6.417852515 | 7.776103988 | 6.547665748 | 6.759555343 | 7.799605422 | 7.117383367 | 7.78496195 | 8.326204751 | 7.117902789 | 7.270996061 | 0.112030806 | 0.694214658 |
| hsa-miR-202* | 7.484218708 | 7.284477118 | 7.370251481 | 7.640606469 | 8.259507895 | 8.894817763 | 8.607700188 | 8.106955457 | 8.300123725 | 9.403012024 | 0.001357832 | 1.150464398 |
| hsa-miR-202*:9.1 | 8.128252152 | 7.892998093 | 9.90523656 | 9.929554316 | 13.10212335 | 13.39700607 | 9.223036338 | 13.5347649 | 14.07082534 | 12.25017997 | 0.003893135 | 3.63231238 |
| hsa-miR-203 | 7.206526016 | 11.36779694 | 10.45491614 | 10.98463207 | 5.837943242 | 5.992088609 | 5.87282876 | 6.0725346 | 6.10433666 | 5.92243574 | 0.023812693 | -4.036439857 |
| hsa-miR-204 | 8.568716189 | 8.910492832 | 8.854556752 | 8.975274788 | 11.71334355 | 10.96079838 | 12.03675744 | 10.52503135 | 11.93475974 | 11.01164658 | 6.39E-05 | 2.5364627 |
| hsa-miR-205 | 12.62956153 | 13.76677734 | 13.65089467 | 13.7905725 | 9.32395546 | 8.25502857 | 13.28896698 | 8.812498225 | 13.27035373 | 8.063395081 | 0.020492691 | -3.290418504 |
| hsa-miR-206 | 9.916028718 | 9.533719071 | 10.72766524 | 9.28748153 | 8.381110294 | 8.040837649 | 8.514911265 | 8.797337288 | 8.453682186 | 7.712870868 | 0.008913091 | -1.549432048 |
| hsa-miR-208a | 5.876516947 | 6.06608919 | 6 | 6.196725061 | 7.641690523 | 7.117383367 | 7.400025518 | 7.388878339 | 10.027906 | 6.45779126 | 0.021610136 | 1.637446368 |
| hsa-miR-208b | 7.655709596 | 6.815063017 | 6.653203362 | 6.964629667 | 9.248164779 | 8.710462074 | 9.32395546 | 9.182890893 | 8.959132577 | 8.405566974 | 0.00045504 | 1.949544049 |
| hsa-miR-20a | 11.54099979 | 13.65089467 | 13.00499288 | 13.45752196 | 8.607700188 | 13.35328469 | 9.40779885 | 12.12350713 | 8.11997861 | 12.19614103 | 0.057935287 | -2.278867242 |
| hsa-miR-20a* | 8.741635516 | 9.644306962 | 9.692266772 | 9.332707934 | 10.21188829 | 6.951867504 | 11.7417619 | 11.25844826 | 11.70111149 | 7.006746832 | 0.647018071 | 0.459241417 |
| hsa-miR-20b | 10.06123617 | 9.996897126 | 9.56414949 | 7.427103287 | 8.968378801 | 8.15810472 | 8.289557848 | 12.90809234 | 8.959132577 | 9.57799522 | 0.826996634 | 0.214530401 |
| hsa-miR-20b* | 6.483815777 | 6.95070169 | 7.062855655 | 7.30833903 | 6.862947248 | 7.117383367 | 6.207502459 | 6.953032377 | 6.584962501 | 6.569855608 | 0.320620905 | -0.235480778 |
| hsa-miR-21 | 14.14972331 | 14.71007995 | 14.61467534 | 14.51607549 | 13.27035373 | 14.61467534 | 13.92203613 | 12.53225851 | 8.838573653 | 14.61467534 | 0.146029608 | -1.532209738 |
| hsa-miR-21* | 8.328226119 | 7.66035251 | 6.830990481 | 7.491853096 | 6.671010241 | 7.316960287 | 6.721782768 | 8.60936345 | 7.408117408 | 7.925109324 | 0.761188976 | -0.135798305 |
| hsa-miR-210 | 8.907191126 | 10.71020375 | 10.50143915 | 10.82033854 | 6.862947248 | 6.465974465 | 7.145677455 | 6.422905743 | 6.83541884 | 6.714245518 | 0.003144555 | -3.493598263 |
| hsa-miR-211 | 6.36719631 | 6.45779126 | 6.483009577 | 6.819540461 | 7.982993575 | 9.101450482 | 8.730809755 | 7.388878339 | 7.117902789 | 8.405566974 | 0.003037412 | 1.589382584 |
| hsa-miR-212 | 9.116863758 | 10.31118066 | 10.06123617 | 7.815703503 | 7.075746538 | 7.316960287 | 7.145677455 | 6.953032377 | 7.117902789 | 6.714245518 | 0.026286478 | -2.272318528 |
| hsa-miR-214 | 13.94706157 | 14.07082534 | 14.14972331 | 14.37188552 | 6.530601415 | 6.755555262 | 7.400025518 | 6.953032377 | 6.584962501 | 7.270996061 | 2.54068E-10 | -7.219011748 |
| hsa-miR-214* | 8.964052011 | 11.01663421 | 11.35980446 | 11.15785217 | 8.68580002 | 7.75087444 | 8.002252452 | 7.515305605 | 8.725877459 | 7.502235115 | 0.012539705 | -2.594194862 |
| hsa-miR-215 | 8.75788999 | 9.924812504 | 10.30138187 | 7.697315122 | 7.273795599 | 9.91064273 | 8.167418146 | 8.003096215 | 7.864805194 | 7.502235115 | 0.191003992 | -1.050017705 |
| hsa-miR-216a | 7.450798854 | 6.697662633 | 6.784634846 | 6.928370323 | 6.862947248 | 7.117383367 | 12.93049711 | 7.661778098 | 6.584962501 | 11.5740258 | 0.165437262 | 1.823232357 |
| hsa-miR-216b | 9.429825157 | 9.364134655 | 9.223036338 | 10.09882166 | 9.730979533 | 9.56414949 | 9.650154214 | 8.689299161 | 9.783898589 | 9.160249616 | 0.715135842 | -0.099166019 |
| hsa-miR-217 | 6.275193334 | 6.318316841 | 6.350497247 | 6.501439145 | 6.862947248 | 7.117383367 | 6.721782768 | 6.790511412 | 6.83541884 | 6.45779126 | 0.002917442 | 0.436277507 |
| hsa-miR-218 | 9.589463893 | 12.36194377 | 12.33776126 | 10.5844928 | 13.00499288 | 6.196725061 | 6.356672044 | 6.422905743 | 6.584962501 | 6.45779126 | 0.021867492 | -3.714407182 |
| hsa-miR-218-1* | 9.403012024 | 9.393819448 | 9.191059215 | 9.589463893 | 11.08261478 | 10.46546437 | 11.30138187 | 10.75200424 | 11.36380623 | 10.19789241 | 0.00026762 | 1.466188675 |
| hsa-miR-218-2* | 6.352264173 | 6.612499946 | 6.569855608 | 6.776762002 | 6.274261661 | 6.092757141 | 6.089582893 | 6.0725346 | 6.250772132 | 7.141596278 | 0.215089396 | -0.257594648 |
| hsa-miR-219-1-3p | 8.185866545 | 6.339850003 | 6.343407822 | 6.431288654 | 6.530601415 | 6.755555262 | 13.00499288 | 10.2701785 | 6.83541884 | 6.857980995 | 0.233610705 | 1.550684727 |
| hsa-miR-219-2-3p | 6.965784285 | 7.263034406 | 7.048214385 | 7.718361626 | 6.530601415 | 7.117383367 | 6.721782768 | 6.422905743 | 7.117902789 | 7.141596278 | 0.103741336 | -0.406819949 |
| hsa-miR-219-5p | 6.130313146 | 9.00365845 | 8.461479447 | 6.374170076 | 6.399171094 | 6.316507819 | 6.523561956 | 6.422905743 | 6.83541884 | 6.329123596 | 0.254547374 | -1.021290438 |
| hsa-miR-22 | 10.87198222 | 11.75768169 | 12.36194377 | 10.79075575 | 6.274261661 | 5.992088609 | 6.930145948 | 6.668175869 | 6.411087227 | 12.06062839 | 0.006197859 | -4.056192907 |
| hsa-miR-22* | 11.51491126 | 10.84172158 | 11.21249639 | 9.230500854 | 6.530601415 | 8.040837649 | 7.145677455 | 11.42621233 | 6.83541884 | 7.925109324 | 0.01595606 | -2.715931352 |
| hsa-miR-220a | 7.273795599 | 7.029563314 | 7.687900522 | 9.638435914 | 7.799605422 | 7.501040996 | 8.167418146 | 7.515305605 | 8.597307691 | 13.24425976 | 0.43150652 | 0.896732433 |
| hsa-miR-220b | 10.11517373 | 7.974988112 | 7.533719071 | 7.839833648 | 8.893756568 | 8.521207816 | 11.2444233 | 12.50061784 | 12.69522829 | 8.168922782 | 0.09130365 | 1.971430792 |
| hsa-miR-220c | 7.427103287 | 6.800252806 | 6.851749041 | 7.168922782 | 7.075746538 | 7.501040996 | 6.721782768 | 6.668175869 | 7.117902789 | 6.857980995 | 0.722447802 | -0.071568653 |
| hsa-miR-221 | 14.04598144 | 14.02323456 | 13.40900634 | 13.67325386 | 14.23044081 | 12.83547806 | 14.18441048 | 14.37188552 | 14.18441048 | 13.90966811 | 0.569000454 | 0.164846529 |
| hsa-miR-221* | 7.056366761 | 9.787902559 | 9.116863758 | 9.885543861 | 6.862947248 | 6.465974465 | 7.400025518 | 6.422905743 | 7.117902789 | 12.36194377 | 0.327241711 | -1.189719312 |
| hsa-miR-222 | 11.849366 | 13.30457949 | 13.19263957 | 13.36342298 | 7.641690523 | 7.625708843 | 13.05706098 | 11.34318572 | 12.89026428 | 8.324180547 | 0.046267692 | -2.780486865 |
| hsa-miR-222* | 7.089582893 | 7.533719071 | 7.275193334 | 7.66035251 | 8.68580002 | 8.950993232 | 8.167418146 | 7.927777962 | 8.453682186 | 9.63499251 | 0.002774596 | 1.247065391 |
| hsa-miR-223 | 11.33220442 | 12.65244168 | 12.26617162 | 12.58282411 | 13.7905725 | 14.23044081 | 12.89026428 | 11.1909358 | 7.635173947 | 14.11566957 | 0.929392953 | 0.100432357 |
| hsa-miR-223* | 7.87036472 | 7.806710718 | 7.697315122 | 6.819540461 | 7.799605422 | 7.501040996 | 8.514911265 | 8.725877459 | 7.635173947 | 11.08261478 | 0.142097533 | 0.994721223 |
| hsa-miR-224 | 13.59243368 | 13.05706098 | 13.88718253 | 11.41494944 | 5.837943242 | 5.790511412 | 6.356672044 | 5.902073579 | 5.581953751 | 13.00499288 | 0.002930863 | -5.908882172 |
| hsa-miR-23a | 14.02323456 | 13.87028765 | 13.92203613 | 13.74706006 | 10.44159552 | 14.01553701 | 11.07487734 | 10.47421294 | 10.71020375 | 14.07082534 | 0.032769611 | -2.092779281 |
| hsa-miR-23a* | 10.51914479 | 9.517472554 | 11.15785217 | 8.548436625 | 8.968378801 | 8.710462074 | 8.889199726 | 8.60936345 | 9.456765088 | 9.315715658 | 0.197680939 | -0.944079068 |
| hsa-miR-23b | 13.73190236 | 13.03414554 | 13.35328469 | 13.03414554 | 6.671010241 | 13.28896698 | 6.089582893 | 6.422905743 | 6.411087227 | 13.38548496 | 0.025825179 | -4.576863193 |
| hsa-miR-23b* | 9.068240861 | 8.189330401 | 8.232660757 | 8.128252152 | 8.518849829 | 8.040837649 | 8.514911265 | 7.753885283 | 8.838573653 | 8.324180547 | 0.798527356 | -0.072748005 |
| hsa-miR-24 | 13.57510193 | 13.50039377 | 13.61622719 | 13.70497352 | 11.95728346 | 13.45752196 | 9.507001733 | 9.252665432 | 9.692266772 | 13.50039377 | 0.03324599 | -2.371318581 |
| hsa-miR-24-1* | 7.708049252 | 9.14822234 | 9.435878577 | 9.05853297 | 7.799605422 | 7.501040996 | 8.730809755 | 8.326204751 | 7.864805194 | 7.141596278 | 0.088083525 | -0.943660386 |
| hsa-miR-24-2* | 13.00499288 | 10.61369739 | 10.68088692 | 10.8622499 | 11.99848466 | 11.49355527 | 12.65244168 | 13.55601501 | 12.40167953 | 11.79445648 | 0.178428762 | 1.025648664 |
| hsa-miR-25 | 13.90966811 | 14.11566957 | 13.96936853 | 13.98751273 | 13.76677734 | 14.14972331 | 9.958697703 | 13.47772033 | 13.26034622 | 14.27431991 | 0.253859117 | -0.847623936 |
| hsa-miR-25* | 9.318768743 | 9.435878577 | 8.933690655 | 8.321026129 | 8.893756568 | 8.710462074 | 10.11517373 | 11.48316077 | 8.725877459 | 9.116863758 | 0.357074414 | 0.505208035 |
| hsa-miR-26a | 13.88718253 | 13.96936853 | 13.85368967 | 13.94706157 | 9.792139552 | 13.98751273 | 8.289557848 | 9.403012024 | 9.958697703 | 13.94706157 | 0.029443405 | -3.017995339 |
| hsa-miR-26a-1* | 5.831623937 | 6.236492618 | 5.944858446 | 5.987548259 | 6.399171094 | 6.092757141 | 6.207502459 | 6.168922782 | 6.250772132 | 10.78598367 | 0.254053138 | 0.984054065 |
| hsa-miR-26a-2* | 6.201633861 | 7.964052011 | 8.137247341 | 6.447909749 | 8.800252806 | 8.25502857 | 8.607700188 | 8.326204751 | 8.453682186 | 6.45779126 | 0.168065744 | 0.96239922 |
| hsa-miR-26b | 12.40167953 | 13.70497352 | 13.70497352 | 14.02323456 | 13.24425976 | 13.55601501 | 6.356672044 | 6.332707934 | 6.411087227 | 12.55837288 | 0.057771757 | -3.715529472 |
| hsa-miR-26b* | 9.676662335 | 11.23595438 | 11.36779694 | 11.2444233 | 6.530601415 | 6.755555262 | 6.721782768 | 11.30691611 | 7.635173947 | 13.47772033 | 0.140660205 | -2.143250934 |
| hsa-miR-27a | 13.19746245 | 14.23044081 | 13.98751273 | 14.27431991 | 6.862947248 | 12.19614103 | 6.523561956 | 6.953032377 | 6.584962501 | 6.45779126 | 0.000705524 | -6.326027912 |
| hsa-miR-27a* | 9.66035251 | 10.15000109 | 10.04384754 | 10.027906 | 11.02873489 | 9.715533064 | 9.32395546 | 9.233739497 | 9.574309691 | 8.543805176 | 0.298910083 | -0.400513822 |
| hsa-miR-27b | 11.77020962 | 13.59243368 | 13.41629614 | 13.73190236 | 9.412569847 | 8.60936345 | 9.821455119 | 9.152284842 | 9.574309691 | 9.802758679 | 0.001600595 | -3.732253511 |
| hsa-miR-27b* | 9.09037711 | 8.933690655 | 8.053654418 | 9.779883475 | 10.35110487 | 9.824322351 | 10.39687232 | 9.318768743 | 10.42500604 | 9.276589716 | 0.065491638 | 0.967709259 |
| hsa-miR-28-3p | 11.52645033 | 11.71334355 | 11.47126876 | 11.51491126 | 8.151523994 | 12.69522829 | 8.413204789 | 12.75713995 | 8.300123725 | 8.248876343 | 0.113959292 | -1.795477296 |
| hsa-miR-28-5p | 11.95728346 | 13.36342298 | 13.12028648 | 13.60579201 | 6.862947248 | 7.117383367 | 7.599168993 | 6.953032377 | 8.597307691 | 9.044667331 | 1.10E-05 | -5.315945065 |
| hsa-miR-296-3p | 7.6727789 | 9.306175642 | 9.096188046 | 6.559950613 | 9.248164779 | 7.316960287 | 9.32395546 | 7.753885283 | 9.852139328 | 8.75788999 | 0.499546185 | 0.550059221 |
| hsa-miR-296-5p | 6.944858446 | 7.544578117 | 7.48984796 | 8.490650349 | 7.982993575 | 7.625708843 | 8.289557848 | 7.661778098 | 8.300123725 | 7.006746832 | 0.629254994 | 0.193667769 |
| hsa-miR-297 | 8.910492832 | 8.437544026 | 8.263034406 | 8.536441505 | 9.67357409 | 9.024308597 | 9.87175126 | 9.152284842 | 9.783898589 | 8.647638302 | 0.009699462 | 0.822031088 |
| hsa-miR-298 | 9.171927354 | 9.353587949 | 9.242578689 | 9.601399391 | 10.1365426 | 9.595443985 | 10.00070427 | 8.858291887 | 10.36888837 | 9.451211112 | 0.152382445 | 0.392807024 |
| hsa-miR-299-3p | 8.063395081 | 7.617651119 | 7.84862294 | 7.614709844 | 6.530601415 | 7.501040996 | 6.207502459 | 6.422905743 | 6.250772132 | 7.270996061 | 0.003348935 | -1.088791612 |
| hsa-miR-299-5p | 8.554972496 | 8.356672044 | 7.146186791 | 9.152284842 | 6.399171094 | 6.465974465 | 6.523561956 | 8.326204751 | 6.584962501 | 11.86546264 | 0.555251327 | -0.608306142 |
| hsa-miR-29a | 11.1909358 | 13.26034622 | 12.89026428 | 13.05706098 | 7.484218708 | 8.344073938 | 7.78496195 | 6.543805176 | 6.83541884 | 7.502235115 | 0.000249156 | -5.183866197 |
| hsa-miR-29a* | 7.735725245 | 10.94807516 | 9.334385031 | 10.84172158 | 6.862947248 | 6.609548139 | 6.930145948 | 6.668175869 | 6.10433666 | 6.714245518 | 0.025408378 | -3.066743524 |
| hsa-miR-29b | 11.80779765 | 13.57510193 | 13.34410169 | 13.12847772 | 12.26617162 | 13.34410169 | 6.523561956 | 9.152284842 | 6.83541884 | 13.12847772 | 0.084979997 | -2.755533633 |
| hsa-miR-29b-1* | 11.72144102 | 11.05372229 | 11.32187174 | 10.52503135 | 8.259507895 | 8.25502857 | 8.002252452 | 7.388878339 | 8.11997861 | 10.31118066 | 0.000475771 | -2.76604551 |
| hsa-miR-29b-2* | 10.08308037 | 8.92659251 | 8.502235115 | 9.171927354 | 6.399171094 | 7.117383367 | 7.599168993 | 7.661778098 | 7.864805194 | 6.569855608 | 0.003064708 | -1.968931778 |
| hsa-miR-29c | 9.6121313 | 12.1027959 | 11.7417619 | 11.50858711 | 9.52277767 | 8.437544026 | 9.160249616 | 9.403012024 | 10.07961811 | 7.625708843 | 0.018344356 | -2.203167337 |
| hsa-miR-29c* | 11.7417619 | 12.12350713 | 11.71334355 | 11.72144102 | 8.381110294 | 9.595443985 | 8.289557848 | 7.857047916 | 8.11997861 | 9.250416861 | 2.81E-05 | -3.242754148 |
| hsa-miR-300 | 7.162894799 | 7.511752654 | 7.141596278 | 7.216745858 | 9.049167872 | 8.437544026 | 8.413204789 | 8.106955457 | 10.36888837 | 10.37585398 | 0.005692706 | 1.867021684 |
| hsa-miR-301a | 9.353587949 | 9.487840034 | 9.182890893 | 9.336060181 | 10.45491614 | 10.08832449 | 12.78426676 | 9.889656058 | 12.08470875 | 10.45491614 | 0.019943604 | 1.619369958 |
| hsa-miR-301b | 6.085339669 | 5.851749041 | 5.760220946 | 5.94016675 | 5.980710829 | 6.092757141 | 5.988684687 | 8.897542973 | 6.10433666 | 6.100136671 | 0.251804502 | 0.617992391 |
| hsa-miR-302a | 6.029011087 | 6.373300197 | 6.175923742 | 7.017365205 | 6.530601415 | 6.316507819 | 6.523561956 | 6.332707934 | 6.411087227 | 6.857980995 | 0.700288998 | 0.096507833 |
| hsa-miR-302a* | 6.582706527 | 6.754219433 | 6.903279342 | 6.951867504 | 6.274261661 | 7.117383367 | 6.721782768 | 6.668175869 | 6.10433666 | 7.141596278 | 0.530993155 | -0.126762101 |
| hsa-miR-302b | 6.800252806 | 7.388878339 | 6.139551352 | 6.255500733 | 6.399171094 | 6.951867504 | 6.523561956 | 6.422905743 | 6.411087227 | 6.714245518 | 0.815025239 | -0.075572634 |
| hsa-miR-302b* | 13.5347649 | 9.77016832 | 10.80727441 | 12.53225851 | 13.85368967 | 13.60579201 | 13.84325371 | 13.94706157 | 13.96936853 | 13.63051741 | 0.084264038 | 2.14716395 |
| hsa-miR-302c | 6.641690523 | 6.21916852 | 6.283551423 | 6.292781749 | 7.273795599 | 7.921245889 | 7.400025518 | 7.1017131 | 7.635173947 | 7.799605422 | 9.09E-05 | 1.162628525 |
| hsa-miR-302c* | 7.242221395 | 6.697662633 | 6.75087444 | 7.180406485 | 9.14822234 | 9.101450482 | 9.40779885 | 8.689299161 | 8.959132577 | 9.160249616 | 2.42E-05 | 2.109900933 |
| hsa-miR-302d | 14.41700199 | 9.589463893 | 10.82631014 | 11.95728346 | 14.27431991 | 14.04598144 | 14.14972331 | 14.23044081 | 14.14972331 | 13.98751273 | 0.097754562 | 2.442102048 |
| hsa-miR-302d* | 6.731997787 | 6.651769271 | 7.40599236 | 6.819540461 | 7.075746538 | 6.609548139 | 7.599168993 | 7.661778098 | 7.117902789 | 8.647638302 | 0.140053686 | 0.54963884 |
| hsa-miR-302e | 6.224001674 | 5.980710829 | 7.640606469 | 6.004501392 | 6.671010241 | 6.755555262 | 6.930145948 | 6.790511412 | 7.408117408 | 6.194756854 | 0.484416427 | 0.329227763 |
| hsa-miR-302f | 5.716990894 | 5.760220946 | 5.794415866 | 5.820178962 | 6.05202456 | 5.911691582 | 6.089582893 | 6.168922782 | 6.10433666 | 5.842978832 | 0.002816478 | 0.255304551 |
| hsa-miR-30a | 11.71334355 | 13.21598794 | 12.01300855 | 12.179878 | 5.837943242 | 5.992088609 | 5.927185358 | 5.988684687 | 5.9795681 | 6.100136671 | 0.00026665 | -6.309620067 |
| hsa-miR-30a* | 10.82631014 | 12.45213826 | 12.08470875 | 12.08470875 | 6.274261661 | 6.465974465 | 6.523561956 | 6.543805176 | 6.83541884 | 6.194756854 | 0.000337384 | -5.389003316 |
| hsa-miR-30b | 11.55588324 | 13.40900634 | 13.24425976 | 13.5187515 | 6.530601415 | 13.15864124 | 6.207502459 | 5.902073579 | 6.250772132 | 8.814422247 | 0.005292016 | -5.121306365 |
| hsa-miR-30b* | 7.987548259 | 7.166916252 | 7.192785959 | 7.159871337 | 7.641690523 | 7.117383367 | 7.78496195 | 6.422905743 | 8.453682186 | 8.324180547 | 0.525114944 | 0.247353601 |
| hsa-miR-30c | 13.55601501 | 14.04598144 | 14.01553701 | 13.90966811 | 11.61895898 | 12.55837288 | 11.93475974 | 10.4871366 | 11.89958351 | 10.98463207 | 0.000308706 | -2.301226433 |
| hsa-miR-30c-1* | 10.59395128 | 9.766031913 | 10.28505538 | 10.50809187 | 7.273795599 | 7.316960287 | 6.721782768 | 6.953032377 | 7.117902789 | 6.569855608 | 1.12E-05 | -3.296061039 |
| hsa-miR-30c-2* | 8.15936681 | 8.286326727 | 8.15936681 | 9.107217076 | 7.484218708 | 7.921245889 | 8.167418146 | 6.422905743 | 6.584962501 | 8.814422247 | 0.089319323 | -0.86220715 |
| hsa-miR-30d | 13.84325371 | 14.27431991 | 14.27431991 | 14.23044081 | 12.28004576 | 13.03414554 | 7.78496195 | 7.388878339 | 7.864805194 | 12.75713995 | 0.016646103 | -3.97058746 |
| hsa-miR-30d* | 9.939285356 | 9.67047922 | 9.36128586 | 9.528063348 | 11.42621233 | 10.56985561 | 11.47126876 | 10.89231512 | 11.59577031 | 10.68088692 | 0.000154498 | 1.481273063 |
| hsa-miR-30e | 10.28505538 | 11.2444233 | 10.14095755 | 11.23595438 | 11.33220442 | 10.82033854 | 11.55588324 | 10.71020375 | 11.54846071 | 10.97763769 | 0.258823486 | 0.430857075 |
| hsa-miR-30e* | 12.33776126 | 13.85368967 | 13.5187515 | 13.61622719 | 11.15785217 | 11.71334355 | 11.50858711 | 10.59395128 | 11.2062818 | 13.40900634 | 0.010440284 | -1.733437028 |
| hsa-miR-31 | 8.947783026 | 11.67330908 | 10.39081383 | 12.01300855 | 7.075746538 | 8.437544026 | 13.65089467 | 11.54099979 | 7.408117408 | 9.824322351 | 0.407024152 | -1.099957825 |
| hsa-miR-31* | 7.328226119 | 11.139935 | 10.71020375 | 11.02160465 | 7.641690523 | 13.05706098 | 6.930145948 | 6.953032377 | 12.33776126 | 9.116863758 | 0.634922922 | -0.710566571 |
| hsa-miR-32 | 5.876516947 | 6.789859641 | 7.011227255 | 5.961160258 | 6.671010241 | 6.316507819 | 6.523561956 | 6.422905743 | 6.584962501 | 6.329123596 | 0.837877731 | 0.064987617 |
| hsa-miR-32* | 5.831623937 | 6.45779126 | 5.894817763 | 6.529820947 | 6.05202456 | 5.911691582 | 6.089582893 | 6.0725346 | 6.411087227 | 5.842978832 | 0.594120208 | -0.115196861 |
| hsa-miR-320d,hsa-miR-320b,hsa-miR-320a,hsa-miR-320c | 13.96936853 | 14.01553701 | 14.09416843 | 14.07082534 | 13.12847772 | 14.09416843 | 10.11517373 | 9.595443985 | 6.411087227 | 13.67325386 | 0.06601207 | -2.867874005 |
| hsa-miR-323-3p | 7.312429206 | 9.107217076 | 6.68580002 | 6.896029603 | 7.484218708 | 7.316960287 | 8.167418146 | 7.927777962 | 7.864805194 | 10.01680829 | 0.390520354 | 0.629295788 |
| hsa-miR-323-5p | 6.824640581 | 6.74281467 | 6.794415866 | 7.115303806 | 7.982993575 | 8.15810472 | 7.599168993 | 7.927777962 | 8.300123725 | 7.502235115 | 0.000143262 | 1.042440284 |
| hsa-miR-324-3p | 11.47126876 | 13.15864124 | 12.90809234 | 12.82348666 | 8.151523994 | 8.60936345 | 8.730809755 | 10.17816685 | 8.838573653 | 8.647638302 | 0.000203356 | -3.731026251 |
| hsa-miR-324-5p | 12.75713995 | 11.54099979 | 12.93049711 | 13.35328469 | 14.51607549 | 14.41700199 | 14.41700199 | 14.14972331 | 14.37188552 | 14.37188552 | 0.020230451 | 1.728448585 |
| hsa-miR-325 | 6.510961919 | 6.636624621 | 6.731997787 | 7.001126665 | 7.075746538 | 8.040837649 | 7.145677455 | 7.1017131 | 7.117902789 | 8.248876343 | 0.019671329 | 0.734947898 |
| hsa-miR-326 | 11.21249639 | 10.21188829 | 10.16440416 | 9.188712465 | 6.530601415 | 6.092757141 | 6.523561956 | 8.326204751 | 5.9795681 | 6.45779126 | 0.000373471 | -3.542627888 |
| hsa-miR-328 | 11.6092711 | 11.49355527 | 11.95728346 | 12.36194377 | 5.752213368 | 5.716990894 | 5.87282876 | 6.668175869 | 5.804776378 | 5.92243574 | 2.59E-07 | -5.899276565 |
| hsa-miR-329 | 8.300123725 | 10.9918761 | 10.78119576 | 8.554972496 | 8.259507895 | 7.75087444 | 6.930145948 | 6.790511412 | 6.584962501 | 7.625708843 | 0.03937593 | -2.333423514 |
| hsa-miR-330-3p | 8.427522371 | 8.986979709 | 8.275193334 | 8.003939486 | 8.607700188 | 8.15810472 | 8.730809755 | 8.452035274 | 8.725877459 | 7.006746832 | 0.684548521 | -0.143196354 |
| hsa-miR-330-5p | 6.789859641 | 6.350497247 | 6.422905743 | 6.663913842 | 8.893756568 | 8.819061399 | 8.986979709 | 8.763876285 | 8.959132577 | 8.063395081 | 1.56E-06 | 2.190906152 |
| hsa-miR-331-3p | 11.72970571 | 11.21249639 | 11.23595438 | 11.2062818 | 8.381110294 | 7.921245889 | 6.523561956 | 7.388878339 | 8.300123725 | 7.270996061 | 1.00E-05 | -3.715123524 |
| hsa-miR-331-5p | 7.083213368 | 7.094869433 | 6.957682486 | 7.115303806 | 6.671010241 | 7.117383367 | 7.145677455 | 7.1017131 | 6.83541884 | 6.45779126 | 0.199196699 | -0.174601563 |
| hsa-miR-335 | 9.90523656 | 9.58129475 | 9.967802645 | 8.382840144 | 6.671010241 | 8.25502857 | 7.78496195 | 8.405566974 | 6.83541884 | 10.11517373 | 0.051058019 | -1.44810014 |
| hsa-miR-335* | 12.28004576 | 12.03675744 | 11.97799537 | 9.188712465 | 7.075746538 | 8.25502857 | 13.59243368 | 7.388878339 | 8.725877459 | 13.10212335 | 0.26258408 | -1.680863104 |
| hsa-miR-337:9.1 | 6.069315495 | 6.476543706 | 6.175923742 | 6.348728154 | 6.399171094 | 9.315715658 | 13.12028648 | 11.32187174 | 11.42621233 | 10.87198222 | 0.006929675 | 4.141578813 |
| hsa-miR-337-3p | 8.381110294 | 10.80727441 | 10.04916787 | 8.15936681 | 6.530601415 | 7.501040996 | 6.721782768 | 6.0725346 | 6.411087227 | 6.569855608 | 0.019705603 | -2.714746078 |
| hsa-miR-337-5p | 6.374170076 | 6.697662633 | 6.417852515 | 6.715618859 | 9.248164779 | 8.437544026 | 9.40779885 | 8.106955457 | 9.958697703 | 8.647638302 | 0.000184854 | 2.416473832 |
| hsa-miR-338-3p | 7.572131751 | 8.710462074 | 7.533719071 | 9.312656099 | 7.273795599 | 8.040837649 | 6.721782768 | 6.543805176 | 7.117902789 | 8.647638302 | 0.154081689 | -0.891281868 |
| hsa-miR-338-5p | 6.36018867 | 6.623515741 | 6.632995197 | 6.857980995 | 8.607700188 | 7.75087444 | 9.160249616 | 7.753885283 | 9.574309691 | 8.589838376 | 0.000787692 | 1.954139448 |
| hsa-miR-339-3p | 7.136478518 | 8.088523257 | 6.23457796 | 8.1017131 | 5.905687849 | 5.992088609 | 5.927185358 | 5.988684687 | 5.888743249 | 6.001126665 | 0.048298068 | -1.439737139 |
| hsa-miR-339-5p | 10.31118066 | 9.846430632 | 9.945004816 | 10.31118066 | 5.837943242 | 5.790511412 | 5.786596362 | 5.861707287 | 5.716990894 | 6.194756854 | 8.39E-07 | -4.238698184 |
| hsa-miR-33a | 11.88542947 | 7.933100475 | 9.342296946 | 9.70822173 | 12.3835693 | 6.755555262 | 13.36342298 | 13.3303147 | 12.55837288 | 6.329123596 | 0.51872815 | 1.069464299 |
| hsa-miR-33a* | 8.476138625 | 6.965784285 | 7.071462363 | 7.123604124 | 8.151523994 | 8.437544026 | 9.160249616 | 9.364134655 | 6.584962501 | 8.248876343 | 0.127941208 | 0.915301173 |
| hsa-miR-33b | 5.923624611 | 6.636624621 | 6.667466405 | 6.136478518 | 7.075746538 | 6.951867504 | 6.930145948 | 6.422905743 | 7.117902789 | 6.329123596 | 0.090623712 | 0.463566814 |
| hsa-miR-33b* | 6.288173968 | 6.190812368 | 6.250772132 | 6.311975314 | 6.862947248 | 6.609548139 | 10.56985561 | 6.422905743 | 6.584962501 | 6.45779126 | 0.197477043 | 0.990901638 |
| hsa-miR-340 | 7.414473836 | 8.518849829 | 7.933100475 | 7.892998093 | 7.799605422 | 8.710462074 | 8.289557848 | 8.763876285 | 7.864805194 | 8.961594391 | 0.172985984 | 0.458461311 |
| hsa-miR-340* | 9.421644094 | 8.897542973 | 9.252665432 | 8.574025798 | 6.530601415 | 11.72144102 | 6.523561956 | 6.953032377 | 6.83541884 | 11.23595438 | 0.503423084 | -0.73646791 |
| hsa-miR-342-3p | 11.5740258 | 12.33776126 | 11.75768169 | 11.97799537 | 8.518849829 | 12.04668067 | 8.730809755 | 6.953032377 | 8.453682186 | 11.82137539 | 0.030130895 | -2.491127659 |
| hsa-miR-342-5p | 11.18090371 | 11.55588324 | 11.42059188 | 12.03675744 | 8.518849829 | 13.75690028 | 8.889199726 | 8.486231678 | 10.07961811 | 8.405566974 | 0.081558561 | -1.859139635 |
| hsa-miR-345 | 7.170926525 | 7.116863758 | 6.502235115 | 6.300123725 | 6.671010241 | 6.465974465 | 7.145677455 | 6.422905743 | 7.117902789 | 6.329123596 | 0.770820142 | -0.080438232 |
| hsa-miR-345:9.1 | 7.458201525 | 8.388447876 | 8.280306806 | 8.346513733 | 9.248164779 | 8.437544026 | 9.082149041 | 8.566054038 | 9.098821663 | 8.814422247 | 0.031449729 | 0.756158481 |
| hsa-miR-346 | 13.40900634 | 8.490650349 | 10.09354961 | 10.82631014 | 13.84325371 | 13.73190236 | 13.85368967 | 14.02323456 | 13.90966811 | 13.61622719 | 0.055226371 | 3.124783489 |
| hsa-miR-34a | 10.44159552 | 13.39700607 | 12.62956153 | 13.10212335 | 7.273795599 | 7.117383367 | 7.78496195 | 7.1017131 | 7.408117408 | 8.498849207 | 0.003206307 | -4.861768179 |
| hsa-miR-34a* | 7.726558779 | 8.735725245 | 8.128252152 | 8.476138625 | 8.518849829 | 8.15810472 | 8.986979709 | 9.567385727 | 11.66540254 | 8.498849207 | 0.136597138 | 0.965926589 |
| hsa-miR-34b | 6.517275693 | 8.611024797 | 7.250772132 | 9.196970898 | 5.837943242 | 5.992088609 | 5.786596362 | 5.902073579 | 5.888743249 | 6.100136671 | 0.048180102 | -1.976080595 |
| hsa-miR-34b* | 7.103287808 | 7.695228291 | 8.250298418 | 7.365884968 | 9.857825524 | 6.092757141 | 6.523561956 | 6.790511412 | 11.47722702 | 9.353587949 | 0.453057005 | 0.745570296 |
| hsa-miR-34c-3p | 7.378511623 | 8.008988783 | 6.085339669 | 7.594324604 | 8.259507895 | 8.15810472 | 8.514911265 | 8.522581531 | 8.725877459 | 8.168922782 | 0.06980678 | 1.124859772 |
| hsa-miR-34c-5p | 6.36719631 | 9.053654418 | 7.611024797 | 7.78496195 | 7.484218708 | 7.316960287 | 7.78496195 | 7.23935985 | 8.300123725 | 6.857980995 | 0.742465812 | -0.206941783 |
| hsa-miR-361-3p | 9.250416861 | 9.893756568 | 9.741635516 | 9.05853297 | 8.151523994 | 9.809125002 | 8.167418146 | 7.661778098 | 8.453682186 | 9.688862232 | 0.081711551 | -0.830687203 |
| hsa-miR-361-5p | 11.30691611 | 11.15785217 | 11.06709819 | 11.68877483 | 9.14822234 | 11.88542947 | 8.889199726 | 8.25502857 | 9.328338334 | 8.324180547 | 0.013381039 | -2.000093828 |
| hsa-miR-362-3p | 9.250416861 | 10.5844928 | 10.11517373 | 9.787902559 | 11.27653156 | 11.3810562 | 11.34318572 | 10.39081383 | 11.36380623 | 11.50858711 | 0.010814791 | 1.276166955 |
| hsa-miR-362-5p | 9.044667331 | 8.068509595 | 6.0725346 | 9.152284842 | 8.607700188 | 7.501040996 | 8.889199726 | 7.388878339 | 8.725877459 | 7.006746832 | 0.938238531 | -0.064591835 |
| hsa-miR-363 | 10.38046107 | 9.779883475 | 9.638435914 | 8.87036472 | 10.06743436 | 9.024308597 | 9.507001733 | 9.492153626 | 10.21188829 | 9.353587949 | 0.8785989 | -0.057890533 |
| hsa-miR-363* | 6.040015679 | 6 | 5.965784285 | 6.276124405 | 8.381110294 | 7.921245889 | 8.730809755 | 8.566054038 | 9.328338334 | 7.270996061 | 0.000348561 | 2.295944636 |
| hsa-miR-365 | 9.601399391 | 8.405566974 | 9.631540867 | 7.850499414 | 8.151523994 | 8.25502857 | 8.413204789 | 7.927777962 | 7.635173947 | 8.324180547 | 0.18793992 | -0.754436693 |
| hsa-miR-367 | 6.501439145 | 6.918863237 | 6.897240426 | 7.244125943 | 8.151523994 | 6.951867504 | 7.400025518 | 6.953032377 | 7.635173947 | 6.714245518 | 0.161904252 | 0.410560955 |
| hsa-miR-367* | 8.595070954 | 8.244125943 | 8.115563915 | 8.397460726 | 10.98113912 | 10.79075575 | 11.139935 | 10.21188829 | 11.02517418 | 10.10145048 | 5.56E-06 | 2.370335085 |
| hsa-miR-369-3p | 6.249824549 | 7.199672345 | 8.271463028 | 7.062855655 | 13.36342298 | 8.040837649 | 9.32395546 | 12.94553528 | 9.328338334 | 8.814422247 | 0.018905368 | 3.106798098 |
| hsa-miR-369-5p | 8.15936681 | 9.336060181 | 8.287943192 | 8.199181549 | 10.2701785 | 9.768101599 | 10.45491614 | 9.97053734 | 10.42500604 | 9.52277767 | 0.004949636 | 1.572948282 |
| hsa-miR-370 | 9.846430632 | 11.03981011 | 10.65391987 | 8.263034406 | 9.484420132 | 8.819061399 | 9.258330473 | 8.326204751 | 9.456765088 | 8.75788999 | 0.228417608 | -0.933686784 |
| hsa-miR-371-3p | 7.064473329 | 8.093285504 | 7.608069968 | 7.469234794 | 7.273795599 | 9.101450482 | 6.930145948 | 7.388878339 | 7.408117408 | 9.996897126 | 0.431212071 | 0.457781585 |
| hsa-miR-371-5p | 8.510170751 | 8.894817763 | 8.680535344 | 8.612499946 | 10.50476934 | 9.852139328 | 9.821455119 | 9.276589716 | 9.852139328 | 9.044667331 | 0.002885977 | 1.05078741 |
| hsa-miR-372 | 8.60936345 | 8.117902789 | 7.943686952 | 8.15936681 | 10.9918761 | 10.31955923 | 10.81382127 | 10.42500604 | 10.9544508 | 10.48290877 | 4.62E-06 | 2.4570237 |
| hsa-miR-373 | 5.876516947 | 5.851749041 | 5.988684687 | 5.987548259 | 6.155830172 | 5.856736756 | 5.786596362 | 6.168922782 | 6.250772132 | 5.92243574 | 0.297215995 | 0.097424257 |
| hsa-miR-373* | 5.988684687 | 6.029011087 | 7.031770114 | 6.004501392 | 5.905687849 | 6.092757141 | 6.089582893 | 6.168922782 | 6.10433666 | 6.569855608 | 0.711789647 | -0.108301331 |
| hsa-miR-374a | 9.885543861 | 10.19204618 | 10.17816685 | 9.6121313 | 11.41494944 | 10.82631014 | 11.80779765 | 11.20939244 | 11.75768169 | 10.46546437 | 0.001145468 | 1.279960572 |
| hsa-miR-374a* | 8.132885254 | 8.643495471 | 8.440453944 | 8.523953939 | 9.049167872 | 8.60936345 | 8.889199726 | 8.106955457 | 8.597307691 | 9.63499251 | 0.151932892 | 0.379300632 |
| hsa-miR-374b | 6.495855027 | 8.416164165 | 5.977279923 | 5.894817763 | 6.05202456 | 5.992088609 | 5.87282876 | 5.902073579 | 5.9795681 | 5.842978832 | 0.289431005 | -0.755768813 |
| hsa-miR-374b* | 6.019034669 | 6.199672345 | 6.085339669 | 6.121015401 | 6.399171094 | 5.992088609 | 6.089582893 | 6.0725346 | 6.10433666 | 6.194756854 | 0.618177853 | 0.035812931 |
| hsa-miR-375 | 10.027906 | 13.07437565 | 12.6077233 | 13.57510193 | 13.70497352 | 6.951867504 | 7.400025518 | 13.07437565 | 13.55601501 | 13.75690028 | 0.574552844 | -0.913917138 |
| hsa-miR-376a | 8.521207816 | 8.532161085 | 8.618385502 | 7.600656308 | 13.61622719 | 8.437544026 | 13.38548496 | 12.03675744 | 8.959132577 | 12.96155822 | 0.016686906 | 3.248014725 |
| hsa-miR-376a* | 6.121015401 | 7.461070114 | 7.180406485 | 6.004501392 | 6.274261661 | 5.992088609 | 6.207502459 | 6.261154673 | 6.10433666 | 6.329123596 | 0.270668851 | -0.497003738 |
| hsa-miR-376a*:9.1 | 9.024308597 | 7.586464526 | 8.338067798 | 9.824322351 | 13.40900634 | 13.27035373 | 11.95728346 | 11.65745244 | 8.11997861 | 12.67185062 | 0.010211463 | 3.154363383 |
| hsa-miR-376b | 8.397460726 | 8.964052011 | 9.107217076 | 9.809125002 | 11.64029013 | 10.34751064 | 10.94005661 | 12.78426676 | 11.18090371 | 11.64029013 | 0.000760943 | 2.352755961 |
| hsa-miR-376c | 7.78496195 | 10.36654079 | 8.763876285 | 10.48290877 | 13.19746245 | 11.64029013 | 12.62956153 | 12.40167953 | 13.5347649 | 11.88542947 | 0.009587115 | 3.198626052 |
| hsa-miR-377 | 8.300123725 | 8.522581531 | 8.947783026 | 8.437544026 | 12.97835296 | 13.07437565 | 11.00562455 | 11.02873489 | 10.54515755 | 10.16440416 | 0.001758033 | 2.914100216 |
| hsa-miR-377* | 7.083213368 | 7.55688919 | 7.017365205 | 7.964052011 | 7.484218708 | 7.75087444 | 6.721782768 | 6.953032377 | 7.408117408 | 7.925109324 | 0.916788495 | -0.031524106 |
| hsa-miR-378 | 12.08470875 | 12.83547806 | 12.97835296 | 11.99848466 | 6.862947248 | 11.50858711 | 7.145677455 | 6.543805176 | 7.408117408 | 8.589838376 | 0.001342113 | -4.46442731 |
| hsa-miR-378* | 11.28320413 | 10.01680829 | 10.42311591 | 8.30560579 | 13.50039377 | 8.521207816 | 10.52209107 | 9.207990433 | 9.328338334 | 13.19746245 | 0.530254979 | 0.705730447 |
| hsa-miR-379 | 8.6387979 | 10.04384754 | 9.487840034 | 8.582706527 | 11.59577031 | 13.70497352 | 9.920427296 | 13.39700607 | 13.61622719 | 7.502235115 | 0.064438161 | 2.434475249 |
| hsa-miR-379* | 5.988684687 | 6.697662633 | 8.506605116 | 6.338959175 | 6.862947248 | 6.755555262 | 6.207502459 | 6.668175869 | 6.83541884 | 7.625708843 | 0.928035858 | -0.057093149 |
| hsa-miR-380 | 7.532161085 | 7.735725245 | 8.808964175 | 10.08308037 | 13.5347649 | 13.21598794 | 13.27035373 | 12.58282411 | 13.75690028 | 11.77020962 | 0.001468379 | 4.481857379 |
| hsa-miR-380* | 7.353146825 | 7.561478892 | 7.625708843 | 7.653203362 | 12.14063808 | 11.03981011 | 9.40779885 | 8.763876285 | 9.328338334 | 10.51914479 | 0.003473694 | 2.651549928 |
| hsa-miR-381 | 7.947198584 | 10.50143915 | 9.57799522 | 9.429825157 | 9.32395546 | 10.24257869 | 8.413204789 | 8.003096215 | 8.597307691 | 10.68088692 | 0.829015551 | -0.153942899 |
| hsa-miR-382 | 8.324180547 | 10.68088692 | 9.939285356 | 8.481799432 | 6.05202456 | 6.196725061 | 6.356672044 | 6.261154673 | 6.250772132 | 6.329123596 | 0.011871907 | -3.115459386 |
| hsa-miR-383 | 7.145677455 | 8.554972496 | 8.368506462 | 7.089582893 | 9.570614722 | 9.101450482 | 9.726899318 | 8.933690655 | 9.63499251 | 8.168922782 | 0.026484955 | 1.399743585 |
| hsa-miR-384 | 6.986410935 | 6.715618859 | 6.694880193 | 7.031770114 | 7.075746538 | 6.609548139 | 7.599168993 | 7.388878339 | 7.408117408 | 7.39145842 | 0.05248052 | 0.388316281 |
| hsa-miR-409-3p | 8.548436625 | 10.10407452 | 9.857825524 | 6.207502459 | 6.530601415 | 10.68088692 | 6.721782768 | 6.668175869 | 7.635173947 | 6.45779126 | 0.31112222 | -1.230391085 |
| hsa-miR-409-5p | 5.968090752 | 8.87036472 | 8.613973587 | 5.961160258 | 6.274261661 | 6.755555262 | 5.988684687 | 6.422905743 | 6.10433666 | 6.45779126 | 0.294815914 | -1.019474784 |
| hsa-miR-410 | 6.224001674 | 8.848935856 | 7.85237345 | 8.211644987 | 6.399171094 | 6.465974465 | 6.721782768 | 6.668175869 | 6.411087227 | 6.569855608 | 0.111895192 | -1.24489782 |
| hsa-miR-411 | 9.332707934 | 11.39247843 | 10.62561753 | 9.90523656 | 6.530601415 | 6.465974465 | 6.356672044 | 7.388878339 | 6.411087227 | 6.100136671 | 0.001445441 | -3.771785086 |
| hsa-miR-411* | 6.314696526 | 7.338067798 | 7.617651119 | 7.14363831 | 6.155830172 | 6.465974465 | 6.721782768 | 6.668175869 | 6.83541884 | 6.001126665 | 0.106906865 | -0.628795308 |
| hsa-miR-412 | 7.427103287 | 7.40599236 | 7.432124273 | 7.542644987 | 7.273795599 | 8.25502857 | 6.930145948 | 7.23935985 | 7.117902789 | 7.141596278 | 0.545528579 | -0.125661387 |
| hsa-miR-421 | 10.16440416 | 10.93199324 | 11.30691611 | 10.67410397 | 9.14822234 | 8.894817763 | 7.78496195 | 7.857047916 | 8.597307691 | 8.063395081 | 0.000148441 | -2.37839558 |
| hsa-miR-422a | 6.001126665 | 5.944858446 | 5.930737338 | 6.171927354 | 6.05202456 | 5.992088609 | 5.927185358 | 5.820178962 | 6.10433666 | 6.100136671 | 0.862901088 | -0.012837314 |
| hsa-miR-423-3p | 11.67330908 | 12.42385126 | 12.50061784 | 12.45213826 | 7.484218708 | 11.99848466 | 6.930145948 | 12.36194377 | 7.117902789 | 8.405566974 | 0.023988505 | -3.212768633 |
| hsa-miR-423-5p | 14.37188552 | 11.72970571 | 12.19614103 | 12.78426676 | 14.37188552 | 14.07082534 | 14.11566957 | 14.18441048 | 14.27431991 | 14.02323456 | 0.092187418 | 1.402891142 |
| hsa-miR-424 | 10.34751064 | 13.35328469 | 13.07437565 | 12.40167953 | 7.484218708 | 7.316960287 | 7.145677455 | 6.953032377 | 7.635173947 | 7.925109324 | 0.004430558 | -4.884183944 |
| hsa-miR-424* | 9.435878577 | 9.760553633 | 9.730979533 | 8.003939486 | 6.399171094 | 6.316507819 | 6.356672044 | 6.422905743 | 6.584962501 | 6.45779126 | 0.006368793 | -2.809836064 |
| hsa-miR-425 | 11.02873489 | 12.26617162 | 12.42385126 | 11.75768169 | 6.274261661 | 13.50039377 | 6.356672044 | 6.953032377 | 6.584962501 | 13.41629614 | 0.094451116 | -3.021506783 |
| hsa-miR-425* | 9.156335937 | 10.37123213 | 11.12023788 | 9.451211112 | 8.151523994 | 7.501040996 | 11.89958351 | 9.192539408 | 8.11997861 | 8.498849207 | 0.186872029 | -1.130834977 |
| hsa-miR-429 | 8.356672044 | 10.06123617 | 9.650154214 | 6.338959175 | 11.68877483 | 8.15810472 | 13.10212335 | 7.23935985 | 5.888743249 | 13.03414554 | 0.438938196 | 1.250119857 |
| hsa-miR-431 | 10.61369739 | 8.889199726 | 7.170926525 | 10.22363975 | 6.399171094 | 7.117383367 | 6.930145948 | 13.73190236 | 6.83541884 | 7.006746832 | 0.405235529 | -1.220904442 |
| hsa-miR-431* | 7.974988112 | 6.263973355 | 6.343407822 | 6.421223299 | 6.862947248 | 7.316960287 | 6.356672044 | 7.515305605 | 6.584962501 | 6.714245518 | 0.767848324 | 0.14095072 |
| hsa-miR-432 | 10.44935502 | 12.16091137 | 11.86546264 | 11.72970571 | 8.893756568 | 13.3303147 | 13.34410169 | 8.25502857 | 9.207990433 | 13.73190236 | 0.718002541 | -0.4241763 |
| hsa-miR-432* | 6.340740281 | 6.112700133 | 6.085339669 | 6.292781749 | 6.05202456 | 6.316507819 | 6.207502459 | 6.0725346 | 6.250772132 | 6.100136671 | 0.613818281 | -0.041310751 |
| hsa-miR-433 | 8.271463028 | 10.69156906 | 10.12399203 | 9.796364138 | 5.980710829 | 9.393819448 | 5.927185358 | 5.902073579 | 5.888743249 | 5.842978832 | 0.003329673 | -3.231595182 |
| hsa-miR-448 | 5.988684687 | 6.448735798 | 6.350497247 | 5.94016675 | 6.155830172 | 6.316507819 | 6.207502459 | 5.988684687 | 5.888743249 | 6.329123596 | 0.824605339 | -0.034289124 |
| hsa-miR-449a | 6.60659028 | 6.236492618 | 7.783980414 | 6.374170076 | 7.484218708 | 7.117383367 | 7.400025518 | 7.753885283 | 8.453682186 | 7.006746832 | 0.113108067 | 0.785681969 |
| hsa-miR-449b | 8.937520951 | 8.959132577 | 8.797337288 | 9.101450482 | 11.22427306 | 10.71020375 | 11.30138187 | 10.36654079 | 11.33220442 | 10.78598367 | 1.54E-05 | 2.004570937 |
| hsa-miR-450a | 8.534302882 | 9.332707934 | 9.393819448 | 8.937520951 | 9.14822234 | 8.894817763 | 8.002252452 | 9.403012024 | 8.453682186 | 9.477859434 | 0.632922667 | -0.152946771 |
| hsa-miR-450b-3p | 6.112700133 | 5.927185358 | 6.89056829 | 6.053111336 | 7.075746538 | 6.951867504 | 7.400025518 | 7.596935142 | 7.635173947 | 6.45779126 | 0.013981199 | 0.940365372 |
| hsa-miR-450b-5p | 7.726558779 | 8.413204789 | 7.836050355 | 7.78496195 | 7.075746538 | 9.226773423 | 8.986979709 | 9.339181934 | 9.207990433 | 8.986979709 | 0.060806235 | 0.863747989 |
| hsa-miR-451 | 14.09416843 | 14.09416843 | 14.04598144 | 12.94553528 | 12.55837288 | 10.1096348 | 11.32187174 | 12.62956153 | 11.50858711 | 12.82348666 | 0.005130482 | -1.969710941 |
| hsa-miR-452 | 9.692266772 | 9.156335937 | 9.533719071 | 7.927777962 | 6.05202456 | 6.316507819 | 5.927185358 | 5.988684687 | 6.250772132 | 6.329123596 | 0.004404288 | -2.933475243 |
| hsa-miR-452* | 7.351380981 | 8.30833903 | 7.759222426 | 8.692789835 | 8.800252806 | 11.08261478 | 7.78496195 | 7.23935985 | 7.864805194 | 10.21188829 | 0.28131072 | 0.802714078 |
| hsa-miR-452*:9.1 | 7.371558863 | 8.603626345 | 8.160879862 | 8.026246773 | 6.530601415 | 5.992088609 | 6.523561956 | 5.988684687 | 6.250772132 | 5.92243574 | 0.002308564 | -1.839220537 |
| hsa-miR-453 | 6.836050355 | 7.572131751 | 8.156083076 | 7.343407822 | 8.381110294 | 8.437544026 | 8.002252452 | 7.515305605 | 7.635173947 | 9.688862232 | 0.095040909 | 0.799789841 |
| hsa-miR-454 | 7.850499414 | 8.680535344 | 8.122051448 | 7.572131751 | 7.799605422 | 8.15810472 | 8.002252452 | 7.596935142 | 8.453682186 | 9.52277767 | 0.601926346 | 0.199255109 |
| hsa-miR-454* | 8.800252806 | 8.214804795 | 9.082149041 | 7.293701542 | 6.399171094 | 6.196725061 | 5.988684687 | 12.32673844 | 6.411087227 | 10.99573182 | 0.817224102 | -0.29470399 |
| hsa-miR-455-3p | 11.15434346 | 12.28004576 | 12.06062839 | 12.33776126 | 8.607700188 | 8.040837649 | 8.002252452 | 11.33220442 | 7.117902789 | 8.498849207 | 0.00135115 | -3.358236932 |
| hsa-miR-455-5p | 9.981995318 | 9.58129475 | 9.044667331 | 9.846430632 | 13.05706098 | 8.25502857 | 8.514911265 | 11.06709819 | 9.207990433 | 9.116863758 | 0.754690121 | 0.256228524 |
| hsa-miR-483-3p | 10.57601187 | 9.191059215 | 9.09037711 | 10.65391987 | 9.049167872 | 8.60936345 | 8.986979709 | 8.522581531 | 9.456765088 | 9.353587949 | 0.127585387 | -0.881434418 |
| hsa-miR-483-5p | 7.032321287 | 7.146186791 | 7.141596278 | 7.993221467 | 7.484218708 | 6.755555262 | 7.400025518 | 10.94005661 | 7.408117408 | 7.625708843 | 0.386754403 | 0.607282268 |
| hsa-miR-484 | 12.14063808 | 12.62956153 | 12.78426676 | 12.19614103 | 9.049167872 | 12.89026428 | 9.77016832 | 12.28004576 | 8.597307691 | 13.3303147 | 0.150738588 | -1.451440416 |
| hsa-miR-485-3p | 7.898752526 | 8.972692654 | 8.897542973 | 8.453682186 | 7.273795599 | 7.921245889 | 7.78496195 | 7.388878339 | 7.864805194 | 8.647638302 | 0.054517039 | -0.742113372 |
| hsa-miR-485-5p | 8.318768743 | 8.461479447 | 8.088523257 | 8.897542973 | 8.381110294 | 7.625708843 | 8.413204789 | 8.452035274 | 8.300123725 | 7.925109324 | 0.277053945 | -0.258696563 |
| hsa-miR-486-3p | 11.93475974 | 9.818582177 | 10.54515755 | 10.76088624 | 8.381110294 | 7.316960287 | 7.400025518 | 7.388878339 | 7.635173947 | 8.063395081 | 0.002956049 | -3.067255851 |
| hsa-miR-486-5p | 13.87028765 | 13.73190236 | 14.02323456 | 13.5347649 | 13.07437565 | 13.61622719 | 12.28004576 | 13.5187515 | 13.80583454 | 14.45914981 | 0.335526697 | -0.330983291 |
| hsa-miR-487a | 8.255500733 | 7.936637939 | 6.268658955 | 6.804776378 | 7.799605422 | 6.755555262 | 7.145677455 | 11.95728346 | 8.11997861 | 7.006746832 | 0.403755105 | 0.814414338 |
| hsa-miR-487b | 8.613973587 | 11.33770552 | 11.05372229 | 9.67047922 | 7.273795599 | 6.755555262 | 6.930145948 | 7.1017131 | 6.83541884 | 7.625708843 | 0.01449164 | -3.081913888 |
| hsa-miR-488 | 6.439623138 | 6.251719093 | 6.404290064 | 6.64096791 | 7.075746538 | 6.755555262 | 6.523561956 | 6.543805176 | 8.11997861 | 7.141596278 | 0.059550089 | 0.592557252 |
| hsa-miR-488* | 9.014299495 | 6.854868383 | 6.87036472 | 7.421223299 | 11.16804527 | 9.443565213 | 7.599168993 | 11.39247843 | 7.408117408 | 7.925109324 | 0.108164239 | 1.615891798 |
| hsa-miR-489 | 5.968090752 | 6.133399125 | 5.965784285 | 5.910492832 | 6.671010241 | 6.465974465 | 6.523561956 | 6.953032377 | 6.411087227 | 5.92243574 | 0.014324711 | 0.496741919 |
| hsa-miR-490-3p | 7.119459934 | 6.789859641 | 6.851749041 | 7.094869433 | 8.151523994 | 7.75087444 | 7.599168993 | 6.953032377 | 6.83541884 | 7.141596278 | 0.094519902 | 0.441284641 |
| hsa-miR-490-5p | 6.653919873 | 6.429615964 | 6.417852515 | 7.635173947 | 8.893756568 | 7.75087444 | 8.730809755 | 7.927777962 | 8.597307691 | 7.270996061 | 0.008344973 | 1.411113171 |
| hsa-miR-491-3p | 7.450798854 | 8.336283388 | 7.621319301 | 7.900866808 | 8.381110294 | 8.344073938 | 8.167418146 | 7.753885283 | 8.453682186 | 8.647638302 | 0.095187518 | 0.46398427 |
| hsa-miR-491-5p | 9.570614722 | 10.09354961 | 9.186361891 | 11.1028287 | 10.78598367 | 10.24257869 | 10.84889674 | 10.38046107 | 10.9918761 | 10.24257869 | 0.252168397 | 0.593723763 |
| hsa-miR-492 | 6.896029603 | 6.154818109 | 8.725877459 | 5.970393538 | 7.075746538 | 7.117383367 | 8.002252452 | 7.23935985 | 8.300123725 | 6.569855608 | 0.546979996 | 0.447340579 |
| hsa-miR-493 | 12.94553528 | 9.981995318 | 10.79075575 | 10.57601187 | 7.273795599 | 10.16440416 | 12.19614103 | 13.68986697 | 7.408117408 | 6.857980995 | 0.306516571 | -1.475190196 |
| hsa-miR-493* | 8.854556752 | 8.797337288 | 8.595070954 | 8.167418146 | 8.68580002 | 8.344073938 | 8.413204789 | 10.01066796 | 8.453682186 | 8.543805176 | 0.659392549 | 0.13827656 |
| hsa-miR-494 | 11.12023788 | 8.937520951 | 7.75087444 | 8.23457796 | 9.248164779 | 8.819061399 | 9.160249616 | 9.182890893 | 9.328338334 | 9.958697703 | 0.742193876 | 0.272097647 |
| hsa-miR-495 | 7.236014192 | 8.838573653 | 7.964052011 | 8.084808388 | 8.151523994 | 11.39247843 | 7.78496195 | 8.106955457 | 8.725877459 | 8.543805176 | 0.268333311 | 0.753405017 |
| hsa-miR-496 | 6.570614722 | 6.410239331 | 6.268658955 | 6.465974465 | 6.862947248 | 6.609548139 | 7.145677455 | 7.388878339 | 7.635173947 | 7.141596278 | 0.003896494 | 0.701765033 |
| hsa-miR-497 | 11.05372229 | 11.80779765 | 11.01164658 | 12.16091137 | 6.274261661 | 7.117383367 | 6.356672044 | 6.422905743 | 6.250772132 | 6.569855608 | 5.23E-05 | -5.009877712 |
| hsa-miR-497* | 5.898450233 | 5.851749041 | 5.944858446 | 5.987548259 | 5.716990894 | 5.716990894 | 5.927185358 | 5.581953751 | 5.716990894 | 6.001126665 | 0.080376325 | -0.143778419 |
| hsa-miR-498 | 6.470862199 | 7.992088609 | 8.214804795 | 8.725877459 | 7.273795599 | 6.755555262 | 6.721782768 | 7.515305605 | 12.36194377 | 7.270996061 | 0.899206046 | 0.132321579 |
| hsa-miR-499-3p | 6.746178381 | 6.82527683 | 6.417852515 | 9.818582177 | 13.75690028 | 6.465974465 | 7.400025518 | 7.515305605 | 7.864805194 | 6.569855608 | 0.571843089 | 0.810171969 |
| hsa-miR-499-5p | 10.04384754 | 10.79075575 | 10.82033854 | 10.51914479 | 11.36779694 | 10.92577695 | 11.71334355 | 10.99573182 | 11.39247843 | 10.84172158 | 0.02523279 | 0.662619891 |
| hsa-miR-500 | 10.41309898 | 11.50858711 | 10.98463207 | 10.99957727 | 7.641690523 | 7.316960287 | 7.400025518 | 7.515305605 | 7.635173947 | 7.799605422 | 0.000236431 | -3.425013643 |
| hsa-miR-501-3p | 7.540709263 | 9.186361891 | 9.271929844 | 8.498849207 | 5.905687849 | 5.856736756 | 6.207502459 | 5.861707287 | 5.9795681 | 6.001126665 | 0.00642078 | -2.655741032 |
| hsa-miR-501-5p | 7.098032083 | 8.113221243 | 8.68580002 | 5.987548259 | 7.273795599 | 7.316960287 | 8.002252452 | 7.515305605 | 8.11997861 | 11.54099979 | 0.382917733 | 0.823731655 |
| hsa-miR-502-3p,hsa-miR-500* | 10.31955923 | 11.01663421 | 11.49355527 | 11.28320413 | 6.862947248 | 6.951867504 | 6.930145948 | 6.261154673 | 6.83541884 | 7.141596278 | 6.49E-05 | -4.197716462 |
| hsa-miR-502-5p | 6.89056829 | 7.74348804 | 7.635173947 | 8.854556752 | 6.399171094 | 6.951867504 | 6.356672044 | 6.168922782 | 6.250772132 | 7.502235115 | 0.053316859 | -1.176006646 |
| hsa-miR-503 | 10.40290547 | 12.69522829 | 12.53225851 | 11.86546264 | 6.862947248 | 6.755555262 | 7.400025518 | 6.790511412 | 7.408117408 | 7.625708843 | 0.001650914 | -4.733486113 |
| hsa-miR-504 | 14.01553701 | 11.12023788 | 11.79445648 | 13.34410169 | 14.02323456 | 13.87028765 | 14.27431991 | 14.41700199 | 14.23044081 | 13.60579201 | 0.109159726 | 1.501596222 |
| hsa-miR-505 | 9.97053734 | 9.90523656 | 9.760553633 | 9.318768743 | 6.399171094 | 6.092757141 | 5.988684687 | 6.668175869 | 6.10433666 | 6.194756854 | 1.61E-06 | -3.497460351 |
| hsa-miR-505* | 8.073606041 | 8.002252452 | 7.887525271 | 9.570614722 | 6.05202456 | 5.856736756 | 5.87282876 | 5.942514505 | 5.9795681 | 5.842978832 | 0.008248175 | -2.459057702 |
| hsa-miR-506 | 7.266786541 | 8.259507895 | 8.356672044 | 8.908842924 | 7.982993575 | 7.75087444 | 6.930145948 | 7.1017131 | 6.83541884 | 9.044667331 | 0.259891566 | -0.590316812 |
| hsa-miR-507 | 8.775116404 | 8.595070954 | 9.276589716 | 8.843292973 | 11.1028287 | 10.48290877 | 11.33220442 | 10.65391987 | 11.25844826 | 10.40290547 | 1.90E-05 | 1.99968507 |
| hsa-miR-508-3p | 6.163901214 | 7.09117089 | 7.572889668 | 6.912290583 | 5.905687849 | 6.316507819 | 8.289557848 | 5.861707287 | 5.804776378 | 6.194756854 | 0.298795137 | -0.539564083 |
| hsa-miR-508-5p | 6.396604781 | 6.726558779 | 6.578938713 | 6.696272084 | 6.671010241 | 6.609548139 | 6.930145948 | 7.515305605 | 7.117902789 | 7.925109324 | 0.052853463 | 0.528576752 |
| hsa-miR-509-3-5p | 6.001126665 | 6 | 6.05202456 | 6.411087227 | 6.399171094 | 6.196725061 | 6.356672044 | 6.790511412 | 6.584962501 | 6.714245518 | 0.022919034 | 0.390988325 |
| hsa-miR-509-3p | 9.336060181 | 6.623515741 | 8.003096215 | 7.66035251 | 13.34410169 | 6.609548139 | 6.930145948 | 11.26982798 | 13.40900634 | 6.714245518 | 0.262156449 | 1.80705644 |
| hsa-miR-509-5p | 7.017365205 | 7.609548139 | 6.161887682 | 6.207502459 | 6.862947248 | 6.951867504 | 7.145677455 | 6.543805176 | 7.635173947 | 6.100136671 | 0.77253566 | 0.124192129 |
| hsa-miR-510 | 6.545350645 | 6.498250868 | 6.613236955 | 6.887525271 | 6.862947248 | 6.755555262 | 7.400025518 | 6.790511412 | 7.117902789 | 6.329123596 | 0.201327563 | 0.239920036 |
| hsa-miR-511 | 10.33918193 | 10.4871366 | 10.87198222 | 11.12023788 | 6.530601415 | 7.316960287 | 6.930145948 | 7.515305605 | 7.117902789 | 6.857980995 | 1.85E-06 | -3.659818484 |
| hsa-miR-512-3p | 9.182890893 | 7.141596278 | 7.708049252 | 7.415319248 | 10.17130192 | 9.676662335 | 10.39687232 | 9.783898589 | 10.42500604 | 8.910492832 | 0.012559851 | 2.032075088 |
| hsa-miR-512-5p | 10.93199324 | 6.504620392 | 7.323279988 | 6.696272084 | 14.01553701 | 12.96155822 | 13.3303147 | 13.61622719 | 13.07437565 | 13.15864124 | 0.012046585 | 5.495400909 |
| hsa-miR-513:9.1 | 6.325530332 | 5.815063017 | 5.977279923 | 6.029011087 | 7.799605422 | 7.501040996 | 8.167418146 | 7.388878339 | 8.11997861 | 6.569855608 | 0.000705262 | 1.55440843 |
| hsa-miR-513a-3p | 6.251719093 | 6.393175914 | 6.502235115 | 6.512542955 | 6.862947248 | 6.755555262 | 6.721782768 | 6.543805176 | 6.584962501 | 6.45779126 | 0.025743347 | 0.239555767 |
| hsa-miR-513a-5p | 8.892694591 | 8.947783026 | 8.700786478 | 8.933690655 | 11.3810562 | 10.72766524 | 11.51491126 | 10.64925618 | 11.44403133 | 10.50143915 | 3.58E-05 | 2.167654538 |
| hsa-miR-513b | 6.62935662 | 6.404290064 | 6.292781749 | 6.653203362 | 6.671010241 | 6.465974465 | 6.089582893 | 6.543805176 | 6.83541884 | 7.502235115 | 0.400526447 | 0.189763173 |
| hsa-miR-513c | 6.275193334 | 6.601399391 | 6.663913842 | 6.787902559 | 7.484218708 | 7.316960287 | 7.400025518 | 7.23935985 | 7.864805194 | 7.712870868 | 0.000408734 | 0.92093779 |
| hsa-miR-514 | 6.483815777 | 5.786596362 | 5.842978832 | 5.820178962 | 6.05202456 | 5.992088609 | 5.988684687 | 6.422905743 | 6.10433666 | 5.842978832 | 0.672520486 | 0.083777365 |
| hsa-miR-515-3p | 6.874059203 | 7.1017131 | 6.964629667 | 7.410239331 | 10.81382127 | 10.37123213 | 11.03981011 | 9.62580015 | 11.01164658 | 9.760553633 | 8.08E-06 | 3.349483654 |
| hsa-miR-515-5p | 6.053111336 | 6.176921112 | 6.187846909 | 7.45532722 | 11.55588324 | 6.465974465 | 11.99848466 | 6.332707934 | 6.584962501 | 6.329123596 | 0.190683146 | 1.742887755 |
| hsa-miR-516a-3p,hsa-miR-516b* | 8.368506462 | 8.185866545 | 7.985272714 | 8.255500733 | 9.920427296 | 9.315715658 | 10.43426334 | 10.01066796 | 9.958697703 | 9.655262373 | 2.05E-05 | 1.683719108 |
| hsa-miR-516a-5p | 9.00365845 | 9.207990433 | 8.986979709 | 9.276589716 | 10.53974043 | 10.06743436 | 10.78598367 | 10.30138187 | 10.67410397 | 10.04651016 | 3.53E-05 | 1.283721167 |
| hsa-miR-516b | 6.815063017 | 6.339850003 | 7.263034406 | 6.365447589 | 8.151523994 | 8.25502857 | 8.514911265 | 7.753885283 | 8.453682186 | 6.194756854 | 0.022352411 | 1.191449272 |
| hsa-miR-517* | 6.292781749 | 6.849248703 | 6.154818109 | 6.224966365 | 6.155830172 | 6.465974465 | 6.721782768 | 6.953032377 | 6.250772132 | 7.006746832 | 0.358311126 | 0.211902726 |
| hsa-miR-517a | 10.39081383 | 10.027906 | 10.21188829 | 10.37585398 | 12.179878 | 11.23595438 | 12.46867495 | 11.44403133 | 12.58282411 | 11.55588324 | 0.000465699 | 1.659592142 |
| hsa-miR-517a,hsa-miR-517b | 11.42621233 | 7.266786541 | 9.053654418 | 10.41309898 | 13.90966811 | 12.90809234 | 13.5187515 | 13.74706006 | 13.59243368 | 11.75768169 | 0.019885607 | 3.699009827 |
| hsa-miR-517c | 7.916775185 | 8.339850003 | 8.063395081 | 8.92659251 | 10.38046107 | 10.31118066 | 10.35110487 | 8.977852308 | 10.07961811 | 8.910492832 | 0.002962247 | 1.523465114 |
| hsa-miR-518a-3p | 11.64029013 | 11.44403133 | 11.18090371 | 11.25844826 | 12.01300855 | 11.42621233 | 12.25017997 | 12.08470875 | 12.28004576 | 11.44403133 | 0.022106915 | 0.535446092 |
| hsa-miR-518a-5p,hsa-miR-527 | 6.417852515 | 6.154818109 | 6.23457796 | 6.447909749 | 8.68580002 | 8.521207816 | 8.514911265 | 7.753885283 | 8.838573653 | 6.714245518 | 0.002027067 | 1.857647676 |
| hsa-miR-518b | 11.86546264 | 7.276124405 | 7.031770114 | 8.71630504 | 12.42385126 | 11.75768169 | 12.90809234 | 13.26034622 | 12.6077233 | 10.8560364 | 0.042650095 | 3.579872984 |
| hsa-miR-518c | 6.411087227 | 6.0725346 | 6.175923742 | 6.155830172 | 6.274261661 | 6.092757141 | 5.988684687 | 5.902073579 | 5.9795681 | 6.45779126 | 0.457674359 | -0.087987864 |
| hsa-miR-518c* | 7.890264277 | 7.992088609 | 7.867896464 | 8.382840144 | 11.49355527 | 10.8560364 | 12.16091137 | 10.44159552 | 11.7417619 | 10.80727441 | 1.47E-05 | 3.216916772 |
| hsa-miR-518d-3p | 8.741635516 | 8.747521673 | 8.6387979 | 8.599540966 | 11.01164658 | 10.41811614 | 11.16804527 | 10.35110487 | 11.12023788 | 10.36654079 | 3.23E-05 | 2.057407908 |
| hsa-miR-518e | 6.731997787 | 6.653919873 | 6.709428501 | 7.062855655 | 9.792139552 | 8.15810472 | 8.986979709 | 8.647638302 | 8.453682186 | 7.39145842 | 0.002243085 | 1.782116694 |
| hsa-miR-518e*,hsa-miR-519a*,hsa-miR-519b-5p,hsa-miR-519c-5p,hsa-miR-522*,hsa-miR-523* | 8.493054842 | 6.726558779 | 8.523953939 | 6.619853147 | 11.97799537 | 6.755555262 | 7.145677455 | 7.1017131 | 8.300123725 | 7.006746832 | 0.651396998 | 0.457113447 |
| hsa-miR-518e:9.1 | 7.600656308 | 7.338067798 | 7.370251481 | 7.378511623 | 7.799605422 | 7.625708843 | 7.78496195 | 7.388878339 | 7.117902789 | 7.270996061 | 0.575725526 | 0.076137098 |
| hsa-miR-518f | 6.559950613 | 7.111657346 | 7.87282876 | 6.255500733 | 6.274261661 | 6.092757141 | 6.089582893 | 13.30457949 | 6.411087227 | 6.194756854 | 0.731539912 | 0.444519848 |
| hsa-miR-518f*,hsa-miR-518d-5p,hsa-miR-520c-5p,hsa-miR-526a | 6.352264173 | 6.277984747 | 6.227856573 | 6.54225805 | 6.671010241 | 6.316507819 | 6.830990481 | 7.23935985 | 7.117902789 | 6.714245518 | 0.018015845 | 0.464911897 |
| hsa-miR-518f:9.1 | 6.506208389 | 6.199672345 | 7.653203362 | 6.255500733 | 5.837943242 | 5.856736756 | 7.145677455 | 6.0725346 | 6.250772132 | 5.92243574 | 0.284724455 | -0.472629553 |
| hsa-miR-519a | 6.599912842 | 7.056366761 | 7.146186791 | 7.614709844 | 7.484218708 | 6.755555262 | 7.145677455 | 6.953032377 | 7.408117408 | 6.569855608 | 0.846569212 | -0.051551257 |
| hsa-miR-519b-3p | 9.299665944 | 9.403012024 | 9.299665944 | 9.832731815 | 11.39247843 | 10.84172158 | 11.6092711 | 10.56985561 | 11.54846071 | 10.74306722 | 8.03E-05 | 1.658706842 |
| hsa-miR-519c-3p | 6.723831566 | 7.056366761 | 7.30833903 | 7.675957033 | 7.982993575 | 7.75087444 | 8.413204789 | 8.106955457 | 8.597307691 | 7.39145842 | 0.016050779 | 0.849342131 |
| hsa-miR-519d | 6.782670659 | 5.944858446 | 7.084276911 | 6.276124405 | 8.518849829 | 7.501040996 | 7.599168993 | 7.927777962 | 8.597307691 | 7.006746832 | 0.006553637 | 1.336499445 |
| hsa-miR-519e | 8.033973543 | 7.730639956 | 7.315602457 | 7.920055055 | 7.641690523 | 8.819061399 | 10.06433859 | 9.91064273 | 9.456765088 | 9.616089314 | 0.007847722 | 1.501363522 |
| hsa-miR-519e* | 6.545350645 | 6.545350645 | 6.154818109 | 6.325530332 | 6.399171094 | 6.196725061 | 6.089582893 | 6.332707934 | 6.250772132 | 6.194756854 | 0.223055713 | -0.148809771 |
| hsa-miR-520a-3p | 8.194264383 | 8.021812824 | 8.324180547 | 7.77280941 | 7.982993575 | 7.921245889 | 8.167418146 | 8.60936345 | 8.300123725 | 7.799605422 | 0.767745013 | 0.051858244 |
| hsa-miR-520a-5p | 6.301953395 | 6.417852515 | 6.45779126 | 6.578938713 | 8.381110294 | 7.921245889 | 8.002252452 | 7.515305605 | 8.11997861 | 7.625708843 | 2.16E-05 | 1.488466311 |
| hsa-miR-520b,hsa-miR-520c-3p,hsa-miR-520f | 6.707359132 | 6.60659028 | 6.709428501 | 7.563768278 | 9.570614722 | 8.25502857 | 8.889199726 | 8.933690655 | 8.597307691 | 8.168922782 | 0.000478998 | 1.839007476 |
| hsa-miR-520c-3p,hsa-miR-520f | 6.570614722 | 6.422905743 | 6.428778891 | 6.74281467 | 10.65391987 | 9.177419538 | 9.40779885 | 9.182890893 | 10.31118066 | 8.75788999 | 9.68E-05 | 3.040571461 |
| hsa-miR-520d:9.1 | 6.452858965 | 6.82527683 | 6.536830005 | 6.465974465 | 7.075746538 | 6.316507819 | 6.721782768 | 6.422905743 | 7.117902789 | 6.714245518 | 0.351584844 | 0.157946796 |
| hsa-miR-520d-3p | 6.422905743 | 6.504620392 | 6.709428501 | 6.399171094 | 5.980710829 | 5.992088609 | 6.207502459 | 5.902073579 | 6.10433666 | 6.001126665 | 0.001832426 | -0.477724966 |
| hsa-miR-520d-5p | 6.980710829 | 6.504620392 | 6.54225805 | 7.038918989 | 7.484218708 | 7.501040996 | 9.507001733 | 9.435878577 | 9.098821663 | 7.39145842 | 0.010665575 | 1.636442951 |
| hsa-miR-520e | 6.155830172 | 5.927185358 | 7.257859235 | 6.937815169 | 10.98113912 | 6.316507819 | 6.356672044 | 12.82348666 | 13.03414554 | 10.4871366 | 0.037544552 | 3.430175482 |
| hsa-miR-520f | 6.201633861 | 6.393175914 | 6.394033895 | 6.601399391 | 6.862947248 | 7.117383367 | 6.356672044 | 6.790511412 | 6.250772132 | 6.45779126 | 0.170548954 | 0.241785478 |
| hsa-miR-520g | 5.944858446 | 5.911691582 | 5.902073579 | 6.207502459 | 5.62935662 | 5.911691582 | 5.786596362 | 5.752213368 | 5.888743249 | 5.92243574 | 0.091893011 | -0.176358696 |
| hsa-miR-520h,hsa-miR-520g | 7.495855027 | 7.949534933 | 9.258330473 | 6.338959175 | 6.399171094 | 6.755555262 | 7.145677455 | 6.790511412 | 6.584962501 | 6.714245518 | 0.186329747 | -1.028982695 |
| hsa-miR-521 | 7.890264277 | 5.988684687 | 6.05202456 | 6.196725061 | 6.399171094 | 6.092757141 | 6.207502459 | 6.168922782 | 6.10433666 | 7.270996061 | 0.763748116 | -0.157976947 |
| hsa-miR-522 | 7.106432078 | 7.284477118 | 7.017365205 | 7.487840034 | 7.484218708 | 7.316960287 | 12.45213826 | 7.857047916 | 12.14063808 | 7.799605422 | 0.106378885 | 1.951072837 |
| hsa-miR-523 | 6.476543706 | 6.773468928 | 6.613236955 | 7.062855655 | 7.799605422 | 7.921245889 | 8.002252452 | 11.64029013 | 7.864805194 | 8.063395081 | 0.031820676 | 1.817072717 |
| hsa-miR-524-3p | 7.594324604 | 7.188836073 | 7.55688919 | 7.216745858 | 7.641690523 | 13.67325386 | 7.599168993 | 8.15810472 | 9.328338334 | 10.72766524 | 0.077726934 | 2.132171346 |
| hsa-miR-524-5p | 6.636624621 | 6.350497247 | 6.451211112 | 6.787902559 | 6.671010241 | 6.951867504 | 7.145677455 | 6.953032377 | 7.117902789 | 7.141596278 | 0.010660296 | 0.44028889 |
| hsa-miR-525-3p | 7.594324604 | 8.168922782 | 8.047669251 | 8.339850003 | 7.075746538 | 7.75087444 | 6.721782768 | 6.953032377 | 7.117902789 | 7.712870868 | 0.008652227 | -0.815656697 |
| hsa-miR-525-5p | 9.107217076 | 8.440453944 | 8.664269492 | 8.612499946 | 11.05372229 | 10.61966977 | 11.68877483 | 11.15785217 | 11.49355527 | 10.28505538 | 1.97E-05 | 2.343661504 |
| hsa-miR-526b | 5.581953751 | 5.581953751 | 5.696272084 | 5.760220946 | 8.893756568 | 9.024308597 | 9.32395546 | 8.203837384 | 8.959132577 | 6.45779126 | 0.001178885 | 2.822030174 |
| hsa-miR-526b* | 5.988684687 | 5.927185358 | 5.930737338 | 5.94016675 | 6.155830172 | 6.316507819 | 6.207502459 | 6.168922782 | 6.10433666 | 6.329123596 | 0.000413159 | 0.267010381 |
| hsa-miR-526b:9.1 | 6.523561956 | 6.800252806 | 6.77478706 | 7.1048608 | 11.5740258 | 7.117383367 | 7.599168993 | 7.23935985 | 7.635173947 | 7.270996061 | 0.132412077 | 1.271819014 |
| hsa-miR-532-3p | 10.56367296 | 9.182890893 | 10.15000109 | 10.35469017 | 8.381110294 | 7.625708843 | 8.730809755 | 7.388878339 | 8.11997861 | 7.270996061 | 0.001212185 | -2.143233462 |
| hsa-miR-532-5p | 13.18780053 | 10.72766524 | 11.03981011 | 11.5740258 | 6.862947248 | 11.2062818 | 13.26034622 | 13.7905725 | 13.60579201 | 8.248876343 | 0.735026175 | -0.469856065 |
| hsa-miR-539 | 7.764871591 | 10.06743436 | 8.416164165 | 7.764871591 | 8.968378801 | 8.040837649 | 7.400025518 | 7.1017131 | 6.83541884 | 8.910492832 | 0.380105126 | -0.62719097 |
| hsa-miR-541 | 6.301953395 | 6.504620392 | 6.483009577 | 6.759555343 | 6.862947248 | 6.951867504 | 7.145677455 | 6.332707934 | 6.584962501 | 7.502235115 | 0.083958604 | 0.384448282 |
| hsa-miR-541* | 7.87036472 | 6.429615964 | 6.510961919 | 6.887525271 | 8.518849829 | 7.75087444 | 8.607700188 | 10.35110487 | 8.300123725 | 7.799605422 | 0.012790522 | 1.630092777 |
| hsa-miR-542-3p | 6.691394579 | 9.916028718 | 8.1017131 | 7.314243347 | 6.671010241 | 6.465974465 | 6.721782768 | 6.422905743 | 6.83541884 | 7.141596278 | 0.160076827 | -1.296063547 |
| hsa-miR-542-5p | 7.032321287 | 9.248164779 | 8.862017377 | 7.477353527 | 7.273795599 | 11.44403133 | 11.86546264 | 7.23935985 | 7.635173947 | 7.141596278 | 0.581252406 | 0.611605698 |
| hsa-miR-543 | 6.782670659 | 7.495855027 | 7.160879862 | 7.275193334 | 7.273795599 | 7.117383367 | 6.930145948 | 7.1017131 | 7.408117408 | 11.05372229 | 0.381368456 | 0.635496565 |
| hsa-miR-544 | 14.18441048 | 13.88718253 | 14.37188552 | 14.14972331 | 14.71007995 | 14.71007995 | 14.71007995 | 14.61467534 | 14.71007995 | 14.71007995 | 0.010920397 | 0.545878724 |
| hsa-miR-545 | 6.917670434 | 6.636624621 | 6.767522386 | 7.314243347 | 8.607700188 | 7.625708843 | 8.607700188 | 7.1017131 | 9.328338334 | 7.39145842 | 0.017884112 | 1.201421315 |
| hsa-miR-545* | 7.641690523 | 8.014578465 | 7.927777962 | 8.862017377 | 6.399171094 | 6.465974465 | 7.78496195 | 6.543805176 | 6.250772132 | 7.006746832 | 0.005913721 | -1.369610807 |
| hsa-miR-545:9.1 | 8.002252452 | 7.949534933 | 7.837312556 | 8.110091752 | 12.83547806 | 10.24257869 | 9.650154214 | 8.956666555 | 10.61966977 | 12.03675744 | 0.005682191 | 2.748752865 |
| hsa-miR-548a-3p | 9.487840034 | 9.223036338 | 9.192539408 | 9.58458675 | 11.35980446 | 10.89231512 | 11.72970571 | 11.08261478 | 11.63444806 | 11.28320413 | 2.09E-06 | 1.958348078 |
| hsa-miR-548a-5p | 6.112700133 | 6.301953395 | 6.36018867 | 6.465974465 | 6.05202456 | 6.316507819 | 6.207502459 | 6.422905743 | 6.250772132 | 6.714245518 | 0.888467023 | 0.017122206 |
| hsa-miR-548b-3p | 6.830990481 | 7.216745858 | 7.444600814 | 7.353146825 | 11.12023788 | 10.77107674 | 11.5740258 | 8.933690655 | 10.89231512 | 9.779883475 | 0.000208132 | 3.300500617 |
| hsa-miR-548b-5p | 9.70822173 | 9.929554316 | 9.796364138 | 9.958697703 | 11.29582632 | 10.74306722 | 11.49355527 | 10.67410397 | 11.33220442 | 11.02160465 | 9.28E-05 | 1.245184171 |
| hsa-miR-548c-3p | 7.220136447 | 7.276124405 | 7.111657346 | 7.495855027 | 8.518849829 | 8.437544026 | 8.986979709 | 9.014299495 | 13.19746245 | 8.063395081 | 0.043299324 | 2.093811791 |
| hsa-miR-548c-5p | 6.937815169 | 7.259272487 | 6.917670434 | 7.978424465 | 7.799605422 | 7.921245889 | 8.289557848 | 8.003096215 | 7.864805194 | 7.625708843 | 0.07413833 | 0.64404093 |
| hsa-miR-548d-3p | 7.536441505 | 7.24697806 | 7.384567923 | 6.976707312 | 7.075746538 | 6.755555262 | 6.930145948 | 6.953032377 | 7.117902789 | 6.857980995 | 0.057186611 | -0.337779715 |
| hsa-miR-548d-5p | 9.140318539 | 9.528063348 | 9.474719947 | 9.857825524 | 9.248164779 | 8.710462074 | 9.082149041 | 8.25502857 | 9.328338334 | 8.248876343 | 0.023312617 | -0.688061983 |
| hsa-miR-548e | 6.676662335 | 6.845490051 | 6.836681593 | 7.123604124 | 6.671010241 | 6.316507819 | 6.356672044 | 6.261154673 | 7.864805194 | 7.502235115 | 0.891875742 | -0.041878678 |
| hsa-miR-548f | 7.519243094 | 7.628627802 | 7.396604781 | 7.697315122 | 9.939285356 | 9.49645436 | 10.31118066 | 9.014299495 | 10.07961811 | 9.250416861 | 6.92E-05 | 2.121428108 |
| hsa-miR-548g | 6.874059203 | 6.483815777 | 6.957682486 | 6.559950613 | 6.862947248 | 7.117383367 | 7.400025518 | 6.422905743 | 6.83541884 | 9.315715658 | 0.214801154 | 0.606855709 |
| hsa-miR-548h | 6.261154673 | 6.083213368 | 6.132371199 | 6.171927354 | 6.530601415 | 6.755555262 | 7.145677455 | 6.953032377 | 6.584962501 | 6.569855608 | 0.001274216 | 0.594447455 |
| hsa-miR-548i | 6.874059203 | 7.452035274 | 7.286326727 | 7.712183051 | 7.799605422 | 7.625708843 | 8.289557848 | 7.753885283 | 8.11997861 | 7.141596278 | 0.097685635 | 0.45723765 |
| hsa-miR-548j | 7.259272487 | 6.510961919 | 7.111657346 | 6.776762002 | 7.799605422 | 8.710462074 | 8.289557848 | 7.857047916 | 7.864805194 | 9.276589716 | 0.001588994 | 1.38501459 |
| hsa-miR-548k | 6.130313146 | 6.209453366 | 7.362820526 | 6.619853147 | 7.075746538 | 6.755555262 | 7.400025518 | 6.953032377 | 6.83541884 | 6.857980995 | 0.256484471 | 0.399016542 |
| hsa-miR-548l | 6.860466259 | 6.961160258 | 6.755555262 | 7.227856573 | 8.151523994 | 7.625708843 | 8.167418146 | 8.326204751 | 8.11997861 | 12.83547806 | 0.061258059 | 1.919792479 |
| hsa-miR-548m | 8.318768743 | 8.514911265 | 8.316507819 | 8.562624039 | 7.484218708 | 9.981995318 | 7.145677455 | 10.1572206 | 9.098821663 | 7.625708843 | 0.789641927 | 0.154070798 |
| hsa-miR-548n | 5.857980995 | 5.894817763 | 5.977279923 | 5.87774425 | 6.274261661 | 6.092757141 | 6.207502459 | 6.0725346 | 5.9795681 | 6.100136671 | 0.00260543 | 0.219171039 |
| hsa-miR-548o | 6.201633861 | 6.876516947 | 6.112700133 | 6.224966365 | 7.484218708 | 7.316960287 | 8.002252452 | 7.753885283 | 7.635173947 | 7.141596278 | 0.00149105 | 1.201726833 |
| hsa-miR-548p | 6.590961241 | 7.230741003 | 7.078417702 | 7.449561375 | 7.982993575 | 7.921245889 | 7.145677455 | 7.857047916 | 7.864805194 | 7.502235115 | 0.032210138 | 0.624913861 |
| hsa-miR-549 | 6.881419908 | 7.368942914 | 6.972692654 | 7.526694846 | 8.518849829 | 8.15810472 | 8.607700188 | 8.486231678 | 8.11997861 | 9.353587949 | 0.000487933 | 1.353304582 |
| hsa-miR-550 | 9.186361891 | 9.140318539 | 8.843292973 | 10.16440416 | 7.273795599 | 6.196725061 | 7.400025518 | 8.486231678 | 7.117902789 | 7.141596278 | 0.00124047 | -2.064214903 |
| hsa-miR-550* | 9.192539408 | 8.812498225 | 10.00450139 | 10.64925618 | 14.07082534 | 9.101450482 | 14.09416843 | 13.36342298 | 13.98751273 | 13.88718253 | 0.006585377 | 3.419394949 |
| hsa-miR-551a | 9.893756568 | 9.318768743 | 9.412569847 | 10.30138187 | 13.60579201 | 13.80583454 | 12.1027959 | 13.12028648 | 12.179878 | 11.34318572 | 0.000252017 | 2.96134285 |
| hsa-miR-551b | 7.474111514 | 8.122051448 | 8.611024797 | 8.498849207 | 6.05202456 | 6.196725061 | 6.207502459 | 6.168922782 | 6.411087227 | 6.194756854 | 0.003796055 | -1.971339417 |
| hsa-miR-551b* | 7.277054876 | 6.122051448 | 8.236492618 | 5.675251386 | 6.862947248 | 6.465974465 | 6.089582893 | 6.168922782 | 6.10433666 | 7.270996061 | 0.615849643 | -0.333919231 |
| hsa-miR-552 | 6.396604781 | 6.133399125 | 6.628627802 | 6.292781749 | 10.17130192 | 7.501040996 | 8.002252452 | 8.522581531 | 7.117902789 | 7.925109324 | 0.007564594 | 1.843844804 |
| hsa-miR-553 | 6.139551352 | 6.451211112 | 6.36719631 | 6.300123725 | 6.274261661 | 5.992088609 | 6.356672044 | 6.261154673 | 6.411087227 | 8.063395081 | 0.466574939 | 0.245255924 |
| hsa-miR-554 | 8.808964175 | 8.633721813 | 8.532161085 | 8.147713722 | 8.607700188 | 8.950993232 | 9.726899318 | 12.3835693 | 9.783898589 | 11.03981011 | 0.042043506 | 1.551504925 |
| hsa-miR-555 | 6.433794059 | 6.43629512 | 6.417852515 | 6.54225805 | 6.399171094 | 6.465974465 | 6.930145948 | 6.790511412 | 6.411087227 | 6.329123596 | 0.389294207 | 0.096785688 |
| hsa-miR-556-3p | 5.876516947 | 7.155830172 | 6.224966365 | 5.923624611 | 5.980710829 | 5.856736756 | 6.089582893 | 5.942514505 | 5.804776378 | 5.842978832 | 0.296194158 | -0.375684491 |
| hsa-miR-556-5p | 5.857980995 | 6.483815777 | 6.027905997 | 5.94016675 | 6.155830172 | 6.196725061 | 6.356672044 | 6.261154673 | 6.584962501 | 6.194756854 | 0.23230181 | 0.214216171 |
| hsa-miR-557 | 9.276589716 | 6.205548911 | 7.136478518 | 8.450386479 | 6.862947248 | 9.852139328 | 10.11517373 | 12.46867495 | 6.411087227 | 8.689299161 | 0.28917729 | 1.299302702 |
| hsa-miR-558 | 5.911691582 | 6.263973355 | 6.105384749 | 6.136478518 | 6.862947248 | 6.755555262 | 11.66540254 | 6.668175869 | 7.408117408 | 12.1027959 | 0.065583422 | 2.472783653 |
| hsa-miR-559 | 6.680886921 | 7.24697806 | 8.713386515 | 9.09037711 | 6.862947248 | 7.625708843 | 13.76677734 | 6.543805176 | 7.635173947 | 8.405566974 | 0.674355572 | 0.54042277 |
| hsa-miR-560:9.1 | 10.21188829 | 10.1321141 | 9.927185358 | 9.967802645 | 8.151523994 | 7.501040996 | 7.145677455 | 7.753885283 | 7.864805194 | 8.168922782 | 5.76E-06 | -2.295438316 |
| hsa-miR-561 | 6.411087227 | 6.612499946 | 6.653203362 | 6.976707312 | 9.412569847 | 9.393819448 | 9.32395546 | 8.452035274 | 10.1365426 | 8.589838376 | 4.21E-05 | 2.554752373 |
| hsa-miR-562 | 5.923624611 | 5.867896464 | 5.944858446 | 6.053111336 | 6.399171094 | 6.465974465 | 7.145677455 | 6.422905743 | 7.117902789 | 6.329123596 | 0.005398652 | 0.699419809 |
| hsa-miR-563 | 7.30833903 | 7.318316841 | 7.160879862 | 7.424586226 | 9.412569847 | 9.353587949 | 9.082149041 | 8.003096215 | 8.597307691 | 9.726899318 | 0.000883351 | 1.726237854 |
| hsa-miR-564 | 8.933690655 | 8.725877459 | 8.520029304 | 8.670656249 | 9.570614722 | 9.644306962 | 9.57799522 | 9.192539408 | 9.574309691 | 9.160249616 | 0.000383469 | 0.74077252 |
| hsa-miR-565:9.1 | 13.30457949 | 11.7417619 | 12.75713995 | 10.94807516 | 6.862947248 | 6.316507819 | 6.930145948 | 6.422905743 | 6.83541884 | 6.714245518 | 0.00140202 | -5.507527272 |
| hsa-miR-566 | 9.258330473 | 7.487840034 | 6.74281467 | 7.05202456 | 7.273795599 | 6.609548139 | 6.930145948 | 6.953032377 | 7.117902789 | 6.857980995 | 0.315533533 | -0.678184793 |
| hsa-miR-567 | 7.002252452 | 7.071462363 | 7.041111535 | 7.371558863 | 8.607700188 | 8.437544026 | 9.160249616 | 8.647638302 | 8.959132577 | 8.168922782 | 2.40E-05 | 1.541934946 |
| hsa-miR-568 | 6.62935662 | 6.861707287 | 6.77478706 | 10.19204618 | 7.982993575 | 6.951867504 | 8.514911265 | 12.89026428 | 7.635173947 | 8.910492832 | 0.355139116 | 1.199809614 |
| hsa-miR-569 | 6.815063017 | 6.617651119 | 6.87774425 | 6.663913842 | 6.862947248 | 6.951867504 | 7.78496195 | 8.25502857 | 7.864805194 | 7.925109324 | 0.012287786 | 0.863860241 |
| hsa-miR-570 | 7.290940402 | 7.061776198 | 6.642412773 | 7.00337736 | 7.799605422 | 8.25502857 | 8.730809755 | 8.60936345 | 8.300123725 | 8.248876343 | 0.000155969 | 1.324341194 |
| hsa-miR-571 | 8.452035274 | 8.381110294 | 8.838573653 | 9.36128586 | 10.06743436 | 9.676662335 | 10.00070427 | 9.082149041 | 9.574309691 | 9.57799522 | 0.017149051 | 0.904957883 |
| hsa-miR-572 | 8.725877459 | 7.343407822 | 7.958262697 | 7.235535607 | 7.484218708 | 7.75087444 | 7.400025518 | 7.23935985 | 7.864805194 | 7.270996061 | 0.435633563 | -0.314057601 |
| hsa-miR-573 | 7.756889855 | 7.599168993 | 7.416164165 | 7.703211467 | 10.00070427 | 9.768101599 | 10.2564446 | 9.152284842 | 10.07961811 | 8.647638302 | 0.000295685 | 2.03194 |
| hsa-miR-574-3p | 13.43564422 | 12.46867495 | 12.58282411 | 13.15864124 | 6.155830172 | 13.12847772 | 6.523561956 | 13.24425976 | 7.117902789 | 11.47126876 | 0.061254231 | -3.304562604 |
| hsa-miR-574-5p | 10.35469017 | 10.59395128 | 10.74306722 | 11.39247843 | 9.32395546 | 10.93199324 | 8.607700188 | 8.522581531 | 12.06062839 | 8.961594391 | 0.147473395 | -1.036304576 |
| hsa-miR-575 | 5.974988112 | 6.429615964 | 5.902073579 | 6.078951341 | 6.155830172 | 6.092757141 | 6.207502459 | 6.0725346 | 5.9795681 | 6.001126665 | 0.929939054 | -0.011520726 |
| hsa-miR-576-3p | 6.334496768 | 6.980710829 | 6.885086225 | 8.328226119 | 10.47421294 | 9.715533064 | 10.71896091 | 8.858291887 | 10.67410397 | 9.184627436 | 0.001657108 | 2.805491716 |
| hsa-miR-576-5p | 10.71020375 | 12.06062839 | 12.1027959 | 12.12350713 | 7.075746538 | 7.625708843 | 7.145677455 | 7.1017131 | 8.11997861 | 7.270996061 | 0.000196404 | -4.35931369 |
| hsa-miR-577 | 6.121015401 | 6.100136671 | 6.268658955 | 6.365447589 | 6.05202456 | 6.092757141 | 6.207502459 | 6.261154673 | 6.250772132 | 6.329123596 | 0.85190103 | -0.01492556 |
| hsa-miR-578 | 7.141596278 | 7.518849829 | 8.173926932 | 7.721782768 | 7.484218708 | 7.501040996 | 6.523561956 | 6.422905743 | 7.117902789 | 7.39145842 | 0.093525755 | -0.565524183 |
| hsa-miR-579 | 6.28169825 | 5.988684687 | 6.105384749 | 6.255500733 | 7.641690523 | 6.316507819 | 6.930145948 | 6.261154673 | 6.83541884 | 8.063395081 | 0.033188506 | 0.850235043 |
| hsa-miR-580 | 6.860466259 | 7.398316163 | 7.180406485 | 7.535275377 | 7.982993575 | 7.117383367 | 8.002252452 | 7.661778098 | 7.117902789 | 7.625708843 | 0.157457536 | 0.341053783 |
| hsa-miR-581 | 7.032321287 | 7.845490051 | 7.312429206 | 7.708049252 | 9.049167872 | 9.676662335 | 7.78496195 | 11.50109077 | 7.864805194 | 8.405566974 | 0.039913641 | 1.572470067 |
| hsa-miR-582-3p | 6.288173968 | 6.659639187 | 6.881419908 | 6.951867504 | 6.530601415 | 7.117383367 | 6.523561956 | 6.790511412 | 6.10433666 | 8.910492832 | 0.512178018 | 0.300872798 |
| hsa-miR-582-5p | 6.89056829 | 8.700786478 | 9.14822234 | 7.192785959 | 7.484218708 | 7.316960287 | 7.599168993 | 7.23935985 | 7.864805194 | 7.006746832 | 0.387477522 | -0.564547456 |
| hsa-miR-583 | 7.861707287 | 8.344073938 | 8.221103725 | 8.324180547 | 10.50476934 | 9.852139328 | 9.57799522 | 8.897542973 | 10.07961811 | 9.802758679 | 0.000308619 | 1.598037568 |
| hsa-miR-584 | 11.99848466 | 9.364134655 | 10.08308037 | 9.620036498 | 13.38548496 | 12.6077233 | 12.26617162 | 13.92203613 | 13.28896698 | 13.28896698 | 0.011249681 | 2.860124285 |
| hsa-miR-585 | 8.286326727 | 8.670656249 | 8.892694591 | 8.797337288 | 8.151523994 | 8.894817763 | 8.607700188 | 11.849366 | 12.83547806 | 8.063395081 | 0.262541598 | 1.0719598 |
| hsa-miR-586 | 7.206526016 | 6.972692654 | 6.844234988 | 7.314243347 | 7.075746538 | 6.951867504 | 6.930145948 | 7.515305605 | 7.408117408 | 8.063395081 | 0.282767262 | 0.239672096 |
| hsa-miR-587 | 6.301953395 | 6.587964989 | 6.54225805 | 7.972692654 | 7.075746538 | 7.625708843 | 6.523561956 | 6.953032377 | 6.411087227 | 7.006746832 | 0.854535769 | 0.081430024 |
| hsa-miR-588 | 8.862017377 | 6.292781749 | 6.383704292 | 6.529820947 | 13.55601501 | 7.316960287 | 6.930145948 | 13.05706098 | 11.3810562 | 6.714245518 | 0.093373291 | 2.8088329 |
| hsa-miR-589 | 13.35328469 | 9.28748153 | 10.52503135 | 11.30691611 | 12.82348666 | 8.710462074 | 13.39700607 | 13.83329532 | 13.87028765 | 11.15785217 | 0.351726086 | 1.180553238 |
| hsa-miR-589* | 6.900866808 | 6.587964989 | 6.451211112 | 6.663913842 | 7.075746538 | 8.15810472 | 6.930145948 | 6.790511412 | 7.117902789 | 7.141596278 | 0.040922924 | 0.551345426 |
| hsa-miR-590-3p | 6.105384749 | 6.273329387 | 6.318316841 | 6.512542955 | 8.381110294 | 7.921245889 | 7.78496195 | 7.857047916 | 8.453682186 | 7.712870868 | 4.74E-06 | 1.716093034 |
| hsa-miR-590-5p | 6.151777655 | 6.339850003 | 6.422905743 | 6.663913842 | 9.857825524 | 9.101450482 | 9.726899318 | 8.25502857 | 10.31118066 | 9.425740424 | 4.92E-05 | 3.051742352 |
| hsa-miR-591 | 8.564340058 | 8.71630504 | 8.510170751 | 8.710462074 | 10.30628959 | 9.809125002 | 10.06433859 | 9.924812504 | 10.36888837 | 9.958697703 | 1.34E-06 | 1.446705811 |
| hsa-miR-592 | 7.536441505 | 7.987548259 | 7.721099189 | 7.947198584 | 11.03981011 | 9.768101599 | 10.76088624 | 9.821455119 | 10.82332743 | 9.688862232 | 5.54E-05 | 2.519001905 |
| hsa-miR-593 | 7.061776198 | 6.925999419 | 6.789859641 | 7.293701542 | 7.799605422 | 8.25502857 | 8.167418146 | 8.203837384 | 7.635173947 | 8.248876343 | 0.000186604 | 1.033822435 |
| hsa-miR-593* | 6.019034669 | 6.561478892 | 6.559950613 | 6.473299871 | 8.259507895 | 11.54099979 | 8.607700188 | 8.203837384 | 8.11997861 | 6.857980995 | 0.017543387 | 2.194893132 |
| hsa-miR-594:9.1 | 12.45213826 | 11.97799537 | 12.45213826 | 11.6092711 | 6.862947248 | 6.465974465 | 6.356672044 | 6.422905743 | 6.584962501 | 6.329123596 | 1.54E-05 | -5.619121481 |
| hsa-miR-595 | 13.05706098 | 8.156083076 | 8.747521673 | 10.37585398 | 7.484218708 | 11.05372229 | 12.50061784 | 13.84325371 | 13.35328469 | 10.93199324 | 0.351826306 | 1.443718486 |
| hsa-miR-596 | 7.74348804 | 8.084808388 | 8.021812824 | 8.227856573 | 8.381110294 | 8.950993232 | 8.002252452 | 11.7417619 | 7.117902789 | 8.647638302 | 0.277012812 | 0.787451705 |
| hsa-miR-597 | 6.731997787 | 7.176422513 | 7.062855655 | 7.594324604 | 7.075746538 | 6.465974465 | 7.78496195 | 7.23935985 | 7.117902789 | 7.39145842 | 0.884146268 | 0.037833862 |
| hsa-miR-598 | 9.236970886 | 10.54515755 | 9.77016832 | 10.04916787 | 6.274261661 | 6.609548139 | 6.523561956 | 6.332707934 | 6.250772132 | 12.50061784 | 0.058828911 | -2.485121214 |
| hsa-miR-599 | 6.001126665 | 6.089582893 | 6.145677455 | 6.986410935 | 6.530601415 | 6.755555262 | 6.930145948 | 6.953032377 | 6.83541884 | 6.714245518 | 0.123638427 | 0.480800406 |
| hsa-miR-600 | 6.711494907 | 7.015693807 | 7.175923742 | 7.357552005 | 8.518849829 | 8.819061399 | 8.730809755 | 8.003096215 | 8.725877459 | 8.910492832 | 6.12E-05 | 1.552865133 |
| hsa-miR-601 | 6.517275693 | 6.176921112 | 6.23457796 | 6.300123725 | 7.273795599 | 7.625708843 | 6.930145948 | 7.1017131 | 7.864805194 | 6.569855608 | 0.003632644 | 0.920446093 |
| hsa-miR-602 | 6.452858965 | 6.965784285 | 6.824004051 | 6.918863237 | 6.671010241 | 6.465974465 | 6.523561956 | 6.543805176 | 6.83541884 | 8.405566974 | 0.730830925 | 0.117178641 |
| hsa-miR-603 | 8.452035274 | 7.006746832 | 6.920055055 | 7.572131751 | 8.518849829 | 8.894817763 | 10.52209107 | 10.82631014 | 10.97763769 | 8.324180547 | 0.007307893 | 2.189572278 |
| hsa-miR-604 | 6.383704292 | 6.738767837 | 7.542644987 | 6.105384749 | 5.980710829 | 5.992088609 | 6.207502459 | 5.988684687 | 6.250772132 | 6.100136671 | 0.14609024 | -0.605976235 |
| hsa-miR-605 | 6.77478706 | 6.199672345 | 6.292781749 | 6.776762002 | 7.273795599 | 7.501040996 | 7.145677455 | 8.60936345 | 8.300123725 | 10.89231512 | 0.025733329 | 1.776051936 |
| hsa-miR-606 | 5.752213368 | 5.876516947 | 5.855491443 | 5.910492832 | 5.905687849 | 5.856736756 | 5.87282876 | 5.820178962 | 5.888743249 | 5.842978832 | 0.686474405 | 0.015847087 |
| hsa-miR-607 | 7.230741003 | 7.970393538 | 7.302867361 | 8.047669251 | 8.381110294 | 8.344073938 | 8.889199726 | 8.60936345 | 8.11997861 | 8.814422247 | 0.016133085 | 0.888440256 |
| hsa-miR-608 | 6.5360529 | 8.506605116 | 7.323279988 | 6.348728154 | 6.530601415 | 6.951867504 | 6.207502459 | 5.988684687 | 5.9795681 | 7.502235115 | 0.293417335 | -0.651923326 |
| hsa-miR-609 | 8.346513733 | 8.3226042 | 8.381110294 | 8.680535344 | 10.99957727 | 10.4268937 | 11.00562455 | 9.233739497 | 11.1909358 | 10.19789241 | 0.000644868 | 2.076419646 |
| hsa-miR-610 | 8.23457796 | 7.083213368 | 6.653203362 | 6.876516947 | 6.862947248 | 6.465974465 | 6.721782768 | 6.261154673 | 6.250772132 | 7.006746832 | 0.17859371 | -0.616981556 |
| hsa-miR-611 | 5.911691582 | 5.966937979 | 6.010108453 | 6.136478518 | 5.980710829 | 5.992088609 | 6.089582893 | 6.261154673 | 5.9795681 | 6.001126665 | 0.521297033 | 0.044401162 |
| hsa-miR-612 | 8.088523257 | 8.275193334 | 8.656067274 | 9.692266772 | 12.32673844 | 6.951867504 | 12.97835296 | 6.543805176 | 13.63051741 | 12.40167953 | 0.167566341 | 2.127480844 |
| hsa-miR-613 | 7.290940402 | 7.537606691 | 7.353146825 | 7.083213368 | 8.607700188 | 8.15810472 | 8.289557848 | 8.003096215 | 7.864805194 | 8.248876343 | 0.000265846 | 0.87912993 |
| hsa-miR-614 | 9.451211112 | 9.824322351 | 8.670656249 | 8.506605116 | 9.484420132 | 9.49645436 | 10.39687232 | 9.688862232 | 9.852139328 | 9.044667331 | 0.192381664 | 0.547370577 |
| hsa-miR-615-3p | 10.94807516 | 10.92577695 | 11.1909358 | 10.9918761 | 11.54099979 | 11.06709819 | 11.82137539 | 11.2444233 | 11.79445648 | 11.16804527 | 0.023562424 | 0.425233736 |
| hsa-miR-615-5p | 8.68580002 | 7.617651119 | 7.73470962 | 10.11517373 | 9.62580015 | 10.50476934 | 10.65391987 | 8.858291887 | 8.838573653 | 9.996897126 | 0.128666143 | 1.208041715 |
| hsa-miR-616 | 7.092757141 | 6.653919873 | 6.199672345 | 6.004501392 | 5.980710829 | 5.911691582 | 5.988684687 | 6.261154673 | 6.10433666 | 5.92243574 | 0.153983473 | -0.459543659 |
| hsa-miR-616* | 6.433794059 | 6.236492618 | 7.602142091 | 6.483815777 | 7.982993575 | 7.75087444 | 8.413204789 | 8.003096215 | 8.453682186 | 6.714245518 | 0.02188772 | 1.197288317 |
| hsa-miR-617 | 7.162894799 | 7.116863758 | 7.099084761 | 7.250772132 | 7.799605422 | 7.316960287 | 7.400025518 | 7.661778098 | 7.864805194 | 8.168922782 | 0.007165737 | 0.544612355 |
| hsa-miR-618 | 7.850499414 | 8.397460726 | 8.287943192 | 8.892694591 | 9.32395546 | 8.15810472 | 6.721782768 | 8.326204751 | 8.11997861 | 7.006746832 | 0.378715529 | -0.414353957 |
| hsa-miR-619 | 7.098032083 | 7.790511412 | 7.236014192 | 7.608069968 | 8.68580002 | 8.819061399 | 8.730809755 | 8.15810472 | 9.098821663 | 8.498849207 | 0.000730032 | 1.232084213 |
| hsa-miR-620 | 7.011227255 | 6.854868383 | 7.001126665 | 7.394033895 | 8.68580002 | 9.024308597 | 7.78496195 | 8.203837384 | 8.11997861 | 8.168922782 | 0.000418643 | 1.265987507 |
| hsa-miR-621 | 6.046578367 | 6.029011087 | 5.988684687 | 6.105384749 | 7.982993575 | 6.951867504 | 7.400025518 | 7.515305605 | 7.864805194 | 6.857980995 | 0.000648625 | 1.38641501 |
| hsa-miR-622 | 13.83329532 | 9.32395546 | 10.5844928 | 11.67330908 | 13.98751273 | 13.88718253 | 13.94706157 | 14.09416843 | 14.11566957 | 13.45752196 | 0.074305387 | 2.561089636 |
| hsa-miR-623 | 7.119459934 | 6.569855608 | 8.170926525 | 6.374170076 | 6.530601415 | 6.465974465 | 6.721782768 | 7.596935142 | 7.117902789 | 13.26034622 | 0.46656167 | 0.890320764 |
| hsa-miR-624 | 7.126188211 | 8.293471649 | 7.802839441 | 7.790511412 | 8.518849829 | 10.09882166 | 9.082149041 | 8.830990481 | 12.26617162 | 10.15000109 | 0.012364405 | 2.071244611 |
| hsa-miR-624* | 8.919459269 | 10.51914479 | 10.50809187 | 8.975274788 | 9.969170641 | 9.315715658 | 10.17816685 | 8.812498225 | 10.21188829 | 13.34410169 | 0.487754282 | 0.574764215 |
| hsa-miR-625 | 11.65745244 | 10.38046107 | 10.69156906 | 13.24425976 | 12.6077233 | 11.32187174 | 12.55837288 | 12.6077233 | 12.97835296 | 6.329123596 | 0.941407342 | -0.092907619 |
| hsa-miR-625* | 10.07614753 | 10.12399203 | 9.13481133 | 10.15000109 | 9.412569847 | 9.715533064 | 9.40779885 | 9.082149041 | 9.456765088 | 10.09354961 | 0.279906476 | -0.343177079 |
| hsa-miR-626 | 7.444600814 | 6.807354922 | 6.802193217 | 10.21188829 | 13.30457949 | 9.177419538 | 9.40779885 | 11.89958351 | 9.456765088 | 7.925109324 | 0.074856662 | 2.378699987 |
| hsa-miR-627 | 8.364134655 | 9.885543861 | 10.41309898 | 8.250298418 | 6.155830172 | 6.316507819 | 8.986979709 | 6.261154673 | 6.250772132 | 6.714245518 | 0.011252118 | -2.447353976 |
| hsa-miR-628-3p | 10.68088692 | 11.06709819 | 11.44403133 | 12.04668067 | 7.799605422 | 12.50061784 | 6.523561956 | 6.668175869 | 7.117902789 | 6.569855608 | 0.013581629 | -3.446387695 |
| hsa-miR-628-5p | 11.139935 | 11.1909358 | 11.28320413 | 11.82137539 | 11.30691611 | 13.92203613 | 11.64029013 | 9.66035251 | 11.25844826 | 11.80779765 | 0.694156204 | 0.240444219 |
| hsa-miR-629 | 9.49645436 | 7.753885283 | 7.452035274 | 6.794415866 | 8.607700188 | 7.921245889 | 8.889199726 | 8.15810472 | 8.725877459 | 7.502235115 | 0.528239209 | 0.426529487 |
| hsa-miR-629* | 7.343407822 | 5.894817763 | 5.988684687 | 8.346513733 | 5.980710829 | 6.465974465 | 5.988684687 | 6.422905743 | 6.250772132 | 6.194756854 | 0.333830505 | -0.676055217 |
| hsa-miR-630 | 6.168922782 | 6.236492618 | 6.250772132 | 6.529820947 | 7.273795599 | 7.501040996 | 7.78496195 | 12.67185062 | 7.408117408 | 6.857980995 | 0.080443844 | 1.953122476 |
| hsa-miR-631 | 7.098032083 | 7.064473329 | 8.185866545 | 6.957102042 | 9.248164779 | 8.60936345 | 8.986979709 | 8.689299161 | 9.207990433 | 8.248876343 | 0.006514742 | 1.50541048 |
| hsa-miR-632 | 13.5187515 | 9.306175642 | 10.37123213 | 11.29582632 | 13.88718253 | 13.90966811 | 13.24425976 | 14.01553701 | 14.04598144 | 13.55601501 | 0.057995353 | 2.653444249 |
| hsa-miR-633 | 6.121015401 | 6.154818109 | 6.302867361 | 6.559950613 | 8.800252806 | 8.25502857 | 7.599168993 | 6.953032377 | 7.635173947 | 7.502235115 | 0.001479983 | 1.50615243 |
| hsa-miR-634 | 6.340740281 | 6.636624621 | 6.699051844 | 7.14363831 | 9.792139552 | 9.443565213 | 10.00070427 | 8.797337288 | 9.852139328 | 8.405566974 | 3.14E-05 | 2.676895007 |
| hsa-miR-635 | 5.930737338 | 6.636624621 | 6.510961919 | 8.185866545 | 5.980710829 | 6.196725061 | 5.927185358 | 6.0725346 | 6.10433666 | 6.194756854 | 0.223749079 | -0.736672712 |
| hsa-miR-636 | 6.794415866 | 7.161887682 | 6.027905997 | 6.136478518 | 6.671010241 | 6.465974465 | 6.523561956 | 6.543805176 | 6.250772132 | 6.001126665 | 0.697218209 | -0.12079691 |
| hsa-miR-637 | 6.36719631 | 6.715618859 | 6.676662335 | 6.601399391 | 6.399171094 | 5.992088609 | 6.356672044 | 5.988684687 | 6.250772132 | 6.001126665 | 0.005577345 | -0.425466685 |
| hsa-miR-638 | 9.312656099 | 7.980710829 | 9.6121313 | 5.910492832 | 6.155830172 | 6.196725061 | 6.721782768 | 6.261154673 | 6.250772132 | 5.92243574 | 0.102376932 | -1.952547674 |
| hsa-miR-639 | 6.255500733 | 6.053111336 | 6.815063017 | 6.105384749 | 6.274261661 | 13.30457949 | 5.927185358 | 6.0725346 | 6.10433666 | 6.100136671 | 0.450828084 | 0.989907447 |
| hsa-miR-640 | 6.881419908 | 6.21916852 | 6.292781749 | 6.354028938 | 6.671010241 | 6.465974465 | 6.356672044 | 6.668175869 | 7.864805194 | 7.006746832 | 0.175811884 | 0.402047662 |
| hsa-miR-641 | 7.525129251 | 8.026246773 | 8.488442705 | 7.99095486 | 7.799605422 | 8.040837649 | 8.607700188 | 7.857047916 | 8.453682186 | 8.689299161 | 0.388987985 | 0.23366869 |
| hsa-miR-642 | 11.25844826 | 10.44935502 | 11.72970571 | 11.06709819 | 6.155830172 | 6.196725061 | 6.356672044 | 6.0725346 | 5.9795681 | 6.194756854 | 0.000220869 | -4.966803988 |
| hsa-miR-643 | 7.719731057 | 7.477353527 | 8.773468928 | 7.563768278 | 8.381110294 | 7.316960287 | 8.289557848 | 7.661778098 | 7.408117408 | 8.405566974 | 0.943650702 | 0.026934704 |
| hsa-miR-644 | 8.95419631 | 7.887525271 | 6.268658955 | 7.867896464 | 7.075746538 | 7.316960287 | 7.599168993 | 8.956666555 | 7.408117408 | 6.857980995 | 0.754708156 | -0.208795787 |
| hsa-miR-645 | 8.437544026 | 7.685099172 | 7.571373436 | 7.883254231 | 8.518849829 | 6.755555262 | 7.400025518 | 10.37123213 | 6.584962501 | 7.006746832 | 0.851552027 | -0.121422371 |
| hsa-miR-646 | 9.631540867 | 8.328226119 | 9.156335937 | 9.49645436 | 13.68986697 | 10.00450139 | 10.82631014 | 11.37444181 | 10.82332743 | 13.39700607 | 0.007613416 | 2.532769647 |
| hsa-miR-647 | 8.339850003 | 7.806710718 | 7.898752526 | 8.063395081 | 10.52503135 | 9.981995318 | 10.48290877 | 9.97053734 | 10.99957727 | 9.726899318 | 1.16E-05 | 2.253981146 |
| hsa-miR-648 | 6.661778098 | 6.399171094 | 6.339850003 | 6.54225805 | 8.381110294 | 8.040837649 | 7.78496195 | 8.15810472 | 9.098821663 | 8.498849207 | 6.22E-05 | 1.841349936 |
| hsa-miR-649 | 6.881419908 | 6.678071905 | 8.392961339 | 6.944858446 | 7.982993575 | 7.625708843 | 7.599168993 | 8.25502857 | 7.408117408 | 7.141596278 | 0.355144649 | 0.444441045 |
| hsa-miR-650 | 6.444600814 | 7.695228291 | 6.175923742 | 6.136478518 | 6.671010241 | 6.609548139 | 6.930145948 | 6.668175869 | 6.411087227 | 6.569855608 | 0.940261859 | 0.030245997 |
| hsa-miR-651 | 8.423746229 | 8.522581531 | 8.147713722 | 8.356672044 | 9.67357409 | 9.537703748 | 10.08308037 | 9.462297766 | 9.958697703 | 9.202491187 | 4.45E-05 | 1.290295762 |
| hsa-miR-652 | 12.53225851 | 11.2062818 | 11.59577031 | 10.71020375 | 9.412569847 | 8.344073938 | 9.258330473 | 9.05853297 | 8.959132577 | 11.12023788 | 0.004780829 | -2.152315645 |
| hsa-miR-653 | 7.24697806 | 7.617651119 | 7.687900522 | 7.726558779 | 9.412569847 | 13.26034622 | 9.507001733 | 8.522581531 | 13.50039377 | 8.589838376 | 0.026846157 | 2.895683126 |
| hsa-miR-653:9.1 | 6.619853147 | 7.441699257 | 8.58082385 | 7.394033895 | 9.920427296 | 9.595443985 | 9.958697703 | 8.897542973 | 10.027906 | 8.405566974 | 0.007873735 | 1.95849495 |
| hsa-miR-654-3p | 13.74706006 | 10.2701785 | 10.35469017 | 11.7417619 | 14.11566957 | 13.57510193 | 13.7905725 | 14.07082534 | 13.65089467 | 13.74706006 | 0.065525755 | 2.296598022 |
| hsa-miR-654-5p | 8.58082385 | 9.096188046 | 8.423746229 | 8.405566974 | 10.84172158 | 10.06743436 | 10.60385812 | 13.41629614 | 13.67325386 | 8.498849207 | 0.025368774 | 2.556987603 |
| hsa-miR-655 | 6.019034669 | 7.085339669 | 7.718361626 | 6.078951341 | 6.530601415 | 6.196725061 | 6.721782768 | 6.543805176 | 6.83541884 | 6.329123596 | 0.666685624 | -0.199179017 |
| hsa-miR-656 | 5.953032377 | 5.894817763 | 6.027905997 | 6.053111336 | 6.399171094 | 6.316507819 | 6.721782768 | 6.543805176 | 6.584962501 | 6.329123596 | 0.000228546 | 0.500341957 |
| hsa-miR-657 | 7.24697806 | 7.046578367 | 6.93191939 | 7.384567923 | 9.857825524 | 10.12805878 | 10.31118066 | 9.595443985 | 9.852139328 | 9.477859434 | 1.70E-07 | 2.717907017 |
| hsa-miR-658 | 7.414473836 | 6.046578367 | 6.121015401 | 6.196725061 | 6.862947248 | 6.196725061 | 6.523561956 | 6.543805176 | 6.83541884 | 6.857980995 | 0.60726948 | 0.192041713 |
| hsa-miR-659 | 7.395319911 | 7.216745858 | 7.384567923 | 7.542644987 | 7.075746538 | 7.316960287 | 6.930145948 | 6.543805176 | 7.117902789 | 7.39145842 | 0.054727153 | -0.32214981 |
| hsa-miR-660 | 7.074141463 | 7.176422513 | 6.762880293 | 7.074141463 | 8.893756568 | 8.60936345 | 9.082149041 | 8.326204751 | 8.725877459 | 8.324180547 | 5.34E-06 | 1.63835887 |
| hsa-miR-661 | 7.032321287 | 6.99095486 | 6.861707287 | 7.537606691 | 7.075746538 | 6.951867504 | 6.930145948 | 7.1017131 | 6.83541884 | 7.006746832 | 0.478701238 | -0.122041071 |
| hsa-miR-662 | 6.085339669 | 5.927185358 | 5.930737338 | 5.861707287 | 5.980710829 | 6.092757141 | 5.87282876 | 6.0725346 | 11.09275714 | 6.001126665 | 0.337441826 | 0.900876776 |
| hsa-miR-663 | 11.34318572 | 10.24257869 | 10.38046107 | 10.06743436 | 6.530601415 | 6.316507819 | 6.356672044 | 6.168922782 | 6.584962501 | 6.100136671 | 0.000357271 | -4.165447752 |
| hsa-miR-663b | 10.22363975 | 10.78119576 | 10.4871366 | 10.53974043 | 7.982993575 | 7.75087444 | 8.289557848 | 8.326204751 | 9.098821663 | 8.063395081 | 1.07E-05 | -2.255953574 |
| hsa-miR-664 | 11.16804527 | 11.47126876 | 11.3810562 | 12.28004576 | 7.484218708 | 6.755555262 | 8.002252452 | 11.55588324 | 7.635173947 | 11.72970571 | 0.028007892 | -2.714639113 |
| hsa-miR-664* | 11.08261478 | 10.97763769 | 10.9918761 | 11.08261478 | 9.32395546 | 11.47126876 | 7.599168993 | 7.661778098 | 7.864805194 | 8.814422247 | 0.013906414 | -2.244452712 |
| hsa-miR-665 | 9.223036338 | 8.236492618 | 8.247452865 | 8.675957033 | 8.893756568 | 8.521207816 | 8.607700188 | 9.958697703 | 8.838573653 | 8.647638302 | 0.353814459 | 0.315527658 |
| hsa-miR-668 | 8.255500733 | 8.144658243 | 7.883254231 | 7.314243347 | 10.35110487 | 11.12023788 | 10.08308037 | 9.233739497 | 10.54515755 | 10.99573182 | 0.000101987 | 2.488761194 |
| hsa-miR-671:9.1 | 7.637349411 | 6.896029603 | 7.336283388 | 7.732676223 | 6.671010241 | 6.465974465 | 7.145677455 | 6.668175869 | 6.584962501 | 6.329123596 | 0.017448011 | -0.756430635 |
| hsa-miR-671-3p | 7.141596278 | 8.053654418 | 8.324180547 | 8.612499946 | 12.12350713 | 6.609548139 | 11.40375769 | 11.80779765 | 12.12350713 | 11.31441331 | 0.020181475 | 2.864105709 |
| hsa-miR-671-5p | 6.175923742 | 6.301953395 | 6.372429792 | 6.570614722 | 6.671010241 | 6.755555262 | 6.930145948 | 6.543805176 | 7.408117408 | 7.39145842 | 0.009891417 | 0.594784996 |
| hsa-miR-675 | 7.916775185 | 8.478971805 | 8.437544026 | 7.257859235 | 6.399171094 | 6.465974465 | 6.721782768 | 7.1017131 | 6.250772132 | 6.45779126 | 0.008619604 | -1.456586759 |
| hsa-miR-7 | 10.00450139 | 11.08261478 | 11.2062818 | 10.1321141 | 7.273795599 | 11.89958351 | 8.167418146 | 6.953032377 | 7.408117408 | 8.543805176 | 0.02984391 | -2.232085984 |
| hsa-miR-708 | 10.5844928 | 11.52645033 | 11.29582632 | 10.96079838 | 7.484218708 | 7.117383367 | 8.002252452 | 7.388878339 | 8.11997861 | 6.45779126 | 3.33E-06 | -3.663474835 |
| hsa-miR-708* | 7.312429206 | 7.270996061 | 6.912290583 | 7.279842694 | 7.273795599 | 6.951867504 | 7.145677455 | 6.790511412 | 7.408117408 | 8.498849207 | 0.588831467 | 0.150913462 |
| hsa-miR-7-1* | 14.61467534 | 13.75690028 | 14.18441048 | 14.45914981 | 14.41700199 | 14.51607549 | 14.61467534 | 14.71007995 | 14.61467534 | 14.41700199 | 0.21600538 | 0.294467706 |
| hsa-miR-7-2* | 8.800252806 | 6.089582893 | 6.861707287 | 6.845490051 | 9.14822234 | 6.316507819 | 11.25844826 | 10.54245153 | 6.250772132 | 6.329123596 | 0.32608078 | 1.158329354 |
| hsa-miR-720 | 13.47772033 | 11.79445648 | 12.21577565 | 12.46867495 | 9.14822234 | 13.7905725 | 9.507001733 | 11.88542947 | 9.574309691 | 12.97835296 | 0.17827696 | -1.341842071 |
| hsa-miR-744 | 11.23595438 | 10.42311591 | 10.8560364 | 10.20371505 | 8.893756568 | 8.521207816 | 13.21598794 | 8.106955457 | 8.959132577 | 11.93475974 | 0.438204104 | -0.741072085 |
| hsa-miR-744* | 7.75087444 | 7.602142091 | 7.343407822 | 7.66035251 | 7.982993575 | 7.75087444 | 8.167418146 | 7.753885283 | 8.300123725 | 7.799605422 | 0.021672973 | 0.369955883 |
| hsa-miR-760 | 9.230500854 | 6.469234794 | 6.851749041 | 6.845490051 | 6.274261661 | 6.316507819 | 6.356672044 | 11.29582632 | 6.10433666 | 6.194756854 | 0.812114331 | -0.258850125 |
| hsa-miR-765 | 6.789859641 | 6.930145948 | 6.755555262 | 7.1048608 | 8.381110294 | 9.177419538 | 7.78496195 | 8.25502857 | 8.300123725 | 7.270996061 | 0.003247286 | 1.29983461 |
| hsa-miR-766 | 9.779883475 | 9.101450482 | 9.230500854 | 8.651769271 | 7.641690523 | 12.40167953 | 7.400025518 | 8.003096215 | 7.117902789 | 12.08470875 | 0.938577476 | -0.082717133 |
| hsa-miR-767-3p | 6.139551352 | 6.373300197 | 6.404290064 | 6.54225805 | 6.671010241 | 6.609548139 | 6.523561956 | 6.543805176 | 6.83541884 | 6.569855608 | 0.042737179 | 0.260683411 |
| hsa-miR-767-5p | 9.857825524 | 9.230500854 | 9.32395546 | 7.896635141 | 7.484218708 | 7.117383367 | 7.400025518 | 12.65244168 | 7.117902789 | 8.063395081 | 0.454932818 | -0.771334721 |
| hsa-miR-768-3p:11.0 | 14.23044081 | 14.18441048 | 14.11566957 | 13.96936853 | 7.982993575 | 12.14063808 | 7.599168993 | 7.1017131 | 7.117902789 | 7.502235115 | 0.000671472 | -5.884197074 |
| hsa-miR-768-5p:11.0 | 12.01300855 | 13.24425976 | 13.55601501 | 13.47772033 | 8.893756568 | 8.344073938 | 8.514911265 | 8.003096215 | 9.207990433 | 9.250416861 | 0.000141385 | -4.370376701 |
| hsa-miR-769-3p | 7.703211467 | 7.609548139 | 7.312429206 | 8.018200179 | 8.518849829 | 7.117383367 | 8.167418146 | 7.753885283 | 8.11997861 | 7.006746832 | 0.688555008 | 0.119863096 |
| hsa-miR-769-5p | 9.824322351 | 10.04916787 | 9.827660269 | 10.33918193 | 10.30628959 | 10.4871366 | 10.31118066 | 9.276589716 | 10.31118066 | 10.60385812 | 0.39616833 | 0.205956117 |
| hsa-miR-770-5p | 6.195741293 | 6.529820947 | 6.302867361 | 6.601399391 | 6.274261661 | 6.755555262 | 6.356672044 | 6.422905743 | 6.411087227 | 6.329123596 | 0.886960443 | 0.017477008 |
| hsa-miR-801:9.1 | 7.78496195 | 8.047669251 | 8.115563915 | 6.255500733 | 5.905687849 | 5.911691582 | 5.988684687 | 6.0725346 | 5.888743249 | 6.714245518 | 0.038460102 | -1.470659382 |
| hsa-miR-802 | 6.292781749 | 6.912290583 | 6.74281467 | 7.269594252 | 8.259507895 | 6.951867504 | 6.523561956 | 7.596935142 | 7.864805194 | 7.925109324 | 0.065687153 | 0.715927522 |
| hsa-miR-873 | 6.112700133 | 6.0725346 | 6.154818109 | 6.292781749 | 8.151523994 | 7.501040996 | 8.167418146 | 8.405566974 | 8.838573653 | 7.712870868 | 0.000101051 | 1.971290457 |
| hsa-miR-874 | 10.45491614 | 9.832731815 | 9.67047922 | 8.582706527 | 6.274261661 | 6.196725061 | 6.207502459 | 6.261154673 | 6.83541884 | 6.194756854 | 0.002289562 | -3.306905167 |
| hsa-miR-875-3p | 6.529820947 | 6.733354341 | 6.726558779 | 6.928370323 | 6.862947248 | 7.117383367 | 7.145677455 | 7.1017131 | 7.408117408 | 6.857980995 | 0.017525606 | 0.352777165 |
| hsa-miR-875-5p | 7.77939106 | 9.116863758 | 8.364134655 | 9.069718276 | 12.04668067 | 8.25502857 | 9.223036338 | 12.69522829 | 8.725877459 | 8.961594391 | 0.139463105 | 1.40204735 |
| hsa-miR-876-3p | 6.74281467 | 6.350497247 | 6.404290064 | 6.721782768 | 9.730979533 | 9.177419538 | 9.87175126 | 9.014299495 | 9.692266772 | 8.814422247 | 1.40E-06 | 2.828676953 |
| hsa-miR-876-5p | 6.422905743 | 7.384567923 | 6.676662335 | 6.83541884 | 6.862947248 | 7.316960287 | 6.721782768 | 7.515305605 | 7.117902789 | 7.270996061 | 0.253771552 | 0.304427083 |
| hsa-miR-877 | 10.06743436 | 10.99957727 | 11.33220442 | 11.16804527 | 9.792139552 | 13.38548496 | 13.88718253 | 10.5844928 | 10.1365426 | 13.21598794 | 0.285606831 | 0.941823066 |
| hsa-miR-877* | 6.195741293 | 6.019034669 | 6.68580002 | 6.121015401 | 6.530601415 | 11.72970571 | 6.523561956 | 7.515305605 | 6.411087227 | 6.45779126 | 0.200236193 | 1.272611016 |
| hsa-miR-885-3p | 6.433794059 | 6.399171094 | 6.518062976 | 6.62935662 | 6.399171094 | 6.609548139 | 6.523561956 | 6.543805176 | 6.584962501 | 6.329123596 | 0.963191726 | 0.00326589 |
| hsa-miR-885-5p | 6.619853147 | 6.668175869 | 6.112700133 | 5.970393538 | 7.273795599 | 6.609548139 | 6.721782768 | 6.261154673 | 6.83541884 | 6.329123596 | 0.200614156 | 0.329023264 |
| hsa-miR-886-3p | 10.69156906 | 10.39081383 | 10.4268937 | 9.00365845 | 7.484218708 | 7.117383367 | 7.78496195 | 7.927777962 | 7.408117408 | 7.925109324 | 0.004163847 | -2.52030564 |
| hsa-miR-886-5p | 9.071194179 | 9.685449638 | 9.685449638 | 7.508587112 | 6.399171094 | 6.951867504 | 7.599168993 | 7.23935985 | 7.117902789 | 7.799605422 | 0.031555848 | -1.803157533 |
| hsa-miR-887 | 7.505414609 | 7.920055055 | 7.425845306 | 7.628627802 | 7.641690523 | 7.921245889 | 8.607700188 | 8.725877459 | 8.453682186 | 8.063395081 | 0.017729524 | 0.615612861 |
| hsa-miR-888 | 6.619853147 | 7.183883459 | 7.353146825 | 7.062855655 | 7.799605422 | 7.921245889 | 8.289557848 | 8.003096215 | 8.453682186 | 7.39145842 | 0.003531245 | 0.921506225 |
| hsa-miR-888* | 6.074676686 | 6.008988783 | 7.225930411 | 6.224966365 | 6.399171094 | 6.196725061 | 6.523561956 | 6.543805176 | 6.584962501 | 6.329123596 | 0.883779859 | 0.045917669 |
| hsa-miR-889 | 9.053654418 | 11.30691611 | 11.26982798 | 9.97053734 | 10.54515755 | 10.99957727 | 10.00070427 | 9.11204848 | 10.1365426 | 11.18090371 | 0.914206542 | -0.071078315 |
| hsa-miR-890 | 8.336283388 | 8.603626345 | 8.488442705 | 8.6387979 | 10.21188829 | 9.49645436 | 9.821455119 | 9.05853297 | 9.958697703 | 10.06433859 | 0.000398322 | 1.251773589 |
| hsa-miR-891a | 8.147713722 | 7.710117632 | 6.194756854 | 7.14363831 | 6.399171094 | 5.911691582 | 6.207502459 | 6.168922782 | 6.10433666 | 6.100136671 | 0.070327844 | -1.150429755 |
| hsa-miR-891b | 5.847996907 | 5.894817763 | 5.911691582 | 5.94016675 | 6.05202456 | 5.856736756 | 6.089582893 | 5.988684687 | 5.9795681 | 5.842978832 | 0.169075881 | 0.069594388 |
| hsa-miR-892a | 5.984133595 | 6.063934306 | 6.145677455 | 6.053111336 | 6.530601415 | 5.992088609 | 6.089582893 | 6.0725346 | 6.10433666 | 6.329123596 | 0.208286832 | 0.124663789 |
| hsa-miR-892b | 6.452858965 | 6.089582893 | 6.091699834 | 6.196725061 | 6.862947248 | 6.755555262 | 6.523561956 | 6.668175869 | 7.408117408 | 6.857980995 | 0.002904691 | 0.638339768 |
| hsa-miR-9 | 6.961160258 | 11.64029013 | 10.61369739 | 9.403012024 | 6.399171094 | 6.316507819 | 6.356672044 | 6.543805176 | 6.584962501 | 6.329123596 | 0.048837012 | -3.232832914 |
| hsa-miR-9* | 10.8622499 | 9.722807531 | 8.539158811 | 8.838573653 | 12.94553528 | 11.36779694 | 8.413204789 | 13.50039377 | 11.80779765 | 12.26617162 | 0.038371231 | 2.226119202 |
| hsa-miR-920 | 7.315602457 | 7.572131751 | 7.703211467 | 7.365884968 | 10.1365426 | 9.963257357 | 10.52209107 | 9.688862232 | 10.42500604 | 9.276589716 | 7.42E-06 | 2.512850509 |
| hsa-miR-921 | 6.60659028 | 7.225930411 | 7.078417702 | 7.410239331 | 6.671010241 | 8.040837649 | 7.599168993 | 7.23935985 | 7.117902789 | 8.862017377 | 0.199713961 | 0.508088386 |
| hsa-miR-922 | 7.414473836 | 7.790511412 | 7.469234794 | 8.211644987 | 7.273795599 | 7.625708843 | 7.145677455 | 7.1017131 | 6.83541884 | 9.958697703 | 0.902401008 | -0.064631 |
| hsa-miR-923 | 12.36194377 | 13.90966811 | 13.87028765 | 13.41629614 | 11.88542947 | 11.7417619 | 6.523561956 | 6.168922782 | 6.411087227 | 7.270996061 | 0.004937669 | -5.055922354 |
| hsa-miR-924 | 7.491853096 | 6.597680144 | 6.694880193 | 9.377644358 | 11.22427306 | 9.939285356 | 11.20939244 | 9.97053734 | 11.52645033 | 10.60385812 | 0.0094978 | 3.205118327 |
| hsa-miR-92a | 13.67325386 | 13.84325371 | 13.75690028 | 13.88718253 | 7.075746538 | 14.02323456 | 13.75690028 | 7.1017131 | 13.39700607 | 14.23044081 | 0.185938844 | -2.192640702 |
| hsa-miR-92a-1* | 7.400025518 | 7.896635141 | 9.312656099 | 7.180406485 | 7.484218708 | 6.755555262 | 7.145677455 | 6.790511412 | 7.864805194 | 7.799605422 | 0.283532724 | -0.640701902 |
| hsa-miR-92a-2* | 6.61176256 | 6.170926525 | 6.121015401 | 7.266786541 | 6.671010241 | 6.465974465 | 8.002252452 | 8.977852308 | 8.597307691 | 6.45779126 | 0.105258589 | 0.986075313 |
| hsa-miR-92b | 11.2062818 | 11.41494944 | 11.42059188 | 12.32673844 | 10.90914305 | 9.715533064 | 11.11155955 | 9.567385727 | 11.14539087 | 10.51914479 | 0.020417817 | -1.097447549 |
| hsa-miR-92b* | 7.982993575 | 6.95070169 | 7.519243094 | 6.300123725 | 6.671010241 | 6.609548139 | 6.930145948 | 6.332707934 | 6.83541884 | 6.45779126 | 0.229447481 | -0.548828461 |
| hsa-miR-93 | 12.89026428 | 13.38548496 | 13.30457949 | 13.40900634 | 6.671010241 | 12.82348666 | 6.930145948 | 6.543805176 | 6.411087227 | 6.714245518 | 0.002793444 | -5.565036973 |
| hsa-miR-93* | 8.416164165 | 8.536441505 | 7.588714636 | 7.92243574 | 7.484218708 | 7.316960287 | 7.78496195 | 6.953032377 | 7.408117408 | 6.329123596 | 0.019613291 | -0.90320329 |
| hsa-miR-933 | 6.053111336 | 6.292781749 | 6.43629512 | 6.928370323 | 7.273795599 | 6.465974465 | 6.721782768 | 6.953032377 | 8.11997861 | 6.569855608 | 0.094520918 | 0.589763606 |
| hsa-miR-934 | 6.325530332 | 6.597680144 | 6.569855608 | 6.83541884 | 6.399171094 | 7.117383367 | 6.721782768 | 6.953032377 | 6.584962501 | 7.141596278 | 0.178634515 | 0.2375335 |
| hsa-miR-935 | 7.712870868 | 8.453682186 | 8.982708429 | 8.643495471 | 6.530601415 | 6.196725061 | 6.356672044 | 6.543805176 | 6.584962501 | 6.329123596 | 0.003634526 | -2.02454094 |
| hsa-miR-936 | 7.943686952 | 7.528258743 | 7.640606469 | 8.073606041 | 11.02160465 | 10.86712427 | 14.02323456 | 10.99573182 | 11.88542947 | 10.24257869 | 0.000807082 | 3.709411024 |
| hsa-miR-937 | 6.340740281 | 6.154818109 | 6.632995197 | 6.171927354 | 6.399171094 | 6.316507819 | 6.089582893 | 6.0725346 | 6.83541884 | 6.001126665 | 0.820873895 | -0.039396583 |
| hsa-miR-938 | 14.27431991 | 11.77020962 | 12.67185062 | 13.55601501 | 14.45914981 | 14.27431991 | 14.37188552 | 14.45914981 | 14.41700199 | 14.18441048 | 0.096959879 | 1.292887463 |
| hsa-miR-939 | 10.9918761 | 11.29582632 | 11.2444233 | 11.01164658 | 11.82137539 | 11.34318572 | 12.07271881 | 11.41494944 | 12.04668067 | 11.31441331 | 0.013308882 | 0.532944147 |
| hsa-miR-940 | 13.61622719 | 11.02873489 | 11.82137539 | 12.67185062 | 14.04598144 | 13.83329532 | 13.98751273 | 13.96936853 | 14.02323456 | 13.87028765 | 0.057270687 | 1.67039968 |
| hsa-miR-941 | 11.1028287 | 8.75788999 | 8.338067798 | 6.483815777 | 6.530601415 | 7.316960287 | 6.356672044 | 12.42385126 | 7.635173947 | 6.569855608 | 0.537877769 | -0.865131473 |
| hsa-miR-942 | 9.650154214 | 8.379378367 | 8.937520951 | 7.328226119 | 5.980710829 | 5.911691582 | 5.988684687 | 5.902073579 | 6.10433666 | 11.59577031 | 0.159136651 | -1.659941972 |
| hsa-miR-943 | 7.074141463 | 7.424586226 | 7.236014192 | 9.533719071 | 8.381110294 | 8.25502857 | 8.607700188 | 8.203837384 | 8.959132577 | 7.799605422 | 0.416989662 | 0.550620501 |
| hsa-miR-944 | 6.175923742 | 7.075746538 | 6.105384749 | 9.548436625 | 13.5187515 | 10.97763769 | 6.356672044 | 6.261154673 | 6.411087227 | 13.07437565 | 0.217336063 | 2.206906883 |
| hsa-miR-95 | 9.929554316 | 10.52503135 | 10.92577695 | 10.89231512 | 7.641690523 | 6.465974465 | 6.721782768 | 6.668175869 | 6.584962501 | 6.194756854 | 5.32E-06 | -3.855278936 |
| hsa-miR-96 | 8.300123725 | 9.456765088 | 9.66035251 | 9.082149041 | 7.075746538 | 6.465974465 | 6.523561956 | 6.543805176 | 6.584962501 | 7.141596278 | 0.001715468 | -2.402239772 |
| hsa-miR-96* | 6.229299509 | 6.224966365 | 6.314696526 | 6.447909749 | 6.399171094 | 6.316507819 | 6.356672044 | 7.23935985 | 6.411087227 | 6.569855608 | 0.156343912 | 0.24455757 |
| hsa-miR-98 | 11.15434346 | 11.72144102 | 11.48316077 | 10.93199324 | 7.273795599 | 6.755555262 | 7.145677455 | 6.790511412 | 6.83541884 | 6.714245518 | 5.28E-06 | -4.403533941 |
| hsa-miR-99a | 12.6077233 | 13.12847772 | 12.55837288 | 12.69522829 | 7.273795599 | 7.316960287 | 6.721782768 | 7.388878339 | 8.300123725 | 7.006746832 | 4.70E-08 | -5.412735955 |
| hsa-miR-99a* | 7.408117408 | 7.502235115 | 7.74348804 | 7.526694846 | 8.259507895 | 8.437544026 | 8.607700188 | 8.15810472 | 8.597307691 | 7.625708843 | 0.0031393 | 0.735845042 |
| hsa-miR-99b | 12.32673844 | 11.26982798 | 11.1028287 | 10.68088692 | 6.862947248 | 7.501040996 | 7.145677455 | 6.790511412 | 7.408117408 | 5.92243574 | 6.85E-05 | -4.406615467 |
| hsa-miR-99b* | 10.76088624 | 9.421644094 | 9.832731815 | 9.044667331 | 8.68580002 | 8.521207816 | 8.730809755 | 11.5740258 | 9.098821663 | 7.799605422 | 0.312853419 | -0.696603958 |
| solexa-1460-671 | 9.28748153 | 7.867896464 | 6.767522386 | 8.122051448 | 6.155830172 | 5.992088609 | 6.207502459 | 6.261154673 | 6.411087227 | 6.194756854 | 0.038766085 | -1.807501291 |
| solexa-15-44487 | 8.440453944 | 8.470048726 | 8.345183447 | 8.512542955 | 11.18592847 | 10.51914479 | 11.28320413 | 10.33918193 | 11.28952903 | 10.40290547 | 3.65E-05 | 2.394591704 |
| solexa-2502-366 | 5.968090752 | 6.663202279 | 6.112700133 | 6.171927354 | 5.980710829 | 6.609548139 | 6.721782768 | 6.790511412 | 6.83541884 | 6.194756854 | 0.200439876 | 0.293141344 |
| solexa-2526-361 | 6.824640581 | 7.123604124 | 6.762880293 | 7.083213368 | 7.484218708 | 7.117383367 | 7.400025518 | 7.661778098 | 7.408117408 | 7.502235115 | 0.005174488 | 0.480375111 |
| solexa-2580-353 | 6.069315495 | 6.762880293 | 6.469234794 | 6.759555343 | 7.799605422 | 6.196725061 | 6.089582893 | 5.942514505 | 6.10433666 | 7.39145842 | 0.848405895 | 0.072124013 |
| solexa-2683-338 | 6.404290064 | 5.944858446 | 5.894817763 | 5.923624611 | 5.837943242 | 5.911691582 | 5.87282876 | 5.902073579 | 5.716990894 | 5.842978832 | 0.207263741 | -0.194479906 |
| solexa-2952-306 | 13.26034622 | 13.28896698 | 13.21598794 | 13.19746245 | 6.274261661 | 11.48316077 | 6.207502459 | 6.543805176 | 6.10433666 | 8.75788999 | 0.001355423 | -5.678864777 |
| solexa-3022-299 | 8.255500733 | 8.347842794 | 8.255500733 | 8.379378367 | 9.730979533 | 9.315715658 | 9.920427296 | 9.233739497 | 9.63499251 | 9.315715658 | 6.12E-05 | 1.215706035 |
| solexa-3044-295 | 8.379378367 | 8.95419631 | 8.470048726 | 9.123086751 | 7.641690523 | 6.951867504 | 7.400025518 | 8.25502857 | 7.117902789 | 6.569855608 | 0.001570396 | -1.40894912 |
| solexa-3126-285 | 8.255500733 | 9.827660269 | 9.480992104 | 6.292781749 | 7.273795599 | 6.951867504 | 7.400025518 | 12.33776126 | 7.408117408 | 6.569855608 | 0.700550986 | -0.473996564 |
| solexa-3277-272 | 7.24697806 | 7.663202279 | 7.242221395 | 7.180406485 | 9.484420132 | 7.921245889 | 8.514911265 | 9.783898589 | 8.725877459 | 8.498849207 | 0.002216016 | 1.488331702 |
| solexa-3464-254 | 12.90809234 | 11.51491126 | 12.3835693 | 10.04384754 | 13.47772033 | 14.37188552 | 14.04598144 | 12.04668067 | 12.94553528 | 11.7417619 | 0.119173223 | 1.392322412 |
| solexa-3695-237 | 6.040015679 | 5.911691582 | 7.759222426 | 5.910492832 | 6.155830172 | 6.196725061 | 6.356672044 | 10.8622499 | 6.83541884 | 6.194756854 | 0.455635474 | 0.694919849 |
| solexa-3793-229 | 6.619853147 | 7.299665944 | 7.561478892 | 8.427522371 | 7.641690523 | 7.75087444 | 7.400025518 | 7.388878339 | 8.11997861 | 12.62956153 | 0.306974379 | 1.011371405 |
| solexa-3927-221 | 14.71007995 | 14.45914981 | 14.71007995 | 14.04598144 | 8.381110294 | 12.62956153 | 7.400025518 | 6.790511412 | 8.11997861 | 13.59243368 | 0.00777065 | -4.995719283 |
| solexa-4793-177 | 10.4268937 | 10.89231512 | 10.24257869 | 9.939285356 | 10.76088624 | 11.18090371 | 10.71896091 | 9.852139328 | 10.64925618 | 11.29582632 | 0.239622187 | 0.367727231 |
| solexa-499-2217 | 11.48316077 | 12.32673844 | 12.28004576 | 12.97835296 | 13.96936853 | 13.94706157 | 14.01553701 | 13.63051741 | 14.09416843 | 8.405566974 | 0.473545581 | 0.743295505 |
| solexa-51-13984 | 8.821773982 | 10.82033854 | 11.02160465 | 11.05372229 | 6.274261661 | 6.196725061 | 6.089582893 | 6.422905743 | 6.10433666 | 5.92243574 | 0.003817793 | -4.260985239 |
| solexa-5169-164 | 6.224001674 | 6.199672345 | 6.590961241 | 6.207502459 | 8.968378801 | 8.60936345 | 9.160249616 | 8.326204751 | 9.098821663 | 7.006746832 | 0.000748522 | 2.222759756 |
| solexa-539-2056 | 12.55837288 | 11.93475974 | 12.96155822 | 13.26034622 | 14.61467534 | 14.45914981 | 14.51607549 | 14.27431991 | 14.51607549 | 14.51607549 | 0.007216341 | 1.803969325 |
| solexa-555-1991 | 13.50039377 | 13.10212335 | 13.26034622 | 11.65745244 | 13.21598794 | 12.58282411 | 13.43564422 | 11.28320413 | 13.05706098 | 13.05706098 | 0.843063814 | -0.108115216 |
| solexa-5620-151 | 7.519243094 | 7.499845887 | 7.269594252 | 6.62935662 | 7.075746538 | 6.465974465 | 7.145677455 | 10.92577695 | 7.408117408 | 6.714245518 | 0.598014474 | 0.393079758 |
| solexa-578-1915 | 12.78426676 | 9.258330473 | 9.927185358 | 9.140318539 | 5.905687849 | 5.911691582 | 5.927185358 | 6.0725346 | 10.61966977 | 6.001126665 | 0.017833283 | -3.537875979 |
| solexa-5874-144 | 6.952450058 | 6.190812368 | 6.334496768 | 6.64096791 | 8.151523994 | 8.710462074 | 9.650154214 | 9.492153626 | 9.328338334 | 7.625708843 | 0.000418821 | 2.296708405 |
| solexa-603-1846 | 8.221103725 | 9.196970898 | 9.885543861 | 9.827660269 | 13.41629614 | 12.46867495 | 13.90966811 | 11.72970571 | 13.73190236 | 13.65089467 | 0.000139209 | 3.868370634 |
| solexa-6676-127 | 6.503030646 | 7.141596278 | 7.764871591 | 6.570614722 | 13.45752196 | 7.316960287 | 7.145677455 | 12.25017997 | 8.11997861 | 7.39145842 | 0.105374907 | 2.285267807 |
| solexa-7111-119 | 6.085339669 | 6.21916852 | 6.199672345 | 7.861707287 | 8.518849829 | 7.921245889 | 8.514911265 | 7.753885283 | 8.597307691 | 11.41494944 | 0.013021731 | 2.195386278 |
| solexa-7297-115 | 8.986979709 | 7.666046429 | 7.469234794 | 7.839833648 | 9.412569847 | 6.465974465 | 7.145677455 | 12.01300855 | 7.117902789 | 10.40290547 | 0.452900809 | 0.769149451 |
| solexa-7509-112 | 7.936637939 | 8.486231678 | 8.189330401 | 8.595070954 | 13.57510193 | 10.58922979 | 11.75768169 | 10.54245153 | 11.86546264 | 10.96079838 | 0.000580685 | 3.246636584 |
| solexa-7534-111 | 7.951867504 | 8.58458675 | 8.633721813 | 8.885696373 | 7.075746538 | 10.06743436 | 5.927185358 | 6.422905743 | 12.53225851 | 7.925109324 | 0.863718011 | -0.188861471 |
| solexa-7764-108 | 11.62858224 | 8.427522371 | 9.034798963 | 10.14095755 | 12.75713995 | 11.849366 | 12.83547806 | 13.57510193 | 12.82348666 | 12.04668067 | 0.021024844 | 2.839910266 |
| solexa-8000-104 | 6.314696526 | 6.918863237 | 7.400025518 | 7.394033895 | 7.641690523 | 12.16091137 | 8.514911265 | 11.72144102 | 7.408117408 | 11.95728346 | 0.025284422 | 2.893821045 |
| solexa-8048-104 | 9.832731815 | 8.618385502 | 8.885696373 | 7.972692654 | 13.92203613 | 13.74706006 | 12.69522829 | 10.06743436 | 13.76677734 | 13.57510193 | 0.000478198 | 4.134896433 |
| solexa-8211-102 | 9.480992104 | 11.68877483 | 11.02873489 | 11.33220442 | 13.63051741 | 11.02517418 | 5.988684687 | 12.97835296 | 6.411087227 | 11.139935 | 0.644331704 | -0.68705132 |
| solexa-826-1288 | 8.92659251 | 8.826389591 | 8.384567923 | 9.421644094 | 12.19614103 | 7.921245889 | 11.02873489 | 6.953032377 | 7.864805194 | 11.6092711 | 0.487478487 | 0.705739884 |
| solexa-8926-93 | 8.247452865 | 8.1017131 | 9.366977836 | 8.781359714 | 12.25017997 | 9.393819448 | 12.41280798 | 9.939285356 | 10.5844928 | 9.885543861 | 0.008767209 | 2.119979023 |
| solexa-9029-92 | 11.68877483 | 11.86546264 | 11.77020962 | 12.14063808 | 14.18441048 | 11.77020962 | 14.23044081 | 12.06062839 | 13.92203613 | 13.80583454 | 0.022392249 | 1.462655369 |
| solexa-9081-91 | 10.8560364 | 10.08308037 | 10.97763769 | 10.34751064 | 13.87028765 | 8.950993232 | 13.41629614 | 13.27035373 | 13.57510193 | 8.543805176 | 0.238226135 | 1.371740034 |
| solexa-9124-90 | 10.50809187 | 8.919459269 | 9.49645436 | 9.164152708 | 13.28896698 | 12.26617162 | 12.12350713 | 13.28896698 | 12.32673844 | 10.51914479 | 0.000918356 | 2.780209773 |
| solexa-9578-86 | 8.975274788 | 8.562624039 | 8.514911265 | 9.223036338 | 13.03414554 | 9.852139328 | 13.40900634 | 11.93475974 | 13.34410169 | 11.48316077 | 0.001367538 | 3.357257295 |
| solexa-9655-85 | 8.885696373 | 9.024308597 | 9.766031913 | 8.512542955 | 10.42311591 | 11.29582632 | 13.03414554 | 13.21598794 | 13.94706157 | 12.12350713 | 0.000891736 | 3.292795777 |
|  |  |  |  |  |  |  |  |  |  |  |  |  |
