# Supplementary material for: Plasma MicroRNAs as Potential Noninvasive Biomarkers for In-Stent Restenosis
Source: PLoS One. 2014 Nov 26;9(11):e112043. doi: 10.1371/journal.pone.0112043 (PMC4245195; doi:10.1371/journal.pone.0112043)
Supplement: Table S3 — List of 10 miRNAs and their target gene. (DOC) [file pone.0112043.s003.doc]

**Table S3. List of 10 miRNAs and their target gene**

| **miRTarBase ID** | **miRNA** | **Species** | **Target Gene** | **Experiments** | **References (PMID)** |
| --- | --- | --- | --- | --- | --- |
| MIRT005913 | hsa-miR-100 | Homo sapiens | ATM | Luciferase reporter assay//qRT-PCR//Western blot | 20869334 |
| MIRT003417 | hsa-miR-100 | Homo sapiens | EGR2 | Microarray//qRT-PCR | 19396866 |
| MIRT003419 | hsa-miR-100 | Homo sapiens | FGFR3 | Luciferase reporter assay//qRT-PCR//Western blot | 19843843 |
| MIRT003418 | hsa-miR-100 | Homo sapiens | ID1 | Microarray//qRT-PCR | 19396866 |
| MIRT003420 | hsa-miR-100 | Homo sapiens | MMP13 | Microarray//qRT-PCR//Western blot | 19396866 |
| MIRT000382 | hsa-miR-100 | Homo sapiens | PLK1 | Luciferase reporter assay//qRT-PCR | 19739117 |
| MIRT004989 | hsa-miR-125b | Homo sapiens | ABCC4 | Microarray | 17891175 |
| MIRT004363 | hsa-miR-125b | Homo sapiens | AKT1 | qRT-PCR//Western blot | 18649363 |
| MIRT005007 | hsa-miR-125b | Homo sapiens | ATXN1 | Microarray | 17891175 |
| MIRT002394 | hsa-miR-125b | Homo sapiens | BAK1 | Luciferase reporter assay//qRT-PCR//Western blot | 18056640 |
| MIRT005915 | hsa-miR-125b | Homo sapiens | BBC3 | Luciferase reporter assay//qRT-PCR//Western blot | 20886540 |
| MIRT003394 | hsa-miR-125b | Homo sapiens | BMF | immunoblot//Luciferase reporter assay//qRT-PCR | 19471102 |
| MIRT000346 | hsa-miR-125b | Homo sapiens | BMPR1B | Luciferase reporter assay | 19738052 |
| MIRT004991 | hsa-miR-125b | Homo sapiens | CASC3 | Microarray | 17891175 |
| MIRT003970 | hsa-miR-125b | Homo sapiens | CBFB | Microarray | 17891175 |
| MIRT004709 | hsa-miR-125b | Homo sapiens | CDKN2A | Western blot | 20347935 |
| MIRT004994 | hsa-miR-125b | Homo sapiens | CGN | Microarray | 17891175 |
| MIRT004408 | hsa-miR-125b | Homo sapiens | CYP24A1 | qRT-PCR//Luciferase reporter assay//Western blot//Northern blot | 19570947 |
| MIRT005503 | hsa-miR-125b | Homo sapiens | E2F3 | Immunohistochemistry//Luciferase reporter assay//Northern blot//qRT-PCR//Western blot | 20549700 |
| MIRT000347 | hsa-miR-125b | Homo sapiens | EIF4EBP1 | Microarray//qRT-PCR | 17891175 |
| MIRT002939 | hsa-miR-125b | Homo sapiens | ERBB2 | Western blot | 19825990 |
| MIRT002938 | hsa-miR-125b | Homo sapiens | ERBB3 | Luciferase reporter assay//Northern blot//qRT-PCR//Western blot | 17110380 |
| MIRT000350 | hsa-miR-125b | Homo sapiens | GLI1 | Luciferase reporter assay | 18756266 |
| MIRT004578 | hsa-miR-125b | Homo sapiens | GRIN2A | Luciferase reporter assay//qRT-PCR | 20159450 |
| MIRT000349 | hsa-miR-125b | Homo sapiens | HMGA1 | Luciferase reporter assay | 17563749 |
| MIRT000348 | hsa-miR-125b | Homo sapiens | HMGA2 | Luciferase reporter assay | 17563749 |
| MIRT005738 | hsa-miR-125b | Homo sapiens | IGF2 | Luciferase reporter assay//Northern blot//Western blot | 21200031 |
| MIRT004534 | hsa-miR-125b | Homo sapiens | IRF4 | qRT-PCR//Luciferase reporter assay | 20497960 |
| MIRT005009 | hsa-miR-125b | Homo sapiens | KCNS3 | Microarray | 17891175 |
| MIRT003421 | hsa-miR-125b | Homo sapiens | KLF13 | Microarray//qRT-PCR//Western blot | 19396866 |
| MIRT004936 | hsa-miR-125b | Homo sapiens | KRT7 | Immunoblot//qRT-PCR | 19378336 |
| MIRT004998 | hsa-miR-125b | Homo sapiens | LACTB | Microarray | 17891175 |
| MIRT001037 | hsa-miR-125b | Homo sapiens | LIF | ELISA | 19011087 |
| MIRT003845 | hsa-miR-125b | Homo sapiens | LIN28A | B-globin reporter assay//Luciferase reporter assay | 16495412 |
| MIRT005804 | hsa-miR-125b | Homo sapiens | LIN28B | Luciferase reporter assay//qRT-PCR//Western blot | 20827722 |
| MIRT004990 | hsa-miR-125b | Homo sapiens | LYPLA2 | Microarray | 17891175 |
| MIRT003997 | hsa-miR-125b | Homo sapiens | MKNK2 | Microarray | 17891175 |
| MIRT000462 | hsa-miR-125b | Homo sapiens | NKIRAS2 | ELISA//qRT-PCR//Luciferase reporter assay//Western blot | 20351193 |
| MIRT004993 | hsa-miR-125b | Homo sapiens | PABPC1 | Microarray | 17891175 |
| MIRT004995 | hsa-miR-125b | Homo sapiens | PLEKHA8 | Microarray | 17891175 |
| MIRT005010 | hsa-miR-125b | Homo sapiens | PPAT | Microarray | 17891175 |
| MIRT004533 | hsa-miR-125b | Homo sapiens | PRDM1 | qRT-PCR//Luciferase reporter assay | 20497960 |
| MIRT004986 | hsa-miR-125b | Homo sapiens | QSOX2 | Microarray | 17891175 |
| MIRT004418 | hsa-miR-125b | Homo sapiens | RAF1 | Western blot | 19825990 |
| MIRT004988 | hsa-miR-125b | Homo sapiens | RNF144A | Microarray | 17891175 |
| MIRT005011 | hsa-miR-125b | Homo sapiens | RPL29 | Microarray | 17891175 |
| MIRT004985 | hsa-miR-125b | Homo sapiens | SAMD10 | Microarray | 17891175 |
| MIRT005008 | hsa-miR-125b | Homo sapiens | SEL1L | Microarray | 17891175 |
| MIRT004997 | hsa-miR-125b | Homo sapiens | SLC35A4 | Microarray | 17891175 |
| MIRT005000 | hsa-miR-125b | Homo sapiens | SLC7A1 | Microarray | 17891175 |
| MIRT004987 | hsa-miR-125b | Homo sapiens | SLC7A6 | Microarray | 17891175 |
| MIRT004999 | hsa-miR-125b | Homo sapiens | SMARCD2 | Microarray | 17891175 |
| MIRT000525 | hsa-miR-125b | Homo sapiens | SMO | Luciferase reporter assay//Western blot | 20216554 |
| MIRT005006 | hsa-miR-125b | Homo sapiens | STAT3 | Microarray | 17891175 |
| MIRT000535 | hsa-miR-125b | Homo sapiens | TP53 | Luciferase reporter assay//Western blot | 20216554 |
| MIRT004996 | hsa-miR-125b | Homo sapiens | TP53INP1 | Microarray | 17891175 |
| MIRT004992 | hsa-miR-125b | Homo sapiens | ULK3 | Microarray | 17891175 |
| MIRT001197 | hsa-miR-125b | Homo sapiens | VDR | Luciferase reporter assay//Western blot | 19437538 |
| MIRT003900 | hsa-miR-130a | Homo sapiens | APP | Luciferase reporter assay | 19110058 |
| MIRT003782 | hsa-miR-130a | Homo sapiens | ATXN1 | Luciferase reporter assay//qRT-PCR//Western blot | 18758459 |
| MIRT004575 | hsa-miR-130a | Homo sapiens | CSF1 | Luciferase reporter assay | 14697198 |
| MIRT005441 | hsa-miR-130a | Homo sapiens | HOXA10 | Luciferase reporter assay//Microarray//qRT-PCR | 20981674 |
| MIRT000958 | hsa-miR-130a | Homo sapiens | HOXA5 | Luciferase reporter assay//qRT-PCR//Western blot//Northern blot | 17957028 |
| MIRT005674 | hsa-miR-130a | Homo sapiens | KLF4 | Luciferase reporter assay//Microarray//qRT-PCR | 20981674 |
| MIRT001141 | hsa-miR-130a | Homo sapiens | MAFB | Luciferase reporter assay | 16549775 |
| MIRT004358 | hsa-miR-130a | Homo sapiens | MEOX2 | Luciferase reporter assay//qRT-PCR//Western blot//Northern blot | 17957028 |
| MIRT005957 | hsa-miR-130a | Homo sapiens | PPARG | GFP reporter assay//qRT-PCR//Western blot | 21135128 |
| MIRT003005 | hsa-miR-130a | Homo sapiens | TAC1 | Luciferase reporter assay | 17855557 |
| MIRT004242 | hsa-miR-143 | Homo sapiens | COL1A1 | Immunoblot//Luciferase reporter assay//qRT-PCR | 19913496 |
| MIRT003964 | hsa-miR-143 | Homo sapiens | COX2 | Microarray | 17875710 |
| MIRT000716 | hsa-miR-143 | Homo sapiens | DNMT3A | qRT-PCR//Luciferase reporter assay//Western blot | 19638978 |
| MIRT003060 | hsa-miR-143 | Homo sapiens | FNDC3B | Luciferase reporter assay//qRT-PCR//Western blot | 19472311 |
| MIRT005771 | hsa-miR-143 | Homo sapiens | FSCN1 | Western blot | 21218087 |
| MIRT005644 | hsa-miR-143 | Homo sapiens | HRAS | Luciferase reporter assay//Northern blot//qRT-PCR | 21276449 |
| MIRT000312 | hsa-miR-143 | Homo sapiens | KRAS | qRT-PCR//Western blot | 19157460 |
| MIRT003759 | hsa-miR-143 | Homo sapiens | MAPK7 | In situ hybridization//qRT-PCR//Western blot | 17504027 |
| MIRT000458 | hsa-miR-143 | Homo sapiens | MYO6 | qRT-PCR//Luciferase reporter assay//Western blot | 20353999 |
| MIRT000305 | hsa-miR-145 | Homo sapiens | BNIP3 | Luciferase reporter assay | 20332243 |
| MIRT005688 | hsa-miR-145 | Homo sapiens | C11orf9 | Luciferase reporter assay | 20737575 |
| MIRT000676 | hsa-miR-145 | Homo sapiens | CBFB | qRT-PCR//Luciferase reporter assay//Microarray | 19915607 |
| MIRT005879 | hsa-miR-145 | Homo sapiens | CDK4 | Luciferase reporter assay//qRT-PCR//Western blot | 21092188 |
| MIRT000575 | hsa-miR-145 | Homo sapiens | CDKN1A | qRT-PCR//Luciferase reporter assay//Western blot | 20190813 |
| MIRT000678 | hsa-miR-145 | Homo sapiens | CLINT1 | qRT-PCR//Luciferase reporter assay//Microarray | 19915607 |
| MIRT005689 | hsa-miR-145 | Homo sapiens | CPEB4 | Luciferase reporter assay | 20737575 |
| MIRT004580 | hsa-miR-145 | Homo sapiens | DFFA | Luciferase reporter assay//qRT-PCR//Western blot | 20687965 |
| MIRT005878 | hsa-miR-145 | Homo sapiens | EIF4E | Luciferase reporter assay//qRT-PCR//Western blot | 21092188 |
| MIRT004496 | hsa-miR-145 | Homo sapiens | FLI1 | qRT-PCR//Luciferase reporter assay//Western blot | 20382729 |
| MIRT003543 | hsa-miR-145 | Homo sapiens | FSCN1 | Luciferase reporter assay//qRT-PCR//Western blot | 20160723 |
| MIRT005690 | hsa-miR-145 | Homo sapiens | FZD7 | Luciferase reporter assay | 20737575 |
| MIRT001917 | hsa-miR-145 | Homo sapiens | HOXA9 | GFP reporter assay//qRT-PCR//Western blot | 18474618 |
| MIRT004616 | hsa-miR-145 | Homo sapiens | IFNB1 | ELISA//Luciferase reporter assay//qRT-PCR | 20130213 |
| MIRT004931 | hsa-miR-145 | Homo sapiens | IGF1R | qRT-PCR//Western blot | 19391107 |
| MIRT000731 | hsa-miR-145 | Homo sapiens | IRS1 | Luciferase reporter assay//Northern blot//qRT-PCR//Western blot | 17827156 |
| MIRT000308 | hsa-miR-145 | Homo sapiens | KLF4 | FACS//Flow//GFP reporter assay//In situ hybridization//Luciferase reporter assay//qRT-PCR | 19409607 |
| MIRT004938 | hsa-miR-145 | Homo sapiens | KRT7 | Immunoblot//qRT-PCR | 19378336 |
| MIRT000426 | hsa-miR-145 | Homo sapiens | MUC1 | Luciferase reporter assay//Western blot | 20407606 |
| MIRT004290 | hsa-miR-145 | Homo sapiens | MYC | qRT-PCR//Luciferase reporter assay//Western blot | 19202062 |
| MIRT000457 | hsa-miR-145 | Homo sapiens | MYO6 | qRT-PCR//Luciferase reporter assay//Western blot | 20353999 |
| MIRT000732 | hsa-miR-145 | Homo sapiens | PARP8 | Luciferase reporter assay | 15131085 |
| MIRT004904 | hsa-miR-145 | Homo sapiens | POU5F1 | FACS//Flow//GFP reporter assay//In situ hybridization//Luciferase reporter assay//qRT-PCR | 19409607 |
| MIRT005683 | hsa-miR-145 | Homo sapiens | PPM1D | Immunoblot//Luciferase reporter assay | 20668064 |
| MIRT000677 | hsa-miR-145 | Homo sapiens | PPP3CA | qRT-PCR//Luciferase reporter assay//Microarray | 19915607 |
| MIRT005809 | hsa-miR-145 | Homo sapiens | ROBO2 | In situ hybridization//Luciferase reporter assay//qRT-PCR//western blot | 21276775 |
| MIRT000307 | hsa-miR-145 | Homo sapiens | SOX2 | FACS//Flow//GFP reporter assay//In situ hybridization//Luciferase reporter assay//qRT-PCR | 19409607 |
| MIRT005810 | hsa-miR-145 | Homo sapiens | SRGAP1 | In situ hybridization//Luciferase reporter assay//qRT-PCR//western blot | 21276775 |
| MIRT000626 | hsa-miR-145 | Homo sapiens | STAT1 | qRT-PCR//Luciferase reporter assay//Western blot//Microarray | 20098684 |
| MIRT004748 | hsa-miR-145 | Homo sapiens | TIRAP | Immunoprecipitaion//Western blot//Communoprecipitaion | 19898489 |
| MIRT001811 | hsa-miR-145 | Homo sapiens | TMOD3 | Luciferase reporter assay | 15131085 |
| MIRT000627 | hsa-miR-145 | Homo sapiens | YES1 | qRT-PCR//Luciferase reporter assay//Western blot//Microarray | 20098684 |
| MIRT001920 | hsa-miR-146a | Homo sapiens | BRCA1 | Luciferase reporter assay | 18660546 |
| MIRT001919 | hsa-miR-146a | Homo sapiens | BRCA2 | Luciferase reporter assay | 18660546 |
| MIRT004017 | hsa-miR-146a | Homo sapiens | CCNA2 | Microarray//qRT-PCR//Western blot | 19944095 |
| MIRT005463 | hsa-miR-146a | Homo sapiens | CD40LG | ELISA//Luciferase reporter assay//qRT-PCR//Western blot | 21236257 |
| MIRT004661 | hsa-miR-146a | Homo sapiens | CDKN1A | Luciferase reporter assay | 20101223 |
| MIRT005705 | hsa-miR-146a | Homo sapiens | CDKN3 | Western blot | 19944095 |
| MIRT000303 | hsa-miR-146a | Homo sapiens | CFH | Western blot | 19540598 |
| MIRT000006 | hsa-miR-146a | Homo sapiens | CXCR4 | qRT-PCR//Luciferase reporter assay//Western blot | 18568019 |
| MIRT004730 | hsa-miR-146a | Homo sapiens | EGFR | qRT-PCR//Western blot | 20124483 |
| MIRT005806 | hsa-miR-146a | Homo sapiens | ERBB4 | Luciferase reporter assay//Microarray//Western blot | 20864407 |
| MIRT000638 | hsa-miR-146a | Homo sapiens | FADD | Luciferase reporter assay | 19965651 |
| MIRT003319 | hsa-miR-146a | Homo sapiens | FAF1 | GFP reporter assay//qRT-PCR | 20459811 |
| MIRT005581 | hsa-miR-146a | Homo sapiens | FAS | Luciferase reporter assay//Western blot | 20656888 |
| MIRT004409 | hsa-miR-146a | Homo sapiens | IL8 | Western blot//Northern blot | 18504431 |
| MIRT000712 | hsa-miR-146a | Homo sapiens | IRAK1 | Western blot//Northern blot | 18504431 |
| MIRT000304 | hsa-miR-146a | Homo sapiens | IRAK2 | qRT-PCR//Western blot | 20124483 |
| MIRT005706 | hsa-miR-146a | Homo sapiens | KIF22 | Western blot | 19944095 |
| MIRT004731 | hsa-miR-146a | Homo sapiens | MTA2 | qRT-PCR//Western blot | 20124483 |
| MIRT004459 | hsa-miR-146a | Homo sapiens | NFKB1 | Luciferase reporter assay | 18504431 |
| MIRT004018 | hsa-miR-146a | Homo sapiens | PA2G4 | Microarray//qRT-PCR//Western blot | 19944095 |
| MIRT000945 | hsa-miR-146a | Homo sapiens | ROCK1 | flow//Western blot//Northern blot | 18174313 |
| MIRT000449 | hsa-miR-146a | Homo sapiens | TLR2 | qRT-PCR//flow//Luciferase reporter assay//Western blot | 20375304 |
| MIRT000711 | hsa-miR-146a | Homo sapiens | TRAF6 | Western blot//Northern blot | 18504431 |
| MIRT005773 | hsa-miR-21 | Homo sapiens | ANKRD46 | Immunoblot//Immunohistochemistry//Luciferase reporter assay//Microarray//qRT-PCR//Western blot | 21219636 |
| MIRT002410 | hsa-miR-21 | Homo sapiens | APAF1 | Luciferase reporter assay//qRT-PCR//Western blot | 18829576 |
| MIRT000173 | hsa-miR-21 | Homo sapiens | BASP1 | Luciferase reporter assay//Quantitative proteomic approach | 19253296 |
| MIRT000159 | hsa-miR-21 | Homo sapiens | BCL2 | Luciferase reporter assay | 17072344 |
| MIRT003837 | hsa-miR-21 | Homo sapiens | BMPR2 | Luciferase reporter assay//qRT-PCR//Western blot | 19578724 |
| MIRT002416 | hsa-miR-21 | Homo sapiens | BTG2 | semi-qRT-PCR//GFP reporter assay//Western blot | 19546886 |
| MIRT005956 | hsa-miR-21 | Homo sapiens | CCR1 | Luciferase reporter assay | 21131358 |
| MIRT000157 | hsa-miR-21 | Homo sapiens | CDC25A | Microarray//Northern blot//qRT-PCR | 19826040 |
| MIRT005974 | hsa-miR-21 | Homo sapiens | CDK2AP1 | Luciferase reporter assay//qRT-PCR//Western blot | 21191961 |
| MIRT005327 | hsa-miR-21 | Homo sapiens | DAXX | Luciferase reporter assay//qRT-PCR//Western blot | 18829576 |
| MIRT000172 | hsa-miR-21 | Homo sapiens | DERL1 | Luciferase reporter assay//Quantitative proteomic approach | 19253296 |
| MIRT001191 | hsa-miR-21 | Homo sapiens | E2F1 | qRT-PCR//Western blot | 19906824 |
| MIRT001121 | hsa-miR-21 | Homo sapiens | E2F2 | Northern blot | 19528081 |
| MIRT005807 | hsa-miR-21 | Homo sapiens | EGFR | Luciferase reporter assay//Microarray//Western blot | 20864407 |
| MIRT004813 | hsa-miR-21 | Homo sapiens | EIF2S1 | Quantitative proteomic approach | 19253296 |
| MIRT005772 | hsa-miR-21 | Homo sapiens | EIF4A2 | Immunoblot//Immunohistochemistry//Luciferase reporter assay//Microarray//qRT-PCR//Western blot | 21219636 |
| MIRT004318 | hsa-miR-21 | Homo sapiens | ERBB2 | qRT-PCR//Western blot | 19419954 |
| MIRT001188 | hsa-miR-21 | Homo sapiens | FMOD | Western blot | 19906824 |
| MIRT005325 | hsa-miR-21 | Homo sapiens | HNRNPK | Luciferase reporter assay//qRT-PCR | 18829576 |
| MIRT005952 | hsa-miR-21 | Homo sapiens | ICAM1 | Luciferase reporter assay//Microarray//qRT-PCR | 21131358 |
| MIRT005951 | hsa-miR-21 | Homo sapiens | IL1B | Luciferase reporter assay//Microarray//qRT-PCR | 21131358 |
| MIRT005717 | hsa-miR-21 | Homo sapiens | ISCU | Immunoblot//Immunohistochemistry//Luciferase reporter assay//qRT-PCR//Western blot | 20480266 |
| MIRT000176 | hsa-miR-21 | Homo sapiens | JAG1 | Flow//Immunoblot//Luciferase reporter assay//Microarray//qRT-PCR | 19398721 |
| MIRT005323 | hsa-miR-21 | Homo sapiens | JMY | Luciferase reporter assay//qRT-PCR | 18829576 |
| MIRT001220 | hsa-miR-21 | Homo sapiens | LRRFIP1 | semi-qRT-PCR//Luciferase reporter assay//Western blot | 19559015 |
| MIRT001221 | hsa-miR-21 | Homo sapiens | MARCKS | Luciferase reporter assay//Western blot | 19302977 |
| MIRT005737 | hsa-miR-21 | Homo sapiens | MEF2C | Immunofluorescence//In situ hybridization//Luciferase reporter assay | 21170291 |
| MIRT005429 | hsa-miR-21 | Homo sapiens | MSH2 | Luciferase reporter assay//Northern blot//qRT-PCR//Western blot | 21078976 |
| MIRT005430 | hsa-miR-21 | Homo sapiens | MSH6 | Luciferase reporter assay//Northern blot//qRT-PCR//Western blot | 21078976 |
| MIRT000961 | hsa-miR-21 | Homo sapiens | MTAP | Western blot//Northern blot | 19013014 |
| MIRT004307 | hsa-miR-21 | Homo sapiens | MYC | Northern blot | 19528081 |
| MIRT000171 | hsa-miR-21 | Homo sapiens | NCAPG | Luciferase reporter assay//Quantitative proteomic approach | 19253296 |
| MIRT004306 | hsa-miR-21 | Homo sapiens | NCOA3 | Luciferase reporter assay | 19528081 |
| MIRT004814 | hsa-miR-21 | Homo sapiens | PCBP1 | Quantitative proteomic approach | 19253296 |
| MIRT003054 | hsa-miR-21 | Homo sapiens | PDCD4 | Luciferase reporter assay//Westren blot | 17968323 |
| MIRT000170 | hsa-miR-21 | Homo sapiens | PDHA2 | Quantitative proteomic approach | 19253296 |
| MIRT005953 | hsa-miR-21 | Homo sapiens | PLAT | Luciferase reporter assay//Microarray//qRT-PCR | 21131358 |
| MIRT000169 | hsa-miR-21 | Homo sapiens | PLOD3 | Luciferase reporter assay//Quantitative proteomic approach | 19253296 |
| MIRT005331 | hsa-miR-21 | Homo sapiens | PPIF | Luciferase reporter assay//qRT-PCR | 18829576 |
| MIRT001190 | hsa-miR-21 | Homo sapiens | PTEN | Western blot | 19672202 |
| MIRT005954 | hsa-miR-21 | Homo sapiens | PTX3 | Luciferase reporter assay | 21131358 |
| MIRT000019 | hsa-miR-21 | Homo sapiens | RASGRP1 | qRT-PCR//Luciferase reporter assay//Western blot | 20483747 |
| MIRT000969 | hsa-miR-21 | Homo sapiens | RECK | Luciferase reporter assay//qRT-PCR//Western blot | 20480266 |
| MIRT000177 | hsa-miR-21 | Homo sapiens | REST | Luciferase reporter assay | 19242418 |
| MIRT003317 | hsa-miR-21 | Homo sapiens | RHOB | Luciferase reporter assay//qRT-PCR//Western blot | 20460403 |
| MIRT000168 | hsa-miR-21 | Homo sapiens | RPS7 | qRT-PCR//Quantitative proteomic approach | 19253296 |
| MIRT000167 | hsa-miR-21 | Homo sapiens | RTN4 | Luciferase reporter assay//Quantitative proteomic approach | 19253296 |
| MIRT003567 | hsa-miR-21 | Homo sapiens | SERPINB5 | Luciferase reporter assay//Western blot | 18270520 |
| MIRT000960 | hsa-miR-21 | Homo sapiens | SOX5 | Western blot//Northern blot | 19013014 |
| MIRT004812 | hsa-miR-21 | Homo sapiens | SPATS2L | Quantitative proteomic approach | 19253296 |
| MIRT000672 | hsa-miR-21 | Homo sapiens | SPRY2 | Luciferase reporter assay | 18508928 |
| MIRT001208 | hsa-miR-21 | Homo sapiens | TGFBI | Luciferase reporter assay//Western blot | 19136465 |
| MIRT001189 | hsa-miR-21 | Homo sapiens | TGFBR2 | qRT-PCR//Western blot | 19906824 |
| MIRT005330 | hsa-miR-21 | Homo sapiens | TGFBR3 | Luciferase reporter assay//qRT-PCR | 18829576 |
| MIRT004287 | hsa-miR-21 | Homo sapiens | TGIF1 | Western blot | 19906824 |
| MIRT005565 | hsa-miR-21 | Homo sapiens | TIAM1 | Luciferase reporter assay//Microarray//Northern blot//qRT-PCR | 20826792 |
| MIRT000954 | hsa-miR-21 | Homo sapiens | TIMP3 | Luciferase reporter assay | 18591254 |
| MIRT000166 | hsa-miR-21 | Homo sapiens | TM9SF3 | Luciferase reporter assay | 19253296 |
| MIRT005955 | hsa-miR-21 | Homo sapiens | TNFAIP3 | Luciferase reporter assay | 21131358 |
| MIRT005324 | hsa-miR-21 | Homo sapiens | TOPORS | Luciferase reporter assay//qRT-PCR | 18829576 |
| MIRT005328 | hsa-miR-21 | Homo sapiens | TP53BP2 | Luciferase reporter assay//qRT-PCR | 18829576 |
| MIRT005329 | hsa-miR-21 | Homo sapiens | TP63 | Luciferase reporter assay//qRT-PCR//Western blot | 18829576 |
| MIRT001980 | hsa-miR-21 | Homo sapiens | TPM1 | Luciferase reporter assay | 18270520 |
| MIRT000164 | hsa-miR-21 | Homo sapiens | WFS1 | Quantitative proteomic approach | 19253296 |
| MIRT000163 | hsa-miR-21 | Homo sapiens | WIBG | Quantitative proteomic approach | 19253296 |
| MIRT003169 | hsa-miR-210 | Homo sapiens | ABCB9 | immunoprecipitaion//Microarray//qRT-PCR | 19826008 |
| MIRT003185 | hsa-miR-210 | Homo sapiens | ACVR1B | immunoprecipitaion//qRT-PCR | 19826008 |
| MIRT003183 | hsa-miR-210 | Homo sapiens | APC | immunoprecipitaion//Microarray//qRT-PCR | 19826008 |
| MIRT003182 | hsa-miR-210 | Homo sapiens | ATP11C | immunoprecipitaion//Microarray//qRT-PCR | 19826008 |
| MIRT003153 | hsa-miR-210 | Homo sapiens | BDNF | 2DGE//immunoprecipitaion//Luciferase reporter assay//Mass spectrometry//Microarray//qRT-PCR//Western blot | 19826008 |
| MIRT003170 | hsa-miR-210 | Homo sapiens | CBX1 | immunoprecipitaion//Microarray//qRT-PCR | 19826008 |
| MIRT003168 | hsa-miR-210 | Homo sapiens | CDK10 | immunoprecipitaion//Microarray//qRT-PCR | 19826008 |
| MIRT003181 | hsa-miR-210 | Homo sapiens | CHD9 | immunoprecipitaion//Microarray//qRT-PCR | 19826008 |
| MIRT003180 | hsa-miR-210 | Homo sapiens | CLASP2 | immunoprecipitaion//Microarray//qRT-PCR | 19826008 |
| MIRT003167 | hsa-miR-210 | Homo sapiens | CPEB2 | immunoprecipitaion//Luciferase reporter assay//Microarray//qRT-PCR | 19826008 |
| MIRT003179 | hsa-miR-210 | Homo sapiens | DDAH1 | immunoprecipitaion//Luciferase reporter assay//Microarray//qRT-PCR | 19826008 |
| MIRT003184 | hsa-miR-210 | Homo sapiens | E2F3 | immunoprecipitaion//qRT-PCR | 19826008 |
| MIRT002024 | hsa-miR-210 | Homo sapiens | EFNA3 | Luciferase reporter assay | 18539147 |
| MIRT003178 | hsa-miR-210 | Homo sapiens | ELK3 | immunoprecipitaion//Microarray//qRT-PCR | 19826008 |
| MIRT003166 | hsa-miR-210 | Homo sapiens | FAM116A | immunoprecipitaion//Microarray//qRT-PCR | 19826008 |
| MIRT000153 | hsa-miR-210 | Homo sapiens | FGFRL1 | Luciferase reporter assay//Microarray//qRT-PCR//Western blot | 21044961 |
| MIRT003165 | hsa-miR-210 | Homo sapiens | GPD1L | immunoprecipitaion//Luciferase reporter assay//Microarray//qRT-PCR | 19826008 |
| MIRT003177 | hsa-miR-210 | Homo sapiens | HECTD1 | immunoprecipitaion//Microarray//qRT-PCR | 19826008 |
| MIRT000152 | hsa-miR-210 | Homo sapiens | HOXA1 | Luciferase reporter assay | 19782034 |
| MIRT003164 | hsa-miR-210 | Homo sapiens | HOXA3 | immunoprecipitaion//Microarray//qRT-PCR | 19826008 |
| MIRT000149 | hsa-miR-210 | Homo sapiens | HOXA9 | Luciferase reporter assay | 19782034 |
| MIRT003163 | hsa-miR-210 | Homo sapiens | ISCU | immunoprecipitaion//Microarray//qRT-PCR | 19826008 |
| MIRT003162 | hsa-miR-210 | Homo sapiens | KIAA1161 | immunoprecipitaion//Microarray//qRT-PCR | 19826008 |
| MIRT003161 | hsa-miR-210 | Homo sapiens | MDGA1 | immunoprecipitaion//Microarray//qRT-PCR | 19826008 |
| MIRT003176 | hsa-miR-210 | Homo sapiens | MIB1 | immunoprecipitaion//Microarray//qRT-PCR | 19826008 |
| MIRT003160 | hsa-miR-210 | Homo sapiens | MID1IP1 | immunoprecipitaion//Microarray//qRT-PCR | 19826008 |
| MIRT003916 | hsa-miR-210 | Homo sapiens | MRE11A | Western blot | 19141645 |
| MIRT003159 | hsa-miR-210 | Homo sapiens | NCAM1 | immunoprecipitaion//Luciferase reporter assay//Microarray//qRT-PCR | 19826008 |
| MIRT003175 | hsa-miR-210 | Homo sapiens | NIPBL | immunoprecipitaion//Microarray//qRT-PCR | 19826008 |
| MIRT001930 | hsa-miR-210 | Homo sapiens | NPTX1 | Luciferase reporter assay | 18539147 |
| MIRT003155 | hsa-miR-210 | Homo sapiens | P4HB | 2DGE//immunoprecipitaion//Mass spectrometry//Microarray//qRT-PCR//Western blot | 19826008 |
| MIRT000151 | hsa-miR-210 | Homo sapiens | PIM1 | Luciferase reporter assay | 19782034 |
| MIRT003174 | hsa-miR-210 | Homo sapiens | PTAR1 | immunoprecipitaion//Microarray//qRT-PCR | 19826008 |
| MIRT003154 | hsa-miR-210 | Homo sapiens | PTPN1 | 2DGE//immunoprecipitaion//Luciferase reporter assay//Mass spectrometry//Microarray//qRT-PCR//Western blot | 19826008 |
| MIRT000156 | hsa-miR-210 | Homo sapiens | RAD52 | Luciferase reporter assay//qRT-PCR//Western blot | 19141645 |
| MIRT003158 | hsa-miR-210 | Homo sapiens | SEH1L | immunoprecipitaion//Microarray//qRT-PCR | 19826008 |
| MIRT003157 | hsa-miR-210 | Homo sapiens | SERTAD2 | immunoprecipitaion//Microarray//qRT-PCR | 19826008 |
| MIRT003173 | hsa-miR-210 | Homo sapiens | SMCHD1 | immunoprecipitaion//Microarray//qRT-PCR | 19826008 |
| MIRT003172 | hsa-miR-210 | Homo sapiens | TNPO1 | immunoprecipitaion//Microarray//qRT-PCR | 19826008 |
| MIRT000150 | hsa-miR-210 | Homo sapiens | TP53I11 | Luciferase reporter assay | 19782034 |
| MIRT003156 | hsa-miR-210 | Homo sapiens | UBQLN1 | immunoprecipitaion//Microarray//qRT-PCR | 19826008 |
| MIRT003171 | hsa-miR-210 | Homo sapiens | XIST | immunoprecipitaion//Luciferase reporter assay//Microarray//qRT-PCR | 19826008 |
| MIRT003917 | hsa-miR-210 | Homo sapiens | XPA | Western blot | 19141645 |
| MIRT003367 | hsa-miR-221 | Homo sapiens | BBC3 | Immunohistochemistry//In situ hybridization//Luciferase reporter assay//Northern blot//Western blot | 20813046 |
| MIRT000141 | hsa-miR-221 | Homo sapiens | BMF | Luciferase reporter assay//qRT-PCR//Western blot | 19671867 |
| MIRT004753 | hsa-miR-221 | Homo sapiens | BNIP3 | Luciferase reporter assay//Microarray//qRT-PCR//Western blot | 20018759 |
| MIRT000137 | hsa-miR-221 | Homo sapiens | CDKN1B | Luciferase reporter assay//Western blot | 17569667 |
| MIRT002272 | hsa-miR-221 | Homo sapiens | CDKN1C | qRT-PCR//Luciferase reporter assay//Western blot | 19153141 |
| MIRT005787 | hsa-miR-221 | Homo sapiens | CORO1A | Western blot | 21226887 |
| MIRT003358 | hsa-miR-221 | Homo sapiens | DDIT4 | Luciferase reporter assay//Microarray//qRT-PCR//Western blot | 20018759 |
| MIRT005320 | hsa-miR-221 | Homo sapiens | ESR1 | Luciferase reporter assay//qRT-PCR//Western blot | 18790736 |
| MIRT004484 | hsa-miR-221 | Homo sapiens | FOS | qRT-PCR//Luciferase reporter assay//Western blot//Northern blot | 20299489 |
| MIRT000434 | hsa-miR-221 | Homo sapiens | FOXO3 | qRT-PCR//ChIP//Luciferase reporter assay//Western blot//Northern blot | 20388878 |
| MIRT004430 | hsa-miR-221 | Homo sapiens | ICAM1 | Luciferase reporter assay//Western blot | 20110463 |
| MIRT001780 | hsa-miR-221 | Homo sapiens | KIT | Luciferase reporter assay//qRT-PCR//Western blot | 19088079 |
| MIRT005295 | hsa-miR-221 | Homo sapiens | NAIP | Western blot | 18759060 |
| MIRT005585 | hsa-miR-221 | Homo sapiens | PTEN | FACS//Flow//Luciferase reporter assay//Northern blot//Western blot | 20618998 |
| MIRT005714 | hsa-miR-221 | Homo sapiens | SELE | Luciferase reporter assay | 19949084 |
| MIRT005788 | hsa-miR-221 | Homo sapiens | SSSCA1 | Western blot | 21226887 |
| MIRT005789 | hsa-miR-221 | Homo sapiens | TCEAL1 | Western blot | 21226887 |
| MIRT005475 | hsa-miR-221 | Homo sapiens | TICAM1 | Luciferase reporter assay//Northern blot//qRT-PCR//Western blot | 21236259 |
| MIRT005785 | hsa-miR-221 | Homo sapiens | TP53 | Western blot | 21226887 |
| MIRT004981 | hsa-miR-31 | Homo sapiens | ARPC5 | Luciferase reporter assay//qRT-PCR | 19524507 |
| MIRT004597 | hsa-miR-31 | Homo sapiens | CASR | LacZ assay | 20145132 |
| MIRT004980 | hsa-miR-31 | Homo sapiens | CXCL12 | Luciferase reporter assay//qRT-PCR | 19524507 |
| MIRT005875 | hsa-miR-31 | Homo sapiens | DACT3 | Immunoprecipitaion//Luciferase reporter assay//Microarray//qRT-PCR//Western blot | 21048943 |
| MIRT005874 | hsa-miR-31 | Homo sapiens | DKK1 | Immunoprecipitaion//Luciferase reporter assay//Microarray//qRT-PCR//Western blot | 21048943 |
| MIRT005456 | hsa-miR-31 | Homo sapiens | DMD | Luciferase reporter assay//qRT-PCR//Western blot | 21212803 |
| MIRT004975 | hsa-miR-31 | Homo sapiens | ETS1 | Luciferase reporter assay//qRT-PCR | 19524507 |
| MIRT001180 | hsa-miR-31 | Homo sapiens | FOXP3 | qRT-PCR//Luciferase reporter assay//Western blot | 19408243 |
| MIRT004984 | hsa-miR-31 | Homo sapiens | FZD3 | Luciferase reporter assay//qRT-PCR//Western blot | 19524507 |
| MIRT004974 | hsa-miR-31 | Homo sapiens | HOXC13 | Luciferase reporter assay//qRT-PCR | 19524507 |
| MIRT005707 | hsa-miR-31 | Homo sapiens | ICAM1 | Luciferase reporter assay | 19949084 |
| MIRT004976 | hsa-miR-31 | Homo sapiens | ITGA5 | Luciferase reporter assay//qRT-PCR//Western blot | 19524507 |
| MIRT004973 | hsa-miR-31 | Homo sapiens | JAZF1 | Luciferase reporter assay//qRT-PCR | 19524507 |
| MIRT004972 | hsa-miR-31 | Homo sapiens | KLF13 | Luciferase reporter assay//qRT-PCR | 19524507 |
| MIRT000491 | hsa-miR-31 | Homo sapiens | LATS2 | qRT-PCR//Luciferase reporter assay | 20237410 |
| MIRT004978 | hsa-miR-31 | Homo sapiens | MMP16 | Luciferase reporter assay//qRT-PCR//Western blot | 19524507 |
| MIRT004977 | hsa-miR-31 | Homo sapiens | MPRIP | Luciferase reporter assay//qRT-PCR | 19524507 |
| MIRT004971 | hsa-miR-31 | Homo sapiens | NFAT5 | Luciferase reporter assay//qRT-PCR | 19524507 |
| MIRT004970 | hsa-miR-31 | Homo sapiens | NUMB | Luciferase reporter assay//qRT-PCR | 19524507 |
| MIRT000490 | hsa-miR-31 | Homo sapiens | PPP2R2A | qRT-PCR//Luciferase reporter assay | 20237410 |
| MIRT004979 | hsa-miR-31 | Homo sapiens | RDX | Luciferase reporter assay//qRT-PCR//Western blot | 19524507 |
| MIRT004969 | hsa-miR-31 | Homo sapiens | RET | Luciferase reporter assay//qRT-PCR | 19524507 |
| MIRT000088 | hsa-miR-31 | Homo sapiens | RHOA | Luciferase reporter assay//qRT-PCR//Western blot | 19524507 |
| MIRT004019 | hsa-miR-31 | Homo sapiens | SELE | Immunocytochemistry//Northern blot//qRT-PCR//Western blot | 19949084 |
| MIRT005566 | hsa-miR-31 | Homo sapiens | TIAM1 | Luciferase reporter assay//Microarray//Northern blot//qRT-PCR | 20826792 |
| MIRT004968 | hsa-miR-31 | Homo sapiens | YY1 | Luciferase reporter assay//qRT-PCR | 19524507 |
|  |  |  |  |  |  |
